# Regio‐ and Stereoselective Homologation of 1,2‐Bis(Boronic Esters): Stereocontrolled Synthesis of 1,3‐Diols and Sch 725674

**DOI:** 10.1002/anie.201608406

**Published:** 2016-10-26

**Authors:** Alexander Fawcett, Dominik Nitsch, Muhammad Ali, Joseph M. Bateman, Eddie L. Myers, Varinder K. Aggarwal

**Affiliations:** ^1^ School of ChemistryUniversity of BristolCantock's CloseBristolBS8 1TSUK; ^2^ Department of ChemistryCOMSATS Institute of Information TechnologyUniversity RoadAbbottabad-22060, KPKPakistan

**Keywords:** 1,3-diols, diboration, homologation, lithiation, Sch 725674

## Abstract

1,2‐Bis(boronic esters), derived from the enantioselective diboration of terminal alkenes, can be selectively homologated at the primary boronic ester by using enantioenriched primary/secondary lithiated carbamates or benzoates to give 1,3‐bis(boronic esters), which can be subsequently oxidized to the corresponding secondary‐secondary and secondary‐tertiary 1,3‐diols with full stereocontrol. The transformation was applied to a concise total synthesis of the 14‐membered macrolactone, Sch 725674. The nine‐step synthetic route also features a novel desymmetrizing enantioselective diboration of a divinyl carbinol derivative and high‐yielding late‐stage cross‐metathesis and Yamaguchi macrolactonization reactions.

Developments in the homologation of boronic esters has continued unabated for almost 40 years, reaching a point where iterative homologation (“one pot”) of a simple boronic ester into a molecule bearing 10 contiguous methyl substituents with full stereocontrol was recently demonstrated.^1^ Its use in complex natural product synthesis, such as (+)‐10‐hydroxyphthioceranic acid, has also been demonstrated (Scheme 1 a).^2^ The methodology clearly works well in the construction of carbon chains rich in non‐polar residues, but these represent a rather small sub‐set of natural products. Most natural products contain polar residues where 1,3‐hydroxy groups are ubiquitous. However, whilst 1,3‐hydroxy groups can be easily prepared from alternative starting materials (e.g. carbonyl compounds),^3^ they cannot be prepared from boronic esters because the intermediate boronate complex that would be required to generate this moiety can either undergo the desired 1,2‐migration or undesired β‐elimination (Scheme 1 b). When X is a halide (Matteson homologation) or a carbamate (our work), the desired 1,2‐migration does occur but β‐elimination often competes limiting its efficiency and generality, thus rendering these common motifs inaccessible to the current methodology.^4^ To address this problem, we considered masking the β‐alkoxy group with another boron atom (Scheme 1 c) because 1) the β‐boronic ester does not undergo elimination,^5^ 2) oxygen functionality can be readily generated, and 3) the required 1,2‐bis(boronic esters) are readily available through Morken/Nishiyama catalytic asymmetric diboration of terminal alkenes.^6^ The new process would require regio‐ and stereoselective homologation of the 1,2‐bis(boronic ester).^7^, ^8^ By combining Morken/Nishiyama diboration with lithiation–borylation we now show that powerful new methodology can be generated, enabling incorporation of the 1,3‐diol motif through boronic ester homologation. In addition, we demonstrate its application in the concise total synthesis of the 14‐membered macrolactone, Sch 725674.

**Scheme 1:**
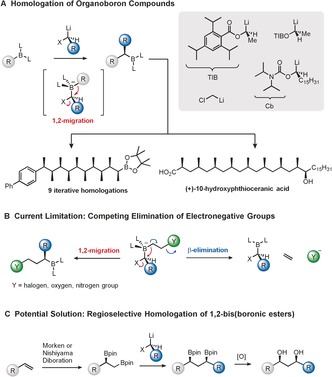
Homologation of organoboron compounds.

The key selective homologation of a primary boronic ester over a secondary boronic ester was initially examined. A 1,2‐bis(boronic ester), (*R*)‐**2** (e.r. 95:5, 1.2 equiv), which was prepared by Morken asymmetric diboration of 1‐octene,6c was added to a solution of preformed sparteine‐ligated lithiated carbamate **1 a**‐Li‐(+)‐sp (1.0 equiv, >99:1 e.r.)^9^ at −78 °C. Subsequent heating at 35 °C for 16 h, followed by oxidation (H_2_O_2_/NaOH/H_2_O), gave the desired 1,3‐diol (*S*,*S*)‐**3** (66 % yield) together with only trace amounts of the double‐addition product, that is, that derived from homologation of both the primary and secondary boronic esters (Scheme 2 a, entry 1). The use of the lithiated triisopropylbenzoate (**1 b**‐Li‐(+)‐sp 1.0 equiv, 96:4 e.r.)^10^ in place of the carbamate gave similar yields of **3** (69 % yield; Scheme 2 a, entry 4).

**Scheme 2:**
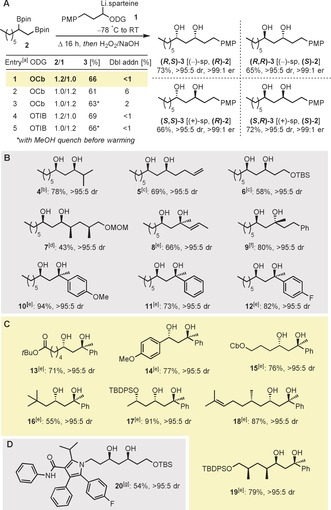
Selective homologation of 1,2‐bis(boronic esters): optimization and scope. Yields given are of isolated product, d.r. values were determined by using ^13^C NMR spectroscopy. [a] 0.55 mmol of the limiting reagent was used; **1**, *s*‐BuLi, (+)‐ or (−)‐sparteine, Et_2_O (0.2 m), −78 °C; then **2** (1 m in Et_2_O), −78 °C, 1 h; for ODG=OCb: warm to RT, then 35 °C overnight; for ODG=OTIB: warm to RT; 3 m aq. NaOH/30 % aq. H_2_O_2_ (2:1), THF, 0 °C to RT. [b] Reaction conditions: entry 4. [c] Reaction conditions: entry 1. [d] Reaction conditions: entry 2. [e] Reaction conditions: entry 1; sparteine was not used; MgBr_2_ in MeOH was added prior to warming. [f] Reaction conditions: entry 4; TMEDA was used in place of sparteine. [g] 0.28 mmol of the TIB ester (0.33 m) and 0.14 mmol of the 1,2‐bis(boronic ester) was used. DG=directing group, Cb=*N*,*N*‐diisopropyl carbamoyl, TIB=triisopropylbenzoate, TMEDA=tetramethylethylenediamine.

Because the enantioenriched 1,2‐bis(boronic ester) can sometimes be more valuable than the lithium‐stabilized carbenoid, we were keen on identifying conditions where the latter could be used in excess. However, standard conditions led to increased amounts of the double‐addition product (Scheme 2 a, entry 2). Suspecting that this product was only generated while the reaction mixture was being warmed from −78 °C to room temperature, we performed an experiment using excess carbamate and where MeOH was added to the reaction mixture immediately prior to warming, thus protonating any remaining lithiated carbamate.^11^ Pleasingly, the use of this methanol‐quench protocol, for both the carbamate and the benzoate, gave a good yield of **3** and only trace amounts of the double‐addition product (Scheme 2 a, entries 3 and 5), thus supporting our hypothesis and completing a suite of conditions for the regio‐ and stereoselective homologation of 1,2‐bis(boronic esters).

The selectivity of this transformation may seem unremarkable: the less hindered primary boronic ester reacts in preference to the secondary boronic ester. However, we have found that it is critically dependent on the nature of the nucleophile. For example, the use of the either the TMEDA‐ligated or diamine‐free lithiated carbamate **1 a**, or chloromethyl lithium (all less‐hindered) gave a mixture of starting material, mono‐ and double‐addition products (see the Supporting Information for details). Thus, only by using suitably hindered diamine‐ligated lithiated carbamate **1 a** or benzoate **1 b** can high selectivity for reaction of the primary boronic ester over the secondary boronic ester be achieved.

With these conditions established, we prepared the remaining three stereoisomers of **3** by using the appropriate enantiomer of both 1,2‐bis(boronic esters) **2** (1.2 equiv) and lithiated carbamate **1 a**‐Li (Scheme 2 A). In all cases, diols **3** were obtained with the same high diastereoselectivity and yield showing that there were no matched/mis‐matched effects and that the reactions were dominated by reagent control. The scope of the selective transformation was also explored. The 1,2‐bis(boronic ester), (*R*)‐**2**, was treated with a range of lithiated primary and alkyl‐alkyl, alkyl‐aryl, and alkyl‐vinyl secondary carbamates/benzoates to give the corresponding secondary‐secondary and secondary‐tertiary 1,3‐diols in moderate to good yield and with excellent levels of diastereo‐ and enantioselectivity (**4**–**12**, Scheme 2 B). By using the secondary benzylic carbamate, in combination with a range of 1,2‐bis(boronic esters) of different steric demand bearing commonly encountered functional groups (ester, silyl ether, carbamate, alkene), the 1,3‐diols were again obtained with high regio‐ and stereoselectivity (**13**–**19**, Scheme 2 C). The ability to prepare secondary‐tertiary 1,3‐diols in any stereoisomeric form with such high selectivity is especially notable because such a transformation is unprecedented.^12^ Finally, a TBS‐protected derivative of the lipid‐lowering drug, atorvastatin, was prepared in good yield and excellent levels of stereoselectivity by using the corresponding pyrrole‐containing 1,2‐bis(boronic ester) and lithiated benzoate containing the primary TBS ether (**20**, Scheme 2 D), demonstrating further scope and potential application.

We decided to showcase this methodology in a total synthesis of Sch 725674 (**21**; Figure 1), a 14‐membered macrolactone, which was isolated in 2005 from *Aspergillus* sp.^13^ The molecule exhibits moderate antifungal activity and is a rare example of a macrocyclic polyketide natural product that does not contain any methyl‐group branching. It is a popular target that has been frequently used to demonstrate methodology,^14^, ^15^ as it is a representative of a much larger class of important macrolactone polyketide‐derived enolides.^16^ Our retrosynthesis involves a novel desymmetrizing diboration of a divinylcarbinol derivative (**25**→**22**, Figure 1), setting the C4 and C5 stereocenters, followed by two reagent‐controlled C−C bond‐forming lithiation–borylation reactions on a dicarbenoid precursor, **24**, the first being a regioselective transformation of the primary boronic ester of **22** and the second installing the pentyl‐substituted C13 carbinol. Cognizant of previous syntheses of Sch 725674,^14^, ^15^ we decided on cross‐metathesis/macrolactonization to incorporate C1 and C2 and form the ring.

**Figure 1:**
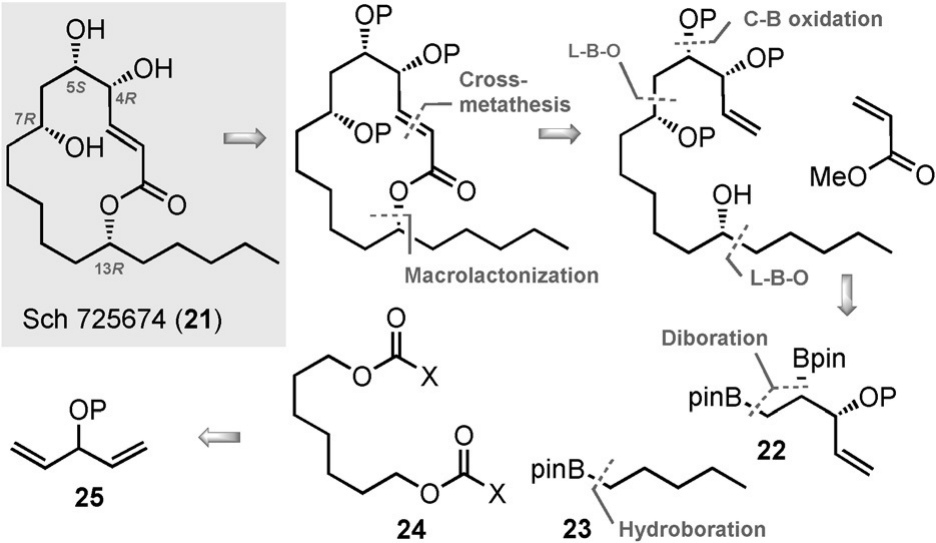
Retrosynthetic analysis of Sch 725674. L‐B‐O: lithiation–borylation–oxidation.

We began by investigating the novel desymmetrizing asymmetric diboration of divinyl carbinol derivatives. We found that *O*‐silyl derivatives, such as **25 a**, gave very high yields of the single‐diboration product (**27**) using Morken's conditions, which were obtained as single diastereomers (>95:5 d.r.; Scheme 3). Nishiyama's conditions and other more coordinating *O*‐protecting groups including the free alcohol were also tested but they performed less well (see the Supporting Information for details), so the TBS‐protected derivative **27** (90 % yield, >95:5 d.r.) was taken forward. Conversion of 1,2‐bis(boronic ester) **27** to the known triol (C−B oxidation/TBS deprotection), and then to the tri(*p*‐bromobenzoyl ester) confirmed both the identity of the major diastereomer as being *anti* and the high levels of enantioselectivity (98:2 e.r.).

**Scheme 3:**
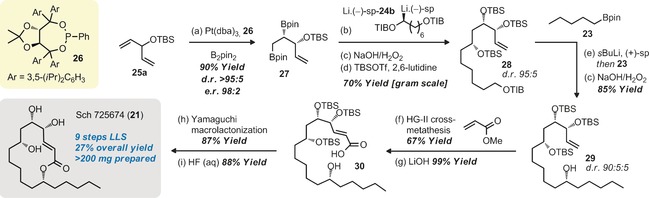
Synthesis of Sch 725674. Reaction conditions: a) Pt(dba)_3_ (1 mol %), **26** (1.2 mol %), B_2_pin_2_ (1.1 equiv), THF, 60 °C, 16 h. b) **24 b** (1.3 equiv), *s*BuLi (1.2 equiv), (−)‐sp. (1.3 equiv), Et_2_O, −78 °C, 2 h; then **27** (1.0 equiv), −78 °C, 1 h; then 35 °C, 16 h. c) 2 m aq. NaOH/30 % aq. H_2_O_2_ (2:1), THF, RT, 1 h. d) TBSOTf (4.1 equiv), 2,6‐lutidine (6.2 equiv), CH_2_Cl_2_, RT, 1.5 h. e) **28** (1.0 equiv), *s*BuLi (1.2 equiv), (+)‐sp. (1.3 equiv), Et_2_O, −78 °C, 2 h; then **23** (1.4 equiv), −78 °C, 1 h; then 35 °C, 16 h. f) Hoveyda–Grubbs 2^nd^ gen cat. (10 mol %), methyl acrylate (3.0 equiv), EtOAc, 80 °C, 16 h. g) LiOH (10 equiv), THF/MeOH/H_2_O (1:1:1), 40 °C, 16 h. h) trichlorobenzoyl chloride (1.2 equiv), NEt_3_ (3.0 equiv), toluene, RT, 4 h; then DMAP (2.0 equiv), 80 °C, 16 h. i) HF (48 wt %, H_2_O)/CH_2_Cl_2_/CH_3_CN (1:2:6), RT, 3 h.

With a significant amount of **27** in hand, we moved to the other coupling partner **24 a** (X=N(*i*Pr)_2_, Figure 1). Unfortunately the bis‐carbamate did not undergo lithiation under standard deprotonation conditions (*s*BuLi, diamine, Et_2_O, −78 °C), but the corresponding bis(triisopropylbenzoate) **24 b** (X=2,3,5‐triisopropylbenzene, Figure 1) did. Thus, enantioselective deprotonation of **24 b** with *s*BuLi (1.2 equiv) in Et_2_O in the presence of (−)‐sparteine (1.3 equiv) at −78 °C, followed by addition of 1,2‐bis(boronic ester) **27**, warming to room temperature, an oxidative workup, and TBS protection of the resulting secondary hydroxy groups gave the tris‐TBS protected 1,2,4‐triol **28** in 70 % yield on multigram scale (Scheme 3). The diastereoselectivity of the transformation was ca. 95:5, which is in line with the levels of enantioselectivity we often obtain for sparteine‐mediated deprotonation of primary triisopropyl‐benzoates.^11^ The reaction was selective for transformation of the primary boronic ester; we did not observe any products arising from homologation of the secondary boronic ester or homologation of both boronic esters. The remaining benzoate ester was then reacted with pentyl boronic ester **23** in another lithiation–borylation–oxidation to give tris(*tert*‐butylsilyl)‐protected tetraol **29**, which was isolated in 85 % yield as a 90:5:5 mixture of diastereomers. The diastereopurity of **29** again is in line with the expected reagent‐controlled ≈95:5 selectivity in the sparteine‐mediated deprotonation of **28**, imposed on a 95:5 mixture of diastereomers of **28**. This reaction was scaled up to provide grams of material (2.0 g). The terminal alkene was then converted into the α,β‐unsaturated methyl ester through ruthenium‐catalyzed cross‐metathesis with methyl acrylate. This reaction initially proved rather difficult, presumably due to the steric hindrance surrounding the terminal alkene. However, by slow dropwise addition of a solution of the second‐generation Hoveyda–Grubbs catalyst (10 mol %) to the reaction mixture, which was maintained at 80 °C, over a period of 19 hours the product was obtained in good yield (67 %).^17^ Conducting the reaction at ambient temperature, having the full amount of the catalyst present at the beginning of the reaction or by adding it in portions gave poor yields of the cross‐metathesis product. These results were suggestive of a catalyst decomposition pathway that was at least second order in catalyst concentration.^18^ Following hydrolysis of the methyl ester, which could be isolated in diastereomerically pure form, macrolactonization of seco‐acid **30** was accomplished using Yamaguchi conditions giving the hydroxy‐protected macrocycle in 87 % yield.^19^ All three TBS groups were then removed by treatment with aq. HF/MeCN/CH_2_Cl_2_,^20^ thus giving the target compound, Sch 725674 (**21**) in 88 % yield.

In conclusion, we have demonstrated that 1,2‐bis(boronic esters) derived from the asymmetric Morken/Nishiyama diboration of terminal alkenes, undergo regio‐ and stereoselective homologation of the primary boronic ester, in the presence of enantioenriched lithiated carbamates or benzoates, to give stereodefined 1,3‐bis(boronic esters), which can be oxidized to the corresponding 1,3‐diol. This merging of asymmetric diboration with lithiation–borylation overcomes the long‐standing difficulty in homologating β‐alkoxy boronic esters, thus allowing lithiation–borylation to be used for preparing highly oxygenated target molecules, including secondary‐tertiary 1,3‐diols, for which there have been no generally applicable synthetic routes. We employed this methodology in a very short (9 steps LLS), high‐yielding and scalable synthesis of Sch 725674. The synthesis was additionally enabled by a novel desymmetrizing diboration of divinyl carbinols, the products of which should prove to be highly useful intermediates in synthesis.

## Supporting information

As a service to our authors and readers, this journal provides supporting information supplied by the authors. Such materials are peer reviewed and may be re‐organized for online delivery, but are not copy‐edited or typeset. Technical support issues arising from supporting information (other than missing files) should be addressed to the authors.

Supplementary
