# Supplementary material for: Regio‐ and Stereoselective Homologation of 1,2‐Bis(Boronic Esters): Stereocontrolled Synthesis of 1,3‐Diols and Sch 725674
Source: Angew Chem Int Ed Engl. 2016 Oct 26;55(47):14663–7. doi: 10.1002/anie.201608406 (PMC5129522; doi:10.1002/anie.201608406)

Angewandte Chemie
Eine Zeitschrift der Gesellschaft Deutscher Chemiker

Supporting Information

Regio- and Stereoselective Homologation of 1,2-Bis(Boronic Esters): Stereocontrolled Synthesis of 1,3-Diols and Sch 725674

*Alexander Fawcett[+], Dominik Nitsch[+], Muhammad Ali[+], Joseph M. Bateman, Eddie L. Myers, and Varinder K. Aggarwal**

anie_201608406_sm_miscellaneous_information.pdf

Contents

1. General Experimental 2
1.1 Solvents and Reagents 2
1.2 Chromatography and Spectroscopy 2
1.3 Naming of compounds 3
1.4 The Fawcett Flask 3
2. Optimization Studies: Regioselective Homologation of 1,2-bis(boronic esters) 5
2.1 Synthesis of starting materials 5
2.2 Why the need for a hydroxyl surrogate? 14
2.2.1 Previous group results 14
2.2.2 β-Elimination Studies 14
2.2.3 Non-Selective homologations of 1,2-bis(boronic esters) 17
2.3 Regioselective Homologation of 1,2-bis(boronic ester) development 20
2.4 Final Procedures 27
2.5 Rationalizing the regioselectivity 33
3. Regioselective Homologation of 1,2-bis(boronic esters): Substrate Scope 36
3.1 Synthesis of Starting Materials 36
3.1 Carbenoid Scope 54
3.2 1,2-Bis(Boronic Ester) Scope 65
4. Diboration of divinyl carbinols 76
3.1 Synthesis of starting materials 76
3.2 Diboration optimization 79
3.3 Proof of absolute stereochemistry of 6d 86
4. Total synthesis of Sch725674 89
4.1 Synthesis of starting materials 89
3.2 Linear synthesis of Sch725674 91
5. NMR Data 99

1. General Experimental

1.1 Solvents and Reagents

All air and water-sensitive reactions were carried out in flame-dried glassware under a nitrogen atmosphere using standard Schlenk manifold technique. Bulk solutions were evaporated under reduced pressure using a Büchi rotary evaporator. All solvents were commercially supplied or provided by the communal stills of the School of Chemistry, University of Bristol. Anhydrous diethyl ether, toluene, tetrahydrofuran and dichloromethane were dried using a purification column composed of activated alumina and stored over thoroughly dried 3 Å mol sieves.[1] (+)-Sparteine and (–)-sparteine were obtained from the commercially available sulfate pentahydrate salt (99%, Acros) and isolated according to literature procedure.[2] The sparteine free base readily absorbs atmospheric carbon dioxide (CO_2) and so should be stored under argon/nitrogen at −20 °C in a sealed Schlenk-tube. *sec*-BuLi was purchased from Acros as a 1.3 M solution in cyclohexane:hexane 98:2. The molarity of organolithium solutions were determined by titration using *N*-benzyl benzamide as an indicator.[3] All other reagents were purchased from commercial sources and used as received.

1.2 Chromatography and Spectroscopy

Flash column chromatography was carried out using Sigma Aldrich silica gel 60 (40–63 μm). All reactions were followed by thin-layer chromatography (TLC) when practical, using Merck Kieselgel 60 F254 fluorescent treated silica which was visualised under UV light, by staining with 5% solution of phosphomolybdic acid ($H_3PMo_{12}O_{40}$) in EtOH followed by heating, or by staining with an aqueous solution of $KMnO_4$ followed by heating.

^{1}H and ^{13}C NMR spectra were recorded using Jeol ECS 400 MHz, Varian VNMR 400 MHz and Varian VNMR 500 MHz spectrometers. Chemical shifts (δ) are given in parts per million (ppm), and coupling constants (J) are given in Hertz (Hz), rounded to the nearest 0.1 Hz. The ^{1}H NMR spectra are reported as follows: ppm (multiplicity, coupling constants, number of protons, assignment). NMR assignments are made according to spin systems, using two dimensional (COSY, HSQC, HMBC) NMR spectroscopy to assist the assignment. Where an

---

[1] A.B. Pangborn, M.A. Giardello, R.H. Grubbs, R.K. Rosen and F.J. Timmers, *Organometallics* **1996**, 15, 1518.
[2] N.A. Nikolic and P. Beak, *Org. Synth.* **1997**, 74, 23.
[3] A.F. Burchat, J.M. Chong and N. Nielsen, *J. Organomet. Chem.* **1997**, 542, 281.

assignment could not be made unambiguously, possible assignments are given. ^{13}C signals adjacent to boron are generally not observed due to quadrupolar relaxation.

High resolution mass spectra (HRMS) were recorded on a Brüker Daltonics MicrOTOF II by Electrospray Ionisation (ESI) or a Brüker Daltonics UltrafleXtreme (MALDI). IR spectra were recorded on a Perkin Elmer Spectrum One FT-IR as a thin film. Only selected absorption maxima (ν_{max}) are reported. Optical rotation ($[\alpha]_D^T$) was measured on a Bellingham and Stanley Ltd. ADP220 polarimeter. Chiral high performance liquid chromatography (HPLC) separations were performed on an Agilent 1100 Series HPLC unit equipped with UV-vis diode-array detector monitored at 210.8 nm, using a Daicel Chiralpak IA column (4.6 × 250 mm^2 , 5 μm) fitted with its respective guard (4 × 10 mm^2). Chiral GC was performed on an Agilent 7890A using Chiraldex columns (30 m x 0.25 mm x 0.25 μm). Supercritical fluid chromatography (SFC) was performed on a Thar SFC investigator using a Daicel Chiralpak Whelk-01 (4.6 × 250 mm^2, 5 μm).

1.3 Naming of compounds

Compound names are those generated by ChemBioDraw 13.0 software (PerkinElmer), following the IUPAC nomenclature.

1.4 The Fawcett Flask

The Fawcett Flask was first conceived by Alexander Fawcett as a means to create a simple method to add a stirred solution at a cryogenic temperature to a second stirred solution at the same temperature. The final form was settled upon after many discussions with Professor Varinder K. Aggarwal, Dr Eddie L. Myers and Dr Daniel J. Blair. All Fawcett Flasks were constructed by the University of Bristol School of Chemistry glassblower, Duncan Tarling. Alternative names are: Aggarwal Inverse-Addition Vessel, Tipping Flask, Myers Mixer and the Tarling Tipper.

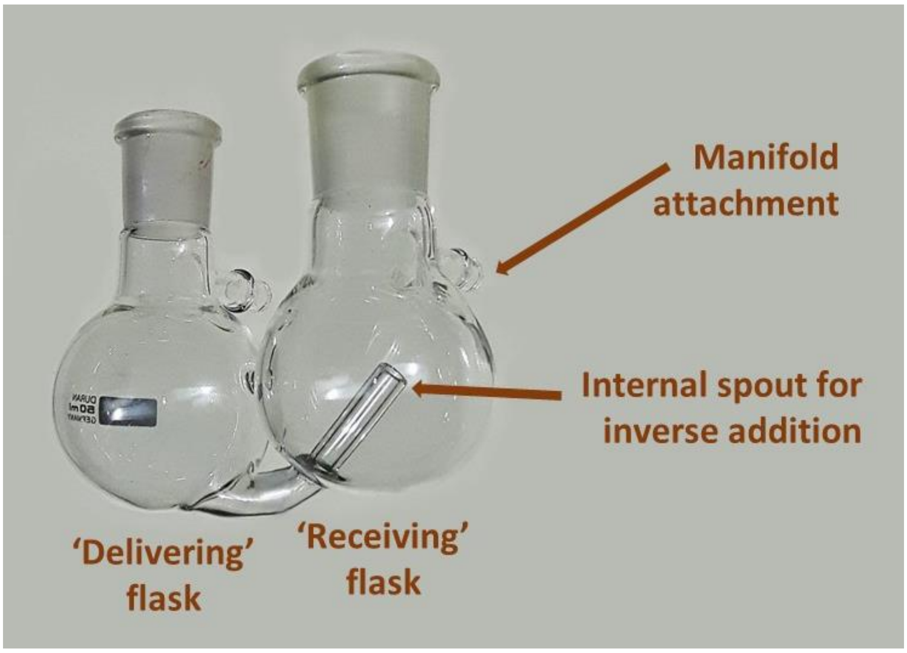

Figure 1. The Fawcett Flask – custom glassware for the addition of solutions at cryogenic temperatures to a second solution at the same temperature.

Operation: After flame-drying the flask is attach to a Schlenk manifold, and the two ground-glass joints are stoppered with suba-seals, before applying a high vacuum until the glassware has cooled to ambient temperature. The necessary reagents and solvents are then added into the appropriate sides of the flask (we typically add the reagents as a solution to the receiving flask a few minutes prior to transfer as it can be difficult to control the stirring in both halves when the flask is clamped into position). The flask can then be comfortably lowered into a cooling bath for the required reaction time. To perform the inverse-addition procedure the flask needs to be unclamped (we have found that the flask can sit happily in a cooling bath without the need for clamping for short periods of time) and held at an angle to allow proper stirring in both halves. Simple tipping of the flask, without removing either side from the cooling bath, will allow the solution in the delivering half to pour across into the receiving half. We have found that it is easy to control the rate of addition, so that it is comparable to dropwise or small portion-wise addition. To pour the final few drops across it is necessary to close the Schlenk tap on the side of the receiving flask and insert a needle into the suba-seal of the receiving flask. A finger over the end of the needle is sufficient to control the rate of addition of these final few drops.

2. Optimization Studies: Regioselective Homologation of 1,2-bis(boronic esters)

2.1 Synthesis of starting materials

3-(4-Methoxyphenyl)propyl diisopropylcarbamate (**1a**), 3-(4-methoxyphenyl)propyl 2,4,6-triisopropylbenzoate (**1b**)[4], 2-hexyl-4,4,5,5-tetramethyl-1,3,2-dioxaborolane (**31**)[5], isobutyl 2,4,6-triisopropylbenzoate (**32**)[6] and (*R*)-3-(4-methoxyphenyl)-1-(tributylstannyl)propyl diisopropylcarbamate (**33**)[7] were prepared according to published procedures. All collected data matched that reported.

***tert*-Butyldimethyl((1-(4,4,5,5-tetramethyl-1,3,2-dioxaborolan-2-yl)heptyl)oxy)silane (34):**

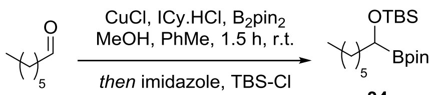

According to modified literature procedures,[8] ICy.HCl (173 mg, 0.64 mmol, 3 mol%), copper(I) chloride (64 mg, 0.64 mmol, 3 mol%) and sodium *tert*-butoxide (124 mg, 1.29 mmol, 6 mol%) were added to a flame-dried Schlenk-tube purged with argon. Anhydrous toluene (6 mL) was added before stirring the reaction for 5 min. B_2pin_2 (5.45 g, 21.46 mmol, 1.00 eq.) and anhydrous toluene (15 mL) were then added before stirring for a further 10 min. Freshly distilled heptanal (3.00 mL, 21.46 mmol, 1.00 eq.) and anhydrous methanol (1.74 mL, 42.93 mmol, 2.00 eq.) were finally added before stirring the mixture for 1.5 h at ambient temperature. The mixture was quickly passed through a wetted plug of silica, eluting with Et_2O (40 mL). The combined solutions were diluted with CH_2Cl_2 (40 mL) and then imidazole (4.38 g, 64.39 mmol, 3.00 eq.) and TBSCl (3.56 g, 23.61 mmol, 1.10 eq.) were added before stirring for 3 h. The reaction mixture was passed through a wetted plug of silica, eluting with Et_2O (100 mL), and concentrated under reduced pressure. The crude residue was purified by flash column chromatography (SiO_2; 98:2 pentane:Et_2O) to yield **34** (2.07 g, 27%) as a colorless oil.

TLC: R_f = 0.52 (95:5 pentane:Et_2O)

---

[4] R. Larouche-Gautier, T.G. Elford and V.K. Aggarwal, *J. Am. Chem. Soc.* **2011**, *133*, 16794.
[5] C.-T. Yang, Z.-Q. Zhang, H. Tajuddin, C.-C. Wu, J. Liang, J.-H. Liu, Y. Fu, M. Czyzewska, P. G. Steel, T. B. Marder and L. Liu, *Angew. Chem. Int. Ed.* **2012**, *51*, 528.
[6] J. L.Y. Chen, H. K. Scott, M. J. Hesse, C. L. Willis and V. K. Aggarwal, *J. Am. Chem. Soc.* **2013**, *135*, 5316.
[7] M. J. Dearden, C. R. Firkin, J.-P. R. Hermet and P. B'Brien, *J. Am. Chem. Soc.* **2002**, *124*, 11870.
[8] (a) K. Kubota, E. Yamamoto and H. Ito, *J. Am. Chem. Soc.* **2015**, *137*, 420. (b) G. A. Molander and S.R. Wisniewski, *J. Am. Chem. Soc.* **2012**, *134*, 16856.

^{1}H NMR: (400 MHz, $CDCl_3$) δ 3.51 (dd, J = 7.9, 6.0 Hz, 1H, CHOSi), 1.64 – 1.47 (m, 2H, CH_2CHOSi), 1.47 – 1.03 (m, 8H, 4×CH_2), 1.26 (s, 6H, $C(CH_3)_2$), 1.24 (s, 6H, $C(CH_3)_2$), 0.89 s, 9H, $SiC(CH_3)_3$), 0.87 (t, J = 7.0 Hz, 3H, CH_3), 0.05 (s, 3H, $SiCH_3$), 0.04 (s, 3H, $SiCH_3$) ppm

^{13}C NMR: (101 MHz, $CDCl_3$) δ 83.7 ($C(CH_3)_2$), 34.7 (CH_2), 32.0 (CH_2), 29.5 (CH_2), 26.5 (CH_2), 26.2 ($SiC(CH_3)_3$), 25.2 ($C(CH_3)_2$), 24.7 ($C(CH_3)_2$), 22.8 (CH_2), 18.7 ($SiC(CH_3)_3$), 14.2 (CH_3), –4.7 ($SiCH_3$), –4.8 ($SiCH_3$) ppm

HRMS (*m/z*): (ESI) calc'd for $C_{19}H_{41}BO_3SiNa$ $[M+Na]^+$: 379.2814, found: 379.2827

IR (neat) ν_{max}: 2956, 2928, 2856, 1334, 1252, 1146, 1084, 834 and 773 cm^{-1}

***tert*-Butyldimethyl((1-(4,4,5,5-tetramethyl-1,3,2-dioxaborolan-2-yl)octan-2-yl)oxy)silane (35):**

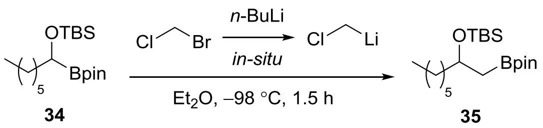

A solution of **34** (140 mg, 0.39 mmol, 1.00 eq.) and bromochloromethane (0.08 mL, 1.18 mmol, 3.00 eq.) in anhydrous Et_2O (2.0 mL) was cooled to –78 °C (dry ice/acetone) before adding *n*-BuLi (0.61 mL, 1.60 M, 0.98 mmol, 2.50 eq.) dropwise. After the addition of *n*-BuLi was complete the mixture was allowed to stir for 30 min at the same temperature before warming to ambient temperature. The reaction mixture was filtered through a wetted plug of silica, eluting with Et_2O (10 mL), and concentrating under reduced pressure to yield **35** (153 mg, >95%) as a colorless oil.

TLC: R_f = 0.48 (95:5 pentane:Et_2O)

^{1}H NMR: (400 MHz, $CDCl_3$) δ 3.97 – 3.87 (m, 1H, CHO), 1.50 – 1.14 (m, 10H, 5×CH_2), 1.23 (s, 6H, $C(CH_3)_2$), 1.22 (s, 6H, $C(CH_3)_2$), 1.13 – 1.02 (m, 2H, CHBpin), 0.90 – 0.83 (m, 12H, CH_3 and $SiC(CH_3)_3$), 0.05 (s, 3H, $SiCH_3$), 0.04 (s, 3H, $SiCH_3$) ppm

^{13}C NMR: (101 MHz, $CDCl_3$) δ 83.0 ($C(CH_3)_2$), 70.1 (CHO), 39.5 (CH_2), 32.0 (CH_2), 29.5 (CH_2), 26.1 ($SiC(CH_3)_3$), 25.7 (CH_2), 25.2 ($C(CH_3)_2$), 24.8 ($C(CH_3)_2$), 22.8 (CH_2), 18.3 ($SiC(CH_3)_3$), 14.3 (CH_3), –4.1 ($SiCH_3$), –4.3 ($SiCH_3$) ppm

HRMS (*m/z*): (ESI) calc'd for $C_{20}H_{43}BO_3SiNa$ $[M+Na]^+$: 393.2971, found: 393.2980

IR (neat) ν_{max}: 2956, 2928, 2857, 1371, 1319, 1253, 1146, 834 and 772 cm^{-1}

2,2'-(Octane-1,2-diyl)bis(4,4,5,5-tetramethyl-1,3,2-dioxaborolane) (2)[9]

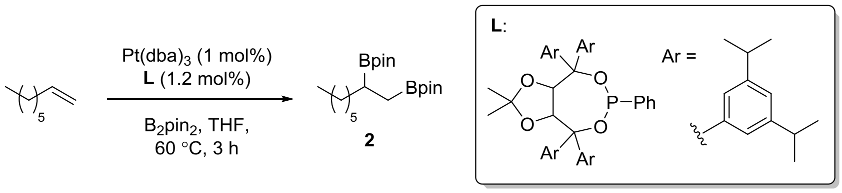

$Pt(dba)_3$ (286.1 mg, 0.32 mmol, 1.0 mol%), **L** (347.6 mg, 0.38 mmol, 1.2 mol%) and B_2pin_2 (8.50 g, 33.5 mmol, 1.05 eq.) were added to a flame-dried Schlenk-tube purged with N_2. THF (32 mL) was added before sealing the flask and heating at 80 °C (oil bath) for 30 mins. After cooling to ambient temperature 1-octene (5.00 mL, 31.86 mmol, 1.00 eq.) was added before re-sealing and heating for 3 hr at 60 °C (oil bath). The solution was then cooled to ambient temperature and concentrated under reduced pressure. The crude residue was directly purified by flash column chromatography (SiO_2; 95:5 pentane:Et_2O) to yield the enantioenriched 1,2-bis(boronic esters) as pale yellow oils.

For (*R*)-2 (using *R*,*R*-L): 7.52 g, 64%

For (*S*)-2 (using *S*,*S*-L): 7.98 g, 68%

TLC: R_f = 0.28 (90:10 pentane:Et_2O)

^{1}H NMR: (400 MHz, $CDCl_3$) δ 1.22 (m, 1H, CH_2, CHBpin), 1.22 (s, 12H, 2×$C(CH_3)_2$), 1.22 (s, 12H, 2×$C(CH_3)_2$), 0.86 (dd, J = 15.8, 7.9 Hz, 1H, CH^aCH^bBpin), 0.86 (t, J = 6.9 Hz, 3H, CH_3), 0.78 (dd, J = 15.8, 5.9 Hz, 1H, CH^aCH^bBpin) ppm

^{13}C NMR: (101 MHz, $CDCl_3$) δ 82.9 ($C(CH_3)_2$), 82.9 ($C(CH_3)_2$), 34.0 (CH_2), 32.0 (CH_2), 29.7 (CH_2), 29.0 (CH_2), 25.1 ($C(CH_3)_2$), 25.0 ($C(CH_3)_2$), 24.9 ($C(CH_3)_2$), 24.9 ($C(CH_3)_2$), 22.8 (CH_2), 14.2 (CH_3) ppm

HRMS (*m/z*): (ESI) calc'd for $C_{20}H_{40}B_2O_4Na$ $[M+Na]^+$: 389.3012, found: 389.3019

IR (neat) ν_{max}: 2978, 2924, 2855, 1370, 1310, 1140, 968 and 846 cm^{-1}

---

[9] J.R. Coombs, F. Haeffner, L.T. Kliman and J.P. Morken, *J. Am. Chem. Soc.* **2013**, 135, 11222.

For (*R*)-2 (using *R*,*R*-L), $[\alpha]_D^{24}$: –9.0 (*c* = 10.0, $CHCl_3$)

For (*S*)-2 (using *S*,*S*-L), $[\alpha]_D^{24}$: +10.0 (*c* = 10.0, $CHCl_3$)

Racemic 1,2-bis(boronic ester) *rac*-**2** was synthesised using the following procedure:[10]

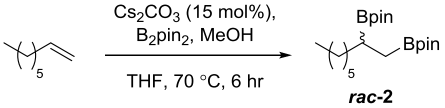

***rac*-2**

Cs_2CO_3 (1.56 g, 4.78 mmol, 15 mol%) and B_2pin_2 (8.90 g, 35.1 mmol, 1.10 eq.) were added to a flame-dried Schlenk-tube purged with N_2. THF (125 mL), MeOH (6.45 mL, 159 mmol, 5.00 eq.) and 1-octene (5.00 mL, 31.9 mmol, 1.00 eq.) were then added and the mixture was heated at 70 °C (oil bath) for 6 hr. The reaction mixture was cooled to ambient temperature and concentred under reduced pressure. The crude residue was directly purified by flash column chromatography (SiO_2; 95:5 pentane:Et_2O) to yield the racemic 1,2-bis(boronic ester) (7.87 g, 67%) as a colorless oil.

The collected data was identical to that described above.

Aliquots of the reaction mixtures were oxidised and protected as acetonides[6] for analysis of enantiomeric purity by chiral-GC.

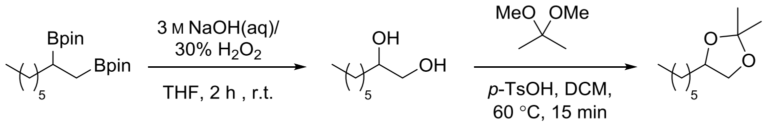

[10] A. Bonet, C. Pubill-Ulldemolins, C. Bo. H. Gulyás and E. Fernández, *Angew. Chem. Int. Ed.* **2011**, 50, 7158.

Chiral-GC: Chiraldex β-DM, injector T = 250 °C, detector T = 300 °C. Oven conditions: T = 80 °C for 5 min, then ramp (1 °C/min) until 100 °C, then ramp (20 °C/min) until 180 °C and hold for 5 min. He carrier gas at 2.0 mL/min.

Racemic:

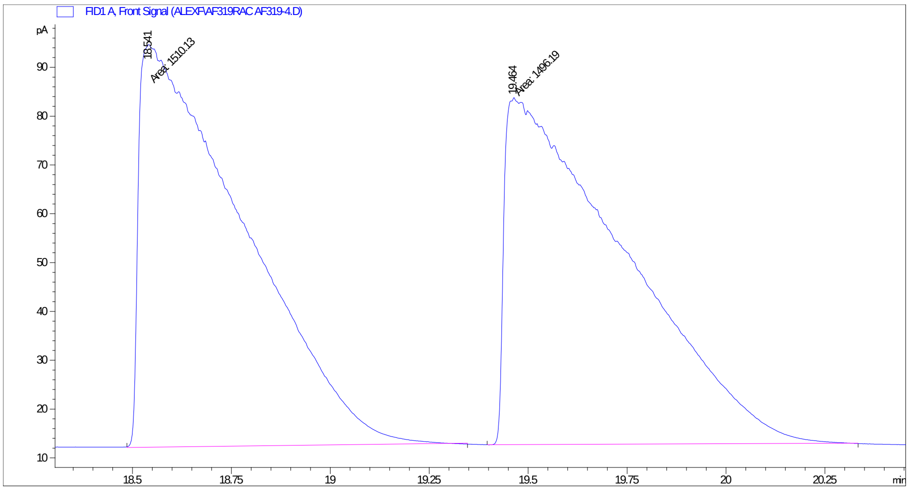

For (R)-**2** (using (R,R)-**L**): 95.3:4.7 e.r. [t_R= 19.2 min (minor), t_R = 18.8 min (major)]

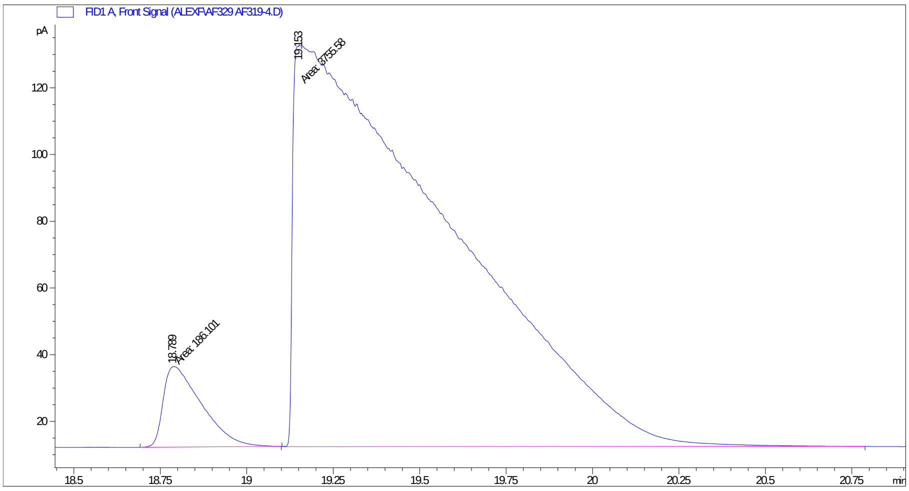

For (*S*)-**2** (using (*S*,*S*)-**L**): 96.5:3.5 e.r. [t_R = 19.1 min (minor), t_R = 20.1 min (major)]

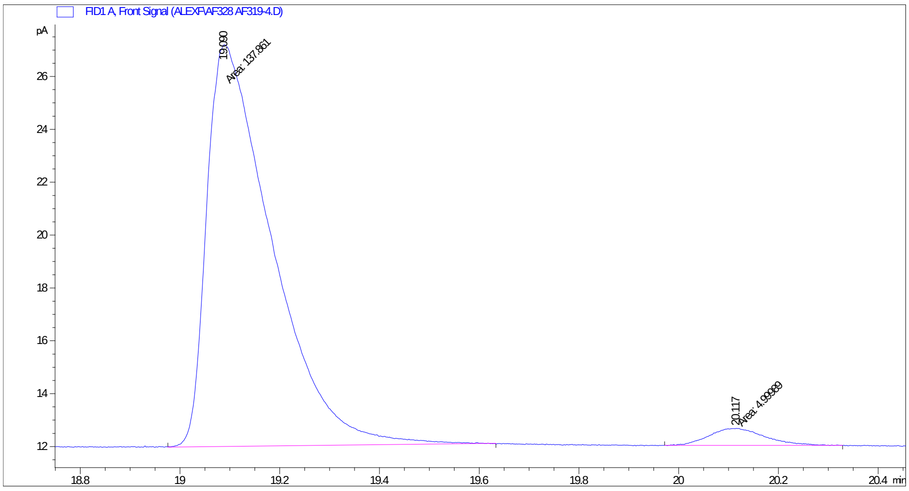

Isopentyl 2,4,6-triisopropylbenzoate (36):

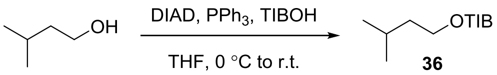

DIAD (5.96 mL, 30.29 mmol, 1.10 eq.) was added drop-wise over 10 min to a stirred solution of PPh_3 (7.94 g, 30.29 mmol, 1.10 eq.), 3-methyl-1-butanol (3.00 mL, 27.53 mmol, 1.00 eq.) and 2,4,6-triisopropylbenzoic acid (7.86 g, 31.66 mmol, 1.15 eq.) in anhydrous THF (41 mL) at 0 °C (ice/water). After warming slowly to ambient temperature over 16 h the solvent was removed under reduced pressure. Pentane (50 mL) was added and the mixture stirred for 10 min. The white suspension was filtered and the filter cake washed with pentane (50 mL). The solvent was removed under reduced pressure and the crude residue was purified by flash column chromatography (SiO_2; 99.75:0.25 pentane:Et_2O) to yield **36** (7.55 g, 86%) as a colorless oil.

TLC: R_f = 0.17 (99.75:0.25 pentane:Et_2O)

^{1}H NMR: (400 MHz, $CDCl_3$) δ 7.02 (s, 2H, ArH), 4.35 (t, *J* = 6.8 Hz, 2H, CH_2O), 2.95 – 2.82 (m, 3H, ArC*H*(CH_3)$_2$), 1.85 – 1.70 (m, 1H, CH_2C*H*(CH_3)$_2$), 1.63 (q, *J* = 6.8 Hz, 2H, C*H*$_2$$CH_2O$),

1.26 (d, J = 6.9 Hz, 12H, 2×CH(CH_3)$_2$), 1.26 (d, J = 7.0 Hz, 6H, CH(CH_3)$_2$), 0.96 (d, J = 6.6 Hz, 6H, CH_2CH(CH_3)$_2$) ppm

^{13}C NMR: (101 MHz, $CDCl_3$) δ 171.2 (C=O), 150.2 (ArC), 144.9 (ArC), 130.9 (ArC), 121.0 (ArH), 63.6 (CH_2O), 37.5 (CH_2), 34.6 (Ar*C*H(CH_3)$_2$), 31.6 (Ar*C*H(CH_3)$_2$), 25.1 (CH_2*C*H), 24.3 (2×ArCH(*C*H_3)$_2$), 24.1 (ArCH(*C*H_3)$_2$), 22.5 (CH_2CH(*C*H_3)$_2$) ppm

HRMS (*m/z*): (ESI) calc'd for $C_{21}H_{34}O_2Na$ [M+Na]$^+$: 341.2451, found: 341.2459

IR (neat) ν_{max}: 2959, 2870, 1724 (CO), 1607, 1462, 1250, 1136, 1103, 1075 and 876 cm^{-1}

4,4,5,5-Tetramethyl-2-(2-methyldecan-4-yl)-1,3,2-dioxaborolane (37):

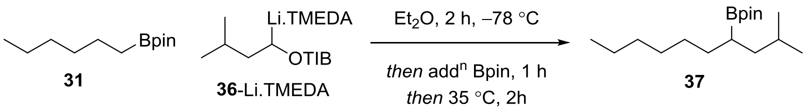

sec-BuLi (5.44 mL, 1.30 m, 7.08 mmol, 1.50 eq.) was added drop-wise to a solution of **36** (2.26 g, 7.08 mmol, 1.50 eq.) and TMEDA (1.06 mL, 7.08 mmol, 1.50 eq.) in anhydrous Et_2O (35 mL) at −78 °C (dry ice/acetone). The resultant mixture was stirred at this temperature for 2 h before adding 2-hexyl-4,4,5,5-tetramethyl-1,3,2-dioxaborolane (**31**) (1.00 g, 4.72 mmol, 1.00 eq.) dropwise over 20 min. After allowing to stir for a further 1 h the mixture was allowed to warm to ambient temperature before heating at 35 °C (oil bath) for 2 h. HCl (30 mL, 1.0 M aqueous solution) was added and the phases separated. The aqueous phase was extracted with Et_2O (30 mL) and the combined organic phases were dried over $MgSO_4$, filtered and concentrated under reduced pressure. The crude residue was purified by flash column chromatography (SiO_2; pentane) to yield **37** (555 mg, 42%) as a colorless oil.

TLC: R_f = 0.38 (98:2 pentane:Et_2O)

^{1}H NMR: (400 MHz, $CDCl_3$) δ 1.60 – 1.50 (m, 1H, C*H*(CH_3)$_2$), 1.43 – 0.97 (m, 13H, 6×CH_2 and CHBpin), 1.23 (s, 12H, C(CH_3)$_2$), 0.89 – 0.85 (m, 9H, CH(CH_3)$_2$ and CH_3) ppm

^{13}C NMR: (101 MHz, $CDCl_3$) δ 82.9 (*C*(CH_3)$_2$), 41.0 (CH_2), 32.0 (CH_2), 31.9 (CH_2), 29.8 (CH_2), 29.4 (CH_2), 27.5 (*C*H(CH_3)$_2$), 25.0 (C(*C*H_3)$_2$), 24.9 (C(*C*H_3)$_2$), 23.3 (CH(*C*H_3)$_2$), 22.8 (CH_2), 22.7 (CH(*C*H_3)$_2$), 14.3 (CH_3) ppm

HRMS (*m/z*): (EI) 239 ([M–^{i}Pr]$^+$, 33%), 267 ([M–Me]$^+$, 100%), 283 ([M+H]$^+$, 7%). *Despite repeated attempts, a HRMS could not be obtained.*

IR (neat) ν_{max}: 2956, 2924, 2854, 1467, 1379, 1314, 1253, 1143, 967 and 862 cm^{-1}

4,4,5,5-Tetramethyl-2-(2-methylnonan-3-yl)-1,3,2-dioxaborolane (38):

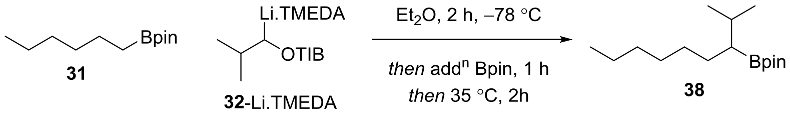

sec-BuLi (5.44 mL, 1.30 m, 7.08 mmol, 1.50 eq.) was added drop-wise to a solution of isobutyl 2,4,6-triisopropylbenzoate (**32**) (2.15 g, 7.07 mmol, 1.50 eq.) and TMEDA (1.06 mL, 7.07 mmol, 1.50 eq.) in anhydrous Et_2O (35 mL) at –78 °C (dry ice/acetone). The resulting mixture was stirred at this temperature for 2 h before adding 2-hexyl-4,4,5,5-tetramethyl-1,3,2-dioxaborolane (**31**) (1.00 g, 4.71 mmol, 1.00 eq.) dropwise over 20 min. After allowing to stir for a further 1 h the mixture was allowed to warm to ambient temperature before heating at 35 °C (oil bath) for 2 h. HCl (30 mL, 1.0 M aqueous solution) was added and the phases separated. The aqueous phase was extracted with Et_2O (30 mL) and the combined organic phases were dried over $MgSO_4$, filtered and concentrated under reduced pressure. The crude residue was purified by flash column chromatography (SiO_2; 99:1 pentane:CH_2Cl_2) to yield **38** (513 mg, 41%) as a colorless oil.

TLC: R_f = 0.15 (99:1 pentane:CH_2Cl_2)

^{1}H NMR: (400 MHz, $CDCl_3$) δ 1.75 – 1.63 (m, 1H, C*H*(CH_3)$_2$), 1.46 – 1.14 (m, 10H, 5×CH_2), 1.25 (s, 12H, C(CH_3)$_2$), 0.92 (d, *J* = 6.7 Hz, 3H, CH(CH_3)$_2$), 0.91 (d, *J* = 6.7 Hz, 3H, CH(CH_3)$_2$), 0.87 (t, *J* = 6.8 Hz, 3H, CH_3), 0.85 – 0.78 (m, 1H, CHBpin) ppm

^{13}C NMR: (101 MHz, $CDCl_3$) δ 82.9 (*C*(CH_3)$_2$), 32.0 (CH_2), 29.8 (*C*H(CH_3)$_2$ and CH_2), 29.8 (CH_2), 29.4 (CH_2), 25.1 (C(*C*H_3)$_2$), 25.0 (C(*C*H_3)$_2$), 22.8 (CH_2), 22.5 (CH(*C*H_3)$_2$), 22.0 (CH(*C*H_3)$_2$), 14.23 (CH_3) ppm

HRMS (*m/z*): (EI) 253 ([M–Me]$^+$, 100%), 269 ([M+H]$^+$, 16%). *Despite repeated attempts, a HRMS could not be obtained.*

IR (neat) ν_{max}: 2956, 2925, 2857, 1466, 1379, 1312, 1213, 1144 and 969 cm^{-1}

2-(2-Isopropyloctyl)-4,4,5,5-tetramethyl-1,3,2-dioxaborolane (39):

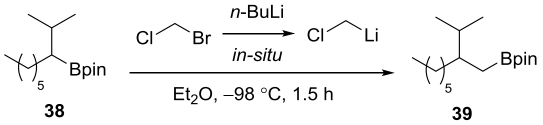

A solution of **38** (486 mg, 1.81 mmol, 1.00 eq.) and bromochloromethane (0.35 mL, 5.44 mmol, 3.00 eq.) in anhydrous Et_2O (12 mL) was cooled to –98 °C (liquid N_2/MeOH) before adding *n*-BuLi (2.83 mL, 1.60 M, 4.53 mmol, 2.50 eq.) dropwise. After the addition of *n*-BuLi was complete the mixture was allowed to stir for 1.5 h at the same temperature before warming to ambient temperature. The reaction mixture was filtered through a wetted plug of silica, eluting with Et_2O (20 mL), and concentrating under reduced pressure to yield **39** (511 mg, >95%) as a colorless oil.

TLC: R_f = 0.16 (pentane)

^{1}H NMR: (400 MHz, $CDCl_3$) δ 1.68 – 1.58 (m, 1H, C*H*(CH_3)$_2$), 1.53 – 1.44 (m, 1H, C*H*CH(CH_3)$_2$), 1.35 – 1.08 (m, 10H, 5×CH_2), 1.24 (s, 12H, C(CH_3)$_2$), 0.87 (t, *J* = 6.5 Hz, 3H, CH_3), 0.84 (d, *J* = 6.8 Hz, 3H, CH(C*H*$_3$)$_2$), 0.79 (d, *J* = 6.8 Hz, 3H, CH(C*H*$_3$)$_2$), 0.74 (dd, *J* = 15.4, 5.9 Hz, 1H, C$H^a$$H^b$Bpin), 0.63 (dd, *J* = 15.4, 8.3 Hz, 1H, C$H^a$$H^b$Bpin) ppm

^{13}C NMR: (101 MHz, $CDCl_3$) δ 82.9 (*C*(CH_3)$_2$), 40.2 (*C*HCH(CH_3)$_2$), 33.8 (CH_2), 32.1 (CH_2), 31.6 (*C*H(CH_3)$_2$), 30.0 (CH_2), 27.6 (CH_2), 25.0 (C(*C*H$_3$)$_2$), 24.9 (C(*C*H$_3$)$_2$), 22.9 (CH_2), 20.0 (CH(*C*H$_3$)$_2$), 18.5 (CH(*C*H$_3$)$_2$), 14.3 (CH_3) ppm

HRMS (*m/z*): (EI) 239 ([M–^{i}Pr]$^+$, 100%), 267 ([M–Me]$^+$, 39%). *Despite repeated attempts, a HRMS could not be obtained.*

IR (neat) ν_{max}: 2957, 2927, 2872, 1466, 1371, 1317, 1146, 967 and 848 cm^{-1}

2.2 Why the need for a hydroxyl surrogate?

2.2.1 Previous group results[11]

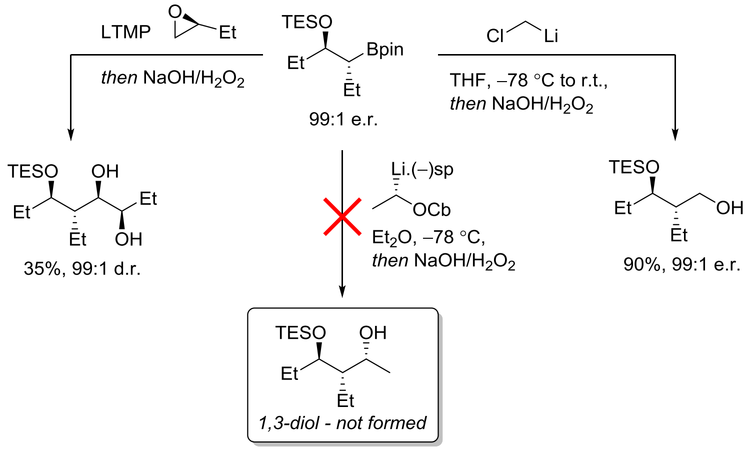

2.2.2 β-Elimination Studies

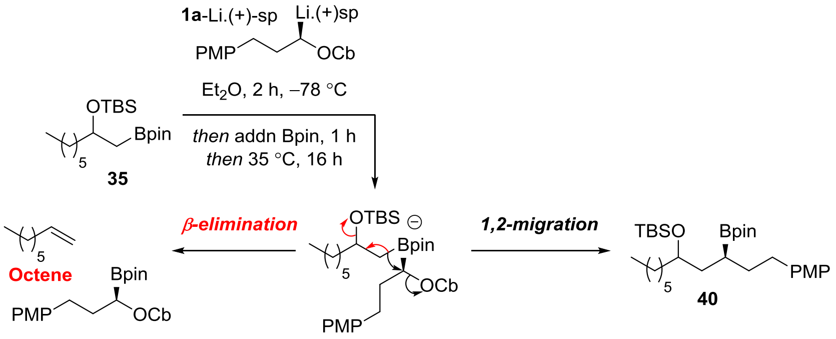

sec-BuLi (0.51 mL, 1.30 M, 0.66 mmol, 1.20 eq.) was added dropwise to a solution of **1a** (203 mg, 0.69 mmol, 1.25 eq.) and (+)-sparteine (0.16 mL, 0.69 mmol, 1.25 eq.) in anhydrous Et_2O (3.46 mL) at –78 °C (dry ice/acetone). After 2 h and solution of **35** (205 mg, 0.55 mmol, 1.00 eq.) in anhydrous Et_2O (0.55 mL) was added dropwise and the resulting solution was allowed to react for a further 1 h. The solution was warmed to ambient temperature and then heated at 35 °C (oil bath) for 16 h. After cooling to ambient temperature 1,3,5-trimethoxybenzene (internal standard) (30.8 mg, 0.183 mmol) was added, and then a 0.7 mL sample of the reaction mixture was analysed by ^{1}H NMR. This revealed octene (26%) as a side-product, which confirms β-elimination can occur. Water (10 mL) was added and the reaction mixture was extracted with Et_2O (3×10 mL). The combined organic fractions were dried over $MgSO_4$, filtered and concentrated under reduced pressure. The crude residue was purified by flash column chromatography (SiO2; 98:2 pentane:Et_2O) to yield the 1,2-migration product **40** (176 mg, 62%, mixture of diastereomers) as a colorless oil.

[11] E. Vedrenne, O.A. Wallner, M. Vitale, F. Schmidt and V.K. Aggarwal, *Org. Lett.* **2009**, 11, 165.

^{1}H NMR showing octene:

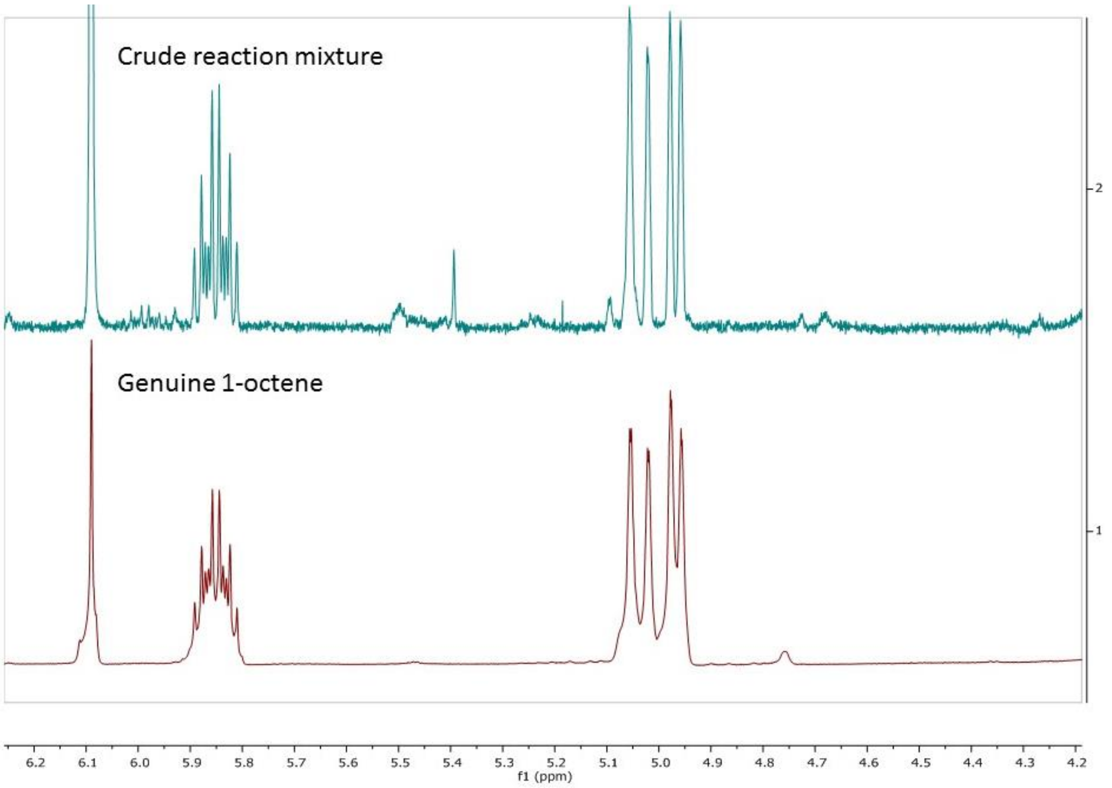

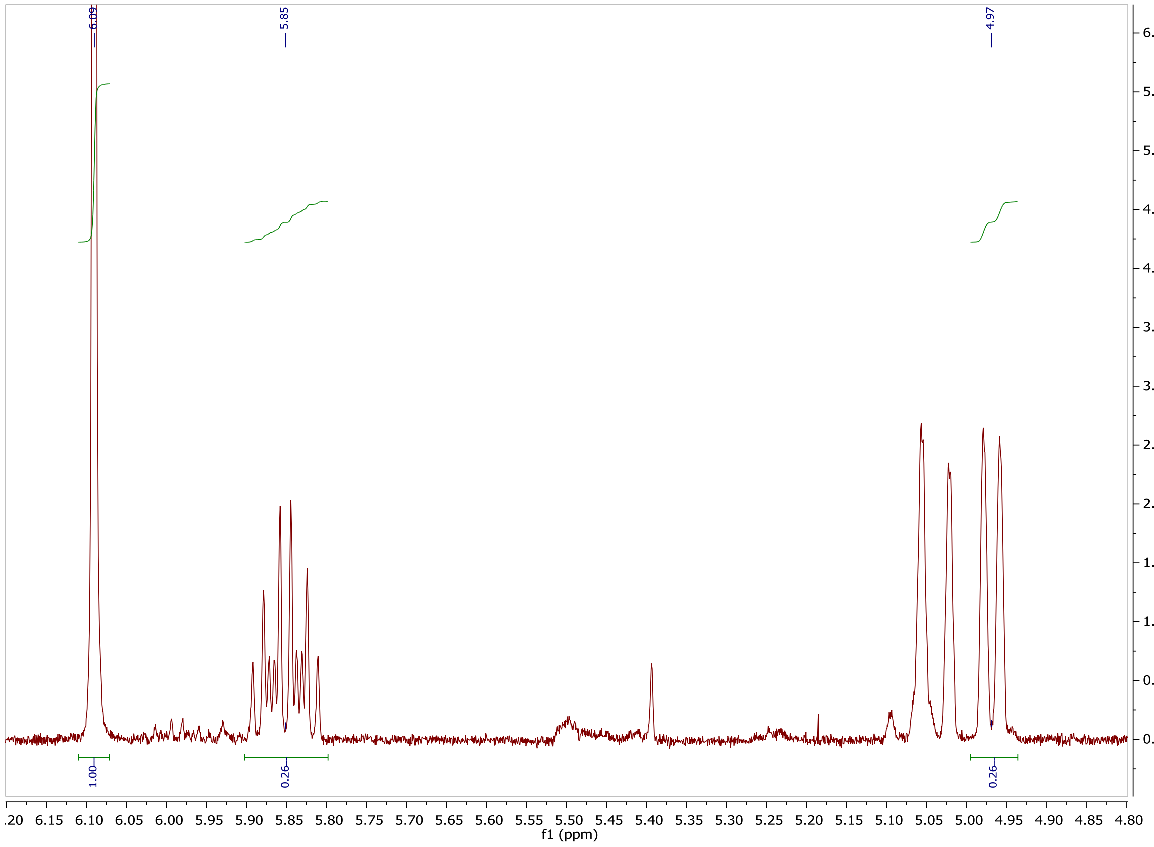

***tert*-Butyl((1-(4-methoxyphenyl)-3-(4,4,5,5-tetramethyl-1,3,2-dioxaborolan-2-yl)undecan-5-yl)oxy)dimethylsilane (40):**

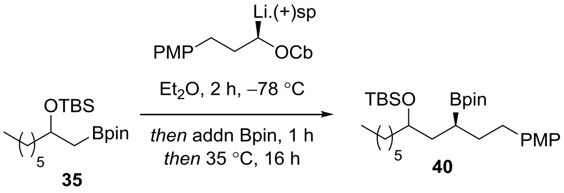

TLC: R_f = 0.39 (95:5 pentane:Et_2O)

^{1}H NMR: (400 MHz, $CDCl_3$) δ 7.09 (d, J = 7.9 Hz, 2H, ArH), 6.81 (d, J = 7.9 Hz, 2H, ArH), 6.09 (*impurity*, 1,3,5-trimethoxybenzene), 3.78 (s, 3H, OCH_3), 3.77 (*impurity*, 1,3,5-trimethoxybenzene), 3.73 – 3.55 (m, 1H, CHOSi), 2.64 – 2.45 (m, 2H, $ArCH_2$), 1.79 – 1.01 (m, 27H, 7×CH_2, CHBpin and $OC(CH_3)_2$), 0.92 – 0.84 (m, 12H, CH_3 and $SiC(CH_3)_3$), 0.07 – 0.02 (m, 6H, $Si(CH_3)_2$) ppm (*mixture of diastereoisomers*)

^{13}C NMR: (101 MHz, $CDCl_3$) δ 161.7 (*impurity*, 1,3,5-trimethoxybenzene), 157.8 (ArCOMe), 135.3 (ArC), 129.4 (ArH), 113.8 (ArH), 93.1 (*impurity*, 1,3,5-trimethoxybenzene), 83.1 (O*C*$(CH_3)_2$), 83.0 (O*C*$(CH_3)_2$), 72.6 (CHO), 71.5 (CHO), 55.5 (*impurity*, 1,3,5-trimethoxybenzene), 55.4 (CH_2), 39.1 (CH_2), 39.0 (CH_2), 38.0 (CH_2), 36.7 (CH_2), 34.8 (CH_2), 34.4 (CH_2), 32.1 (CH_2), 29.6 (CH_2), 26.2 (SiC(*C*$H_3)_3$), 26.1 (SiC(*C*$H_3)_3$), 25.3 (CH_2), 25.3 (CH_2), 25.1 (SiC(*C*$H_3)_3$), 25.1 (SiC(*C*$H_3)_3$), 25.0 (SiC(*C*$H_3)_3$), 25.0 (SiC(*C*$H_3)_3$), 22.8 (CH_2), 18.3 (Si*C*$(CH_3)_3$), 14.3 (CH_3), −4.1 ($Si(CH_3)_2$), −4.1 ($Si(CH_3)_2$), −4.1 ($Si(CH_3)_2$), −4.3 ($Si(CH_3)_2$) ppm (*mixture of diastereoisomers; observed signals*)

HRMS (*m/z*): (ESI) calc'd for $C_{30}H_{55}BO_4SiNa$ $[M+Na]^+$: 541.3861, found: 541.3862

IR (neat) ν_{max}: 2928, 2856, 1611, 1512, 1245, 1144, 1068, 1041, 834 and 773 cm^{-1}

2.2.3 Non-Selective homologations of 1,2-bis(boronic esters)

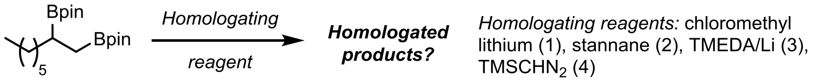

Entry	Carbenoid	Single add^n/ %	Double add^n/ %	SM/ %	Other
1	Cl Li	10	32	32	3% secondary single homologation
2	*(from Sn)* Li.Et_2O PMP OCb	17	37	43	
3	Li.TMEDA PMP OCb	5	19	47	normal addition
4	N_2 TMS	0	0	100	

Entry 1 – Matteson homologation:

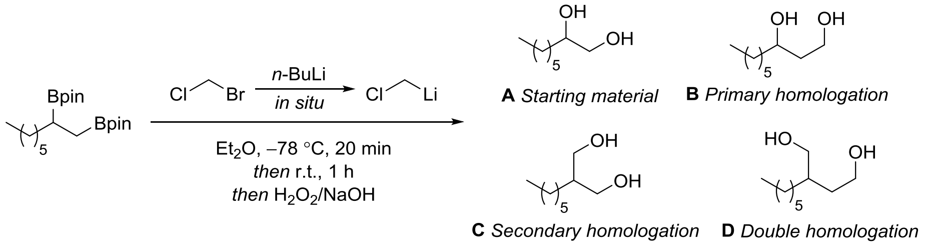

2,2'-(Octane-1,2-diyl)bis(4,4,5,5-tetramethyl-1,3,2-dioxaborolane) (100 mg, 0.27 mmol, 1.0 eq.), bromochloromethane (0.02 mL, 0.30 mmol, 1.10 eq.) and anhydrous Et_2O (1.40 mL) were added to a flame-dried Schlenk-tube purged with N_2. The solution was cooled to –78 °C (dry ice/acetone) and *n*-BuLi (1.6 M, 0.17 mL, 0.27 mmol, 1.0 eq.) was added dropwise (one drop every 20 seconds) before leaving the mixture for 20 min at this temperature. After warming to ambient temperature and leaving for 1 hr, THF (1.4 mL) was added and the resultant mixture was cooled to 0 °C (ice/water). A pre-mixed 2:1 v:v mixture of 3 M NaOH and 30% H_2O_2 (1.0 mL) was then added, before warming to ambient temperature. After 1 hr EtOAc (3 mL) and H_2O (2 mL) were added, and the mixture was extracted 3× with EtOAc (3 mL). The combined organic fractions were dried over Na_2SO_4, filtered, and concentrated under reduced pressure. The crude residue was passed through a plug of silica gel, eluting with EtOAc, and concentrated under reduced pressure to yield a mixture of **A**, **B**, **C**, **D** and pinacol as a colorless oil.

Analysis of the crude mixture, with 1,3,5-trimethoxybenzene as internal standard, by ^1H and ^{13}C NMR showed a ratio of 10:3:1:10 of **A**:**B**:**C**:**D**, which corresponds to yields of 32%:10%:3%:32%, respectively.

Entry 2 – Diamine-free homologation:

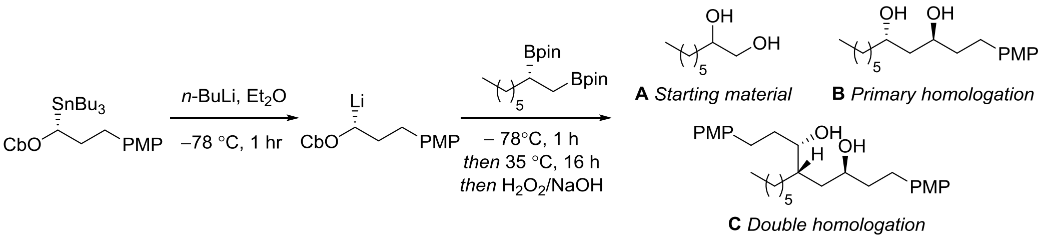

n-BuLi (1.6 M, 0.34 mL, 0.54 mmol, 1.0 eq.) was added dropwise to a solution of (*R*)-3-(4-methoxyphenyl)-1-(tributylstannyl)propyl diisopropylcarbamate (317 mg, 0.54 mmol, 1.0 eq.) in Et_2O (2.72 mL) at –78 °C (dry ice/acetone). After 1 h a solution of **2** (199 mg, 0.54 mmol, 1.0 eq.) in Et_2O (0.54 mL) was added rapidly and the resulting solution was allowed to stir at the same temperature for 1 h. The solution was warmed to ambient temperature and then heated at 35 °C (oil bath) for 16 h. After cooling to ambient temperature, the solution was cooled to 0 °C (ice/water) before adding a 2:1 v:v mixture of 3 M aqueous NaOH (4 mL) and 30% aqueous H_2O_2 (2 mL), which was prepared at 0 °C (ice/water) and degassed by gently bubbling N_2 through the solution. The mixture was subsequently warmed to ambient temperature and allowed to react for 1 hr. 2 M aqueous HCl (10 mL) was carefully added and the reaction mixture was extracted with EtOAc (3×20 mL). The combined organic fractions were dried over Na_2SO_4, filtered and concentrated under reduced pressure. The broad regions containing products **A**, and **B** and **C**, were collected by purification of the crude residue by flash column chromatography (SiO_2; 40:60 EtOAc:pentane) to yield **A** as a 1:2.27 mixture with pinacol (97 mg, 43% **A**) as a colorless viscous oil, and a 1:2.13 mixture of **B** and **C** (117 mg, 17% **B**, 37% **C**) as a colorless viscous oil.

Entry 3 – TMEDA-ligated carbenoid homologation:

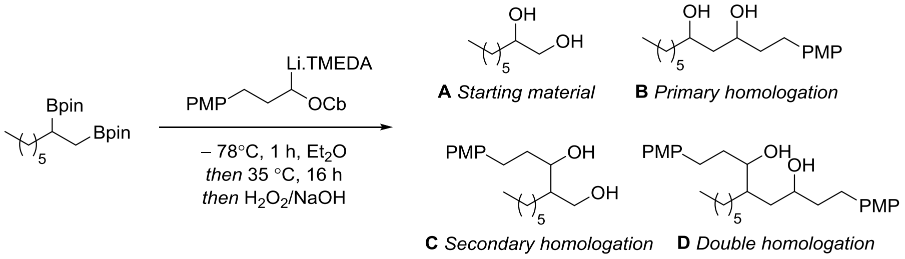

sec-BuLi (0.42 mL, 1.30 M, 0.55 mmol, 1.00 eq.) was added dropwise to a solution of **1a** (168 mg, 0.57 mmol, 1.05 eq.) and TMEDA (0.09 mL, 0.57 mmol, 1.05 eq.) in anhydrous Et_2O (2.87 mL) at −78 °C (dry ice/acetone). After 2 h and solution of *rac*-**2** (200 mg, 0.55 mmol, 1.00 eq.) in anhydrous Et_2O (0.55 mL) was added dropwise and the resulting solution was

allowed to react for a further 1 h. The solution was warmed to ambient temperature and then heated at 35 °C (oil bath) for 16 h. After cooling to ambient temperature, the solution was cooled to 0 °C (ice/water) before adding a 2:1 v:v mixture of 3 M aqueous NaOH (4 mL) and 30% aqueous H_2O_2 (2 mL), which was prepared at 0 °C (ice/water) and degassed by gently bubbling N_2 through the solution. The mixture was subsequently warmed to ambient temperature and allowed to react for 1 hr. 2 M aqueous HCl (10 mL) was carefully added and the reaction mixture was extracted with EtOAc (3×20 mL). The combined organic fractions were dried over Na_2SO_4, filtered and concentrated under reduced pressure. The broad regions containing products **A**, and **B**, **C** and **D**, were collected by purification of the crude residue by flash column chromatography (SiO_2; 40:60 EtOAc:CH_2Cl_2) to yield **A** (70 mg, 47%) as a colorless viscous oil, and a mixture of rearranged carbamate (65 mg), **B** and **D** (27 mg, 5% **B**; 46 mg, 19% **D**) as a colorless viscous oil. No presence of **C** was detected.

Entry 4 – TMS-diazomethane homologation:

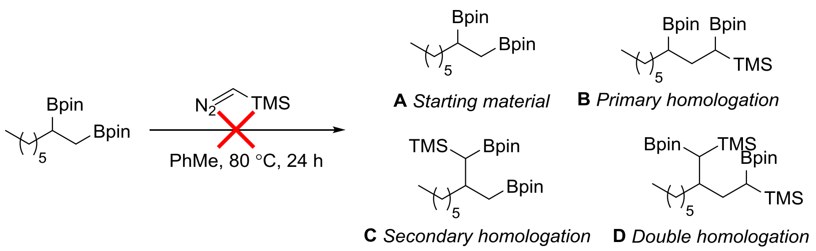

According to a modified literature procedure, **2** (100 mg, 0.27 mmol, 1.00 eq.) was dissolved in anhydrous toluene (1.00 mL) and trimethylsilyldiazomethane (0.55 mL, 2.0 m solution in hexanes, 1.09 mmol, 4.00 eq.) was added. The resulting solution was heated for 8 h at 80 °C (oil bath) before cooling to ambient temperature, adding another portion of trimethylsilyldiazomethane (0.55 mL, 2.0 m solution in hexanes, 1.09 mmol, 4.00 eq.) and heating at 80 °C (oil bath) for 16 h. After cooling to ambient temperature a few drops of acetic acid were added to quench any unreacted trimethylsilyldiazomethane. Analysis of the crude reaction mixture by GC-MS only showed the starting 1,2-bis(boronic ester) (**2**) and no other compounds, even in trace amounts.

2.3 Regioselective Homologation of 1,2-bis(boronic ester) development

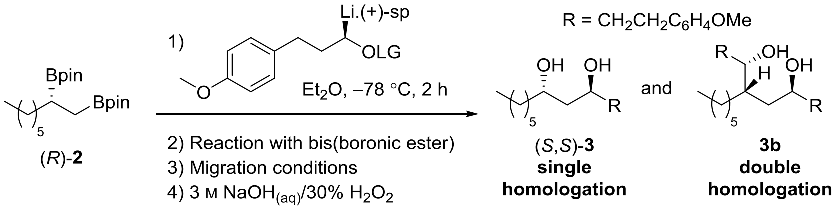

Entry	OLG	Eq. OLG	Eq. **2**	Add'n type	Migration	**3** Y/%	**3b** Y/%	**2** Y/%
1	OCb	1.00	1.00	Normal	35 °C, 16 h	60	3	traces
2	OTIB	1.00	1.00	Normal	warm to r.t.	53	9	traces
3	OCb	1.00	1.00	Inverse	35 °C, 16 h	57	3	traces
4	OTIB	1.00	1.00	Inverse	warm to r.t.	57	2	traces
5 (2)	OCb	1.20	1.00	Normal	35 °C, 16 h	61	6	0
6 (1)	OCb	1.00	1.20	Normal	35 °C, 16 h	66	traces	n.d.
7	OTIB	1.20	1.00	Normal	warm to r.t.	67	19	0
8 (4)	OTIB	1.00	1.20	Normal	warm to r.t.	69	traces	n.d.
9	OTIB	1.00	1.20	Normal	$MgBr_2$.MeOH	48	0	n.d.
10	OCb	1.00	1.20	Normal	Solv. exchange to $CHCl_3$	62	traces	n.d.
11	OCb	1.00	1.20	Normal	$MgBr_2.Et_2O$	0	0	n.d.
12 (5)	OTIB	1.20	1.00	Normal	MeOH quench	66	traces	n.d.
13 (3)	OCb	1.20	1.00	Normal	MeOH quench	63	2	n.d.

Table 1. Reaction optimization table. Results highlighted in yellow are shown in the manuscript, with the table entry from the manuscript shown in brackets.

Entry 1: 3-(4-Methoxyphenyl)propyl diisopropylcarbamate (168 mg, 0.57 mmol, 1.05 eq.), (+)-sparteine (0.13 mL, 0.57 mmol, 1.05 eq.) and anhydrous Et_2O (2.85 mL) were added to a flame-dried Schlenk-tube purged with N_2. The solution was cooled to –78 °C (dry ice/acetone) before adding *sec*-BuLi (0.43 mL, 1.28 M, 0.55 mmol, 1.00 eq.) dropwise over 5 min and leaving to react for 2 hr at this temperature. (*R*)-**2** (200 mg, 0.55 mmol, 1.00 eq.) was dissolved in anhydrous Et_2O (0.55 mL) and added dropwise to the reaction mixture over 1 min before leaving to react for a further 1 hr at the same temperature. After warming to ambient temperature the Schlenk-tube was sealed and heated at 35 °C (oil bath) for 16 h. The flask was allowed to cool to ambient temperature before adding THF (2.85 mL) and one crystal of BHT, and was then cooled to 0 °C (ice/water). A 2:1 v:v mixture of 3 M aqueous NaOH (4 mL) and 30% aqueous H_2O_2 (2 mL) was prepared at 0 °C (ice/water) and degassed by gently bubbling N_2 through the solution. This aqueous solution was added dropwise to the vigorously stirred reaction mixture, which was subsequently warmed to ambient temperature and allowed to react

for 1 hr. 2 M aqueous HCl (10 mL) was carefully added and the reaction mixture was extracted with EtOAc (3×20 mL). The combined organic fractions were dried over Na_2SO_4, filtered and concentrated under reduced pressure. The crude residue was purified by flash column chromatography (SiO_2; 75:25 petroleum ether 40/60:EtOAc) to yield (*S*,*S*)-**3** (97 mg, 60%) as a gummy white solid and **3b** (6 mg, 3%) as a colorless oil.

Entry 2: 3-(4-Methoxyphenyl)propyl 2,4,6-triisopropylbenzoate (227 mg, 0.57 mmol, 1.05 eq.), (+)-sparteine (0.13 mL, 0.57 mmol, 1.05 eq.) and anhydrous Et_2O (2.85 mL) were added to a flame-dried Schlenk-tube purged with N_2. The solution was cooled to –78 °C (dry ice/acetone) before adding *sec*-BuLi (0.43 mL, 1.28 M, 0.55 mmol, 1.00 eq.) dropwise over 5 min and leaving to react for 2 hr at this temperature. (*R*)-**2** (200 mg, 0.55 mmol, 1.00 eq.) was dissolved in anhydrous Et_2O (0.55 mL) and added dropwise to the reaction mixture over 1 min before leaving to react for a further 1 hr at the same temperature. The flask was allowed to warm to ambient temperature before adding THF (2.85 mL) and one crystal of BHT, and was then cooled to 0 °C (ice/water). A 2:1 v:v mixture of 3 M aqueous NaOH (4 mL) and 30% aqueous H_2O_2 (2 mL) was prepared at 0 °C (ice/water) and degassed by gently bubbling N_2 through the solution. This aqueous solution was added dropwise to the vigorously stirred reaction mixture, which was subsequently warmed to ambient temperature and allowed to react for 1 hr. 2 M aqueous HCl (10 mL) was carefully added and the reaction mixture was extracted with EtOAc (3×20 mL). The combined organic fractions were dried over Na_2SO_4, filtered and concentrated under reduced pressure. The crude residue was purified by flash column chromatography (SiO_2; 75:25 petroleum ether 40/60:EtOAc) to yield (*S*,*S*)-**3** (86 mg, 53%) as a gummy white solid and **3b** (21 mg, 9%) as a colorless oil.

Entry 3: 3-(4-Methoxyphenyl)propyl diisopropylcarbamate (337 mg, 1.15 mmol, 1.05 eq.), (+)-sparteine (0.26 mL, 1.15 mmol, 1.05 eq.) and anhydrous Et_2O (5.75 mL) were added to the left-hand side of a flame-dried Fawcett Flask (*see* **Figure 1**) purged with N_2. The solution was cooled to –78 °C (dry ice/acetone) before adding *sec*-BuLi (0.85 mL, 1.28 M, 0.55 mmol, 1.00 eq.) dropwise over 5 min and leaving to react for 2 hr at this temperature. (*R*)-**2** (400 mg, 1.09 mmol, 1.00 eq.) was dissolved in anhydrous Et_2O (1.10 mL) in the right-hand flask and allowed to cool to -78 °C over 5 min. The solution of lithiated carbamate was added dropwise to the boronic ester solution over 5 min before leaving to react for a further 1 hr at the same temperature. After warming to ambient temperature the flask was sealed and heated at 35 °C (oil bath) for 16 hr. The flask was allowed to cool to ambient temperature before adding THF (5.75 mL) and one crystal of BHT, and was then cooled to 0 °C (ice/water). A 2:1 v:v mixture

of 3 M aqueous NaOH (8 mL) and 30% aqueous H_2O_2 (4 mL) was prepared at 0 °C (ice/water) and degassed by gently bubbling N_2 through the solution. This aqueous solution was added dropwise to the vigorously stirred reaction mixture, which was subsequently warmed to ambient temperature and allowed to react for 1 hr. 2 M aqueous HCl (20 mL) was carefully added and the reaction mixture was extracted with EtOAc (3×40 mL). The combined organic fractions were dried over Na_2SO_4, filtered and concentrated under reduced pressure. The crude residue was purified by flash column chromatography (SiO_2; 75:25 petroleum ether 40/60:EtOAc) to yield (*S*,*S*)-**3** (183 mg, 57%) as a gummy white solid and **4** (13 mg, 3%) as a colorless oil.

Entry 4: 3-(4-Methoxyphenyl)propyl 2,4,6-triisopropylbenzoate (455 mg, 1.15 mmol, 1.05 eq.), (+)-sparteine (0.26 mL, 1.15 mmol, 1.05 eq.) and anhydrous Et_2O (5.75 mL) were added to the left-hand side of a flame-dried Fawcett Flask (*see* **Figure 1**) purged with N_2. The solution was cooled to –78 °C (dry ice/acetone) before adding *sec*-BuLi (0.85 mL, 1.28 M, 0.55 mmol, 1.00 eq.) dropwise over 5 min and leaving to react for 2 hr at this temperature. (*R*)-**2** (400 mg, 1.09 mmol, 1.00 eq.) was dissolved in anhydrous Et_2O (1.10 mL) in the right-hand flask and allowed to cool to -78 °C over 5 min. The solution of lithiated benzoate was added dropwise to the boronic ester solution over 5 min before leaving to react for a further 1 hr at the same temperature. The flask was allowed to warm to ambient temperature before adding THF (5.75 mL) and one crystal of BHT, and was then cooled to 0 °C (ice/water). A 2:1 v:v mixture of 3 M aqueous NaOH (8 mL) and 30% aqueous H_2O_2 (4 mL) was prepared at 0 °C (ice/water) and degassed by gently bubbling N_2 through the solution. This aqueous solution was added dropwise to the vigorously stirred reaction mixture, which was subsequently warmed to ambient temperature and allowed to react for 1 hr. 2 M aqueous HCl (20 mL) was carefully added and the reaction mixture was extracted with EtOAc (3×40 mL). The combined organic fractions were dried over Na_2SO_4, filtered and concentrated under reduced pressure. The crude residue was purified by flash column chromatography (SiO_2; 75:25 petroleum ether 40/60:EtOAc) to yield (*S*,*S*)-**3** (183 mg, 57%) as a gummy white solid and **3b** (11 mg, 2%) as a colorless oil.

Entry 5: 3-(4-Methoxyphenyl)propyl diisopropylcarbamate (200 mg, 0.68 mmol, 1.25 eq.), (+)-sparteine (0.16 mL, 0.68 mmol, 1.25 eq.) and anhydrous Et_2O (3.40 mL) were added to a flame-dried Schlenk-tube purged with N_2. The solution was cooled to –78 °C (dry ice/acetone) before adding *sec*-BuLi (0.51 mL, 1.30 M, 0.66 mmol, 1.20 eq.) dropwise over 5 min and leaving to react for 2 hr at this temperature. (*R*)-**2** (200 mg, 0.55 mmol, 1.00 eq.) was dissolved

in anhydrous Et_2O (0.55 mL) and added dropwise to the reaction mixture over 1 min before leaving to react for a further 1 hr at the same temperature. After warming to ambient temperature the Schlenk-tube was sealed and heated at 35 °C (oil bath) for 16 hr. The flask was allowed to cool to ambient temperature before adding THF (3.40 mL) and one crystal of BHT, and was then cooled to 0 °C (ice/water). A 2:1 v:v mixture of 3 M aqueous NaOH (4 mL) and 30% aqueous H_2O_2 (2 mL) was prepared at 0 °C (ice/water) and degassed by gently bubbling N_2 through the solution. This aqueous solution was added dropwise to the vigorously stirred reaction mixture, which was subsequently warmed to ambient temperature and allowed to react for 1 hr. 2 M aqueous HCl (10 mL) was carefully added and the reaction mixture was extracted with EtOAc (3×20 mL). The combined organic fractions were dried over Na_2SO_4, filtered and concentrated under reduced pressure. The crude residue was purified by flash column chromatography (SiO_2; 75:25 petroleum ether 40/60:EtOAc) to yield (*S*,*S*)-**3** (99 mg, 61%) as a gummy white solid and **3b** (14 mg, 6%) as a colorless oil.

Entry 6: 3-(4-Methoxyphenyl)propyl diisopropylcarbamate (167 mg, 0.57 mmol, 1.05 eq.), (+)-sparteine (0.13 mL, 0.57 mmol, 1.05 eq.) and anhydrous Et_2O (2.85 mL) were added to a flame-dried Schlenk-tube purged with N_2. The solution was cooled to −78 °C (dry ice/acetone) before adding *sec*-BuLi (0.42 mL, 1.30 M, 0.55 mmol, 1.00 eq.) dropwise over 5 min and leaving to react for 2 hr at this temperature. (*R*)-**2** (242 mg, 0.66 mmol, 1.20 eq.) was dissolved in anhydrous Et_2O (0.66 mL) and added dropwise to the reaction mixture over 1 min before leaving to react for a further 1 hr at the same temperature. After warming to ambient temperature the Schlenk-tube was sealed and heated at 35 °C (oil bath) for 16 hr. The flask was allowed to cool to ambient temperature before adding THF (2.85 mL) and one crystal of BHT, and was then cooled to 0 °C (ice/water). A 2:1 v:v mixture of 3 M aqueous NaOH (4 mL) and 30% aqueous H_2O_2 (2 mL) was prepared at 0 °C (ice/water) and degassed by gently bubbling N_2 through the solution. This aqueous solution was added dropwise to the vigorously stirred reaction mixture, which was subsequently warmed to ambient temperature and allowed to react for 1 hr. 2 M aqueous HCl (10 mL) was carefully added and the reaction mixture was extracted with EtOAc (3×20 mL). The combined organic fractions were dried over Na_2SO_4, filtered and concentrated under reduced pressure. The crude residue was purified by flash column chromatography (SiO_2; 75:25 petroleum ether 40/60:EtOAc) to yield (*S*,*S*)-**3** (106 mg, 66%) as a gummy white solid.

Entry 7: 3-(4-Methoxyphenyl)propyl 2,4,6-triisopropylbenzoate (271 mg, 0.68 mmol, 1.25 eq.), (+)-sparteine (0.16 mL, 0.68 mmol, 1.25 eq.) and anhydrous Et_2O (3.40 mL) were added

to a flame-dried Schlenk-tube purged with N_2. The solution was cooled to −78 °C (dry ice/acetone) before adding *sec*-BuLi (0.51 mL, 1.30 M, 0.66 mmol, 1.20 eq.) dropwise over 5 min and leaving to react for 2 hr at this temperature. (*R*)-**2** (200 mg, 0.55 mmol, 1.00 eq.) was dissolved in anhydrous Et_2O (0.55 mL) and added dropwise to the reaction mixture over 1 min before leaving to react for a further 1 hr at the same temperature. The flask was allowed to warm to ambient temperature before adding THF (3.40 mL) and one crystal of BHT, and was then cooled to 0 °C (ice/water). A 2:1 v:v mixture of 3 M aqueous NaOH (4 mL) and 30% aqueous H_2O_2 (2 mL) was prepared at 0 °C (ice/water) and degassed by gently bubbling N_2 through the solution. This aqueous solution was added dropwise to the vigorously stirred reaction mixture, which was subsequently warmed to ambient temperature and allowed to react for 1 hr. 2 M aqueous HCl (10 mL) was carefully added and the reaction mixture was extracted with EtOAc (3×20 mL). The combined organic fractions were dried over Na_2SO_4, filtered and concentrated under reduced pressure. The crude residue was purified by flash column chromatography (SiO_2; 75:25 petroleum ether 40/60:EtOAc) to yield (*S*,*S*)-**3** (109 mg, 67%) as a gummy white solid and **3b** (46 mg, 19%) as a colorless oil.

Entry 8: 3-(4-Methoxyphenyl)propyl 2,4,6-triisopropylbenzoate (226 mg, 0.57 mmol, 1.05 eq.), (+)-sparteine (0.13 mL, 0.57 mmol, 1.05 eq.) and anhydrous Et_2O (2.85 mL) were added to a flame-dried Schlenk-tube purged with N_2. The solution was cooled to −78 °C (dry ice/acetone) before adding *sec*-BuLi (0.42 mL, 1.30 M, 0.55 mmol, 1.00 eq.) dropwise over 5 min and leaving to react for 2 hr at this temperature. (*R*)-**2** (242 mg, 0.66 mmol, 1.20 eq.) was dissolved in anhydrous Et_2O (0.66 mL) and added dropwise to the reaction mixture over 1 min before leaving to react for a further 1 hr at the same temperature. The flask was allowed to warm to ambient temperature before adding THF (2.85 mL) and one crystal of BHT, and was then cooled to 0 °C (ice/water). A 2:1 v:v mixture of 3 M aqueous NaOH (4 mL) and 30% aqueous H_2O_2 (2 mL) was prepared at 0 °C (ice/water) and degassed by gently bubbling N_2 through the solution. This aqueous solution was added dropwise to the vigorously stirred reaction mixture, which was subsequently warmed to ambient temperature and allowed to react for 1 hr. 2 M aqueous HCl (10 mL) was carefully added and the reaction mixture was extracted with EtOAc (3×20 mL). The combined organic fractions were dried over Na_2SO_4, filtered and concentrated under reduced pressure. The crude residue was purified by flash column chromatography (SiO_2; 75:25 petroleum ether 40/60:EtOAc) to yield (*S*,*S*)-**3** (111 mg, 69%) as a gummy white solid.

Entry 9: 3-(4-Methoxyphenyl)propyl 2,4,6-triisopropylbenzoate (226 mg, 0.57 mmol, 1.05 eq.), (+)-sparteine (0.13 mL, 0.57 mmol, 1.05 eq.) and anhydrous Et_2O (2.85 mL) were added to a flame-dried Schlenk-tube purged with N_2. The solution was cooled to −78 °C (dry ice/acetone) before adding *sec*-BuLi (0.42 mL, 1.30 M, 0.55 mmol, 1.00 eq.) dropwise over 5 min and leaving to react for 2 hr at this temperature. (*R*)-**2** (242 mg, 0.66 mmol, 1.20 eq.) was dissolved in anhydrous Et_2O (0.66 mL) and added dropwise to the reaction mixture over 1 min before leaving to react for a further 1 hr at the same temperature. A 1 M solution of $MgBr_2$ in MeOH (0.83 mL, 0.83 mmol, 1.50 eq.) was added dropwise over 2 min and the mixture was allowed to react for a further 2 min before warming to ambient temperature. THF (2.85 mL) and one crystal of BHT were added, and the mixture was subsequently cooled to 0 °C (ice/water). A 2:1 v:v mixture of 3 M aqueous NaOH (4 mL) and 30% aqueous H_2O_2 (2 mL) was prepared at 0 °C (ice/water) and degassed by gently bubbling N_2 through the solution. This aqueous solution was added dropwise to the vigorously stirred reaction mixture, which was subsequently warmed to ambient temperature and allowed to react for 1 hr. 2 M aqueous HCl (10 mL) was carefully added and the reaction mixture was extracted with EtOAc (3×20 mL). The combined organic fractions were dried over Na_2SO_4, filtered and concentrated under reduced pressure. The crude residue was purified by flash column chromatography (SiO_2; 75:25 petroleum ether 40/60:EtOAc) to yield (*S*,*S*)-**3** (77 mg, 48%) as a gummy white solid.

Entry 10: 3-(4-Methoxyphenyl)propyl diisopropylcarbamate (167 mg, 0.57 mmol, 1.05 eq.), (+)-sparteine (0.13 mL, 0.57 mmol, 1.05 eq.) and anhydrous Et_2O (2.85 mL) were added to a flame-dried Schlenk-tube purged with N_2. The solution was cooled to −78 °C (dry ice/acetone) before adding *sec*-BuLi (0.42 mL, 1.30 M, 0.55 mmol, 1.00 eq.) dropwise over 5 min and leaving to react for 2 hr at this temperature. (*R*)-**2** (242 mg, 0.66 mmol, 1.20 eq.) was dissolved in anhydrous Et_2O (0.66 mL) and added dropwise to the reaction mixture over 1 min before leaving to react for a further 1 hr at the same temperature. After warming to ambient temperature the Et_2O was carefully removed under reduced pressure and replaced with anhydrous $CHCl_3$ (4.0 mL) before sealing the Schlenk-tube and heating at 65 °C (oil bath) for 2 hr. The flask was allowed to cool to ambient temperature before adding THF (2.85 mL) and one crystal of BHT, and was then cooled to 0 °C (ice/water). A 2:1 v:v mixture of 3 M aqueous NaOH (4 mL) and 30% aqueous H_2O_2 (2 mL) was prepared at 0 °C (ice/water) and degassed by gently bubbling N_2 through the solution. This aqueous solution was added dropwise to the vigorously stirred reaction mixture, which was subsequently warmed to ambient temperature and allowed to react for 1 hr. 2 M aqueous HCl (10 mL) was carefully added and the reaction

mixture was extracted with EtOAc (3×20 mL). The combined organic fractions were dried over Na_2SO_4, filtered and concentrated under reduced pressure. The crude residue was purified by flash column chromatography (SiO_2; 75:25 petroleum ether 40/60:EtOAc) to yield (*S*,*S*)-**3** (101 mg, 62%) as a gummy white solid.

Entry 11: 3-(4-Methoxyphenyl)propyl diisopropylcarbamate (167 mg, 0.57 mmol, 1.05 eq.), (+)-sparteine (0.13 mL, 0.57 mmol, 1.05 eq.) and anhydrous Et_2O (2.85 mL) were added to a flame-dried Schlenk-tube purged with N_2. The solution was cooled to -78 °C (dry ice/acetone) before adding *sec*-BuLi (0.42 mL, 1.30 M, 0.55 mmol, 1.00 eq.) dropwise over 5 min and leaving to react for 2 hr at this temperature. (*R*)-**2** (242 mg, 0.66 mmol, 1.20 eq.) was dissolved in anhydrous Et_2O (0.66 mL) and added dropwise to the reaction mixture over 1 min before leaving to react for a further 1 hr at the same temperature. A freshly prepared 1 M solution of $MgBr_2$ in Et_2O (0.83 mL, 0.83 mmol, 1.50 eq.) was added dropwise over 2 min and the mixture was allowed to react for a further 2 min before warming to ambient temperature. At this stage ^{11}B NMR showed no ate-complex and TLC showed no formation of single or doubly homologated products.

Entry 12: 3-(4-Methoxyphenyl)propyl 2,4,6-triisopropylbenzoate (271 mg, 0.68 mmol, 1.25 eq.), (+)-sparteine (0.16 mL, 0.68 mmol, 1.25 eq.) and anhydrous Et_2O (3.40 mL) were added to a flame-dried Schlenk-tube purged with N_2. The solution was cooled to −78 °C (dry ice/acetone) before adding *sec*-BuLi (0.51 mL, 1.30 M, 0.66 mmol, 1.20 eq.) dropwise over 5 min and leaving to react for 2 hr at this temperature. (*R*)-**2** (200 mg, 0.55 mmol, 1.00 eq.) was dissolved in anhydrous Et_2O (0.55 mL) and added dropwise to the reaction mixture over 1 min before leaving to react for a further 1 hr at the same temperature. MeOH (0.1 mL) was added dropwise before allowing the flask to warm to ambient temperature. THF (3.40 mL) and one crystal of BHT were added, before cooling to 0 °C (ice/water). A 2:1 v:v mixture of 3 M aqueous NaOH (4 mL) and 30% aqueous H_2O_2 (2 mL) was prepared at 0 °C (ice/water) and degassed by gently bubbling N_2 through the solution. This aqueous solution was added dropwise to the vigorously stirred reaction mixture, which was subsequently warmed to ambient temperature and allowed to react for 1 hr. 2 M aqueous HCl (10 mL) was carefully added and the reaction mixture was extracted with EtOAc (3×20 mL). The combined organic fractions were dried over Na_2SO_4, filtered and concentrated under reduced pressure. The crude residue was purified by flash column chromatography (SiO_2; 75:25 petroleum ether 40/60:EtOAc) to yield (*S*,*S*)-**3** (107 mg, 66%) as a gummy white solid.

Entry 13: 3-(4-Methoxyphenyl)propyl diisopropylcarbamate (200 mg, 0.68 mmol, 1.25 eq.), (+)-sparteine (0.16 mL, 0.68 mmol, 1.25 eq.) and anhydrous Et_2O (3.40 mL) were added to a flame-dried Schlenk-tube purged with N_2. The solution was cooled to −78 °C (dry ice/acetone) before adding *sec*-BuLi (0.51 mL, 1.30 M, 0.66 mmol, 1.20 eq.) dropwise over 5 min and leaving to react for 2 hr at this temperature. (*R*)-**2** (200 mg, 0.55 mmol, 1.00 eq.) was dissolved in anhydrous Et_2O (0.55 mL) and added dropwise to the reaction mixture over 1 min before leaving to react for a further 1 hr at the same temperature. Anhydrous methanol (0.1 mL) was added dropwise and the reaction was left for a further 2 min. After warming to ambient temperature the Schlenk-tube was sealed and heated at 35 °C (oil bath) for 16 hr. The flask was allowed to cool to ambient temperature before adding THF (3.40 mL) and one crystal of BHT, and was then cooled to 0 °C (ice/water). A 2:1 v:v mixture of 3 M aqueous NaOH (4 mL) and 30% aqueous H_2O_2 (2 mL) was prepared at 0 °C (ice/water) and degassed by gently bubbling N_2 through the solution. This aqueous solution was added dropwise to the vigorously stirred reaction mixture, which was subsequently warmed to ambient temperature and allowed to react for 1 hr. 2 M aqueous HCl (10 mL) was carefully added and the reaction mixture was extracted with EtOAc (3×20 mL). The combined organic fractions were dried over Na_2SO_4, filtered and concentrated under reduced pressure. The crude residue was purified by flash column chromatography (SiO_2; 75:25 petroleum ether 40/60:EtOAc) to yield (*S*,*S*)-**3** (102 mg, 63%) as a gummy white solid and **3b** (5 mg, 2%) as a colorless oil.

2.4 Final Procedures

(3*S*,5*S*)-1-(4-Methoxyphenyl)undecane-3,5-diol (*S*,*S*-3):

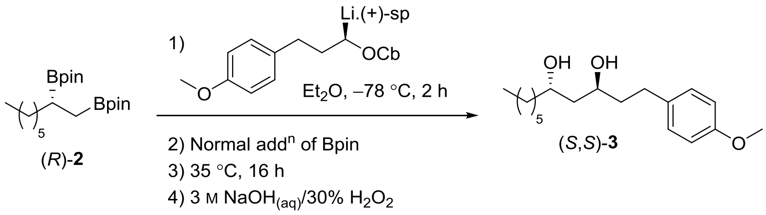

3-(4-Methoxyphenyl)propyl diisopropylcarbamate (167 mg, 0.57 mmol, 1.05 eq.), (+)-sparteine (0.13 mL, 0.57 mmol, 1.05 eq.) and anhydrous Et_2O (2.85 mL) were added to a flame-dried Schlenk-tube purged with N_2. The solution was cooled to −78 °C (dry ice/acetone) before adding *sec*-BuLi (0.42 mL, 1.30 M, 0.55 mmol, 1.00 eq.) dropwise over 5 min and leaving to react for 2 hr at this temperature. (*R*)-**2** (242 mg, 0.66 mmol, 1.20 eq.) was dissolved in anhydrous Et_2O (0.66 mL) and added dropwise to the reaction mixture over 1 min before

leaving to react for a further 1 hr at the same temperature. After warming to ambient temperature the Schlenk-tube was sealed and heated at 35 °C (oil bath) for 16 hr. The flask was allowed to cool to ambient temperature before adding THF (2.85 mL) and one crystal of BHT, and was then cooled to 0 °C (ice/water). A 2:1 v:v mixture of 3 M aqueous NaOH (4 mL) and 30% aqueous H_2O_2 (2 mL) was prepared at 0 °C (ice/water) and degassed by gently bubbling N_2 through the solution. This aqueous solution was added dropwise to the vigorously stirred reaction mixture, which was subsequently warmed to ambient temperature and allowed to react for 1 hr. 2 M aqueous HCl (10 mL) was carefully added and the reaction mixture was extracted with EtOAc (3×20 mL). The combined organic fractions were dried over Na_2SO_4, filtered and concentrated under reduced pressure. The crude residue was purified by flash column chromatography (SiO_2; 75:25 petroleum ether 40/60:EtOAc) to yield (*S*,*S*)-**3** (106 mg, 66%) as a gummy white solid.

TLC: R_f = 0.18 (60:40 petroleum ether 40/60:EtOAc)

^{1}H NMR: (500 MHz, $CDCl_3$) δ 7.90 – 7.14 (m, 2H, ArH), 6.85 – 6.81 (m, 2H, ArH), 3.99 – 3.91 (m, 2H, CHOH), 3.78 (s, 3H, OCH_3), 2.73 (ddd, *J* = 13.8, 9.8, 5.7 Hz, 1H, ArCH^aH^b), 2.62 (ddd, *J* = 13.8, 9.6, 6.43, 1H, ArCH^aH^b), 2.40 (br s, 2H, OH), 1.87 – 1.80 (m, 1H, $ArCH_2CH^aH^b$), 1.78 – 1.69 (m, 1H, $ArCH_2CH^aH^b$), 1.63 (ddd, *J* = 6.8, 3.9, 2.1 Hz, 2H, $CHOHCH_2CHOH$), 1.57 – 1.20 (m, 10H, 5× CH_2), 0.88 (t, *J* = 6.8 Hz, 3H, CH_3) ppm

^{13}C NMR: (126 MHz, $CDCl_3$) δ 157.9 (Ar*C*OCH_3), 134.2 (ArC), 129.40 (ArH), 114.0 (ArH), 69.6 (C*H*OH), 69.0 (C*H*OH), 55.4 (OCH_3), 42.6 ($CHOHCH_2CHOH$), 39.5 ($ArCH_2CH_2$), 37.7 (CH_2), 31.9 (CH_2), 31.4 ($ArCH_2$), 29.4 (CH_2), 25.9 (CH_2), 22.7 (CH_2), 14.2 (CH_3) ppm

HRMS (*m*/*z*): (ESI) calc'd for $C_{18}H_{30}O_3Na$ $[M+Na]^+$: 317.2087, found: 317.2095

IR (solid state) ν_{max}: 3284, 2924, 1611, 1512, 1248, 1033 and 705 cm^{-1}

$[\alpha]_D^{24}$: −7.0 (*c* = 1.0, $CHCl_3$)

(3*R*,5*S*)-1-(4-Methoxyphenyl)undecane-3,5-diol (*R*,*S*-3):

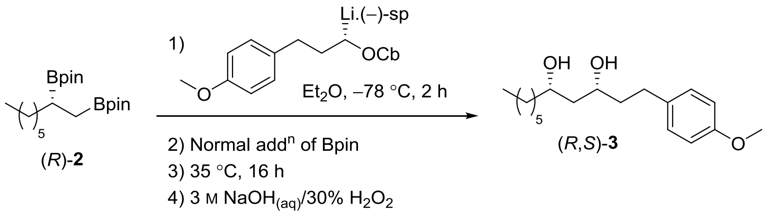

3-(4-Methoxyphenyl)propyl diisopropylcarbamate (167 mg, 0.57 mmol, 1.05 eq.), (–)-sparteine (0.13 mL, 0.57 mmol, 1.05 eq.) and anhydrous Et_2O (2.85 mL) were added to a flame-dried Schlenk-tube purged with N_2. The solution was cooled to −78 °C (dry ice/acetone) before adding *sec*-BuLi (0.42 mL, 1.30 M, 0.55 mmol, 1.00 eq.) dropwise over 5 min and leaving to react for 2 hr at this temperature. (*R*)-**2** (242 mg, 0.66 mmol, 1.20 eq.) was dissolved in anhydrous Et_2O (0.66 mL) and added dropwise to the reaction mixture over 1 min before leaving to react for a further 1 hr at the same temperature. After warming to ambient temperature the Schlenk-tube was sealed and heated at 35 °C (oil bath) for 16 hr. The flask was allowed to cool to ambient temperature before adding THF (2.85 mL) and one crystal of BHT, and was then cooled to 0 °C (ice/water). A 2:1 v:v mixture of 3 M aqueous NaOH (4 mL) and 30% aqueous H_2O_2 (2 mL) was prepared at 0 °C (ice/water) and degassed by gently bubbling N_2 through the solution. This aqueous solution was added dropwise to the vigorously stirred reaction mixture, which was subsequently warmed to ambient temperature and allowed to react for 1 hr. 2 M aqueous HCl (10 mL) was carefully added and the reaction mixture was extracted with EtOAc (3×20 mL). The combined organic fractions were dried over Na_2SO_4, filtered and concentrated under reduced pressure. The crude residue was purified by flash column chromatography (SiO_2; 75:25 petroleum ether 40/60:EtOAc) to yield (*R*,*S*)-**3** (118 mg, 73%) as a colorless oil.

TLC: R_f = 0.23 (60:40 petroleum ether 40/40:EtOAc)

^{1}H NMR: (500 MHz, $CDCl_3$) δ 7.14 – 7.09 (m, 2H, ArH), 6.85 – 6.80 (m, 2H, ArH), 3.90 – 3.81(m, 2H, C*H*OH), 3.79 (s, 3H, OCH_3), 2.85 (br s, 2H, OH), 2.75 – 2.59 (m, 2H, $ArCH_2$), 1.85 – 1.67 (m, 2H, $ArCH_2CH_2$), 1.64 – 1.23 (m, 12H, 6× CH_2), 0.88 (t, J = 7.1 Hz, 3H, CH_3) ppm

^{13}C NMR: (126 MHz, $CDCl_3$) δ 157.9 (Ar*C*OCH_3), 134.1 (ArC), 129.4 (ArH), 114.0 (ArH), 73.4 (CHOH), 72.5 (CHOH), 55.4 (OCH_3), 43.1 (CH_2), 40.1 ($ArCH_2CH_2$), 38.5 (CH_2), 31.9 (CH_2), 30.9 ($ArCH_2$), 29.4 (CH_2), 25.4 (CH_2), 22.7 (CH_2), 14.2 (CH_3) ppm

HRMS (*m/z*): (ESI) calc'd for $C_{18}H_{30}O_3Na$ $[M+Na]^+$: 317.2087, found: 317.2092

IR (neat) ν_{max}: 3358, 2928, 2856, 1512, 1244, 1037 and 820 cm^{-1}

$[\alpha]_D^{24}$**:** +11.0 (*c* = 1.0, $CHCl_3$)

(3*R*,5*R*)-1-(4-Methoxyphenyl)undecane-3,5-diol (*R*,*R*-3):

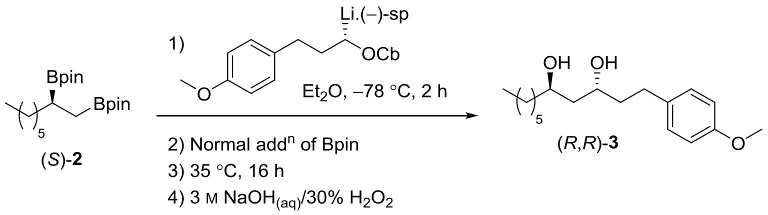

3-(4-Methoxyphenyl)propyl diisopropylcarbamate (167 mg, 0.57 mmol, 1.05 eq.), (−)-sparteine (0.13 mL, 0.57 mmol, 1.05 eq.) and anhydrous Et_2O (2.85 mL) were added to a flame-dried Schlenk-tube purged with N_2. The solution was cooled to −78 °C (dry ice/acetone) before adding *sec*-BuLi (0.42 mL, 1.30 M, 0.55 mmol, 1.00 eq.) dropwise over 5 min and leaving to react for 2 hr at this temperature. (*S*)-**2** (242 mg, 0.66 mmol, 1.20 eq.) was dissolved in anhydrous Et_2O (0.66 mL) and added dropwise to the reaction mixture over 1 min before leaving to react for a further 1 hr at the same temperature. After warming to ambient temperature the Schlenk-tube was sealed and heated at 35 °C (oil bath) for 16 hr. The flask was allowed to cool to ambient temperature before adding THF (2.85 mL) and one crystal of BHT, and was then cooled to 0 °C (ice/water). A 2:1 v:v mixture of 3 M aqueous NaOH (4 mL) and 30% aqueous H_2O_2 (2 mL) was prepared at 0 °C (ice/water) and degassed by gently bubbling N_2 through the solution. This aqueous solution was added dropwise to the vigorously stirred reaction mixture, which was subsequently warmed to ambient temperature and allowed to react for 1 hr. 2 M aqueous HCl (10 mL) was carefully added and the reaction mixture was extracted with EtOAc (3×20 mL). The combined organic fractions were dried over Na_2SO_4, filtered and concentrated under reduced pressure. The crude residue was purified by flash column chromatography (SiO_2; 75:25 petroleum ether 40/60:EtOAc) to yield (*R*,*R*)-**3** (105 mg, 65%) as a gummy white solid.

$[\alpha]_D^{24}$**:** +14.0 (*c* = 1.0, $CHCl_3$)

All other physical data matched that given for (*S*,*S*)-**3**.

(3*S*,5*R*)-1-(4-Methoxyphenyl)undecane-3,5-diol (*S*,*R*-3):

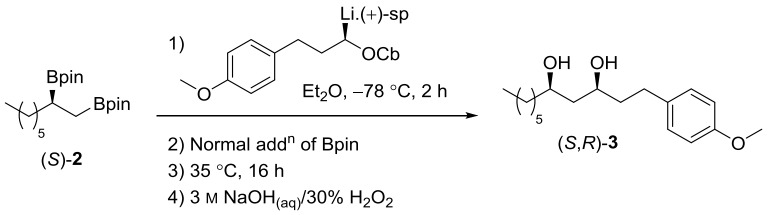

3-(4-Methoxyphenyl)propyl diisopropylcarbamate (167 mg, 0.57 mmol, 1.05 eq.), (+)-sparteine (0.13 mL, 0.57 mmol, 1.05 eq.) and anhydrous Et_2O (2.85 mL) were added to a flame-dried Schlenk-tube purged with N_2. The solution was cooled to −78 °C (dry ice/acetone) before adding *sec*-BuLi (0.42 mL, 1.30 M, 0.55 mmol, 1.00 eq.) dropwise over 5 min and leaving to react for 2 hr at this temperature. (*S*)-**2** (242 mg, 0.66 mmol, 1.20 eq.) was dissolved in anhydrous Et_2O (0.66 mL) and added dropwise to the reaction mixture over 1 min before leaving to react for a further 1 hr at the same temperature. After warming to ambient temperature the Schlenk-tube was sealed and heated at 35 °C (oil bath) for 16 hr. The flask was allowed to cool to ambient temperature before adding THF (2.85 mL) and one crystal of BHT, and was then cooled to 0 °C (ice/water). A 2:1 v:v mixture of 3 M aqueous NaOH (4 mL) and 30% aqueous H_2O_2 (2 mL) was prepared at 0 °C (ice/water) and degassed by gently bubbling N_2 through the solution. This aqueous solution was added dropwise to the vigorously stirred reaction mixture, which was subsequently warmed to ambient temperature and allowed to react for 1 hr. 2 M aqueous HCl (10 mL) was carefully added and the reaction mixture was extracted with EtOAc (3×20 mL). The combined organic fractions were dried over Na_2SO_4, filtered and concentrated under reduced pressure. The crude residue was purified by flash column chromatography (SiO_2; 75:25 petroleum ether 40/60:EtOAc) to yield (*S*,*R*)-**3** (117 mg, 72%) as a colorless oil.

$[\boldsymbol{\alpha}]_{\mathbf{D}}^{\mathbf{24}}$**:** −15.0 ($c$ = 1.0, $CHCl_3$)

All other physical data matched that given for (*R*,*S*)-**3**.

(3*S*,4*S*,6*S*)-4-Hexyl-1,8-bis(4-methoxyphenyl)octane-3,6-diol (3b):

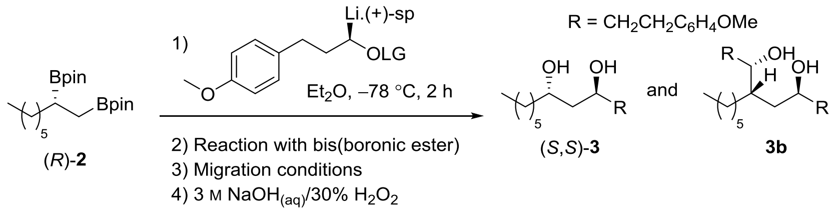

Collected from reactions corresponding to Entries 1-12 from Table 1.

TLC: R_f = 0.26 (60:40 petroleum ether 40/40:EtOAc)

^{1}H NMR: (400 MHz, $CDCl_3$) δ 7.13 – 7.10 (m, 4H, ArH), 6.86 – 6.80 (m, 4H, ArH), 3.79 (s, 3H, OCH_3), 3.79 (s, 3H, OCH_3), 3.83 – 3.77 (m, 1H, CHOH), 3.69 (dt, J = 9.3, 2.8 Hz, 1H, CHOH), 2.80 – 2.67 (m, 2H, $ArCH_2$), 2.60 (m, 2H $ArCH_2$), 2.50 (br s, 2H, OH), 1.82 – 1.63 (m, 6H, CH/CH^aH^b/2×CH_2), 1.47 – 1.39 (m, 1H, CH^aH^b), 1.35 – 1.18 (m, 5×2H), 0.88 (t, J = 6.9 Hz, 1H) ppm

^{13}C NMR: (101 MHz, $CDCl_3$) δ 158.0 (Ar*C*OCH_3), 157.9 (Ar*C*OCH_3), 134.3 (ArC), 134.2 (ArC), 129.4 (ArH), 129.4 (ArH), 114.0 (ArH), 114.0 (ArH), 74.4 (COH), 68.5 (COH), 55.4 (OCH_3), 55.4 (OCH_3), 40.2 (CH), 39.8 (CH_2), 38.7 (CH_2), 35.4 (CH_2), 32.3 (CH_2), 31.9 (CH_2), 31.6 (CH_2), 29.7 (CH_2), 29.5 (CH_2), 27.8 (CH_2), 22.8 (CH_2), 14.2 (CH_3) ppm

HRMS (*m*/*z*): (ESI) calc'd for $C_{28}H_{42}O_4Na$ $[M+Na]^+$: 465.2975, found: 465.2964

IR (neat) ν_{max}: 3310, 2927, 2855, 1747, 1612, 1511, 1243, 1176, 1036, 823 and 753 cm^{-1}

$[\alpha]_D^{23}$: +2.0 (c = 1.0, $CHCl_3$)

2.5 Rationalizing the regioselectivity

We attempted to probe the reason we were getting such high regioselectivity for the primary boronic ester of a 1,2-bis(boronic ester).

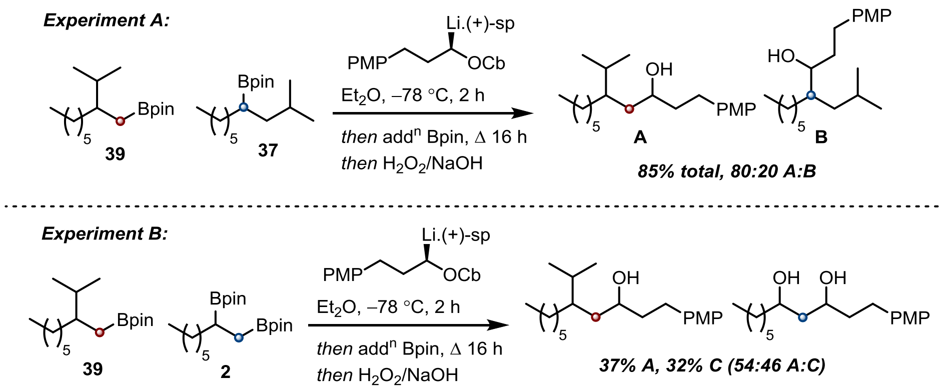

Experiment A shows, as expected, that (hindered) sparteine-ligated carbenoids will preferentially react with primary boronic esters if given the choice between a primary (**39**) and a secondary boronic ester (**37**) in a ratio of 80:20. Presumably the less sterically hindered primary boronic ester is able to react faster with the carbenoid than the more sterically hindered secondary boronic ester. **Experiment B** aimed to probe whether the primary boronic ester of the 1,2-bis(boronic ester) **2** is electronically activated by the neighbouring secondary boronic ester and thus more reactive than a standard primary boronic ester, such as **39**. The results show that both primary boronic esters have roughly equal reactivity and show that there is no electronic effect operating. The slight preference for reaction of **39**, the standard primary boronic ester, probably reflects that increased steric hindrance around the primary boronic ester of the 1,2-bis(boronic ester).

Experiment A:

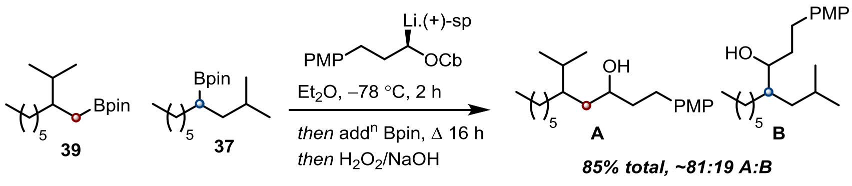

sec-BuLi (0.42 mL, 1.30 M, 0.55 mmol, 1.00 eq.) was added dropwise to a solution of **1a** (168 mg, 0.57 mmol, 1.05 eq.) and (+)-sparteine (0.13 mL, 0.57 mmol, 1.05 eq.) in anhydrous Et_2O (2.87 mL) at –78 °C (dry ice/acetone). After 2 h, a 1:1 mixture of **39** (154 mg, 0.55 mmol, 1.00 eq.) and **37** (154 mg, 0.55 mmol, 1.00 eq.) in anhydrous Et_2O (0.55 mL) was quickly added and the resulting solution was left to react for a further 1 h. After warming to ambient temperature the solution was heated at 35 °C (oil bath) for 16 h. After cooling to ambient

temperature HCl (5 mL, 2 M aqueous solution) was added the the phases separated. The aqueous phase was extracted with Et_2O (3 × 5 mL), dried over $MgSO_4$, filtered and concentrated under reduced pressure. The broad regions containing unreacted starting boronic esters and homologated product boronic esters were separated by flash column chromatography (SiO_2; 97:3 pentane:Et_2O). The ratio of remaining starting boronic esters was 80:20 **37**:**39** by ^{13}C NMR. The mixture of products (174 mg, 85%) was dissolved in THF (6 mL) and cooled to 0 °C, and then a 2:1 v:v mixture of 3 M aqueous NaOH (4 mL) and 30% aqueous H_2O_2 (2 mL) was prepared at 0 °C (ice/water) and degassed by gently bubbling N_2 through the solution. This aqueous solution was added dropwise to the vigorously stirred reaction mixture, which was subsequently warmed to ambient temperature and allowed to react for 1 hr. 2 M aqueous HCl (10 mL) was carefully added and the reaction mixture was extracted with EtOAc (3×10 mL). The combined organic fractions were dried over Na_2SO_4, filtered and concentrated under reduced pressure. The crude residue was analysed by ^{13}C NMR and found to contain a 81:19 ratio of **A**:**B**.

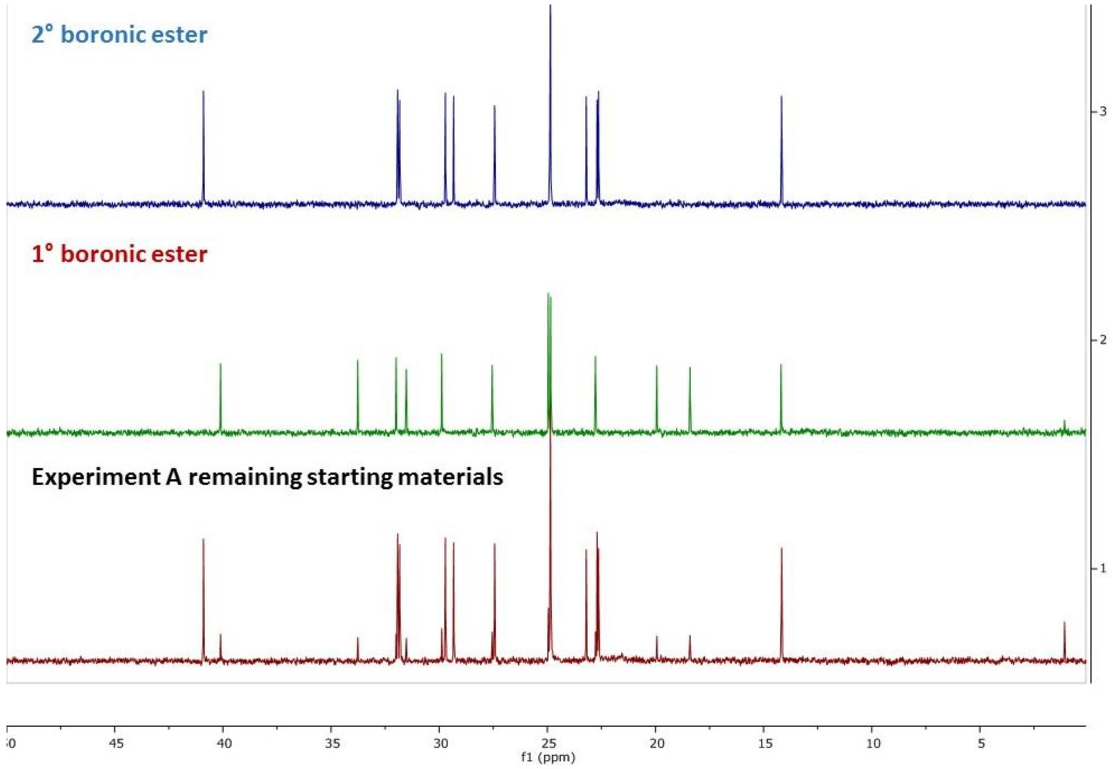

Experiment A oxidised products:

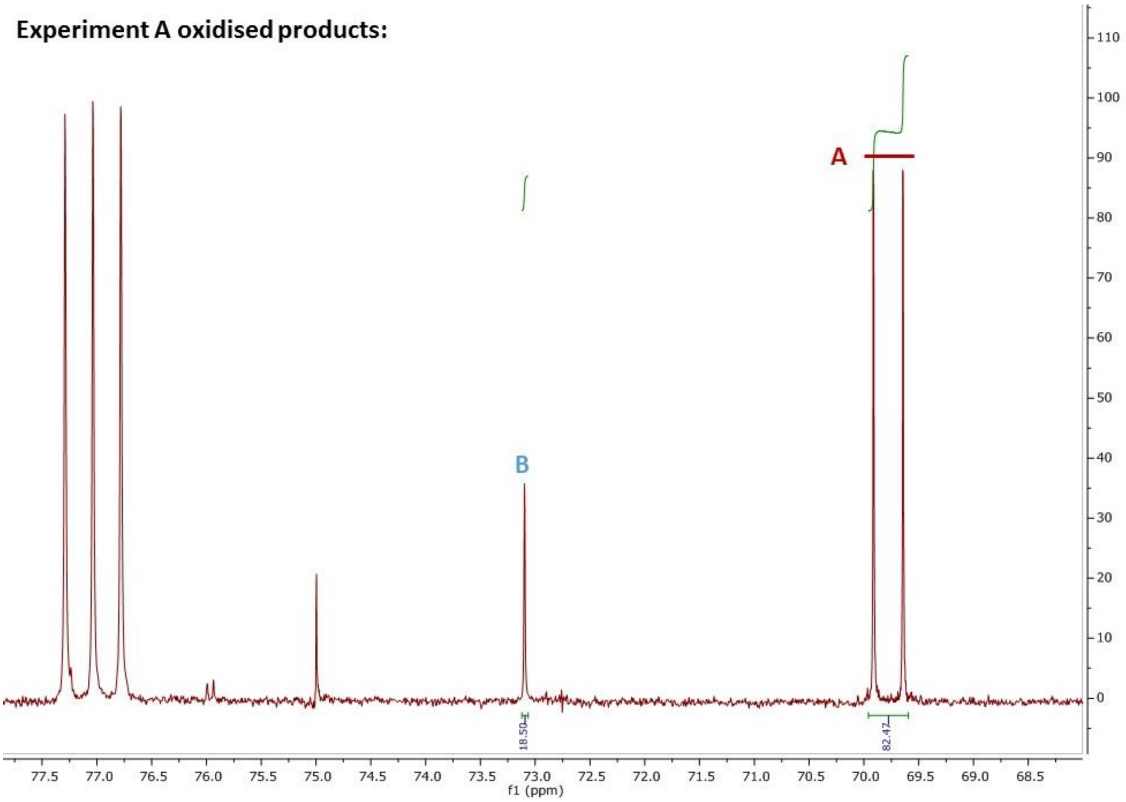

Experiment B:

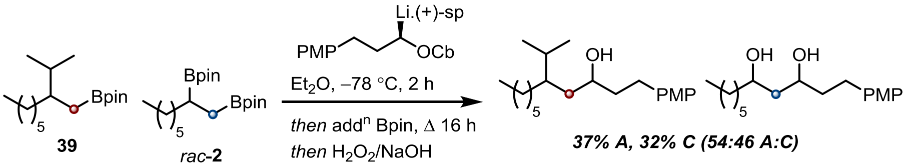

sec-BuLi (0.27 mL, 1.30 M, 0.35 mmol, 1.00 eq.) was added dropwise to a solution of **1a** (108 mg, 0.37 mmol, 1.05 eq.) and (+)-sparteine (0.08 mL, 0.37 mmol, 1.05 eq.) in anhydrous Et_2O (1.84 mL) at –78 °C (dry ice/acetone). After 2 h, a 1:1 mixture of **39** (99 mg, 0.35 mmol, 1.00 eq.) and *rac*-**2** (129 mg, 0.35 mmol, 1.00 eq.) in anhydrous Et_2O (0.35 mL) was quickly added and the resulting solution was left to react for a further 1 h. After warming to ambient temperature the solution was heated at 35 °C (oil bath) for 16 h. The flask was allowed to cool to ambient temperature before adding THF (2 mL), and was then cooled to 0 °C (ice/water). A 2:1 v:v mixture of 3 M aqueous NaOH (2 mL) and 30% aqueous H_2O_2 (1 mL) was prepared at 0 °C (ice/water) and degassed by gently bubbling N_2 through the solution. This aqueous solution was added dropwise to the vigorously stirred reaction mixture, which was subsequently warmed to ambient temperature and allowed to react for 1 hr. 2 M aqueous HCl (5 mL) was carefully added and the reaction mixture was extracted with EtOAc (3 × 10 mL). The combined organic fractions were dried over Na_2SO_4, filtered and concentrated under

reduced pressure. The crude residue was analysed by ^{13}C NMR and found to contain a 54:46 ratio of **A**:**C**.

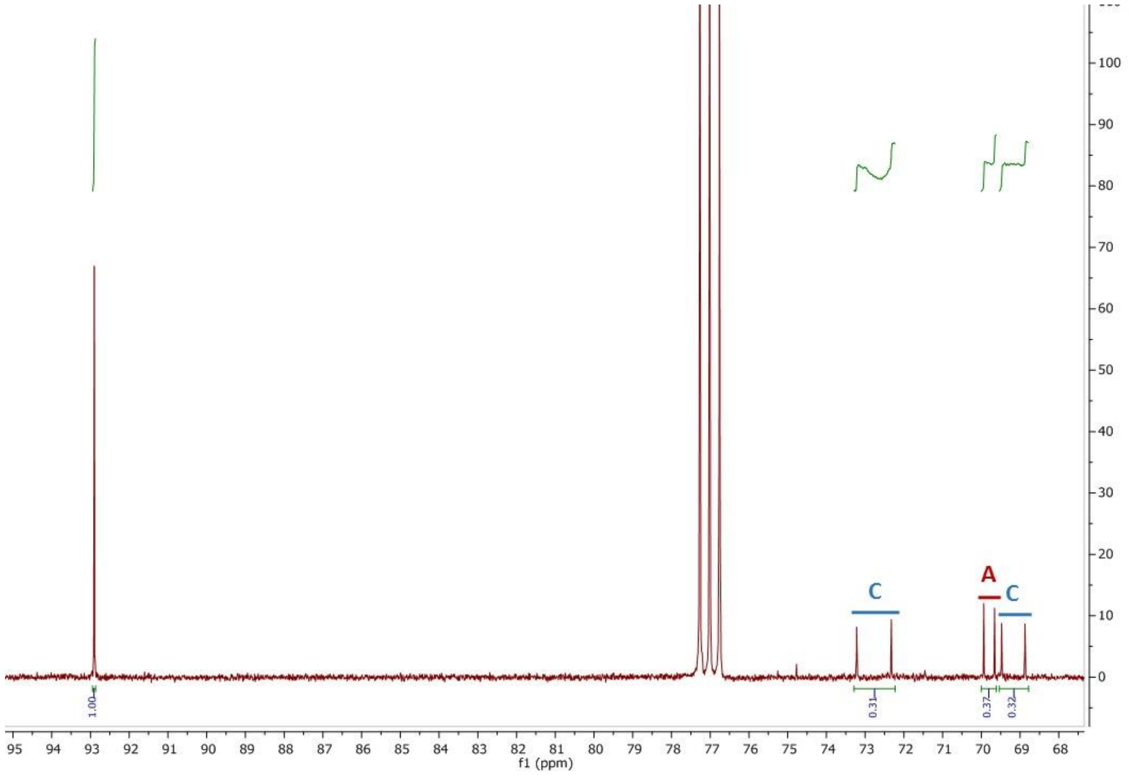

3. Regioselective Homologation of 1,2-bis(boronic esters): Substrate Scope

3.1 Synthesis of Starting Materials

3-((*tert*-Butyldimethylsilyl)oxy)propan-1-ol (**41**)[12], pent-4-en-1-yl diisopropylcarbamate (**42**)[13], (*S*)-*tert*-butyl(pent-4-en-2-yloxy)diphenylsilane (**43**),[14] (*S*)-4,8-dimethylnona-1,7-diene (**44**)[15], *tert*-butyl(((2*R*,4*S*)-2,4-dimethylhex-5-en-1-yl)oxy)diphenylsilane (**45**)[16], isobutyl 2,4,6-triisopropylbenzoate (**32**)[6], (2*R*,4*S*)-5-(methoxymethoxy)-2,4-dimethylpentyl diisopropylcarbamate (**46**)[17], (*S*,*E*)-pent-3-en-2-yl diisopropylcarbamate (**47**)[18], (*S*)-4-phenyl-2-(trimethylstannyl)butan-2-yl 2,4,6-triisopropylbenzoate (**48**)[19], (*S*)-1-(4-

[12] M. S. Mortensen, J. M. Osbourn and G. A. O'Doherty, *Org. Lett.* **2007**, *9*, 3105.
[13] M. J. Hesse, C. P. Butts, C. L. Willis and V. K. Aggarwal, *Angew. Chem. Int. Ed.* **2012**, *51*, 12444.
[14] S. Bujaranipalli and S. Das, *Tetrahedron Lett.* **2015**, *56*, 3747.
[15] G. Zhu, B. Liang and E.-i. Negishi, *Org. Lett.* **2008**, *10*, 1099.
[16] A. B. Smith III, K. Basu and T. Bosanac, *J. Am. Chem. Soc.* **2007**, *129*, 14872.
[17] R. Rasappan and V. K. Aggarwal, *Nat. Chem.* **2014**, *6*, 810.
[18] A. P. Pulis and V. K. Aggarwal, *J. Am. Chem. Soc.* **2012**, *134*, 11298.
[19] C. G. Watson, A. Balanta, T. G. Elford, S. Essafi, J. N. Harvey and V. K. Aggarwal, *J. Am. Chem. Soc.* **2014**, *136*, 17370.

methoxyphenyl)ethyl diisopropylcarbamate (**49**)[20], (*S*)-1-phenylethyl diisopropylcarbamate (**50**)[21] and (*S*)-1-(4-fluorophenyl)ethyl diisopropylcarbamate (**51**)[21] were synthesised according to literature procedures. All data matched that reported.

3-((*tert*-Butyldimethylsilyl)oxy)propyl diisopropylcarbamate (52):

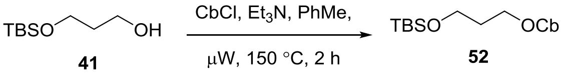

3-((*tert*-Butyldimethylsilyl)oxy)propan-1-ol (**41**) (2.00 g, 10.51 mmol, 1.00 eq.), diisopropylcarbamoyl chloride (2.06 g, 12.61 mmol, 1.20 eq.), triethylamine (1.90 mL, 13.66 mmol, 1.30 eq.) and anhydrous toluene (10.51 mL) were added to a flame-dried microwave vial. After sealing the reaction vessel was heated in a microwave reactor for 2 h at 150 °C. After cooling to ambient temperature the mixture was filtered through a short plug of silica, eluting with diethyl ether (30 mL), and concentrated under reduced pressure. The crude residue was purified by flash column chromatography (SiO_2; 90:10 pentane:Et_2O) to yield **52** (2.68 g, 80%) as a colorless oil.

TLC: R_f = 0.36 (80:20 pentane:Et_2O)

^{1}H NMR: (500 MHz, $CDCl_3$) δ 4.14 (t, J = 6.3 Hz, 2H, $COOCH_2$), 4.22 – 3.58 (br m, 2H, $NCH(CH_3)_2$), 3.70 (t, J = 6.3 Hz, 2H, CH_2OSi), 1.85 (p, J = 6.3 Hz, 2H, CH_2), 1.18 (d, J = 6.8 Hz, 12H, $NCH(CH_3)_2$), 0.87 (s, 9H, $SiC(CH_3)_3$), 0.03 (s, 6H, $Si(CH_3)_2$) ppm

^{13}C NMR: (126 MHz, $CDCl_3$) δ 155.8 (C=O), 61.6 ($COOCH_2$), 60.1 (CH_2OSi), 46.1 (br, $NCH(CH_3)_2$), 45.5 (br, $NCH(CH_3)_2$), 32.5 (CH_2), 26.0 ($SiC(CH_3)_3$), 21.1 (br, $NCH(CH_3)_2$), 18.4 ($SiC(CH_3)_3$), −5.3 ($Si(CH_3)_2$) ppm

HRMS (*m*/*z*): (ESI) calc'd for $C_{16}H_{35}O_3NSiNa$ $[M+Na]^+$: 340.2278, found: 340.2265

IR (neat) ν_{max}: 2957, 2930, 2858, 1692 (CO), 1435, 1308, 1288, 1071, 834 and 772 cm^{-1}

[20] S. Roesner, C. A. Brown, M. Mohiti, A. P. Pulis, R. Rasappan, D. J. Blair, S. Essafi, D. Leonori and V. K. Aggarwal, *Chem. Commun.* **2014**, *50*, 4053.
[21] V. Bagutski, R. M. French and V. K. Aggarwal, *Angew. Chem. Int. Ed.* **2010**, *49*, 5142.

tert-Butyl hept-6-enoate (53):

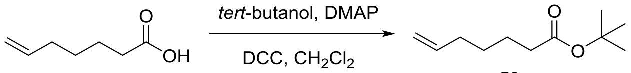

DCC (11.42 g, 55.36 mmol, 1.50 eq) was added in one portion to a solution of 6-heptenoic acid (5.00 mL, 36.90 mmol, 1.00 eq), *tert*-butanol (49.41 mL, 516.66 mmol, 14.00 eq.) and DMAP (902 mg, 7.38 mmol, 0.20 eq.) in dichloromethane (95 mL) at 0 °C (ice water). The solution was allowed to warm to ambient temperature and stir for 48 h. The solution was then filtered through a wetted plug of celite, eluting with dichloromethane (50 mL). The combined solutions were washed with $NaHCO_3$ (sat. aq. solution, 50 mL), dried over $MgSO_4$, filtered and concentrated under reduced pressure. The crude residue was purified by flash column chromatography (SiO_2; 90:10 pentane:CH_2Cl_2) to yield **53** (2.32 g, 34%) as a colorless oil.

TLC: R_f = 0.10 (90:10 pentane:CH_2Cl_2)

^{1}H NMR: (400 MHz, $CDCl_3$) δ 5.79 (ddt, *J* = 16.9, 10.2, 6.7 Hz, 1H, =CH), 5.03 – 4.97 (m, 1H, =CH^aH^b), 4.96 – 4.92 (m, 1H, =CH^aH^b), 2.21 (t, *J* = 7.5 Hz, 2H, CH_2CO), 2.06 (app q, J = 7.2 Hz, 2H, =CHCH_2), 1.64 – 1.54 (m, 2H, C$H_2$$CH_2$CO), 1.44 (s, 9H, OC$(CH_3)_3$), 1.46 – 1.37 (m, 2H, CH_2) ppm

^{13}C NMR: (101 MHz, $CDCl_3$) δ 173.3 (C=O), 138.7 (CH=), 114.7 (=CH_2), 80.1 (O*C*$(CH_3)_3$), 35.6 (*C*H_2CO), 33.6 (*C*H_2CH=), 28.5 (CH_2), 28.3 (OC(*C*H_3)$_3$), 24.7 (*C*$H_2$$CH_2$CO) ppm

LRMS (*m/z*): (EI) 57 ([^{t}Bu]$^+$, 100%), 83 ([M–CO_2^{t}Bu]$^+$, 34%), 111 ([M–O^{t}Bu]$^+$, 46%), 128 ([M–^{t}Bu]$^+$, 25%). *Despite repeated attempts, a HRMS could not be obtained.*

IR (neat) ν_{max}: 2978, 2932, 1730 (CO), 1367, 1148 and 910 cm^{-1}

tert-Butyl (*R*)-6,7-bis(4,4,5,5-tetramethyl-1,3,2-dioxaborolan-2-yl)heptanoate (54):

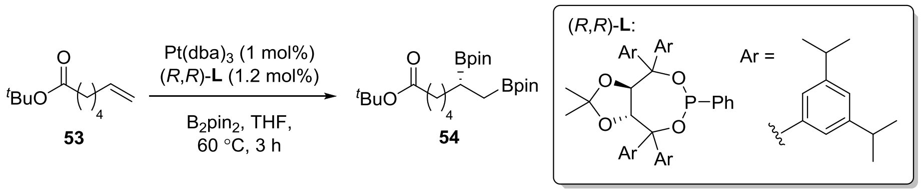

Pt(dba)$_3$ (49 mg, 0.05 mmol, 1.0 mol%), (*R*,*R*)-**L** (59 mg, 0.07 mmol, 1.2 mol%) and B_2pin_2 (1.45 g, 5.70 mmol, 1.05 eq.) were added to a flame-dried Schlenk-tube purged with N_2. THF

(5.43 mL) was added before sealing the flask and heating at 80 °C (oil bath) for 30 mins. After cooling to ambient temperature **53** (1.0 g, 5.43 mmol, 1.00 eq.) was added before re-sealing and heating for 3 hr at 60 °C (oil bath). The solution was then cooled to ambient temperature and concentrated under reduced pressure. The crude residue was directly purified by flash column chromatography (SiO_2; 85:15 pentane:Et_2O) to yield **54** (2.09 g, 92%) as a pale yellow viscous oil.

TLC: R_f = 0.20 (85:15 pentane:Et_2O)

^{1}H NMR: (400 MHz, $CDCl_3$) δ 2.17 (t, J = 7.6 Hz, 2H, $COCH_2$), 1.55 (p, J = 7.6 Hz, 2H, $COCH_2CH_2$), 1.49 (m, 5H, 2×CH_2 and CHBpin), 1.42 (s, 9H, $OC(CH_3)_3$), 1.21 (s, 24H, $C(CH_3)_2$), 0.85 (dd, J = 15.8, 9.5 Hz, CH^aH^bBpin), 0.77 (dd, J = 15.8, 5.9 Hz, CH^aH^bBpin) ppm

^{13}C NMR: (101 MHz, $CDCl_3$) δ 173.5 (C=O), 83.0 ($C(CH_3)_2$), 82.9 ($C(CH_3)_2$), 79.9 ($OC(CH_3)_3$), 35.8 ($COCH_2$), 33.5 (CH_2), 28.5 (CH_2), 28.3 ($OC(CH_3)_3$), 25.5 (CH_2), 25.0 ($C(CH_3)_2$), 25.0 ($C(CH_3)_2$), 24.9 ($C(CH_3)_2$), 24.9 ($C(CH_3)_2$) ppm

HRMS (*m/z*): (ESI) calc'd for $C_{23}H_{44}B_2O_6Na$ $[M+Na]^+$: 461.3224, found: 461.3226

IR (neat) ν_{max}: 2977, 2930, 1730 (CO), 1368, 1311, 1140, 968 and 846 cm^{-1}

$[\alpha]_D^{24}$: +3.0 (c = 1.0, $CHCl_3$)

rac-**54** was synthesised using the following procedure:

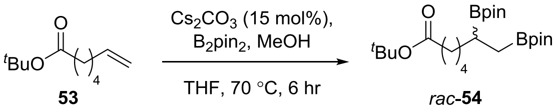

Cs_2CO_3 (133 mg, 0.41 mmol, 15 mol%) and B_2pin_2 (758 mg, 2.98 mmol, 1.10 eq.) were added to a flame-dried Schlenk-tube purged with N_2. THF (10.9 mL), MeOH (0.55 mL, 13.57 mmol, 5.00 eq.) and **53** (500 mg, 2.71 mmol, 1.00 eq.) were then added and the mixture was heated at 70 °C (oil bath) for 6 hr. The reaction mixture was cooled to ambient temperature and concentred under reduced pressure. The crude residue was directly purified by flash column chromatography (SiO_2; 85:15 pentane:Et_2O) to yield *rac*-**54** (923 mg, 78%) as a viscous colorless oil.

The collected data was identical to that described above.

Aliquots of the reaction mixtures were oxidised and protected as acetonides[6] for analysis of enantiomeric purity by chiral-GC.

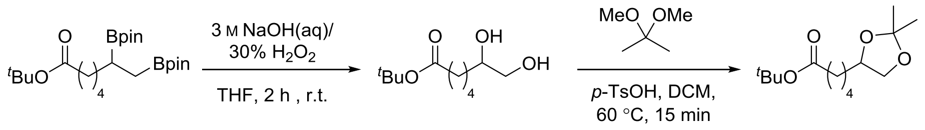

Chiral-GC: Chiraldex β, injector T = 250 °C, detector T = 300 °C. Oven conditions: T = 70 °C for 5 min, then ramp (1 °C/min) until 180 °C and hold for 5 min. He carrier gas at 1.0 mL/min.

Racemic:

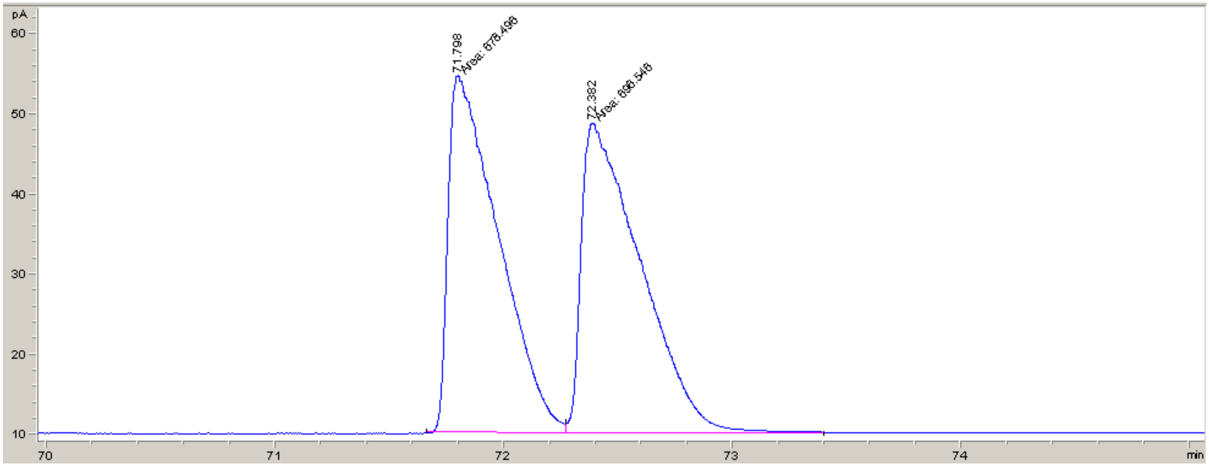

(R)-**54** (using (R,R)-**L**): 96.0:4.0 e.r. [t_R= 72.07 min (minor), t_R = 72.35 min (major)]

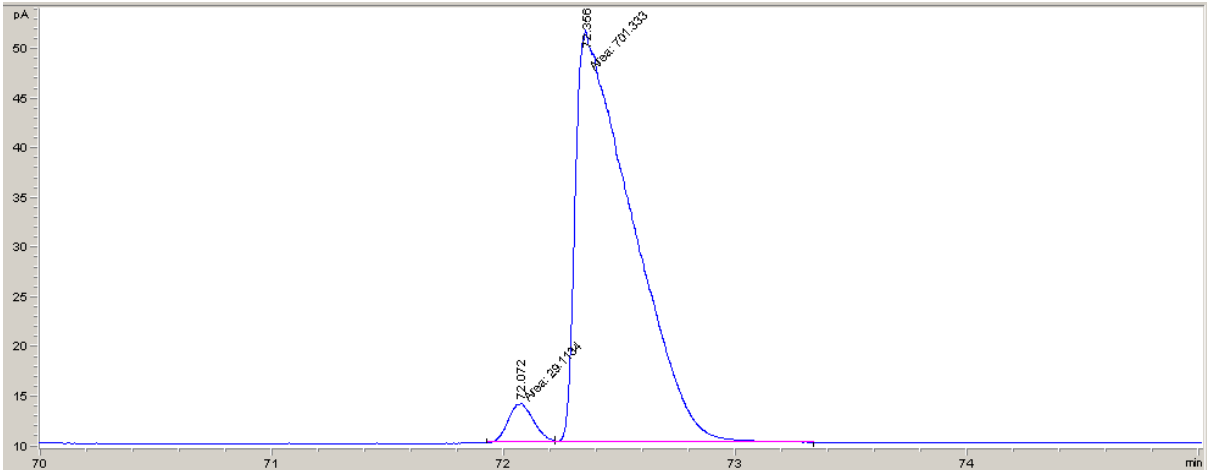

(*R*)-2,2'-(1-(4-Methoxyphenyl)ethane-1,2-diyl)bis(4,4,5,5-tetramethyl-1,3,2-dioxaborolane) (55):

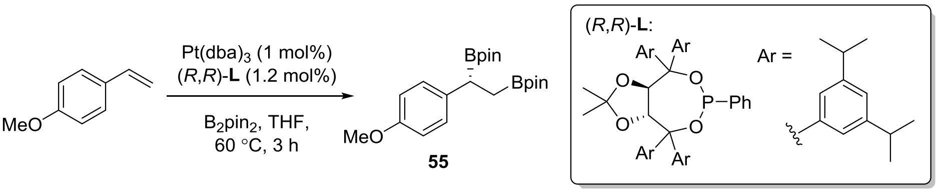

$Pt(dba)_3$ (34 mg, 0.04 mmol, 1.0 mol%), (*R*,*R*)-**L** (41 mg, 0.05 mmol, 1.2 mol%) and B_2pin_2 (1.00 g, 3.95 mmol, 1.05 eq.) were added to a flame-dried Schlenk-tube purged with N_2. THF (3.80 mL) was added before sealing the flask and heating at 80 °C (oil bath) for 30 mins. After cooling to ambient temperature 4-vinylanisole (0.50 mL, 3.76 mmol, 1.00 eq.) was added before re-sealing and heating for 3 hr at 60 °C (oil bath). The solution was then cooled to ambient temperature and concentrated under reduced pressure. The crude residue was directly purified by flash column chromatography (SiO_2; 90:10 pentane:Et_2O) to yield **55** (1.16 g, 80%) as a colorless paste.

TLC: R_f = 0.23 (80:20 pentane:Et_2O)

^{1}H NMR: (400 MHz, $CDCl_3$) δ 7.14 (d, J = 8.6 Hz, 2H, ArH), 6.78 (d, J = 8.6 Hz, 2H, ArH), 3.76 (s, 3H, OCH_3), 2.46 (dd, J = 10.9, 5.8 Hz, 1H, ArCHBpin), 1.33 (dd, J = 16.0, 10.9 Hz, 1H, C$H^a$$H^b$Bpin), 1.20 (s, 12H, $C(CH_3)_2$), 1.19 (s, 6H, $C(CH_3)_2$), 1.17 (s, 6H, $C(CH_3)_2$), 1.08 (dd, J = 16.0, 5.8 Hz, 1H, C$H^a$$H^b$Bpin) ppm

^{13}C NMR: (101 MHz, $CDCl_3$) δ 157.3 (Ar*C*OMe), 137.6 (ArC), 128.9 (ArH), 113.8 (ArH), 83.3 (*C*$(CH_3)_2$), 83.1 (*C*$(CH_3)_2$), 55.3 (OCH_3), 25.1 (C(*C*$H_3)_2$), 24.8 (C(*C*$H_3)_2$), 24.8 (C(*C*$H_3)_2$), 24.6 (C(*C*$H_3)_2$) ppm

HRMS (*m/z*): (ESI) calc'd for $C_{21}H_{34}B_2O_5Na$ $[M+Na]^+$: 411.2492, found: 411.2483

IR (neat) ν_{max}: 2978, 2933, 1509, 1369, 1317, 1244, 1033, 967, 851, 828 and 673 cm^{-1}

$[\alpha]_D^{24}$**:** −14.0 (c = 1.0, $CHCl_3$)

rac-**55** was synthesised using the following procedure:

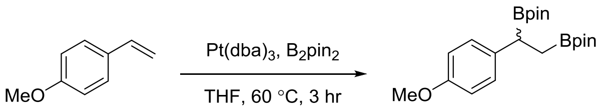

Pt(dba)$_3$ (34 mg, 0.04 mmol, 1.0 mol%), B$_2$pin$_2$ (1.00 g, 3.95 mmol, 1.05 eq.) and 4-vinylanisole (0.50 mL, 3.76 mmol, 1.00 eq.) were added to a flame-dried Schlenk-tube purged with N_2. THF (3.76 mL) was added before sealing the flask and heating at 60 °C (oil bath) for 3 h. The solution was then cooled to ambient temperature and concentrated under reduced pressure. The crude residue was directly purified by flash column chromatography (SiO_2; 90:10 pentane:Et$_2$O) to yield *rac*-**55** (887 mg, 54%) as a colorless paste.

The collected data was identical to that described above.

Chiral SFC: Chiralpak Whelk-01, 125 bar, 40 °C, 4 mL/min, 10% co-colvent (50% IPA/hexane); t_R = 10.4 min (minor), t_R = 11.8 min (major), 94:6 e.r.

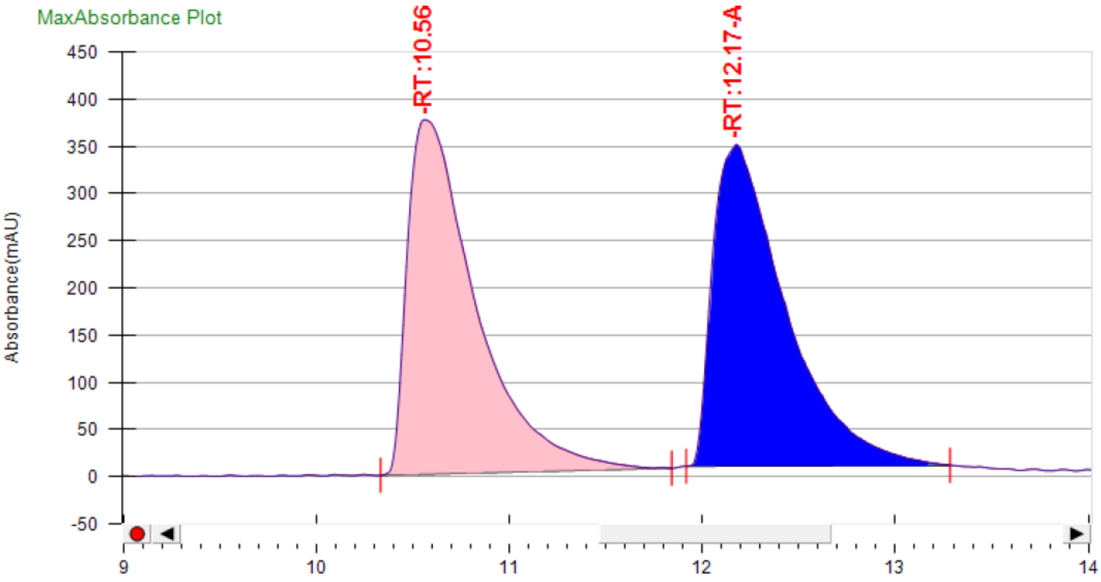

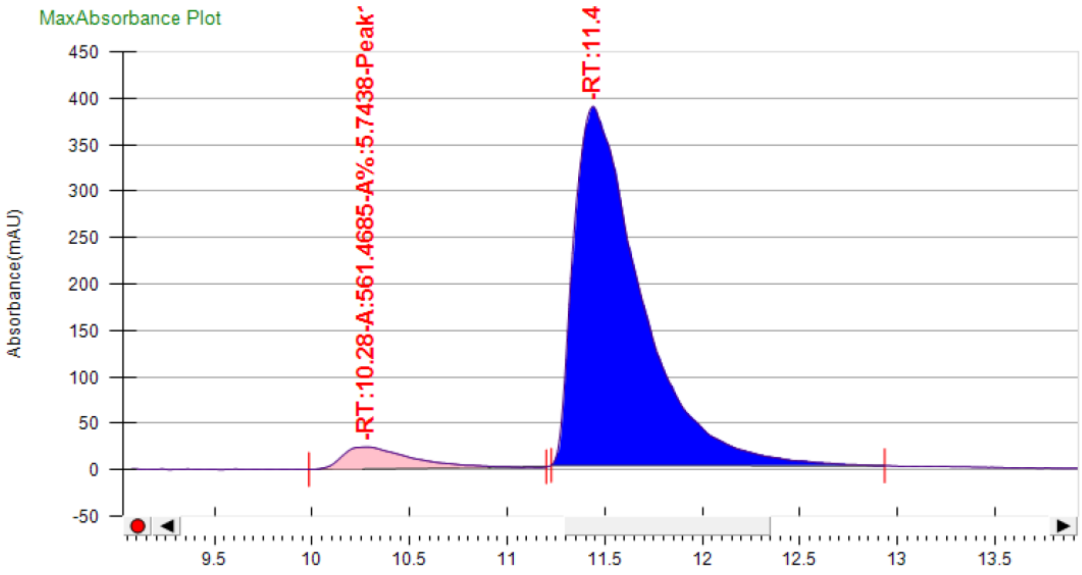

Peak Information

Peak No	% Area	Area	Ret. Time	Height	Cap. Factor
1	5.7438	561.4685	10.28 min	23.5166	0
2	94.2562	9213.6621	11.44 min	386.2789	0

(*R*)-4,5-Bis(4,4,5,5-tetramethyl-1,3,2-dioxaborolan-2-yl)pentyl diisopropylcarbamate (56):

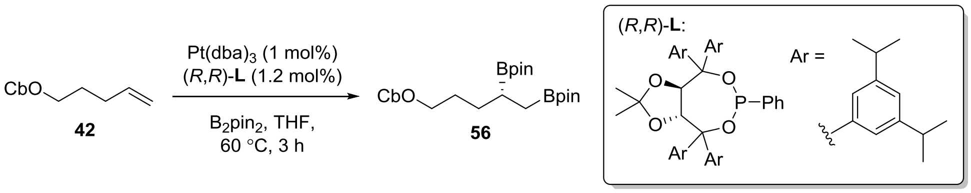

$Pt(dba)_3$ (42 mg, 0.05 mmol, 1.0 mol%), (*R*,*R*)-**L** (51 mg, 0.06 mmol, 1.2 mol%) and B_2pin_2 (1.25 g, 4.92 mmol, 1.05 eq.) were added to a flame-dried Schlenk-tube purged with N_2. THF (4.69 mL) was added before sealing the flask and heating at 80 °C (oil bath) for 30 mins. After cooling to ambient temperature **42** (1.00 g mL, 4.69 mmol, 1.00 eq.) was added before re-sealing and heating for 3 hr at 60 °C (oil bath). The solution was then cooled to ambient temperature and concentrated under reduced pressure. The crude residue was directly purified by flash column chromatography (SiO_2; 70:30 pentane:Et_2O) to yield **56** (1.91 g, 86%) as a pale yellow viscous oil.

TLC: R_f = 0.21 (70:30 pentane:Et_2O)

^{1}H NMR: (400 MHz, $CDCl_3$) δ 4.23 – 3.51 (m, 2H, NC*H*(CH_3)$_2$), 4.03 (t, *J* = 6.5 Hz, 2H, OCH_2), 1.70 – 1.61 (m, 2H, OCH_2CH_2), 1.60 – 1.48 (m, 1H, CH^aH^b), 1.46 – 1.33 (m, 1H, CH^aH^b), 1.22 (s, 12H, $C(CH_3)_2$), 1.22 (s, 12H, $C(CH_3)_2$), 1.18 (d, *J* = 1.18 Hz, 12H, NCH(C*H*$_3$)$_2$), 0.88 (dd, *J* = 15.8, 9.6 Hz, 1H, CH^aH^bBpin), 0.80 (dd, *J* = 15.8, 5.8 Hz, CH^aH^bBpin) ppm

^{13}C NMR: (101 MHz, $CDCl_3$) δ 156.1 (C=O), 83.0 (*C*(CH_3)$_2$), 83.0 (*C*(CH_3)$_2$), 65.3 (OCH_2), 45.6 (br, N*C*H(CH_3)$_2$), 30.6 (CH_2), 28.6 (OCH_2CH_2), 25.0 (C(*C*H_3)$_2$), 25.0 (C(*C*H_3)$_2$), 24.9 (C(*C*H_3)$_2$), 24.9 (C(*C*H_3)$_2$), 21.0 (br, NCH(*C*H_3)$_2$) ppm

HRMS (*m/z*): (ESI) calc'd for $C_{24}H_{47}B_2O_6NNa$ $[M+Na]^+$: 490.3490, found: 490.3483

IR (neat) ν_{max}: 2976, 2932, 1689 (CO), 1369, 1308, 1289, 1139, 968, 846 and 773 cm^{-1}

$[\alpha]_D^{24}$: –5.0 (*c* = 1.0, $CHCl_3$)

rac-**56** was synthesised using the following procedure:

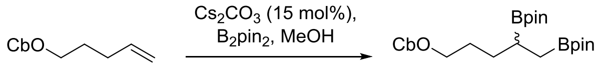

Cs_2CO_3 (115 mg, 0.35 mmol, 15 mol%) and B_2pin_2 (655 mg, 2.58 mmol, 1.10 eq.) were added to a flame-dried Schlenk-tube purged with N_2. THF (9.40 mL), MeOH (0.47 mL, 11.72 mmol, 5.00 eq.) and **42** (500 mg, 2.34 mmol, 1.00 eq.) were then added and the mixture was heated at 70 °C (oil bath) for 6 hr. The reaction mixture was cooled to ambient temperature and concentred under reduced pressure. The crude residue was directly purified by flash column chromatography (SiO_2; 80:20 pentane:Et_2O) to yield *rac*-**56** (730 mg, 66%) as a colorless oil.

The collected data was identical to that described above.

Aliquots of the reaction mixtures were oxidised and protected as acetonides[6] for analysis of enantiomeric purity by chiral-GC.

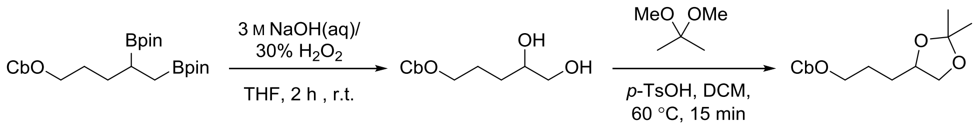

Chiral-GC: Chiraldex β, injector T = 250 °C, detector T = 300 °C. Oven conditions: T = 70 °C for 5 min, then ramp (1 °C/min) until 180 °C and hold for 5 min. He carrier gas at 1.0 mL/min.

Racemic:

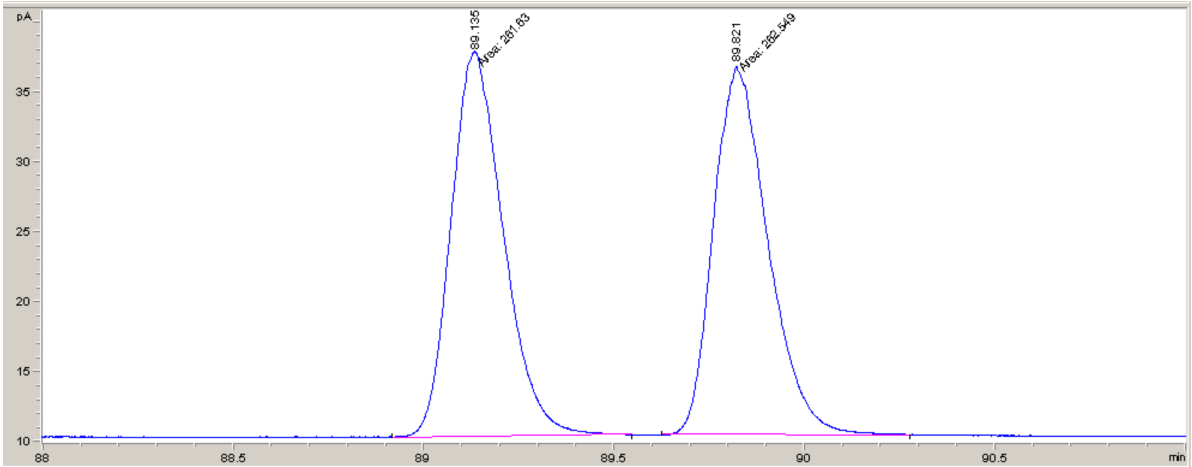

(*R*)-**56** (using (*R*,*R*)-**L**): 90.0:10.0 e.r. [t_R= 89.21 min (minor), t_R = 89.79 min (major)]

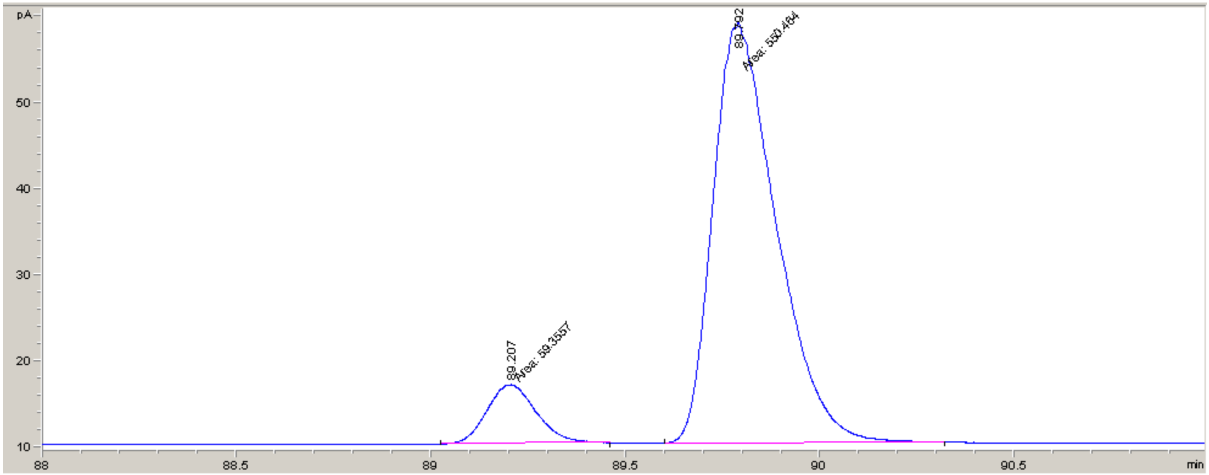

(*R*)-2,2'-(4,4-dimethylpentane-1,2-diyl)bis(4,4,5,5-tetramethyl-1,3,2-dioxaborolane) (57):

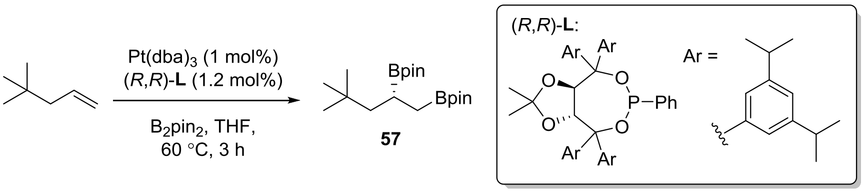

Pt(dba)$_3$ (63 mg, 0.07 mmol, 1.0 mol%), (*R*,*R*)-**L** (76 mg, 0.08 mmol, 1.2 mol%) and B$_2$pin$_2$ (1.85 g, 7.30 mmol, 1.05 eq.) were added to a flame-dried Schlenk-tube purged with N_2. THF

(7.00 mL) was added before sealing the flask and heating at 80 °C (oil bath) for 30 mins. After cooling to ambient temperature 4,4-dimethyl-1-pentene (1.00 mL, 6.96 mmol, 1.00 eq.) was added before re-sealing and heating for 3 hr at 60 °C (oil bath). The solution was then cooled to ambient temperature and concentrated under reduced pressure. The crude residue was directly purified by flash column chromatography (SiO_2; 95:5 pentane:Et_2O) to yield **57** (2.11 g, 86%) as a white solid.

TLC: R_f = 0.33 (90:10 petroleum ether 60/40:Et_2O)

m.p.: 41 – 44 °C (pentane)

^{1}H NMR: (400 MHz, $CDCl_3$) δ 1.58 (dd, J = 13.0, 10.1 Hz, 1H, CH^aH^b), 1.32 – 1.06 (m, 2H, CHBpin and CH^aH^b), 1.22 (s, 24H, C(CH_3)$_2$), 0.90 – 0.82 (m, 1H, CH^aH^{b}Bpin), 0.73 (dd, *J* = 15.5, 7.9 Hz, 1H, CH^aH^bBpin) ppm

^{13}C NMR: (101 MHz, $CDCl_3$) δ 83.0 (*C*(CH_3)$_2$), 82.8 (*C*(CH_3)$_2$), 48.5 (CH_2), 31.3 (*C*(CH_3)$_3$), 29.9 (C(*C*H_3)$_3$), 25.1 (C(*C*H_3)$_2$), 25.0 (C(*C*H_3)$_2$), 24.9 (C(*C*H_3)$_2$) ppm

HRMS (*m/z*): (ESI) calc'd for $C_{19}H_{38}B_2O_4Na$ $[M+Na]^+$: 375.2855, found: 375.2862

IR (neat) ν_{max}: 2977, 2949, 1352, 1315, 1141, 968, 841 and 671 cm^{-1}

$[\alpha]_D^{24}$**:** +10.0 (*c* = 1.0, $CHCl_3$)

rac-**57** was synthesised using the following procedure:

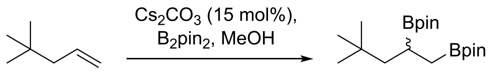

rac-**57**

Cs_2CO_3 (170 mg, 0.52 mmol, 15 mol%) and B_2pin_2 (972 mg, 3.83 mmol, 1.10 eq.) were added to a flame-dried Schlenk-tube purged with N_2. THF (13.90 mL), MeOH (0.70 mL, 17.39 mmol, 5.00 eq.) and 4,4-dimethyl-1-pentene (0.50 mL, 3.48 mmol, 1.00 eq.) were then added and the mixture was heated at 70 °C (oil bath) for 6 hr. The reaction mixture was cooled to ambient temperature and concentred under reduced pressure. The crude residue was directly purified by flash column chromatography (SiO_2; 95:5 pentane:Et_2O) to yield *rac*-**57** (887 mg, 73%) as a colorless amorphous solid.

The collected data was identical to that described above.

Aliquots of the reaction mixtures were oxidised and protected as acetonides[6] for analysis of enantiomeric purity by chiral-GC.

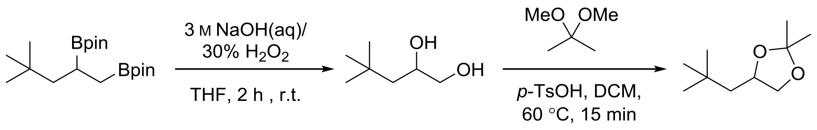

Chiral-GC: Chiraldex β, injector T = 250 °C, detector T = 300 °C. Oven conditions: T = 30 °C for 5 min, then ramp (1 °C/min) until 80 °C and hold for 5 min, then ramp (10 °C/min) until 180 °C and hold for 2 min. He carrier gas at 1.0 mL/min.

Racemic:

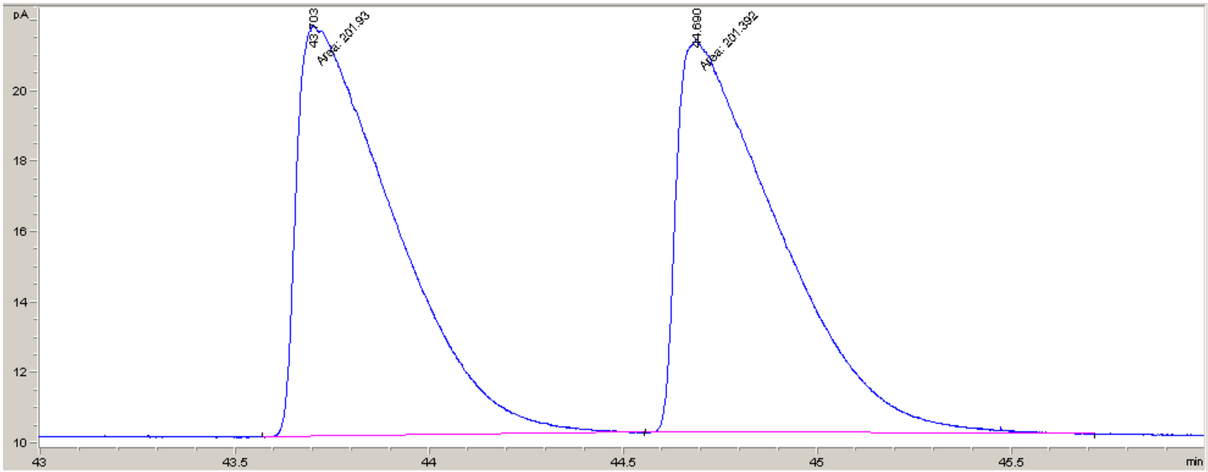

(*R*)-**57** (using (*R*,*R*)-**L**): 98.0:2.0 e.r. [t_R= 43.75 min (minor), t_R = 44.12 min (major)]

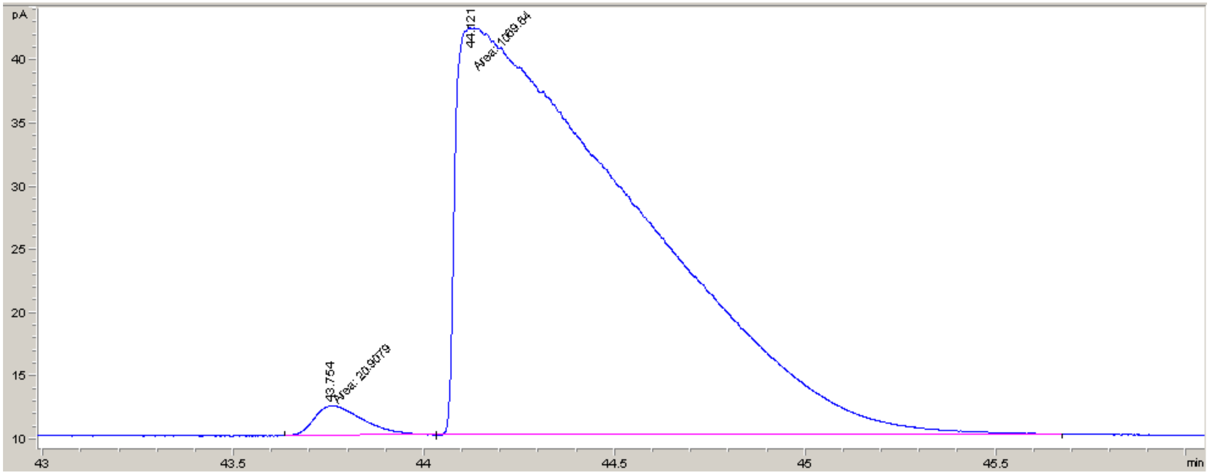

(((2*S*,4*R*)-4,5-Bis(4,4,5,5-tetramethyl-1,3,2-dioxaborolan-2-yl)pentan-2-yl)oxy)(tert-butyl)diphenylsilane (58):

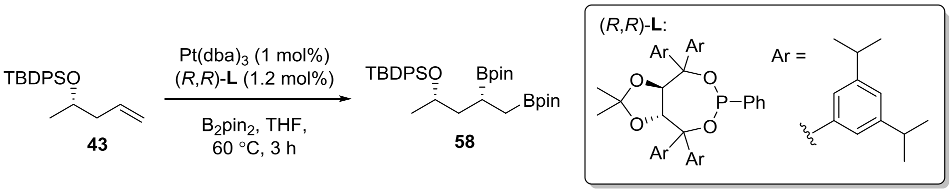

$Pt(dba)_3$ (55 mg, 0.06 mmol, 1.0 mol%), (*R*,*R*)-**L** (67 mg, 0.07 mmol, 1.2 mol%) and B_2pin_2 (1.64 g, 6.47 mmol, 1.05 eq.) were added to a flame-dried Schlenk-tube purged with N_2. THF (6.16 mL) was added before sealing the flask and heating at 80 °C (oil bath) for 30 mins. After cooling to ambient temperature **43** (2.00 g, 6.16 mmol, 1.00 eq.) was added before re-sealing and heating for 3 hr at 60 °C (oil bath). The solution was then cooled to ambient temperature and concentrated under reduced pressure. The crude residue was directly purified by flash column chromatography (SiO_2; 95:5 pentane:Et_2O) to yield **58** (3.21 g, >95:5 d.r., 90%) as a pale yellow viscous oil.

TLC: R_f = 0.26 (90:10 pentane:Et_2O)

^{1}H NMR: (400 MHz, $CDCl_3$) δ 7.72 – 7.67 (m, 4H, ArH), 7.42 – 7.32 (m, 6H, ArH), 3.92 (dqd, *J* = 7.3, 6.0, 5.9 Hz, 1H, CHO), 1.86 (ddd, *J* = 13.2, 8.8, 5.9 Hz, 1H, CH^aH^b with H^a italic: CH^aH^b), 1.42 (app dt, *J* = 13.2, 7.3 Hz, 1H, CH$^a\mathit{H}^b$), 1.27 – 1.19 (m, 1H, CHBpin), 1.21 (s, 12H, $C(CH_3)_2$), 1.14 (s, 12H, $C(CH_3)_2$), 1.05 (s, 9H, $SiC(CH_3)_3$), 1.02 (d, *J* = 6.0 Hz, 3H, CH_3), 0.79 (d, *J* = 7.6 Hz, 2H, CH_2Bpin) ppm

^{13}C NMR: (101 MHz, $CDCl_3$) δ 136.0 (ArH), 135.4 (ArC), 134.8 (ArC), 129.4 (ArH), 129.3 (ArH), 127.5 (ArH), 127.4 (ArH), 82.9 (*C*$(CH_3)_2$), 82.8 (*C*$(CH_3)_2$), 68.7 (CHO), 43.7 (CH_2), 27.3 (SiC(*C*$H_3)_3$), 25.0 (C(*C*$H_3)_2$), 24.9 (C(*C*$H_3)_2$), 24.9 (C(*C*$H_3)_2$), 24.8 (C(*C*$H_3)_2$), 23.6 (CH_3), 19.4 (Si*C*$(CH_3)_3$) ppm

HRMS (*m/z*): (ESI) calc'd for $C_{33}H_{52}B_2O_5SiNa$ $[M+Na]^+$: 601.3674, found: 601.3675

IR (neat) ν_{max}: 2976, 2930, 2857, 1370, 1312, 1139, 1106, 701 and 611 cm^{-1}

$[\alpha]_D^{24}$**:** −10.0 (*c* = 1.0, $CHCl_3$)

2,2'-((2*R*,4*S*)-4,8-Dimethylnon-7-ene-1,2-diyl)bis(4,4,5,5-tetramethyl-1,3,2-dioxaborolane) (59):

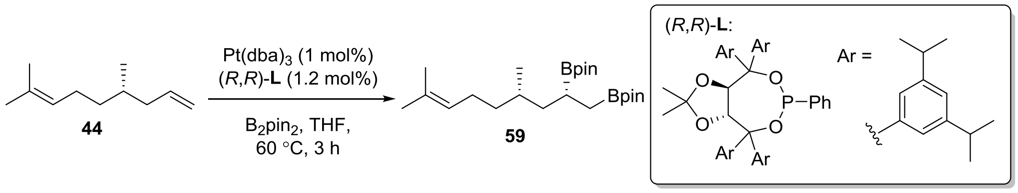

Pt(dba)$_3$ (59 mg, 0.07 mmol, 1.0 mol%), (*R*,*R*)-**L** (72 mg, 0.08 mmol, 1.2 mol%) and B$_2$pin$_2$ (1.75 g, 6.90 mmol, 1.05 eq.) were added to a flame-dried Schlenk-tube purged with N$_2$. THF (6.57 mL) was added before sealing the flask and heating at 80 °C (oil bath) for 30 mins. After cooling to ambient temperature **44** (1.0 g, 6.57 mmol, 1.00 eq.) was added before re-sealing and heating for 3 hr at 60 °C (oil bath). The solution was then cooled to ambient temperature and concentrated under reduced pressure. The crude residue was directly purified by flash column chromatography (SiO$_2$; 95:5 pentane:Et$_2$O) to yield **59** (2.25 g, >95:5 d.r., 84%) as a pale yellow viscous oil.

TLC: R_f = 0.31 (90:10 pentane:Et$_2$O)

^{1}H NMR: (400 MHz, CDCl$_3$) δ 5.09 (t, *J* = 7.0 Hz, 1H, =CH), 2.04 – 1.85 (m, 2H, =CHC*H*$_2$), 1.66 (s, 3H, =C(C*H*$_3$)^a(CH$_3$)^b), 1.58 (s, 3H, =C(CH$_3$)^a(C*H*$_3$)^b), 1.52 – 1.41 (m, 1H, CH), 1.38 – 1.04 (m, 5H, CHBpin, 2×CH$_2$), 1.22 (s, 12H, C(CH$_3$)$_2$), 1.22 (s, 12H, C(CH$_3$)$_2$), 0.82 (d, *J* = 6.6 Hz, 3H, CH$_3$), 0.79 (d, *J* = 7.4 Hz, 2H, CH$_2$Bpin) ppm

^{13}C NMR: (101 MHz, CDCl$_3$) δ 130.8 (=*C*(CH$_3$)$_2$), 125.4 (=CH), 82.9 (*C*(CH$_3$)$_2$), 82.8 (*C*(CH$_3$)$_2$), 40.9 (CH$_2$), 37.4 (CH$_2$), 31.1 (CH), 25.8 (=C(*C*H$_3$)^a(CH$_3$)^b), 25.7 (=CH*C*H$_2$), 25.0 (C(*C*H$_3$)$_2$), 25.0 (C(*C*H$_3$)$_2$), 24.9 (C(*C*H$_3$)$_2$), 19.6 (CH$_3$), 17.8 (=C(CH$_3$)^a(*C*H$_3$)^b) ppm

HRMS (*m/z*): (ESI) calc'd for C$_{23}$H$_{44}$B$_2$O$_4$Na [M+Na]$^+$: 429.3326, found: 429.3320

IR (neat) ν_{max}: 2977, 2914, 1370, 1310, 1140, 968 and 847 cm^{-1}

$[\alpha]_D^{24}$: +7.0 (*c* = 1.0, CHCl$_3$)

***tert*-Butyl(((2*R*,4*S*,5*S*)-2,4-dimethyl-5,6-bis(4,4,5,5-tetramethyl-1,3,2-dioxaborolan-2-yl)hexyl)oxy)diphenylsilane (60):**

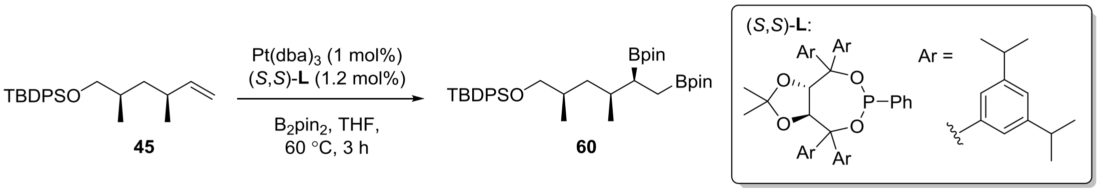

$Pt(dba)_3$ (12 mg, 0.01 mmol, 1.0 mol%), (*S*,*S*)-**L** (15 mg, 0.02 mmol, 1.2 mol%) and B_2pin_2 (364 g, 1.43 mmol, 1.05 eq.) were added to a flame-dried Schlenk-tube purged with N_2. THF (1.36 mL) was added before sealing the flask and heating at 80 °C (oil bath) for 30 mins. After cooling to ambient temperature **45** (500 mg, 1.36 mmol, 1.00 eq.) in THF (1.36 mL) was added before re-sealing and heating for 3 hr at 60 °C (oil bath). The solution was then cooled to ambient temperature and concentrated under reduced pressure. The crude residue was directly purified by flash column chromatography (SiO_2; 95:5 pentane:Et_2O) to yield **60** (637 mg, >95:5 d.r., 75%) as a pale yellow viscous oil.

TLC: R_f = 0.24 (90:10 pentane:EtOAc)

^{1}H NMR: (400 MHz, $CDCl_3$) δ 7.72 – 7.62 (m, 4H, ArH), 7.43 – 7.34 (m, 6H, ArH), 3.61 (dd, J = 9.8, 4.5 Hz, 1H, OCH^aH^b), 3.30 (dd, J = 9.8, 7.6 Hz, 1H, OCH^aH^b), 1.83 – 1.73 (m, 1H, OCH_2CH), 1.57 – 1.47 (m, 1H, CH), 1.38 – 1.28 (m, 1H, CH^aH^b), 1.21 (s, 12H, $C(CH_3)_2$), 1.19 (s, 6H, $C(CH_3)_2$), 1.18 (s, 6H, $C(CH_3)_2$), 1.15 – 1.08 (m, 1H, CH^aH^b), 1.05 (s, 9H, $SiC(CH_3)_3$), 0.96 (d, J = 6.6 Hz, 3H, OCH_2CHCH_3), 0.94 – 0.85 (m, 2H, CH^aH^bBpin and CHBpin), 0.83 (d, J = 6.7 Hz, 3H, CH_3), 0.68 (dd, J = 15.8, 5.1 Hz, 1H, CH^aH^bBpin) ppm

^{13}C NMR: (101 MHz, $CDCl_3$) δ 135.8 (ArH), 134.4 (ArC), 134.3 (ArC), 129.5 (ArH), 129.5 (ArH), 127.7 (ArH), 127.6 (ArH), 82.9 ($C(CH_3)_2$), 82.8 ($C(CH_3)_2$), 69.3 (CH_2O), 40.6 (CH_2), 34.2 (CH), 33.4 (CH), 27.1 ($SiC(CH_3)_3$), 25.1 ($C(CH_3)_2$), 25.1 ($C(CH_3)_2$), 25.0 ($C(CH_3)_2$), 24.9 ($C(CH_3)_2$), 19.4 ($SiC(CH_3)_3$), 18.7 (CH_3), 18.23 (CH_3) ppm

HRMS (*m/z*): (ESI) calc'd for $C_{36}H_{58}B_2O_5SiNa$ $[M+Na]^+$: 643.4145, found: 643.4158

IR (neat) ν_{max}: 2976, 2930, 2858, 1369, 1308, 1140, 1110 and 700 cm^{-1}

$[\alpha]_D^{24}$**:** –13.0 (c = 10.0, $CHCl_3$)

1-(But-3-en-1-yl)-5-(4-fluorophenyl)-2-isopropyl-*N*,4-diphenyl-1H-pyrrole-3-carboxamide (61):[22]

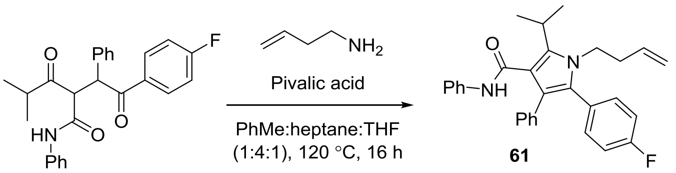

Pivalic acid (0.24 g, 2.40 mmol, 0.20 eq.) was added to a solution of 2-(2-(4-fluorophenyl)-2-oxo-1-phenylethyl)-4-methyl-3-oxo-*N*-phenylpentanamide (5.00 g, 12.00 mmol, 1.00 eq.) and but-3-en-1-amine (1.65 mL, 18.00 mmol, 1.5 eq.) in a mixture of PhMe (5.9 mL), heptane (23.5 mL) and THF (5.9 mL). The reaction mixture was stirred at 120 °C (oil bath) for 16 hr. The reaction mixture was cooled to ambient temperature and diluted with H_2O (50 mL) and Et_2O (50 mL). The phases were separated and the aqueous phase was extracted with Et_2O (3×100 mL) and the combined organic phases were washed with brine (50 mL), dried over $MgSO_4$, filtered and concentrated under reduced pressure. The crude residue was purified by flash column chromatography (SiO_2; pentane:Et_2O 5:1) to yield **61** (5.43 g, 62%) as a white solid.

TLC: R_f = 0.52 (80:20 pentane:Et_2O)

^{1}H NMR: (400 MHz, $CDCl_3$) δ 7.21 – 7.15 (m, 9H, ArH), 7.08 – 7.04 (m, 2H, ArH), 6.95 – 7.03 (m, 3H, ArH), 6.86 (br s, 1H, NH), 5.57 (ddt, J = 17.10, 10.30, 6.80 Hz, 1H, C*H*=CH_2), 5.02 – 4.92 (m, 2H, CH=CH_2), 3.89 (m, 2H, NCH_2), 3.57 (sept, J = 7.20 Hz, C*H*(CH_3)$_2$), 2.27 (m, 2H, NCH_2CH_2), 1.54 (d, J = 7.20 Hz, 6H, CH(CH_3)$_2$) ppm

^{13}C NMR: (101 MHz, $CDCl_3$) 164.8 (C=O), 162.4 (d, $J_{C\text{-}F}$ = 248.0 ArF), 141.5 (C), 138.5 (C), 134.7 (C), 133.7 (*C*H=CH_2), 133.3 (d, $J_{C\text{-}F}$ = 8.2 Hz, ArH and C), 130.6 (ArH), 129.0 (C), 128.8 (ArH), 128.5 (ArH), 128.4 (d, $J_{C\text{-}F}$ = 3.4 Hz, C), 126.7 (ArH), 123.6 (ArH), 121.9 (C), 119.6 (ArH), 117.4 (CH=*C*H_2), 115.5 (d, $J_{C\text{-}F}$ = 21.9 Hz, ArH), 44.1 (NCH_2), 35.8 (NCH_2*C*H_2), 26.3 (*C*H(CH_3)$_2$), 21.8 (CH(*C*H_3)$_2$) ppm

HRMS (*m*/*z*): (ESI) calc'd for $C_{30}H_{30}FN_2O$ $[M+H]^+$ 453.2337, found: 453.2333

IR (neat) ν_{max}: 3392, 2963, 1656 (C=O), 1529, 1218 and 755 cm^{-1}

m.p.: 143 °C – 141 °C (Et_2O)

[22] (a) K. L. Baumann, D. E. Butler, C. F. Deering, K. E. Mennen, A. Millar, T. N. Nanninga, C. W. Palmer and B. D. Roth, *Tetrahedron Lett.* **1992**, *33*, 2283. (b) L. C. Dias, A. S. Vieira and E. J. Barreiro, *Org. Biomol. Chem.* **2016**, *14*, 2291.

(*S*)-1-(3,4-Bis(4,4,5,5-tetramethyl-1,3,2-dioxaborolan-2-yl)butyl)-5-(4-fluorophenyl)-2-isopropyl-*N*,4-diphenyl-1H-pyrrole-3-carboxamide (62):

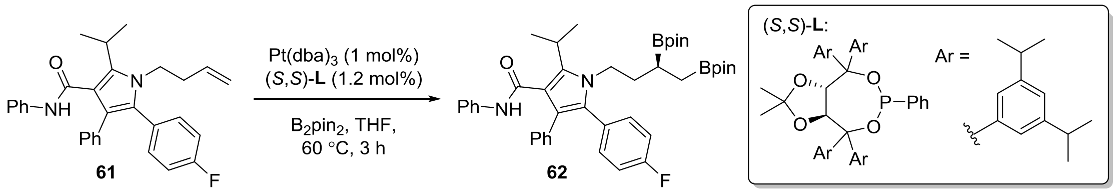

Pt(dba)$_3$ (17.8 mg, 0.02 mmol, 3.0 mol%), (*S*,*S*)-**L** (36.0 mg, 0.04 mmol, 6 mol%) and B$_2$pin$_2$ (335 mg, 1.32 mmol, 2.00 eq.) were added to a flame-dried Schlenk-tube purged with N$_2$. Anhydrous THF (0.66 mL) was added before sealing the flask and heating at 80 °C (oil bath) for 30 mins. After cooling to ambient temperature 1-(but-3-en-1-yl)-5-(4-fluorophenyl)-2-isopropyl-*N*,4-diphenyl-1H-pyrrole-3-carboxamide (**61**) (300 mg, 0.66 mmol, 1.00 eq.) was added before re-sealing and heating for 16 hr at 60 °C (oil bath). The solution was then cooled to ambient temperature and concentrated under reduced pressure. The crude residue was directly purified by flash column chromatography (SiO$_2$; pentane:Et$_2$O 5:1) to yield **62** (287 mg, 62%) as a white foam.

TLC: R_f = 0.24 (80:20 pentane:Et$_2$O)

^{1}H NMR: (400 MHz, CDCl$_3$) δ 7.21 – 7.11 (m, 9H, ArH), 7.08 – 7.03 (m, 2H, ArH), 7.00 – 6.94 (m, 3H, ArH), 6.85 (br s, 1H, NH), 3.87 (dt, *J* = 13.0, 4.9 Hz, 1H, NCH^aCH^b), 3.73 (dt, *J* = 13.0, 4.9 Hz, 1H, NCH^{a}CH^b), 3.58 – 3.44 (m, 1H, CH(CH$_3$)$_2$), 1.84 – 1.71 (m, 1H, NCH$_2$CH^aH^b), 1.69 – 1.59 (m, 1H, NCH$_2$CH^aH^b), 1.54 (d, *J* = 7.1 Hz, 6H, CH(CH$_3$)$_2$), 1.22 (s, 12H, 2×C(CH$_3$)$_2$), 1.18 (s, 12H, 2×C(CH$_3$)$_2$), 1.06 – 0.98 (m, 1H, CHBpin), 0.81 (dd, *J* = 15.9, 9.1 Hz, 1H, CH^aCH^{b}Bpin), 0.69 (dd, *J* = 15.9, 5.8 Hz, 1H, CH^{a}CH^bBpin) ppm

^{13}C NMR: (101 MHz, CDCl$_3$) δ 165.0 (C=O), 161.1 (d, J_{C-F} = 246.7 Hz, ArF), 141.5 (C), 138.6 (C), 135.0 (C), 133.4 (d, J_{C-F} = 8.9 Hz, ArH and C), 130.6 (ArH), 128.7 (C), 128.5 (ArH), 128.3 (ArH), 126.5 (ArH), 123.5 (ArH), 121.6 (C), 119.6 (ArH), 115.3 (d, J_{C-F} = 21.4 Hz, ArH), 83.2 (d, J_{C-F} = 9.3 Hz, C), 44.4 (NCH$_2$), 36.1 (NCH$_2$*C*H$_2$), 26.2 (Ar*C*(CH$_3$)$_2$), 25.0 (pinacol), 24.8 (pinacol), 21.7 (ArC(*C*H$_3$)$_2$) ppm

HRMS (*m/z*): (ESI) calc'd for $C_{42}H_{53}B_2FN_2NaO_5$ [M+Na]$^+$ 729.4031, found: 729.4040

IR (neat) ν_{max}: 3411, 2976, 1669 (C=O), 1509, 1370, 1311, 1220, 1140, 844 and 752 cm^{-1}

$[\alpha]_D^{24}$: −7.0 (c = 1.0, $CHCl_3$)

3-((*tert*-Butyldimethylsilyl)oxy)propyl 2,4,6-triisopropylbenzoate (63):

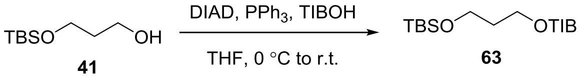

Diisopropyl azodicarboxylate (0.1 mL, 0.52 mmol, 1.1 eq.) was added dropwise to a solution of 3-((*tert*-butyldimethylsilyl)oxy)propan-1-ol (**41**) (89.5 mg, 0.47 mmol, 1 eq.), triphenyl phosphine (136.4 mg, 0.52 mmol, 1.1 eq.) and 2,4,6-triisopropylbenzoic acid (134.1 mg, 0.54 mmol, 1.15 eq.) in anhydrous THF (0.71 mL) at 0 °C (ice/water). The resulting solution was warmed to ambient temperature and was then stirred at this temperature for 4 hr. The reaction mixture was then concentrated under reduced pressure. The crude residue was purified directly by flash column chromatography (SiO_2; pentane:Et_2O 100:1) to yield **63** (135 mg, 68%) as a colourless oil.

TLC: R_f = 0.62 (97:3 pentane:Et_2O)

^{1}H NMR: (400 MHz, $CDCl_3$) δ 7.00 (s, 2H, ArH), 4.40 (t, J = 6.4 Hz, 2H, $TIBOCH_2$), 3.72 (t, J = 6.1, $SiOCH_2$), 2.89 (sept, J = 6.9 Hz, 1H, *para*-C*H*(CH_3)$_2$), 2.85 (sept, J = 6.9 Hz, 2H, 2x*ortho*-C*H*(CH_3)$_2$), 1.93 (app p, J = 6.4 Hz, 2H, OCH_2CH_2), 1.24 (d, J = 6.9 Hz, 18H, 3xCH(C*H*$_3$)$_2$), 0.90 (s, 9H, $SiC(CH_3)_3$), 0.05 (s, 6H, $Si(CH_3)_2$) ppm

^{13}C NMR (101 MHz, $CDCl_3$) δ 171.0 (C=O), 150.1 (ArC), 144.8 (ArC), 130.7 (ArC), 120.9 (ArH), 62.1 ($TIBOCH_2$), 59.6 (SiOC), 34.5 (Ar*C*H(CH_3)$_2$), 32.0 (OCH_2CH_2), 31.6 (2xAr*C*H(CH_3)$_2$), 26.0 (SiC(*C*H$_3$)$_3$), 24.2 (ArC(*C*H$_3$)$_2$), 24.0 (ArC(*C*H$_3$)$_2$), 18.3 (SiC), −5.3 ($Si(CH_3)_2$) ppm

HRMS (*m/z*): (ESI) calc'd for $C_{25}H_{44}NaO_3Si$ $[M+H]^+$ 443.2952, found: 443.2940

IR (neat) ν_{max}: 2958, 2869, 1726 (C=O), 1462, 1250, 1073 and 836 cm^{-1}

3.1 Carbenoid Scope

(3*R*,5*R*)-2-Methylundecane-3,5-diol (4):

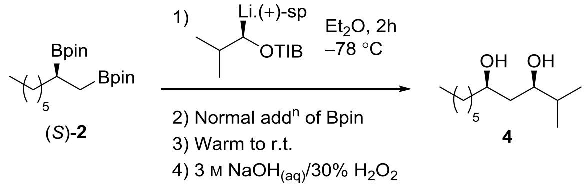

Isobutyl 2,4,6-triisopropylbenzoate (**32**) (175 mg, 0.57 mmol, 1.05 eq.), (+)-sparteine (0.13 mL, 0.57 mmol, 1.05 eq.) and anhydrous Et_2O (2.85 mL) were added to a flame-dried Schlenk-tube purged with N_2. The solution was cooled to −78 °C (dry ice/acetone) before adding *sec*-BuLi (0.42 mL, 1.30 M, 0.55 mmol, 1.00 eq.) dropwise over 5 min and leaving to react for 3 hr at this temperature. (*S*)-**2** (240 mg, 0.66 mmol, 1.20 eq.) was dissolved in anhydrous Et_2O (0.66 mL) and added dropwise to the reaction mixture over 1 min before leaving to react for a further 1 hr at the same temperature. After warming the solution to ambient temperature THF (2.85 mL) and one crystal of BHT was added, and then it cooled to 0 °C (ice/water). A 2:1 v:v mixture of 3 M aqueous NaOH (4 mL) and 30% aqueous H_2O_2 (2 mL) was prepared at 0 °C (ice/water) and degassed by gently bubbling N_2 through the solution. This aqueous solution was added dropwise to the vigorously stirred reaction mixture, which was subsequently warmed to ambient temperature and allowed to react for 1 hr. 2 M aqueous HCl (10 mL) was carefully added and the reaction mixture was extracted with EtOAc (3×20 mL). The combined organic fractions were dried over Na_2SO_4, filtered and concentrated under reduced pressure. The crude residue was purified by flash column chromatography (SiO_2; 90:10 pentane:EtOAc) to yield **4** (86 mg, 78%) as a viscous colorless oil.

TLC: R_f = 0.18 (80:20 pentane:EtOAc)

^{1}H NMR: (400 MHz, $CDCl_3$) δ 3.86 – 3.78 (m, 1H, CHO), 3.66 – 3.58 (m, 1H, CHO), 3.15 (br s, 2H, OH), 1.71 – 1.17 (m, 13H, 6×CH_2 and C*H*(CH_3)$_2$), 0.91 (d, *J* = 6.9 Hz, 6H, CH(CH_3)$_2$), 0.88 (t, *J* = 6.9 Hz, 3H, CH_3) ppm

^{13}C NMR: (101 MHz, $CDCl_3$) δ 78.2 (CHO), 73.5 (CHO), 39.5 (CH_2), 38.5 (CH_2), 34.4 (*C*H(CH_3)$_2$), 32.0 (CH_2), 29.5 (CH_2), 25.5 (CH_2), 22.7 (CH_2), 18.4 (CH(*C*H_3)$_2$), 17.6 (CH(*C*H_3)$_2$), 14.2 (CH_3) ppm

HRMS (*m*/*z*): (ESI) calc'd for $C_{12}H_{26}O_2Na$ $[M+Na]^+$: 225.1825, found: 225.1823

IR (neat) ν_{max}: 3346 (br), 2956, 2927, 2857, 1465, 1064 and 846 cm^{-1}

$[\alpha]_D^{24}$**:** +4.0 (c = 1.0, $CHCl_3$)

(5*S*,7*R*)-Tridec-1-ene-5,7-diol (5):

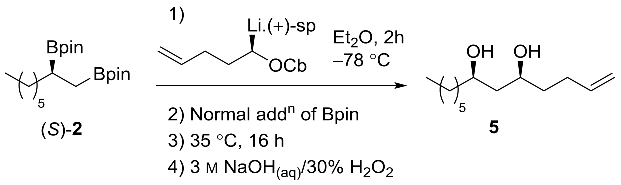

Pent-4-en-1-yl diisopropylcarbamate (**42**) (122 mg, 0.57 mmol, 1.05 eq.), (+)-sparteine (0.13 mL, 0.57 mmol, 1.05 eq.) and anhydrous Et_2O (2.85 mL) were added to a flame-dried Schlenk-tube purged with N_2. The solution was cooled to −78 °C (dry ice/acetone) before adding *sec*-BuLi (0.42 mL, 1.30 M, 0.55 mmol, 1.00 eq.) dropwise over 5 min and leaving to react for 5 hr at this temperature. (*S*)-**2** (240 mg, 0.66 mmol, 1.20 eq.) was dissolved in anhydrous Et_2O (0.66 mL) and added dropwise to the reaction mixture over 1 min before leaving to react for a further 1 hr at the same temperature. After warming to ambient temperature the Schlenk-tube was sealed and heated at 35 °C (oil bath) for 16 hr. The flask was allowed to cool to ambient temperature before adding THF (2.85 mL) and one crystal of BHT, and was then cooled to 0 °C (ice/water). A 2:1 v:v mixture of 3 M aqueous NaOH (4 mL) and 30% aqueous H_2O_2 (2 mL) was prepared at 0 °C (ice/water) and degassed by gently bubbling N_2 through the solution. This aqueous solution was added dropwise to the vigorously stirred reaction mixture, which was subsequently warmed to ambient temperature and allowed to react for 1 hr. 2 M aqueous HCl (10 mL) was carefully added and the reaction mixture was extracted with EtOAc (3×20 mL). The combined organic fractions were dried over Na_2SO_4, filtered and concentrated under reduced pressure. The crude residue was purified by flash column chromatography (SiO_2; 85:15 pentane:EtOAc) to yield **5** (81 mg, 69%) as a viscous colorless oil.

TLC: R_f = 0.37 (60:40 petroleum ether 40/40:EtOAc)

^{1}H NMR: (500 MHz, $CDCl_3$) δ 5.84 (ddt, J = 16.9, 10.2, 6.7 Hz, 1H, =CH), 5.05 (app dq, J = 17.1, 1.8 Hz, 1H, =CH^aH^b), 4.98 (ddt, J = 10.2, 1.8, 1.2 Hz, 1H, =CH^aH^b), 3.93 – 3.81 (m, 2H, 2×CHO), 2.85 (br s, 2H, 2×OH), 2.25 – 2.08 (m, 2H, CH_2CH=), 1.63 – 1.22 (m, 14H, 7×CH_2), 0.88 (t, J = 6.7 Hz, CH_3) ppm

^{13}C NMR: (126 MHz, $CDCl_3$) δ 138.6 (CH=), 115.0 (=CH_2), 73.4 (CHO), 72.8 (CHO), 43.0 (CH_2), 38.4 (CH_2), 37.3 (CH_2), 32.0 (CH_2), 29.9 (CH_2), 29.4 (CH_2CH=), 25.4 (CH_2), 22.7 (CH_2), 14.2 (CH_3) ppm

HRMS (*m/z*): (ESI) calc'd for $C_{13}H_{26}O_2Na$ $[M+Na]^+$: 237.1825, found: 237.1823

IR (neat) ν_{max}: 3366 (br), 2927, 2857, 1641, 1405, 1321, 909 and 665 cm^{-1}

$[\alpha]_D^{24}$: –3.0 (*c* = 1.0, $CHCl_3$)

(3*S*,5*R*)-1-((*tert*-Butyldimethylsilyl)oxy)undecane-3,5-diol (6):

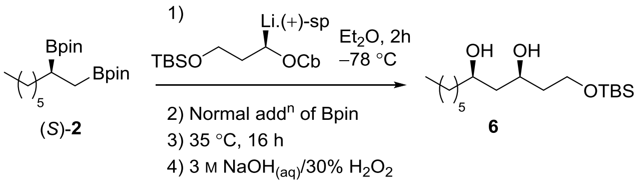

3-((tert-Butyldimethylsilyl)oxy)propyl diisopropylcarbamate (**52**) (182 mg, 0.57 mmol, 1.05 eq.), (+)-sparteine (0.13 mL, 0.57 mmol, 1.05 eq.) and anhydrous Et_2O (2.85 mL) were added to a flame-dried Schlenk-tube purged with N_2. The solution was cooled to –78 °C (dry ice/acetone) before adding *sec*-BuLi (0.42 mL, 1.30 M, 0.55 mmol, 1.00 eq.) dropwise over 5 min and leaving to react for 5 hr at this temperature. (*S*)-**2** (240 mg, 0.66 mmol, 1.20 eq.) was dissolved in anhydrous Et_2O (0.66 mL) and added dropwise to the reaction mixture over 1 min before leaving to react for a further 1 hr at the same temperature. After warming to ambient temperature the Schlenk-tube was sealed and heated at 35 °C (oil bath) for 16 hr. The flask was allowed to cool to ambient temperature before adding THF (2.85 mL) and one crystal of BHT, and was then cooled to 0 °C (ice/water). A 2:1 v:v mixture of 3 M aqueous NaOH (4 mL) and 30% aqueous H_2O_2 (2 mL) was prepared at 0 °C (ice/water) and degassed by gently bubbling N_2 through the solution. This aqueous solution was added dropwise to the vigorously stirred reaction mixture, which was subsequently warmed to ambient temperature and allowed to react for 1 hr. 2 M aqueous HCl (10 mL) was carefully added and the reaction mixture was extracted with EtOAc (3×20 mL). The combined organic fractions were dried over Na_2SO_4, filtered and concentrated under reduced pressure. The crude residue was purified by flash column chromatography (SiO_2; 85:15 pentane:EtOAc) to yield **6** (101 mg, 58%) as a viscous colorless oil.

TLC: R_f = 0.26 (80:20 pentane:EtOAc)

^{1}**H NMR:** (400 MHz, $CDCl_3$) δ 4.09 (app tt, J = 8.3, 3.5 Hz, 1H, CHO), 4.03 (br s, 1H, OH), 3.94 – 3.78 (m, 3H, CHO and CH_2OSi), 3.67 (br s, 1H OH), 1.77 – 1.20 (m, 14H, 7×CH_2), 0.90 (s, 9H, $SiC(CH_3)_3$), 0.88 (t, J = 7.0 Hz, 3H, CH_3), 0.08 (s, 6H, $Si(CH_3)_2$) ppm

^{13}**C NMR:** (101 MHz, $CDCl_3$) δ 73.7 (CHO), 72.6 (CHO), 62.7 (CH_2OSi), 43.4 (CH_2), 38.9 (CH_2), 38.1 (CH_2), 32.0 (CH_2), 29.5 (CH_2), 26.0 ($SiC(\mathit{C}H_3)_3$), 25.5 (CH_2), 22.8 (CH_2), 18.3 ($Si\mathit{C}(CH_3)_3$), 14.2 (CH_3), –5.4 ($Si(CH_3)_2$), –5.4 ($Si(CH_3)_2$) ppm

HRMS (*m/z*): (ESI) calc'd for $C_{17}H_{38}O_3SiNa$ $[M+Na]^+$: 341.2482, found: 341.2488

IR (neat) ν_{max}: 3351 (br), 2928, 2857, 1254, 1090, 834 and 775 cm^{-1}

$[\alpha]_D^{24}$**:** +10.0 (c = 1.0, $CHCl_3$)

(2*S*,4*R*,5*R*,7*R*)-1-(Methoxymethoxy)-2,4-dimethyltridecane-5,7-diol (7):

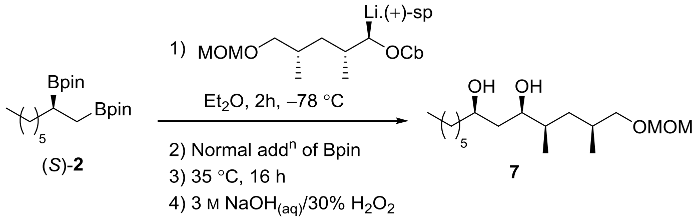

(2*R*,4*S*)-5-(Methoxymethoxy)-2,4-dimethylpentyl diisopropylcarbamate (**46**) (207 mg, 0.68 mmol, 1.25 eq.), (+)-sparteine (0.16 mL, 0.68 mmol, 1.25 eq.) and anhydrous Et_2O (3.41 mL) were added to a flame-dried Schlenk-tube purged with N_2. The solution was cooled to −78 °C (dry ice/acetone) before adding *sec*-BuLi (0.50 mL, 1.30 M, 0.66 mmol, 1.20 eq.) dropwise over 5 min and leaving to react for 5 hr at this temperature. (*S*)-**2** (242 mg, 0.55 mmol, 1.00 eq.) was dissolved in anhydrous Et_2O (0.55 mL) and added dropwise to the reaction mixture over 1 min before leaving to react for a further 1 hr at the same temperature. After warming to ambient temperature the Schlenk-tube was sealed and heated at 35 °C (oil bath) for 16 hr. The flask was allowed to cool to ambient temperature before adding THF (2.85 mL) and one crystal of BHT, and was then cooled to 0 °C (ice/water). A 2:1 v:v mixture of 3 M aqueous NaOH (4 mL) and 30% aqueous H_2O_2 (2 mL) was prepared at 0 °C (ice/water) and degassed by gently bubbling N_2 through the solution. This aqueous solution was added dropwise to the vigorously stirred reaction mixture, which was subsequently warmed to ambient temperature and allowed to react for 1 hr. Water (10 mL) was added and the reaction mixture was extracted with EtOAc (3×20 mL). The combined organic fractions were dried over Na_2SO_4, filtered and concentrated

under reduced pressure. The crude residue was purified by flash column chromatography (SiO_2; 70:30 pentane:EtOAc) to yield **7** (72 mg, 43%) as a viscous colorless oil.

TLC: R_f = 0.23 (70:30 pentane:EtOAc)

^{1}H NMR: (500 MHz, $CDCl_3$) δ 4.61 (s, 2H, OCH_2O), 3.86 – 3.76 (m, 2H, 2×CHO), 3.38 (dd, J = 9.4, 5.9 Hz, 1H, $C\mathit{H}^aH^bO$), 3.35 (s, 3H, OCH_3), 3.32 (dd, J = 9.4, 6.3 Hz, 1H, $CH^a\mathit{H}^bO$), 3.21 (br s, 1H, OH), 3.01 (br s, 1H, OH), 1.89 – 1.78 (m, 1H, C*H*CH_2O), 1.64 – 1.22 (m, 13H, 6×CH_2 and $C\mathit{H}^aH^b$), 1.04 – 0.93 (m, 1H, $CH^a\mathit{H}^b$), 0.95 (d, J = 6.7 Hz, 3H, $CHC\mathit{H}_3$), 0.89 (d, J = 6.7 Hz, 3H, $CHC\mathit{H}_3$), 0.88 (t, J = 7.2 Hz, 3H, CH_3) ppm

^{13}C NMR: (126 MHz, $CDCl_3$) δ 96.8 (OCH_2O), 75.6 (CHO), 73.5 (CHO), 73.3 (CH_2O), 55.3 (OCH_3), 40.0 (CH_2), 38.4 (CH_2), 37.2 (CH_2), 36.6 (*C*HCH_3), 32.0 (CH_2), 30.9 (*C*HCH_3), 29.5 (CH_2), 25.5 (CH_2), 22.8 (CH_2), 18.4 ($CH\mathit{C}H_3$), 14.7 ($CH\mathit{C}H_3$), 14.2 (CH_3) ppm

HRMS (*m/z*): (ESI) calc'd for $C_{17}H_{36}O_4Na$ $[M+Na]^+$: 327.2506, found: 327.2514

IR (neat) ν_{max}: 3416, 2926, 2857, 1405, 1109, 1042, 919, 733 and 667 cm^{-1}

$[\alpha]_D^{24}$**:** +6.0 (c = 1.0, $CHCl_3$)

(4*R*,6*R*,*E*)-4-Methyldodec-2-ene-4,6-diol (8):

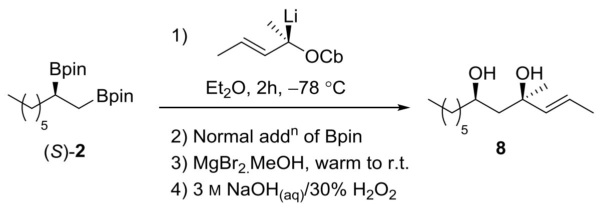

(*S*,*E*)-Pent-3-en-2-yl diisopropylcarbamate (**47**) (117 mg, 0.55 mmol, 1.00 eq.) and anhydrous Et_2O (3.30 mL) were added to a flame-dried Schlenk-tube purged with N_2. The solution was cooled to –78 °C (dry ice/acetone) before adding *sec*-BuLi (0.46 mL, 1.30 M, 0.60 mmol, 1.10 eq.) dropwise over 5 min and leaving to react for 15 min at this temperature. (*S*)-**2** (241 mg, 0.66 mmol, 1.20 eq.) was dissolved in anhydrous Et_2O (0.66 mL) and added dropwise to the reaction mixture over 1 min before leaving to react for a further 1 hr at the same temperature. A 1 M solution of $MgBr_2$ in MeOH (0.83 mL, 0.83 mmol, 1.50 eq.) was added dropwise over 2 min and the mixture was allowed to react for a further 2 min before warming to ambient temperature. THF (2.85 mL) and one crystal of BHT were added, and the mixture was subsequently cooled to 0 °C (ice/water). A 2:1 v:v mixture of 3 M aqueous NaOH (4 mL) and

30% aqueous H_2O_2 (2 mL) was prepared at 0 °C (ice/water) and degassed by gently bubbling N_2 through the solution. This aqueous solution was added dropwise to the vigorously stirred reaction mixture, which was subsequently warmed to ambient temperature and allowed to react for 1 hr. 2 M aqueous HCl (10 mL) was carefully added and the reaction mixture was extracted with EtOAc (3×20 mL). The combined organic fractions were dried over Na_2SO_4, filtered and concentrated under reduced pressure. The crude residue was purified by flash column chromatography (SiO_2; 80:20 pentane:EtOAc) to yield **8** (78 mg, 66%) as a viscous colorless oil.

TLC: R_f = 0.25 (80:20 pentane:EtOAc)

^{1}H NMR: (500 MHz, $CDCl_3$) δ 5.74 – 5.46 (m, 2H, 2×=CH), 4.19 – 3.92 (m, 1H, CHO), 3.18 (br s, 1H, OH), 2.54 (br s, 1H, OH), 1.82 – 1.18 (m, 12H, 6×CH_2), 1.69 (dd, *J* = 6.2, 1.3 Hz, 3H, =CHCH_3), 1.35 (s, 3H, CCH_3), 0.88 (t, *J* = 6.9 Hz, 3H, CH_3) ppm

^{13}C NMR: (126 MHz, $CDCl_3$) δ 139.4 (=CH), 122.5 (=CH), 73.4 (CO), 69.4 (CHO), 47.4 (CH_2), 38.4 (CH_2), 32.0 (CH_2), 29.5 (CH_2), 26.9 (C*C*H_3), 25.6 (CH_2), 22.8 (CH_2), 17.8 (=CH*C*H_3), 14.2 (CH_3) ppm

HRMS (*m/z*): (ESI) calc'd for $C_{13}H_{26}O_2Na$ $[M+Na]^+$: 237.1825, found: 237.1830

IR (neat) ν_{max}: 3368 (br), 2928, 2857, 1409, 1473, 1311, 1132, 966, 849 and 665 cm^{-1}

$[\alpha]_D^{24}$**:** –5.0 (*c* = 1.0, $CHCl_3$)

(3*R*,5*R*)-3-Methyl-1-phenylundecane-3,5-diol (9):

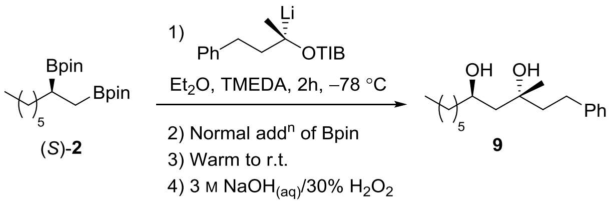

(*S*)-4-Phenyl-2-(trimethylstannyl)butan-2-yl 2,4,6-triisopropylbenzoate (**48**) (300 mg, 0.55 mmol, 1.00 eq.) TMEDA (0.09 mL, 0.61 mmol, 1.10 eq.) and anhydrous Et_2O (2.76 mL) were added to a flame-dried Schlenk-tube purged with N_2. The solution was cooled to −78 °C (dry ice/acetone) before adding *n*-BuLi (0.38 mL, 1.60 M, 0.61 mmol, 1.10 eq.) dropwise over 10 min and leaving to react for 2 hr at this temperature. (*S*)-**2** (243 mg, 0.66 mmol, 1.20 eq.) was dissolved in anhydrous Et_2O (0.66 mL) and added dropwise to the reaction mixture over 1 min

before leaving to react for a further 1 hr at the same temperature. After warming the solution to ambient temperature THF (2.85 mL) and one crystal of BHT was added, and then it cooled to 0 °C (ice/water). A 2:1 v:v mixture of 3 M aqueous NaOH (4 mL) and 30% aqueous H_2O_2 (2 mL) was prepared at 0 °C (ice/water) and degassed by gently bubbling N_2 through the solution. This aqueous solution was added dropwise to the vigorously stirred reaction mixture, which was subsequently warmed to ambient temperature and allowed to react for 1 hr. 2 M aqueous HCl (10 mL) was carefully added and the reaction mixture was extracted with EtOAc (3×20 mL). The combined organic fractions were dried over Na_2SO_4, filtered and concentrated under reduced pressure. The crude residue was purified by flash column chromatography (SiO_2; 80:20 pentane:EtOAc) to yield **9** (123 mg, 80%) as a viscous colorless oil.

TLC: R_f = 0.19 (80:20 pentane:EtOAc)

^{1}H NMR: (500 MHz, $CDCl_3$) δ 7.33 – 7.24 (m, 2H, ArH), 7.24 – 7.15 (m, 3H, ArH), 4.09 – 3.91 (m, 1H CHO), 3.18 (br s, 2H, OH), 2.72 (ddd, J = 13.4, 12.1, 5.0 Hz, 1H, ArCH^aH^b), 2.60 (ddd, J = 13.4, 12.0, 5.3 Hz, ArCH^aH^b), 1.96 (ddd, J = 13.6, 12.0, 5.0 Hz, 1H, $ArCH_2CH^aH^b$), 1.87 (ddd, J = 13.6, 12.1, 5.4 Hz, 1H, $ArCH_2CH^aH^b$), 1.73 – 1.21 (m, 15H, 6×CH_2 and CCH_3), 0.88 (t, J = 7.0 Hz, 3H, CH_3) ppm

^{13}C NMR: (126 MHz, $CDCl_3$) δ 142.5 (ArC), 128.6 (ArH), 128.4 (ArH), 126.0 (ArH), 73.5 (CO), 69.6 (CHO), 46.7 (CH_2), 42.5 ($ArCH_2CH_2$), 38.7 (CH_2), 31.9 (CH_2), 31.1 ($ArCH_2$), 29.4 (CH_2), 28.8 (CCH_3), 25.5 (CH_2), 22.7 (CH_2), 14.2 (CH_3) ppm

HRMS (*m/z*): (ESI) calc'd for $C_{18}H_{30}O_2Na$ $[M+Na]^+$: 301.2138, found: 301.2142

IR (neat) ν_{max}: 3348 (br), 2928, 2857, 1700, 1605, 1130, 741 and 697 cm^{-1}

$[\alpha]_D^{24}$: +7.0 (c = 1.0, $CHCl_3$)

(2*R*,4*R*)-2-(4-Methoxyphenyl)decane-2,4-diol (10):

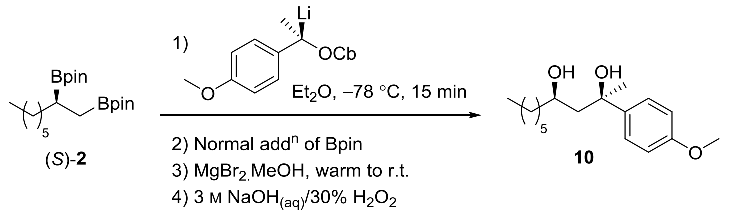

(*S*)-1-(4-Methoxyphenyl)ethyl diisopropylcarbamate (**49**) (155 mg, 0.55 mmol, 1.00 eq.), TMEDA (0.09 mL, 0.61 mmol, 1.10 eq.) and anhydrous Et_2O (2.70 mL) were added to a flame-dried Schlenk-tube purged with N_2. The solution was cooled to –78 °C (dry ice/acetone) before adding *sec*-BuLi (0.48 mL, 1.30 M, 0.62 mmol, 1.12 eq.) dropwise over 5 min and leaving to react for 15 min at this temperature. (*S*)-**2** (244 mg, 0.66 mmol, 1.20 eq.) was dissolved in anhydrous Et_2O (0.66 mL) and added dropwise to the reaction mixture over 1 min before leaving to react for a further 1 hr at the same temperature. A 1 M solution of $MgBr_2$ in MeOH (0.61 mL, 0.61 mmol, 1.10 eq.) was added dropwise over 2 min and the mixture was allowed to react for a further 2 min before warming to ambient temperature. Water (10 mL) was added and the organic phase was collected, followed by extraction of the aqueous phase (3×15 mL Et_2O). The combined organic phases were dried over $MgSO_4$, filtered and concentrated under reduced pressure. The crude residue was dissolved in THF (6 mL) and one crystal of BHT was added, then the mixture was subsequently cooled to 0 °C (ice/water). A 2:1 v:v mixture of 3 M aqueous NaOH (4 mL) and 30% aqueous H_2O_2 (2 mL) was prepared at 0 °C (ice/water) and degassed by gently bubbling N_2 through the solution. This aqueous solution was added dropwise to the vigorously stirred reaction mixture, which was subsequently warmed to ambient temperature and allowed to react for 1 hr. 2 M aqueous HCl (10 mL) was carefully added and the reaction mixture was extracted with EtOAc (3×20 mL). The combined organic fractions were dried over Na_2SO_4, filtered and concentrated under reduced pressure. The crude residue was purified by flash column chromatography (SiO_2; 80:20 pentane:EtOAc) to yield **10** (150 mg, 94%) as a viscous colorless oil.

TLC: R_f = 0.21 (80:20 pentane:EtOAc)

^{1}H NMR: (400 MHz, $CDCl_3$) δ 7.38 (d, J = 8.8 Hz, 2H, ArH), 6.87 (d, J = 8.8 Hz, 2H, ArH), 4.12 – 4.03 (m, 1H, CHO), 3.80 (s, 3H, OCH_3), 3.30 (br s, 2, OH), 1.83 (dd, J = 14.7, 9.7 Hz, 1H, CHOCH^aH^bC), 1.77 (dd, J = 14.7, 2.7 Hz, 1H, CHOC$H^a$$H^b$C), 1.64 (s, 3H, CCH_3), 1.53 – 1.09 (m, 10H, 5×CH_2), 0.88 (t, J = 6.6 Hz, 3H, CH_3) ppm

^{13}C NMR: (101 MHz, $CDCl_3$) δ 158.5 (ArC), 141.7 (ArC), 125.7 (ArH), 113.7 (ArH), 74.9 (CO), 69.9 (CHO), 55.4 (OCH_3), 49.6 (CHO*C*H_2C), 38.4 (CH_2), 31.9 (CH_2), 29.4 (CH_2), 28.1 (CCH_3), 25.5 (CH_2), 22.7 (CH_2), 14.2 (CH_3) ppm

HRMS (*m/z*): (ESI) calc'd for $C_{17}H_{28}O_3Na$ $[M+Na]^+$: 303.1931, found: 303.1928

IR (neat) ν_{max}: 3346 (br), 2927, 2856, 1611, 1511, 1463, 1301, 1247, 1177, 1092, 1034 and 829 cm^{-1}

$[\alpha]_D^{24}$**:** +21.0 (c = 1.0, $CHCl_3$)

(2*R*,4*R*)-2-Phenyldecane-2,4-diol (11):

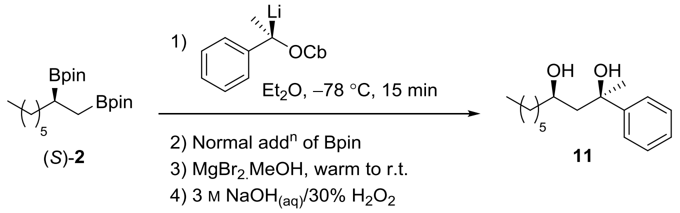

(*S*)-1-Phenylethyl diisopropylcarbamate (**50**) (138 mg, 0.55 mmol, 1.00 eq.) and anhydrous Et_2O (2.77 mL) were added to a flame-dried Schlenk-tube purged with N_2. The solution was cooled to –78 °C (dry ice/acetone) before adding *sec*-BuLi (0.48 mL, 1.30 M, 0.62 mmol, 1.12 eq.) dropwise over 5 min and leaving to react for 15 min at this temperature. (*S*)-**2** (243 mg, 0.66 mmol, 1.20 eq.) was dissolved in anhydrous Et_2O (0.66 mL) and added dropwise to the reaction mixture over 1 min before leaving to react for a further 1 hr at the same temperature. A 1 M solution of $MgBr_2$ in MeOH (0.83 mL, 0.83 mmol, 1.50 eq.) was added dropwise over 2 min and the mixture was allowed to react for a further 2 min before warming to ambient temperature. Water (10 mL) was added and the organic phase was collected, followed by extraction of the aqueous phase (3×15 mL Et_2O). The combined organic phases were dried over $MgSO_4$, filtered and concentrated under reduced pressure. The crude residue was dissolved in THF (6 mL) and one crystal of BHT was added, then the mixture was subsequently cooled to 0 °C (ice/water). A 2:1 v:v mixture of 3 M aqueous NaOH (4 mL) and 30% aqueous H_2O_2 (2 mL) was prepared at 0 °C (ice/water) and degassed by gently bubbling N_2 through the solution. This aqueous solution was added dropwise to the vigorously stirred reaction mixture, which was subsequently warmed to ambient temperature and allowed to react for 1 hr. 2 M aqueous HCl (10 mL) was carefully added and the reaction mixture was extracted with EtOAc

(3×20 mL). The combined organic fractions were dried over Na_2SO_4, filtered and concentrated under reduced pressure. The crude residue was purified by flash column chromatography (SiO_2; 80:20 pentane:EtOAc) to yield **11** (101 mg, 73%) as a viscous colorless oil.

TLC: R_f = 0.24 (80:20 pentane:EtOAc)

^{1}H NMR: (500 MHz, $CDCl_3$) δ 7.47 (d, J = 7.3 Hz, 2H, ArH), 7.35 (t, J = 7.7 Hz, 2H, ArH), 7.25 (t, J = 7.3 Hz, 1H, ArH), 4.12 (app tt, J = 8.5, 4.1 Hz, 1H, CHO), 2.62 (br s, 2H, OH), 1.89 – 1.77 (m, 2H, CHOCH_2C), 1.67 (s, 3H, CCH_3), 1.62 – 1.14 (m, 10H, 5×CH_2), 0.88 (6.7 Hz, 3H, CH_3) ppm

^{13}C NMR: (126 MHz, $CDCl_3$) δ 149.4 (ArC), 128.4 (ArH), 126.9 (ArH), 124.5 (ArH), 75.1 (CO), 69.9 (CHO), 49.5 (CHOCH_2C), 38.5 (CH_2), 31.9 (CH_2), 29.4 (CH_2), 28.2 (CCH_3), 25.5 (CH_2), 22.7 (CH_2), 14.2 (CH_3) ppm

HRMS (*m/z*): (ESI) calc'd for $C_{16}H_{26}O_2Na$ $[M+Na]^+$: 273.1825, found: 273.1831

IR (neat) ν_{max}: 3351, 2927, 2856, 1375, 1412, 1446, 1071, 762 and 698 cm^{-1}

$[\alpha]_D^{24}$**:** +22.0 (c = 1.0, $CHCl_3$)

(2*R*,4*R*)-2-(4-Fluorophenyl)decane-2,4-diol (12):

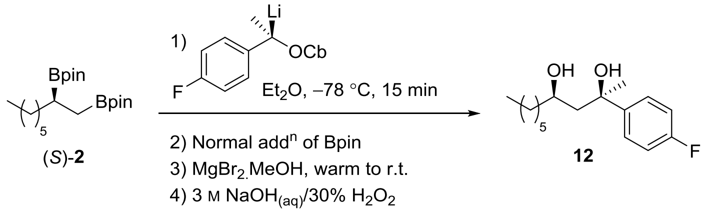

(*S*)-1-(4-Fluorophenyl)ethyl diisopropylcarbamate (**51**) (146 mg, 0.55 mmol, 1.00 eq.) and anhydrous Et_2O (2.73 mL) were added to a flame-dried Schlenk-tube purged with N_2. The solution was cooled to –78 °C (dry ice/acetone) before adding *sec*-BuLi (0.47 mL, 1.30 M, 0.62 mmol, 1.12 eq.) dropwise over 5 min and leaving to react for 15 min at this temperature. (*S*)-**2** (240 mg, 0.66 mmol, 1.20 eq.) was dissolved in anhydrous Et_2O (0.66 mL) and added dropwise to the reaction mixture over 1 min before leaving to react for a further 1 hr at the same temperature. A 1 M solution of $MgBr_2$ in MeOH (0.82 mL, 0.82 mmol, 1.50 eq.) was added dropwise over 2 min and the mixture was allowed to react for a further 2 min before warming to ambient temperature. Water (10 mL) was added and the organic phase was collected,

followed by extraction of the aqueous phase (3×15 mL Et_2O). The combined organic phases were dried over $MgSO_4$, filtered and concentrated under reduced pressure. The crude residue was dissolved in THF (6 mL) and one crystal of BHT was added, then the mixture was subsequently cooled to 0 °C (ice/water). A 2:1 v:v mixture of 3 M aqueous NaOH (4 mL) and 30% aqueous H_2O_2 (2 mL) was prepared at 0 °C (ice/water) and degassed by gently bubbling N_2 through the solution. This aqueous solution was added dropwise to the vigorously stirred reaction mixture, which was subsequently warmed to ambient temperature and allowed to react for 1 hr. 2 M aqueous HCl (10 mL) was carefully added and the reaction mixture was extracted with EtOAc (3×20 mL). The combined organic fractions were dried over Na_2SO_4, filtered and concentrated under reduced pressure. The crude residue was purified by flash column chromatography (SiO_2; 80:20 pentane:EtOAc) to yield **12** (121 mg, 82%) as a viscous colorless oil.

TLC: R_f = 0.20 (80:20 pentane:EtOAc)

^{1}H NMR: (500 MHz, $CDCl_3$) δ 7.47 – 7.37 (m, 2H, ArH), 7.05 – 6.96 (m, 2H, ArH), 4.15 – 4.05 (m, 1H, CHO), 3.67 (s, 1H, OH), 2.76 (s, 1H, OH), 1.85 – 1.73 (m, 2H, CHOC*H*$_2$C), 1.64 (s, 3H, CH_3), 1.55 – 1.16 (m, 10H, 5×CH_2), 0.88 (t, J = 7.1 Hz, 3H, CH_3) ppm

^{13}C NMR: (126 MHz, $CDCl_3$) δ 161.8 (d, J = 244.7 Hz, ArCF), 145.3 (ArC), 126.3 (d, J = 7.9 Hz, ArH), 115.0 (d, J = 21.2 Hz, ArH), 114.9 (ArH), 74.7 (CO), 70.0 (CHO), 49.5 (CHO*C*H_2C), 38.5 (CH_2), 31.9 (CH_2), 29.4 (CH_2), 28.2 (C*C*H_3), 25.5 (CH_2), 22.7 (CH_2), 14.2 (CH_3) ppm

HRMS (*m*/*z*): (ESI) calc'd for $C_{16}H_{25}O_2FNa$ $[M+Na]^+$: 291.1731, found: 291.1732

IR (neat) ν_{max}: 3344, 2928, 2857, 1602, 1509, 1416, 11375, 1225, 1159, 1088 and 834 cm^{-1}

$[\alpha]_D^{24}$: +16.0 (c = 1.0, $CHCl_3$)

3.2 1,2-Bis(Boronic Ester) Scope

tert-Butyl (6*S*,8*R*)-6,8-dihydroxy-8-phenylnonanoate (13):

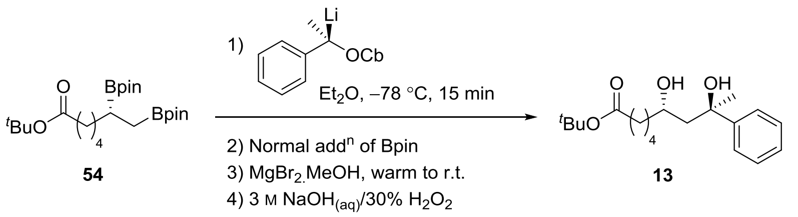

(*S*)-1-Phenylethyl diisopropylcarbamate (**50**) (138 mg, 0.55 mmol, 1.00 eq.) and anhydrous Et_2O (2.77 mL) were added to a flame-dried Schlenk-tube purged with N_2. The solution was cooled to −78 °C (dry ice/acetone) before adding *sec*-BuLi (0.48 mL, 1.30 M, 0.62 mmol, 1.12 eq.) dropwise over 5 min and leaving to react for 15 min at this temperature. **54** (291 mg, 0.66 mmol, 1.20 eq.) was dissolved in anhydrous Et_2O (0.66 mL) and added dropwise to the reaction mixture over 1 min before leaving to react for a further 1 hr at the same temperature. A 1 M solution of $MgBr_2$ in MeOH (0.83 mL, 0.83 mmol, 1.50 eq.) was added dropwise over 2 min and the mixture was allowed to react for a further 2 min before warming to ambient temperature. Water (10 mL) was added and the organic phase was collected, followed by extraction of the aqueous phase (3×15 mL Et_2O). The combined organic phases were dried over $MgSO_4$, filtered and concentrated under reduced pressure. The crude residue was dissolved in THF (6 mL) and one crystal of BHT was added, then the mixture was subsequently cooled to 0 °C (ice/water). A 2:1 v:v mixture of 3 M aqueous NaOH (4 mL) and 30% aqueous H_2O_2 (2 mL) was prepared at 0 °C (ice/water) and degassed by gently bubbling N_2 through the solution. This aqueous solution was added dropwise to the vigorously stirred reaction mixture, which was subsequently warmed to ambient temperature and allowed to react for 1 hr. Water (10 mL) was added and the reaction mixture was extracted with EtOAc (3×20 mL). The combined organic fractions were dried over Na_2SO_4, filtered and concentrated under reduced pressure. The crude residue was purified by flash column chromatography (SiO_2; 70:30 pentane:EtOAc) to yield **13** (126 mg, 71%) as a viscous colorless oil.

TLC: R_f = 0.40 (60:40 pentane:EtOAc)

^{1}H NMR: (500 MHz, $CDCl_3$) δ 7.44 – 7.38 (m, 2H, ArH), 7.37 – 7.29 (m, 2H, ArH), 7.26 – 7.18 (m, 1H, ArH), 4.38 (s, 1H, OH), 3.50 – 3.33 (m, 1H, CHO), 2.89 (s, 1H, OH), 2.15 (t, *J* = 7.4 Hz, 2H, $COCH_2$), 2.01 (dd, *J* = 14.6, 2.0 Hz, 1H, $CHOCH^aH^bC$), 1.90 (dd, *J* = 14.6, 10.6 Hz, 1H, $CHOCH^aH^bC$), 1.55 – 1.15 (m, 6H, 3×CH_2), 1.50 (s, 3H, CCH_3), 1.41 (s, 9H, $OC(CH_3)_3$) ppm

^{13}**C NMR:** (126 MHz, $CDCl_3$) δ 173.3 (C=O), 147.6 (ArC), 128.3 (ArH), 126.5 (ArH), 125.0 (ArH), 80.3 (O*C*(CH_3)$_3$), 75.7 (CO), 70.1 (CHO), 48.4 (CHO*C*H_2C), 37.9 (CH_2), 35.4 (C=O*C*H_2), 32.7 (C*C*H_3), 28.2 (OC(*C*H_3)$_3$), 24.9 (CH_2), 24.7 (CH_2) ppm

HRMS (*m*/*z*): (ESI) calc'd for $C_{19}H_{30}O_4Na$ $[M+Na]^+$: 345.2036, found: 345.2034

IR (neat) ν_{max}: 3378 (OH), 2975, 2932, 1726 (CO), 1366, 1147, 1098, 845, 766 and 701 cm^{-1}

$[\alpha]_D^{24}$**:** +11.0 (*c* = 1.0, $CHCl_3$)

(1*R*,3*R*)-1-(4-Methoxyphenyl)-3-phenylbutane-1,3-diol (14):

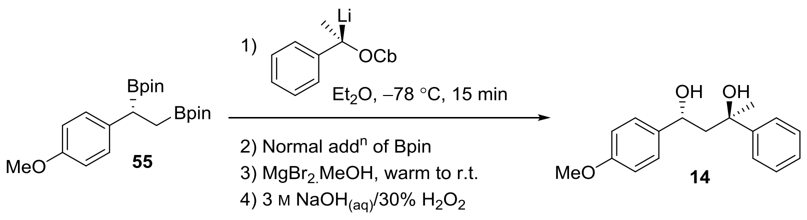

(*S*)-1-Phenylethyl diisopropylcarbamate (**50**) (138 mg, 0.55 mmol, 1.00 eq.) and anhydrous Et_2O (2.77 mL) were added to a flame-dried Schlenk-tube purged with N_2. The solution was cooled to −78 °C (dry ice/acetone) before adding *sec*-BuLi (0.48 mL, 1.30 M, 0.62 mmol, 1.12 eq.) dropwise over 5 min and leaving to react for 15 min at this temperature. **55** (258 mg, 0.66 mmol, 1.20 eq.) was dissolved in anhydrous Et_2O (0.66 mL) and added dropwise to the reaction mixture over 1 min before leaving to react for a further 1 hr at the same temperature. A 1 M solution of $MgBr_2$ in MeOH (0.83 mL, 0.83 mmol, 1.50 eq.) was added dropwise over 2 min and the mixture was allowed to react for a further 2 min before warming to ambient temperature. Water (10 mL) was added and the organic phase was collected, followed by extraction of the aqueous phase (3×15 mL Et_2O). The combined organic phases were dried over $MgSO_4$, filtered and concentrated under reduced pressure. The crude residue was dissolved in THF (6 mL) and one crystal of BHT was added, then the mixture was subsequently cooled to 0 °C (ice/water). A 2:1 v:v mixture of 3 M aqueous NaOH (4 mL) and 30% aqueous H_2O_2 (2 mL) was prepared at 0 °C (ice/water) and degassed by gently bubbling N_2 through the solution. This aqueous solution was added dropwise to the vigorously stirred reaction mixture, which was subsequently warmed to ambient temperature and allowed to react for 1 hr. 2 M aqueous HCl (10 mL) was carefully added and the reaction mixture was extracted with EtOAc

(3×20 mL). The combined organic fractions were dried over Na_2SO_4, filtered and concentrated under reduced pressure. The crude residue was purified by flash column chromatography (SiO_2; 80:20 pentane:EtOAc) to yield **14** (116 mg, 77%) as a viscous colorless oil.

TLC: R_f = 0.15 (80:20 pentane:EtOAc)

^{1}H NMR: (400 MHz, $CDCl_3$) δ 7.53 – 7.48 (m, 2H, ArH), 7.44 – 7.37 (m, 2H, ArH), 7.32 – 7.26 (m, 1H, ArH), 7.18 – 7.12 (m, 2H, ArH), 6.86 – 6.81 (m, 2H, ArH), 4.43 (dd, *J* = 10.8, 2.2 Hz, 1H, CHO), 3.78 (s, 3H, OCH_3), 2.27 (dd, *J* = 14.8, 10.8 Hz, 1H, CH^aH^b), 2.16 (dd, *J* = 14.8, 2.2 Hz, 1H, CH^aH^b), 1.53 (s, 3H, CH_3) ppm

^{13}C NMR: (101 MHz, $CDCl_3$) δ 159.2 (Ar*C*OCH_3), 147.4 (ArC), 136.7 (ArC), 128.4 (ArH), 126.9 (ArH), 126.6 (ArH), 125.1 (ArH), 114.0 (ArH), 75.6 (CO), 72.6 (CHO), 55.4 (OCH_3), 50.9 (CH_2), 32.6 (CH_3) ppm

HRMS (*m/z*): (ESI) calc'd for $C_{17}H_{20}O_3Na$ $[M+Na]^+$: 295.1305, found: 295.1308

IR (neat) ν_{max}: 3331 (OH), 2971, 2836, 1611, 1511, 1443, 1242, 1173, 1072, 1029, 828, 762 and 701 cm^{-1}

$[\alpha]_D^{24}$: –28.0 (*c* = 1.0, $CHCl_3$)

(4*S*,6*R*)-4,6-Dihydroxy-6-phenylheptyl diisopropylcarbamate (15):

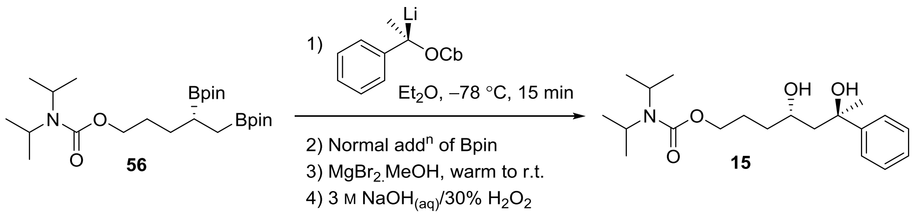

(*S*)-1-Phenylethyl diisopropylcarbamate (**50**) (138 mg, 0.55 mmol, 1.00 eq.) and anhydrous Et_2O (2.77 mL) were added to a flame-dried Schlenk-tube purged with N_2. The solution was cooled to –78 °C (dry ice/acetone) before adding *sec*-BuLi (0.48 mL, 1.30 M, 0.62 mmol, 1.12 eq.) dropwise over 5 min and leaving to react for 15 min at this temperature. **56** (310 mg, 0.66 mmol, 1.20 eq.) was dissolved in anhydrous Et_2O (0.66 mL) and added dropwise to the reaction mixture over 1 min before leaving to react for a further 1 hr at the same temperature. A 1 M solution of $MgBr_2$ in MeOH (0.83 mL, 0.83 mmol, 1.50 eq.) was added dropwise over 2 min and the mixture was allowed to react for a further 2 min before warming to ambient

temperature. Water (10 mL) was added and the organic phase was collected, followed by extraction of the aqueous phase (3×15 mL Et_2O). The combined organic phases were dried over $MgSO_4$, filtered and concentrated under reduced pressure. The crude residue was dissolved in THF (6 mL) and one crystal of BHT was added, then the mixture was subsequently cooled to 0 °C (ice/water). A 2:1 v:v mixture of 3 M aqueous NaOH (4 mL) and 30% aqueous H_2O_2 (2 mL) was prepared at 0 °C (ice/water) and degassed by gently bubbling N_2 through the solution. This aqueous solution was added dropwise to the vigorously stirred reaction mixture, which was subsequently warmed to ambient temperature and allowed to react for 1 hr. 2 M aqueous HCl (10 mL) was carefully added and the reaction mixture was extracted with EtOAc (3×20 mL). The combined organic fractions were dried over Na_2SO_4, filtered and concentrated under reduced pressure. The crude residue was purified by flash column chromatography (SiO_2; 60:40 pentane:EtOAc) to yield **15** (149 mg, 76%) as a viscous colorless oil.

TLC: R_f = 0.38 (60:40 pentane:EtOAc)

^{1}H NMR: (400 MHz, $CDCl_3$) δ 7.50 – 7.41 (m, 2H, ArH), 7.39 – 7.30 (m, 2H, ArH), 7.26 – 7.19 (m, 1H, ArH), 4.20 – 3.56 (br m, 1H, NC*H*(CH_3)$_2$), 4.07 – 3.94 (m, 2H, OCH_2), 3.56 – 2.90 (br m, 1H, NC*H*(CH_3)$_2$), 3.50 – 3.43 (m, 1H, CHO), 2.02 (dd, *J* = 14.5, 2.2 Hz, 1H, $CHCH^aH^bC$), 1.94 (dd, *J* = 14.6, 10.2 Hz, 1H, $CHCH^aH^bC$), 1.70 – 1.35 (m, 4H, 2×CH_2), 1.52 (s, 3H, CH_3), 1.17 (d, *J* = 6.8 Hz, 12H, NCH(C*H*$_3$)$_2$) ppm

^{13}C NMR: (101 MHz, $CDCl_3$) δ 156.0 (C=O), 147.5 (Ar*C*OCH_3), 128.4 (ArH), 126.5 (ArH), 125.0 (ArH), 75.7 (CO), 70.0 (CHO), 64.5 (OCH_2), 48.6 (CH*C*H$_2$C), 34.6 (CH_2), 32.8 (CH_3), 25.2 (CH_2), 20.9 (br, NCH(*C*H$_3$)$_2$) ppm. The signal corresponding to N*C*H(CH_3)$_2$ was too broad to observe.

HRMS (*m/z*): (ESI) calc'd for $C_{20}H_{33}O_4NNa$ $[M+Na]^+$: 374.2302, found: 374.2311

IR (neat) ν_{max}: 3376 (OH), 2970, 2933, 1665 (CO), 1441, 1369, 1299, 1134, 1069, 767 and 701 cm^{-1}

$[\alpha]_D^{24}$**:** +11.0 (*c* = 1.0, $CHCl_3$)

(2*R*,4*S*)-6,6-Dimethyl-2-phenylheptane-2,4-diol (16):

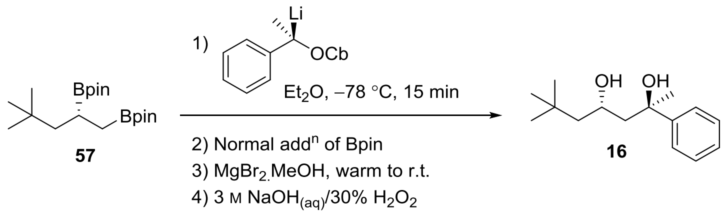

(*S*)-1-Phenylethyl diisopropylcarbamate (**50**) (138 mg, 0.55 mmol, 1.00 eq.) and anhydrous Et_2O (2.77 mL) were added to a flame-dried Schlenk-tube purged with N_2. The solution was cooled to −78 °C (dry ice/acetone) before adding *sec*-BuLi (0.48 mL, 1.30 M, 0.62 mmol, 1.12 eq.) dropwise over 5 min and leaving to react for 15 min at this temperature. **57** (234 mg, 0.66 mmol, 1.20 eq.) was dissolved in anhydrous Et_2O (0.66 mL) and added dropwise to the reaction mixture over 1 min before leaving to react for a further 1 hr at the same temperature. A 1 M solution of $MgBr_2$ in MeOH (0.83 mL, 0.83 mmol, 1.50 eq.) was added dropwise over 2 min and the mixture was allowed to react for a further 2 min before warming to ambient temperature. Water (10 mL) was added and the organic phase was collected, followed by extraction of the aqueous phase (3×15 mL Et_2O). The combined organic phases were dried over $MgSO_4$, filtered and concentrated under reduced pressure. The crude residue was dissolved in THF (6 mL) and one crystal of BHT was added, then the mixture was subsequently cooled to 0 °C (ice/water). A 2:1 v:v mixture of 3 M aqueous NaOH (4 mL) and 30% aqueous H_2O_2 (2 mL) was prepared at 0 °C (ice/water) and degassed by gently bubbling N_2 through the solution. This aqueous solution was added dropwise to the vigorously stirred reaction mixture, which was subsequently warmed to ambient temperature and allowed to react for 1 hr. 2 M aqueous HCl (10 mL) was carefully added and the reaction mixture was extracted with EtOAc (3×20 mL). The combined organic fractions were dried over Na_2SO_4, filtered and concentrated under reduced pressure. The crude residue was purified by flash column chromatography (SiO_2; 80:20 pentane:EtOAc) to yield **16** (72 mg, 55%) as a viscous colorless oil.

TLC: R_f = 0.23 (80:20 pentane:EtOAc)

^{1}H NMR: (500 MHz, $CDCl_3$) δ 7.44 (d, J = 7.4 Hz, 2H, ArH), 7.35 (t, J = 7.7 Hz, 2H, ArH), 7.24 (t, J = 7.3 Hz, 1H, ArH), 4.21 (br s, 1H, OH), 3.66 – 3.54 (m, 1H, CHO), 2.23 (br s, 1H, OH), 2.01 – 1.97 (m, 2H, CH_2), 1.51 (s, 3H, CCH_3), 1.39 (dd, J = 14.6, 7.1 Hz, 1H, CHOCH^aH^bC), 1.27 (dd, J = 14.6, 3.5 Hz, 1H, CHOCH^aH^bC), 0.79 (s, 9H, $C(CH_3)_3$) ppm

^{13}C NMR: (126 MHz, $CDCl_3$) δ 147.5 (ArC), 128.3 (ArH), 126.5 (ArH), 125.0 (ArH), 75.7 (CO), 68.5 (CHO), 52.6 (CHO*C*H_2C), 50.5 (CH_2), 32.8 (C*C*H_3), 30.3 (O*C*(CH_3)$_3$), 30.1 (OC(*C*H_3)$_3$) ppm

HRMS (*m/z*): (ESI) calc'd for $C_{15}H_{24}O_2Na$ $[M+Na]^+$: 259.1669, found: 259.1678

IR (neat) ν_{max}: 3369 (OH), 2957, 2922, 2867, 1364, 1145, 1075, 853, 767 and 700 cm^{-1}

$[\alpha]_D^{24}$**:** +22.0 (c = 1.0, $CHCl_3$)

(2*R*,4*S*,6*S*)-6-((*tert*-Butyldiphenylsilyl)oxy)-2-phenylheptane-2,4-diol (17):

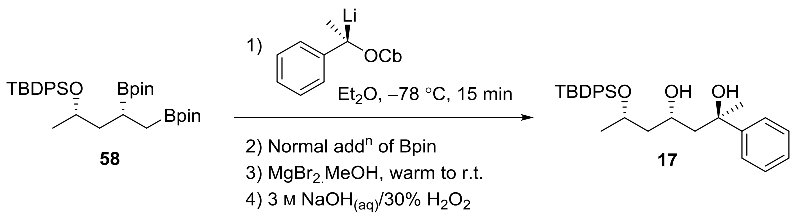

(*S*)-1-Phenylethyl diisopropylcarbamate (**50**) (138 mg, 0.55 mmol, 1.00 eq.) and anhydrous Et_2O (2.77 mL) were added to a flame-dried Schlenk-tube purged with N_2. The solution was cooled to –78 °C (dry ice/acetone) before adding *sec*-BuLi (0.48 mL, 1.30 M, 0.62 mmol, 1.12 eq.) dropwise over 5 min and leaving to react for 15 min at this temperature. **58** (384 mg, 0.66 mmol, 1.20 eq.) was dissolved in anhydrous Et_2O (0.66 mL) and added dropwise to the reaction mixture over 1 min before leaving to react for a further 1 hr at the same temperature. A 1 M solution of $MgBr_2$ in MeOH (0.83 mL, 0.83 mmol, 1.50 eq.) was added dropwise over 2 min and the mixture was allowed to react for a further 2 min before warming to ambient temperature. Water (10 mL) was added and the organic phase was collected, followed by extraction of the aqueous phase (3×15 mL Et_2O). The combined organic phases were dried over $MgSO_4$, filtered and concentrated under reduced pressure. The crude residue was dissolved in THF (6 mL) and one crystal of BHT was added, then the mixture was subsequently cooled to 0 °C (ice/water). A 2:1 v:v mixture of 3 M aqueous NaOH (4 mL) and 30% aqueous H_2O_2 (2 mL) was prepared at 0 °C (ice/water) and degassed by gently bubbling N_2 through the solution. This aqueous solution was added dropwise to the vigorously stirred reaction mixture, which was subsequently warmed to ambient temperature and allowed to react for 1 hr. 2 M aqueous HCl (10 mL) was carefully added and the reaction mixture was extracted with EtOAc (3×20 mL). The combined organic fractions were dried over Na_2SO_4, filtered and concentrated

under reduced pressure. The crude residue was purified by flash column chromatography (SiO_2; 80:20 pentane:EtOAc) to yield **17** (234 mg, 91%) as white solid.

TLC: R_f = 0.34 (80:20 pentane:EtOAc)

m.p.: 82 – 85 °C (CH_2Cl_2)

^{1}H NMR: (400 MHz, $CDCl_3$) δ 7.73 – 7.21 (m, 15H, ArH), 4.86 (br s, 1H, OH), 4.03 – 3.90 (m, 1H, CHOSi), 3.88 – 3.31 (m, 2H, CHO and OH), 1.99 (dd, J = 14.2, 10.7 Hz, 1H, CHC$H^a$$H^b$C), 1.92 (dd, J = 14.2, 2.3 Hz, 1H, CHC$H^a$$H^b$C), 1.74 – 1.61 (m, 1H, CHC$H^a$$H^b$CH), 1.51 (s, 3H, CH_3), 1.46 – 1.36 (m, 1H, CHC$H^a$$H^b$CH), 0.96 (s, 9H, SiC($CH_3$)$_3$), 0.86 (d, J = 6.1 Hz, 3H, CHCH_3) ppm

^{13}C NMR: (101 MHz, $CDCl_3$) δ 148.0 (ArC), 135.9 (ArH), 135.9 (ArH), 134.3 (ArC), 133.2 (ArC), 130.0 (ArH), 129.8 (ArH), 128.2 (ArH), 127.9 (ArH), 127.7 (ArH), 126.3 (ArH), 125.2 (ArH), 75.3 (CO), 70.9 (CHOSi), 69.8 (CHO), 49.0 (CH*C*H_2C), 46.4 (CH_2), 32.3 (CH_3), 27.0 (SiC(*C*H_3)$_3$), 24.2 (CHCH_3), 19.2 (Si*C*(CH_3)$_3$) ppm

HRMS (*m/z*): (ESI) calc'd for $C_{29}H_{38}O_3SiNa$ $[M+Na]^+$: 485.2482, found: 485.2480

IR (neat) ν_{max}: 3324 (OH), 2967, 2940, 2857, 1427, 1380, 1102, 1070, 1006, 739 and 699 cm^{-1}

$[\alpha]_D^{24}$**:** +20.0 (c = 1.0, $CHCl_3$)

(2*R*,4*S*,6*S*)-6,10-Dimethyl-2-phenylundec-9-ene-2,4-diol (18):

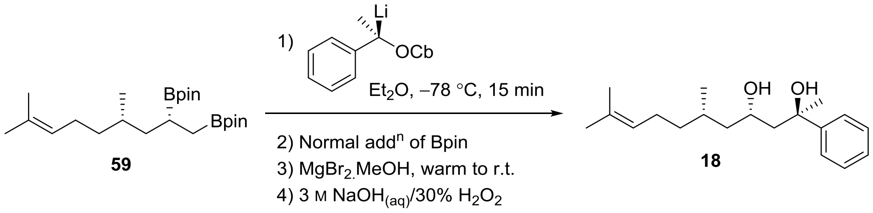

(*S*)-1-Phenylethyl diisopropylcarbamate (**50**) (138 mg, 0.55 mmol, 1.00 eq.) and anhydrous Et_2O (2.77 mL) were added to a flame-dried Schlenk-tube purged with N_2. The solution was cooled to −78 °C (dry ice/acetone) before adding *sec*-BuLi (0.48 mL, 1.30 M, 0.62 mmol, 1.12 eq.) dropwise over 5 min and leaving to react for 15 min at this temperature. **59** (270 mg, 0.66 mmol, 1.20 eq.) was dissolved in anhydrous Et_2O (0.66 mL) and added dropwise to the reaction mixture over 1 min before leaving to react for a further 1 hr at the same temperature. A 1 M

solution of $MgBr_2$ in MeOH (0.83 mL, 0.83 mmol, 1.50 eq.) was added dropwise over 2 min and the mixture was allowed to react for a further 2 min before warming to ambient temperature. Water (10 mL) was added and the organic phase was collected, followed by extraction of the aqueous phase (3×15 mL Et_2O). The combined organic phases were dried over $MgSO_4$, filtered and concentrated under reduced pressure. The crude residue was dissolved in THF (6 mL) and one crystal of BHT was added, then the mixture was subsequently cooled to 0 °C (ice/water). A 2:1 v:v mixture of 3 M aqueous NaOH (4 mL) and 30% aqueous H_2O_2 (2 mL) was prepared at 0 °C (ice/water) and degassed by gently bubbling N_2 through the solution. This aqueous solution was added dropwise to the vigorously stirred reaction mixture, which was subsequently warmed to ambient temperature and allowed to react for 1 hr. 2 M aqueous HCl (10 mL) was carefully added and the reaction mixture was extracted with EtOAc (3×20 mL). The combined organic fractions were dried over Na_2SO_4, filtered and concentrated under reduced pressure. The crude residue was purified by flash column chromatography (SiO_2; 80:20 pentane:EtOAc) to yield **18** (140 mg, 87%) as a viscous colorless oil.

TLC: R_f = 0.25 (80:20 pentane:EtOAc)

^{1}H NMR: (500 MHz, $CDCl_3$) δ 7.43 (d, J = 7.5 Hz, 2H, ArH), 7.35 (t, J = 7.5 Hz, 2H, ArH), 7.23 (t, J = 7.5 Hz, 1H, ArH), 5.02 (t, J = 7.1 Hz, 1H, =CH), 4.18 (br s, 1H, OH), 3.57 – 3.47 (m, 1H, CHO), 2.28 (br s, 1H, OH), 2.06 (dd, J = 14.6, 1.8 Hz, 1H, CHOCH^aH^bC), 1.97 – 1.79 (m, 2H, =CHCH_2), 1.85 (dd, J = 14.6, 10.5 Hz, 1H, CHOC$H^a$$H^b$C), 1.67 (s, 3H, =C(C$H_3$)^a(CH$_3$)^b), 1.56 (s, 3H, =C(CH$_3$)^a(C$H_3$)^b), 1.52 (s, 3H, CCH$_3$), 1.45 – 1.31 (m, 2H, C*H*CH$_3$ and CH^aH^b), 1.30 – 1.15 (m, 2H, CH^aH^b and CH^aH^b), 1.11 – 0.99 (m, 1H, CH^aH^b), 0.71 (d, J = 6.5 Hz, 3H, CHCH_3) ppm

^{13}C NMR: (126 MHz, $CDCl_3$) δ 147.6 (ArC), 131.5 (=*C*(CH$_3$)$_2$), 128.3 (ArH), 126.5 (ArH), 124.9 (ArH), 124.7 (=CH), 75.7 (CO), 68.8 (CHO), 48.4 (CHO*C*H$_2$C), 45.8 (CH$_2$), 37.2 (CH$_2$), 32.8 (C*C*H$_3$), 29.1 (*C*HCH$_3$), 25.8 (=C(C^aH$_3$)(C^{b}H$_3$)), 25.4 (=CH*C*H$_2$), 19.9 (CH*C*H$_3$), 17.8 (=C(C^{a}H$_3$)(C^bH$_3$)) ppm

HRMS (*m/z*): (ESI) calc'd for $C_{19}H_{30}O_2Na$ $[M+Na]^+$: 313.2138, found: 313.2135

IR (neat) ν_{max}: 3339, 2965, 2914, 1444, 1375, 1072, 765 and 700 cm^{-1}

$[\alpha]_D^{24}$**:** +8.0 (c = 1.0, $CHCl_3$)

(2*R*,4*S*,5*S*,7*R*)-8-((*tert*-Butyldiphenylsilyl)oxy)-5,7-dimethyl-2-phenyloctane-2,4-diol (19):

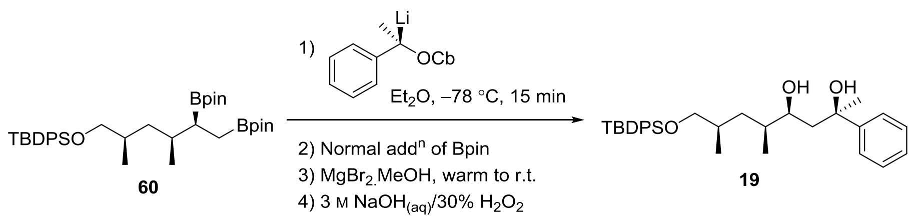

(*S*)-1-Phenylethyl diisopropylcarbamate (**50**) (138 mg, 0.55 mmol, 1.00 eq.) and anhydrous Et_2O (2.77 mL) were added to a flame-dried Schlenk-tube purged with N_2. The solution was cooled to −78 °C (dry ice/acetone) before adding *sec*-BuLi (0.48 mL, 1.30 M, 0.62 mmol, 1.12 eq.) dropwise over 5 min and leaving to react for 15 min at this temperature. **60** (412 mg, 0.66 mmol, 1.20 eq.) was dissolved in anhydrous Et_2O (0.66 mL) and added dropwise to the reaction mixture over 1 min before leaving to react for a further 1 hr at the same temperature. A 1 M solution of $MgBr_2$ in MeOH (0.83 mL, 0.83 mmol, 1.50 eq.) was added dropwise over 2 min and the mixture was allowed to react for a further 2 min before warming to ambient temperature. Water (10 mL) was added and the organic phase was collected, followed by extraction of the aqueous phase (3×15 mL Et_2O). The combined organic phases were dried over $MgSO_4$, filtered and concentrated under reduced pressure. The crude residue was dissolved in THF (6 mL) and one crystal of BHT was added, then the mixture was subsequently cooled to 0 °C (ice/water). A 2:1 v:v mixture of 3 M aqueous NaOH (4 mL) and 30% aqueous H_2O_2 (2 mL) was prepared at 0 °C (ice/water) and degassed by gently bubbling N_2 through the solution. This aqueous solution was added dropwise to the vigorously stirred reaction mixture, which was subsequently warmed to ambient temperature and allowed to react for 1 hr. 2 M aqueous HCl (10 mL) was carefully added and the reaction mixture was extracted with EtOAc (3×20 mL). The combined organic fractions were dried over Na_2SO_4, filtered and concentrated under reduced pressure. The crude residue was purified by flash column chromatography (SiO_2; 80:20 pentane:EtOAc) to yield **19** (221 mg, 79%) as a viscous colorless oil.

TLC: R_f = 0.32 (80:20 pentane:EtOAc)

^{1}H NMR: (500 MHz, $CDCl_3$) δ 7.69 – 7.63 (m, 4H, ArH), 7.50 – 7.30 (m, 10H, ArH), 7.28 – 7.21 (m, 1H, ArH), 4.04 – 3.97 (m, 1H, CHO), 3.53 (br s, 1H, OH), 3.52 (dd, *J* = 10.0, 5.3 Hz, 1H, OCH^aH^b), 3.44 (dd, *J* = 10.0, 6.2 Hz, 1H, OCH^aH^b), 2.59 (br s, 1H, OH), 1.87 (dd, *J* = 14.7, 10.7 Hz, 1H, CHOCH^aH^bC), 1.81 – 1.70 (m, 1H, C*H*CH_3), 1.69 (dd, *J* = 14.7, 1.8 Hz, 1H, CHOCH^aH^bC), 1.64 (CCH_3), 1.59 – 1.47 (m, 2H, CHCH_3 and CH^aH^b), 1.06 (s, 9H,

$SiC(CH_3)_3$), 0.99 – 0.93 (m, 1H, CH^aH^b), 0.95 (d, J = 6.7 Hz, 3H, $CHCH_3$), 0.83 (d, J = 6.7 Hz, 3H, $CHCH_3$) ppm

^{13}C NMR: (101 MHz, $CDCl_3$) δ 149.7 (ArC), 135.8 (ArH), 134.0 (ArC), 129.7 (ArH), 128.4 (ArH), 127.8 (ArH), 126.8 (ArH), 124.5 (ArH), 74.8 (CO), 72.5 (CHO), 68.7 (OCH_2), 46.7 ($CHOCH_2C$), 37.0 ($CHCH_2CH$), 36.7 (CH), 33.2 (CH), 28.0 (CCH_3), 27.1 ($SiC(CH_3)_3$), 19.4 ($SiC(CH_3)_3$), 18.4 ($CHCH_3$), 14.6 ($CHCH_3$) ppm

HRMS (*m/z*): (ESI) calc'd for $C_{32}H_{44}O_3SiNa$ $[M+Na]^+$: 527.2952, found: 527.2941

IR (neat) ν_{max}: 3354 (OH), 2957, 2930, 2857, 1427, 1111, 1069, 737, 699 and 614 cm^{-1}

$[\alpha]_D^{24}$: +4.00 (c = 1.0, $CHCl_3$)

1-((3*R*,5*S*)-7-((*tert*-Butyldimethylsilyl)oxy)-3,5-dihydroxyheptyl)-5-(4-fluorophenyl)-2-isopropyl-*N*,4-diphenyl-1*H*-pyrrole-3-carboxamide (20):

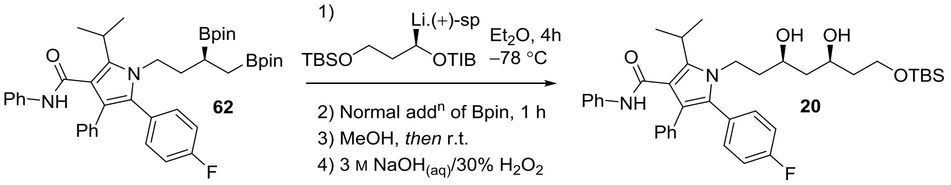

3-((*tert*-Butyldimethylsilyl)oxy)propyl 2,4,6-triisopropylbenzoate (**63**) (117.8 mg, 0.28 mmol, 2 eq.), (+)-sparteine (0.06 mL, 0.28 mmol, 2 eq.) and anhydrous Et_2O (1.18 mL) were added to a flame-dried Schlenk-tube purged with N_2. The solution was cooled to −78 °C (dry ice/acetone) before adding *sec*-BuLi (0.22 mL, 0.28 mmol, 2 eq.) dropwise over 5 min and leaving to react for 4 hr at this temperature. **62** (100 mg, 0.14 mmol, 1 eq.) dissolved in anhydrous Et_2O (0.28 mL) was added dropwise to the reaction mixture over 1 min before leaving to react for a further 1 hr at the same temperature. MeOH (0.1 mL) was added dropwise before allowing the flask to warm to ambient temperature. Water (50 mL) was added and the reaction mixture was extracted with Et_2O (3×50 mL). The combined organic phases were dried over $MgSO_4$, filtered and concentrated under reduced pressure. THF (2 mL) and one crystal of BHT were added, before cooling to 0 °C (ice/water). A 2:1 v:v mixture of 3 M aqueous NaOH (2.74 mL) and 30% aqueous H_2O_2 (1.38 mL) was prepared at 0 °C (ice/water) and degassed by gently bubbling N_2 through the solution. This aqueous solution was added at once to the vigorously stirred reaction mixture, which was subsequently warmed to ambient temperature and allowed to react for 16 hr. Water (50 mL) was added and the reaction mixture was extracted

with Et_2O (3×50 mL). The combined organic phases were dried over $MgSO_4$, filtered and concentrated under reduced pressure. The crude residue was purified by flash column chromatography (SiO_2; pentane:Et_2O 50:50) to yield **20** (50 mg, >95:5, 54%) as a white foam containing 0.05% BHT.

TLC: R_f = 0.39 (40:60 pentane:Et_2O)

^{1}H NMR: (500 MHz, $CDCl_3$) δ 7.21 – 7.13 (m, 9H, ArH), 7.08 – 7.05 (m, 2H, ArH), 7.01 – 7.69 (m, 3H, ArH), 6.86 (s, 1H, NH), 4.16 – 4.08 (m, 2H, NCH^aH^b, O*H*), 4.05 – 3.98 (m, 2H, CH, OH), 3.93 (ddd, *J* = 14.8, 10.9, 5.3 Hz, 1H, NCH^aH^b), 3.88 (*app*. dt, *J* = 9.9, 4.4 Hz, 1H, OCH^aH^b), 3.80 (*app.* td, *J* = 10.1, 9.9, 3.2 Hz, 1H, OCH^aH^b), 3.75 (tdd, *J* = 9.9, 5.1, 3.1 Hz, 1H, CH), 3.58 (sept, *J* = 6.9 Hz, 1H, $CH(CH_3)_2$), 1.73 – 1.59 (m, 4H, NCH_2CH_2, OCH_2CH_2), 1.57 – 1.52 (d, *J* = 6.9 Hz, 6H, $CH(CH_3)_2$), 1.49 – 1.45 (m, 1H, $OHCHCH^aH^b$), 1.23 – 1.19 (m, 1H, $OHCHCH^aH^b$), 0.89 (s, 9H, $SiC(CH_3)_3$), 0.07 (s, 6H, $Si(CH_3)_2$) ppm

^{13}C NMR: (126 MHz, $CDCl_3$) δ 164.8 (C=O), 162.2 (d, J_{C-F} = 247.6 Hz, ArF), 141.6 (C), 138.4 (C), 134.7 (C), 133.2 (d, J_{C-F} = 7.8 Hz, ArH and C), 130.5 (ArH), 128.7 (C), 128.6 (ArH), 128.4 (d, J_{C-F} = 3.5 Hz, C), 128.3 (ArH), 126.5 (ArH), 123.4 (ArH), 121.7 (C), 119.5 (ArH), 115.3 (d, J_{C-F} = 21.3 Hz, ArH), 74.0 (CH), 69.9 (CH), 62.9 (OCH_2), 42.8 ($OHCHCH_2$), 41.4 (NCH_2), 39.2 (OCH_2CH_2), 26.1 (*C*H($CH_3)_2$, 25.8 ($SiC(CH_3)_3$), 21.6 ($CH(CH_3)_2$), 18.0 (SiC), -5.6 ($Si(CH_3)_2$) ppm (*observed signals*)

HRMS (*m/z*): (ESI) calc'd for $C_{39}H_{53}FN_2O_4Si$ $[M+H]^+$ 659.3675, found: 659.3681

IR (neat) ν_{max}: 3411, 2928, 2856, 1644, 1509, 1312, 1076 and 835 cm^{-1}

$[\alpha]_{24}^{D}$ **:** +12.0 (*c* = 1.0, $CHCl_3$)

4. Diboration of divinyl carbinols

3.1 Synthesis of starting materials

Triethyl(penta-1,4-dien-3-yloxy)silane (**25b**),[23] triisopropyl(penta-1,4-dien-3-yloxy)silane (**25c**)[24] and penta-1,4-dien-3-yl benzoate (**25f**)[25] were prepared according to literature procedures.

tert-Butyldimethyl(penta-1,4-dien-3-yloxy)silane (25a)

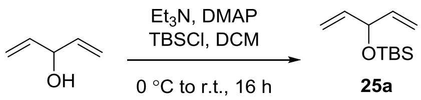

25a

According to the literature,[26] 1,4-pentadien-3-ol (2.89 mL, 30 mmol, 1.0 eq.), triethylamine (6.2 mL, 45 mmol, 1.5 eq.) and DMAP (363 mg, 3.0 mmol, 0.1 eq.) were dissolved in anhydrous dichloromethane (50 mL) and cooled to 0 °C (ice/water) under N_2 atmosphere. *tert*-butyldimethylsilyl chloride (5.4 g, 36 mmol, 1.2 eq.) was added portion wise, then the reaction mixture was allowed to warm to ambient temperature and stirred for 16 hr. The reaction was quenched by the addition of H_2O (25 mL) and the aqueous phase was extracted with dichloromethane (3×25 mL). The combined organic phases were washed with saturated $NH_4Cl_{(aq)}$ solution (50 mL) and brine (50 mL). After drying over $MgSO_4$ and filtration the solvent was removed under reduced pressure to afford the crude oil, which was purified by flash column chromatography (SiO_2; pentane:Et_2O 95:5) to afford the protected alcohol **25a** (SiO_2; 5.9 g, 99%) as a colorless oil.

TLC: R_f = 0.45 (pentane)

^{1}H NMR: (400 MHz, $CDCl_3$) δ 5.81 (ddd, J = 17.1, 10.3, 5.4 Hz, 2H, CH=CH_2), 5.22 (ddd, J = 17.1, 1.6, 1.5 Hz, 2H, CH=CH^aH^b), 5.06 (ddd, J = 10.3, 1.6, 1.5 Hz, 2H, CH=CH^aH^b), 5.06 (dp, J = 5.4, 1.6 Hz, 1H, CHOTBS), 0.92 (s, 9H, OTBS), 0.07 (s, 6H, OTBS) ppm

^{13}C NMR: (101 MHz, $CDCl_3$) δ 140.3 (H_2C=CH), 113.9 (H_2C=CH), 74.8 (CHOTBS), 26.0 (OTBS), 18.5 (OTBS), −4.6 (OTBS) ppm

The data obtained matched those reported in the literature.[11]

---

[23] S.T.A. Shah and P.J. Guiry, *Org. Biomol. Chem.* **2008**, *6*, 2168.
[24] B. Nguyen and J.M. Brown, *Adv. Synth. Catal.* **2009**, *351*, 1333.
[25] E.E. Nagy, I.F.D. Hyatt, K.E. Gettys, S.T. Yeazell, S.K. Frempong Jr. and M.P. Croatt, *Org. Lett.* **2013**, *15*, 586.
[26] P. A. Evans, J. Cui, J. Gharpure, A. Polosukhin, H.-R. Zhang, *J. Am. Chem. Soc.* **2003**, *125*, 14702.

***tert*-Butyl(penta-1,4-dien-3-yloxy)diphenylsilane (25e)**

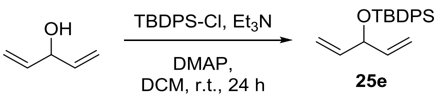

1,4-Pentadien-3-ol (1.16 mL, 12 mmol, 1.0 eq.), triethylamine (3.3 mL, 24 mmol, 1.5 eq.) and DMAP (145 mg, 1.2 mmol, 0.1 eq.) were dissolved in anhydrous dichloromethane (20 mL) and cooled to 0 °C (ice/water) under N_2 atmosphere. *tert*-Butyldiphenylsilyl chloride (4.9 g, 18 mmol, 1.5 eq.) was added portion wise, then the reaction mixture was allowed to warm to ambient temperature and stirred for 16 hr. The reaction was quenched by the addition of H_2O (15 mL) and the aqueous phase was extracted with CH_2Cl_2 (3× 10 mL). The combined organic phases were washed with saturated aqueous NH_4Cl solution (25 mL) and brine (25 mL). After drying over $MgSO_4$ and filtration the solvent was removed under reduced pressure to afford the crude oil. Purification by flash column chromatography (SiO_2; pentane:Et_2O 40:1) afforded the protected alcohol **25e** (3.8 g, 99%) as a colorless oil.

TLC: R_f = 0.84 (pentane:EtOAc 20:1)

^{1}H NMR: (400 MHz, $CDCl_3$) δ 7.72 – 7.66 (m, 4H, ArH), 7.45 – 7.33 (m, 6H, ArH), 5.86 – 5.74 (m, 2H, C*H*=CH_2), 5.09 (dd, *J* = 17.3, 1.7 Hz, 2H, CH=CH^aH^b), 5.00 (dd, *J* = 10.4, 1.7 Hz, 2H, CH=CHH^b), 4.66 – 4.60 (m, 1H, CHOTBDPS), 1.10 (s, 9H, OSiC$(CH_3)_3Ph_2$) ppm

^{13}C NMR: (101 MHz, $CDCl_3$) δ 139.9 (H_2C=*C*H), 136.1 (ArC), 134.2 (ArCH), 129.7 (ArCH), 127.5 (ArCH), 114.4 (H_2*C*=CH), 75.7 (CHOTBDPS), 27.2 (OSi*C*$(CH_3)_3Ph_2$), 19.5 (OSiC(*C*$H_3)_3Ph_2$) ppm

HRMS (*m*/*z*): (ESI) calc'd for $C_{21}H_{26}OSiNa$ $[M+Na]^+$: 345.1645, found: 345.1631

IR (neat) ν_{max}: 2960, 2931, 2858, 1427, 1110 and 698 cm^{-1}

[(Penta-1,4-dien-3-yloxy)methyl]benzene (25g)

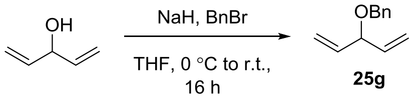

NaH (576 mg, 60% in mineral oil, 14.4 mmol, 1.2 eq.) was added to a solution of 1,4-pentadien-3-ol (1.16 mL, 12 mmol, 1.0 eq.) in THF (20 mL) at 0 °C (ice/water). The reaction mixture was

stirred for 1 hr, then benzyl bromide (2.5 g, 14.4 mmol, 1.2 eq.) was added. After stirring for 16 hr at ambient temperature H_2O (40 mL) was added. The aqueous layer was extracted with Et_2O (3×30 mL) and the combined organic layers were washed with brine (30 mL). After drying over $MgSO_4$ and filtration, the solvent was removed under reduced pressure. Purification by flash column chromatography (SiO_2; pentane:Et_2O 40:1) afforded the protected alcohol **25g** (1.9 g, 90%) as a colorless oil.

TLC: R_f = 0.74 (pentane:EtOAc 10:1)

^{1}H NMR: (400 MHz, $CDCl_3$) δ 7.39 – 7.28 (m, 5H, OBn), 5.86 (ddd, J = 17.2, 10.4, 6.5 Hz, 2H, CH=CH$_2$), 5.29 (dd, J = 17.2, 1.3 Hz, 2H, CH=CH^aH^b), 5.26 – 5.21 (m, 2H, CH=CH^aH^b), 4.54 (s, 2H, CH_2Ph), 4.32 – 4.26 (m, 1H, CHOBn) ppm

^{13}C NMR: (101 MHz, $CDCl_3$) δ 138.6 (H_2C=CH), 137.7 (ArC), 128.5 (ArCH), 127.8 (ArCH), 127.6 (ArCH), 117.0 ($H_2$$C$=CH), 81.1 (CHOBn), 70.0 (CH_2Ph) ppm

HRMS (*m/z*): (ESI) calc'd for $C_{12}H_{14}ONa$ $[M+Na]^+$: 197.0937, found: 197.0938

IR (neat) ν_{max}: 2982, 2864, 1064, 924 and 696 cm^{-1}

3-(Methoxymethoxy)penta-1,4-diene (25h)

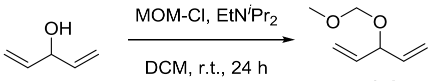

25h

According to a modified literature procedure,[27] *N*,*N*-diisopropylethylamine (5.37 mL, 30.9 mmol, 6.00 eq.) and chloromethyl methyl ether (2.34 mL, 30.9 mmol, 6.00 eq.) were added sequentially to a solution of 1,4-pentadien-3-ol (0.50 mL, 5.14 mmol, 1.00 eq.) in anhydrous dichloromethane (20 mL) that was pre-cooled to 0 °C (ice/water). The resulting solution was warmed to ambient temperature and allowed to react for 24 hr before adding 5% aqueous sodium hydrogen carbonate solution (20 mL). The organic phase was collected, dried over $MgSO_4$ and filtered before concentrating under reduced pressure (*Careful: volatile!*). The crude residue was purified by flash column chromatography (SiO_2; pentane:Et_2O 90:10) to afford the protected alcohol **25h** (545 mg, 83%) as a colorless oil.

[27] L. Hu, C. Yu, Y. Jiang, J. Han, Z. Li, P. Browne, P.R. Race, R.J. Knox, P.F. Searle and E.I. Hyde, *J. Med. Chem.* **2003**, *46*, 4818.

TLC: R_f = 0.35 (90:10 pentane:Et_2O)

^{1}H NMR: (400 MHz, $CDCl_3$) δ 5.80 (ddd, J = 17.2, 10.4, 6.5 Hz, 2H, =CH), 5.28 (dt, J = 17.2, 1.3 Hz, 2H, =CH^aH^b), 5.21 (dt, J = 10.4, 1.3 Hz, 2H, =C$H^a H^b$), 4.67 (s, 2H, OCH_2O), 4.52 (tp, J = 6.5, 1.3 Hz, CHO), 3.38 (s, 3H, OCH_3) ppm

^{13}C NMR: (101 MHz, $CDCl_3$) δ 137.2 (=CH), 117.0 (H_2C=), 93.6 (OCH_2O), 77.6 (CHO), 55.5 (OCH_3) ppm

LRMS (*m/z*): (EI) 67.1 ($[H_2C{=}CHCHCH{=}CH_2]^+$, 100%), 97.1 ($[M\text{-}OMe]^+$, 25%). *Despite repeated attempts, a HRMS could not be obtained.*

IR (neat) ν_{max}: 3083, 2986, 2951, 2887, 2823, 1149, 1095, 1031 and 918 cm^{-1}

3.2 Diboration optimization

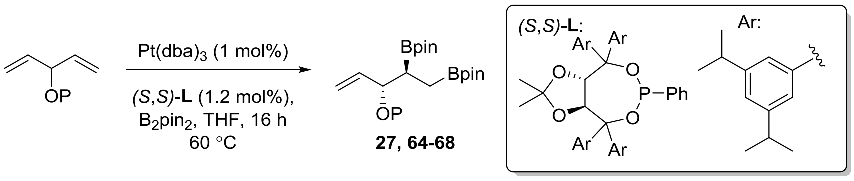

Entry	P	Morken			Nishiyama		
		Yield/ %	d.r.	e.r.	Yield/ %	d.r.	e.r.
1	TBS (**25a**)	90	>95:5	98:2	trace	-	-
2	TES (**25b**)	92	>95:5	-	82	86:14	-
3	TIPS (**25c**)	95	>95:5	-	n.d.	-	-
4	H (**25d**)	-	-	-	-	-	-
5	TBDPS (**25e**)	91	>95:5	-	traces	-	-
6	Bz (**25f**)	-	-	-	n.d.	-	-
7	Bn (**25g**)	22	>95:5	-	38	91:9	-
8	MOM (**25h**)	35	67:3	-	61	91:9	-

Table 2

General procedure A (Morken), alkene diboration:[6]

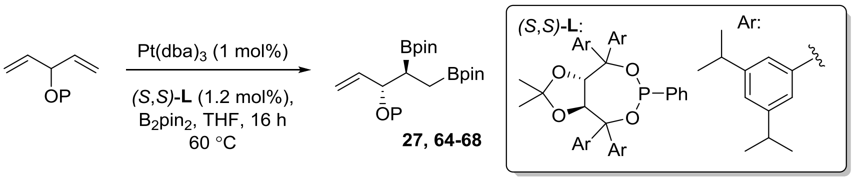

Pt(dba)$_3$ (9.0 mg, 0.01 mmol, 1.0 mol%), (*S*,*S*)-**L** (11.9 mg, 0.012 mmol, 1.2 mol%) and B_2pin_2 (280 mg, 1.10 mmol, 1.10 eq.) were added to a flame-dried Schlenk-tube equipped with a magnetic stirring bar. The tube was sealed and purged three times with N_2. THF (1 mL) was added and the homogeneous mixture was heated to 80 °C (oil bath) for 30 min. The reaction was then cooled to ambient temperature and charged with the desired diene (1.00 mmol, 1.00 eq.) in THF (1 mL). After purging with N_2, the tube was stirred at 60 °C (oil bath) for 16 hr. After cooling to ambient temperature, the solvent was removed under reduced pressure and the crude product was purified by flash column chromatography to yield the desired 1,2-bis(boronic esters).

General Procedure B (Nishiyama), alkene diboration:

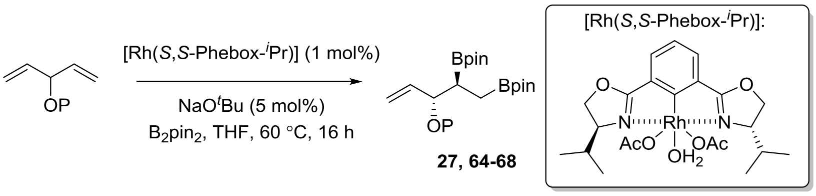

The diene (1.00 mmol, 1.00 eq.) was added to a solution of [Rh(*S*,*S*-Phebox-^{i}Pr)] (0.01 mmol, 1 mol%), B_2pin_2 (1.20 mmol, 1.20 eq.) and NaO^{t}Bu (0.05 mmol, 5 mol%) in THF (2 mL) under N_2. The resulting solution was heated at 60 °C (oil bath) for 16 hr before cooling to ambient temperature and concentrating under reduced pressure. The crude residue was purified by flash column chromatography (SiO_2) to yield the desired 1,2-bis(boronic esters).

{[(3*S*,4*S*)-4,5-Bis(4,4,5,5-tetramethyl-1,3,2-dioxaborolan-2-yl)pent-1-en-3-yl]oxy}(*tert*-butyl)dimethylsilane (27)

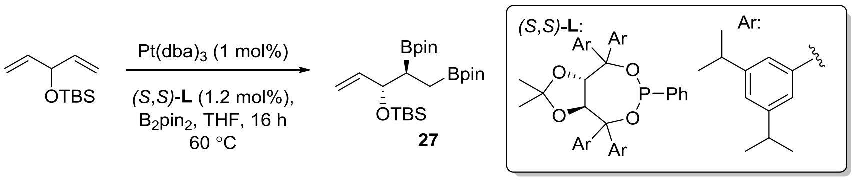

According to general procedure A, but on twice the scale. Pt(dba)$_3$ (18.0 mg, 0.02 mmol, 1.0 mol%), (*S*,*S*)-**L** (23.8 mg, 0.024 mmol, 1.2 mol%) and B_2pin_2 (559 mg, 2.20 mmol, 1.10 eq.) were added to a flame-dried Schlenk-tube equipped with a magnetic stirring bar. The tube was sealed and purged three times with N_2. THF (2 mL) was added and the homogeneous mixture was heated to 80 °C (oil bath) for 30 min. The reaction was cooled to ambient temperature and charged with diene **25a** (397 mg, 2.00 mmol, 1.00 eq.) dissolved in THF (2mL). After purging with N_2, the tube was stirred at 60 °C (oil bath) for 16 hr. After cooling to ambient temperature, the solvent was removed under reduced pressure and the crude product was purified by flash column chromatography (SiO_2; pentane: EtOAc 40:1) yielding the 1,2-bis(boronic ester) **27** (813 mg, 90%, >95:5 d.r.) as a colorless oil.

TLC: R_f = 0.45 (pentane:EtOAc 20:1)

^{1}H NMR: (400 MHz, $CDCl_3$) δ 5.87 (ddd, J = 17.3, 10.2, 7.5 Hz, 1H, C*H*=CH$_2$), 5.07 (ddd, J = 17.3, 1.1, 1.1 Hz, 1H, CH=CH^a*H*^{b}), 4.99 (ddd, J = 10.2, 1.1, 1.1 Hz, 1H, CH=C*H*^{a}H^b), 4.09 (t, J = 7.5 Hz, 1H, CHOTBS), 1.34 (ddd, J = 11.8, 7.5, 4.8 Hz, 1H, CHBpin), 1.22 (s, 24H, Bpin), 0.96 (dd, J = 15.9, 4.8 Hz, 1H, CH^a*H*^{b}Bpin), 0.87 (s, 9H, OTBS), 0.75 (dd, J = 15.9, 11.8 Hz, 1H, C*H*^{a}H^{b}Bpin), 0.04 (s, 3H, OTBS), -0.01 (s, 3H, OTBS) ppm

^{13}C NMR (101 MHz, $CDCl_3$) δ 141.7 (d, H_2C=*C*H), 114.4 (H_2*C*=CH), 83.0 (4×*C*(CH_3)$_2$), 77.4 (CHOTBS), 26.1 (OTBS), 25.2 (C(*C*H$_3$)$_2$), 25.1 (C(*C*H$_3$)$_2$), 24.9 (C(*C*H$_3$)$_2$), 24.9 (C(*C*H$_3$)$_2$), 18.4 (OTBS), -4.0 (OTBS), -4.5 (OTBS) ppm

HRMS (*m*/*z*): (ESI) calc'd for $C_{23}H_{46}B_2O_5SiNa$ [M+Na]$^+$: 475.3202, found: 475.3206

IR (neat) ν_{max}: 2978, 2930, 2857, 1370, 1316, 1141, 834 and 774 cm^{-1}

$[\alpha]_D^{20}$**:** –1.5 (c = 1.0, $CHCl_3$)

{[(3*S*,4*S*)-4,5-Bis(4,4,5,5-tetramethyl-1,3,2-dioxaborolan-2-yl)pent-1-en-3-yl]oxy}triethylsilane (64)

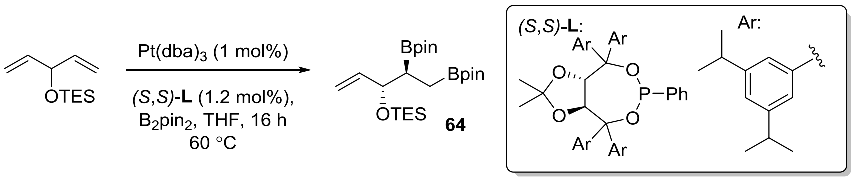

64 was synthesised according to general procedure A, with **25b** as the diene, and purified by flash column chromatography (SiO_2; pentane:EtOAc 20:1) to yield the desired 1,2-bis(boronic ester) (416 mg, 92%, >95:5 d.r.) as a colorless oil.

TLC: R_f = 0.78 (pentane:EtOAc 20:1)

^{1}H NMR: (400 MHz, $CDCl_3$) δ 5.89 (ddd, *J* = 17.2, 10.2, 7.8 Hz, 1H, C*H*=CH_2), 5.08 (ddd, *J* = 17.2, 2.0, 0.9 Hz, 1H, CH=CH^aH^b), 4.99 (ddd, *J* = 10.2, 2.0, 0.9 Hz, 1H, CH=CH^aH^b), 4.08 (t, *J* = 7.8 Hz, 1H, CHOTES), 1.34 (ddd, *J* = 11.9, 7.8, 4.7 Hz, 1H, CHBpin), 1.22 (s, 24H, Bpin), 1.00 (dd, *J* = 15.9, 4.7 Hz, 1H, CH^aH^bBpin), 0.93 (t, *J* = 7.8 Hz, 9H, OSi(CH_2CH_3)$_3$), 0.75 (dd, *J* = 15.9, 11.9 Hz, 1H, CH^aH^bBpin), 0.57 (q, *J* = 7.8 Hz, 6H, OSi(CH_2CH_3)$_3$) ppm

^{13}C NMR (101 MHz, $CDCl_3$) δ 141.8 (H_2C=*C*H), 114.4 (H_2*C*=CH), 83.0 (4×*C*(CH_3)$_2$), 77.4 (CHOTES), 25.1 (C(*C*H_3)$_2$), 25.1 (C(*C*H_3)$_2$), 24.9 (C(*C*H_3)$_2$), 24.9 (C(*C*H_3)$_2$), 7.1 (OSi(CH_2*C*H_3)$_3$), 5.2 (OSi(*C*H_2CH_3)$_3$) ppm

HRMS (*m*/*z*): (ESI) calc'd for $C_{23}H_{46}B_2O_5SiNa$ $[M+Na]^+$: 475.3202, found: 475.3182

IR (neat) ν_{max}: 2978, 2956, 2877, 1370, 1315, 1141, 834 and 739 cm^{-1}

$[\alpha]_D^{20}$**:** –3.3 (*c* = 1.0, $CHCl_3$)

{[(3*S*,4*S*)-4,5-Bis(4,4,5,5-tetramethyl-1,3,2-dioxaborolan-2-yl)pent-1-en-3-yl]oxy}triisopropylsilane (65)

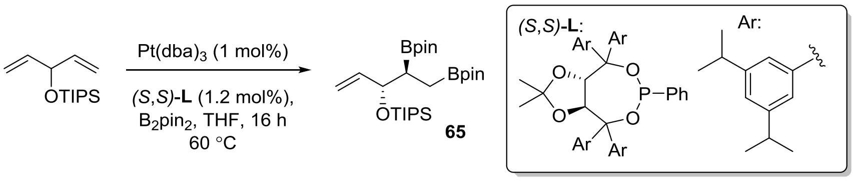

65 was synthesised according to general procedure A, with **25c** as the diene, and purified by flash column chromatography (SiO_2; pentane:EtOAc 20:1) to yield the desired 1,2-bis(boronic ester) (470 mg, 95%, >95:5 d.r.) as a colorless oil.

TLC: R_f = 0.53 (pentane:EtOAc 20:1)

^{1}H NMR: (400 MHz, $CDCl_3$) δ 5.94 (ddd, J = 16.7, 10.1, 8.5 Hz, 1H, CH=CH_2), 5.05 – 4.98 (m, 2H, CH=CH_2), 4.21 (t, J = 7.4 Hz, 1H, CHOTIPS), 1.49 (dt, J = 10.1, 6.4 Hz, 1H, CHBpin), 1.21 (s, 24H, Bpin), 1.04 (s, 18H, OTIPS), 1.02 – 0.96 (m, 1H, C$H^a$$H^b$Bpin), 0.80 (dd, J = 16.3, 8.3 Hz, 1H, C$H^a$$H^b$Bpin) ppm

^{13}C NMR (101 MHz, $CDCl_3$) δ 141.5 (H_2C=CH), 114.8 ($H_2$$C$=CH), 83.0 (4×$C$($CH_3$)$_2$), 78.5 (CHOTIPS), 25.3 (C($C$$H_3$)$_2$), 25.1 (C($C$$H_3$)$_2$), 24.9 (C($C$$H_3$)$_2$), 24.8 (C($C$$H_3$)$_2$), 18.4 (CH($C$$H_3$)$_2$)$_3$), 18.3 (OSi(CH($C$$H_3$)$_2$)$_3$), 12.7 (OSi($C$H($CH_3$)$_2$)$_3$) ppm

HRMS (*m/z*): (ESI) calc'd for $C_{26}H_{52}B_2O_5SiNa$ $[M+Na]^+$: 517.3672, found: 517.3663

IR (neat) ν_{max}: 2977, 2942, 2866, 1370, 1315, 1139 and 1058 cm^{-1}

$[\alpha]_D^{20}$**:** +3.3 (c = 0.9, $CHCl_3$)

{[(3*S*,4*S*)-4,5-Bis(4,4,5,5-tetramethyl-1,3,2-dioxaborolan-2-yl)pent-1-en-3-yl]oxy}(tert-butyl)diphenylsilane (66)

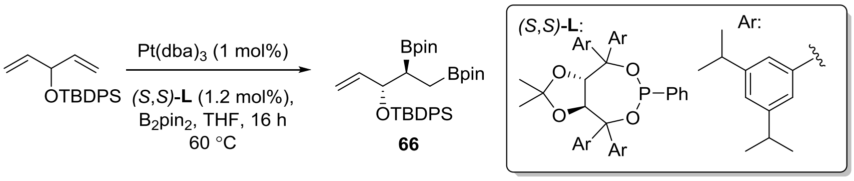

66 was synthesised according to general procedure A, with **25e** as the diene, and purified by flash column chromatography (SiO_2; pentane:EtOAc 20:1) to yield the desired 1,2-bis(boronic ester) (525 mg, 91%, >95:5 d.r.) as a colorless oil.

TLC: R_f = 0.41 (pentane:EtOAc 20:1)

^{1}H NMR: (400 MHz, $CDCl_3$) δ 7.74 – 7.66 (m, 4H, ArH), 7.41 – 7.29 (m, 6H, ArH), 5.87 (ddd, J = 17.3, 10.2, 8.4 Hz, 1H, CH=CH_2), 4.76 (dd, J = 10.2, 1.9 Hz, 1H, CH=CH^aH^b), 4.58 (ddd, J = 17.3, 1.9 Hz, 1H, CH=CH^aH^b), 4.16 – 4.10 (m, 1H, CHOTBDPS), 1.49 (dt, J = 10.1, 6.4 Hz, 1H, CHBpin), 1.23 (s, 6H, Bpin), 1.24 (s, 6H, Bpin), 1.18 (s, 6H, Bpin), 1.17 (s, 6H, Bpin), 1.04 (s, 9H, OTBDPS), 0.90 – 0.86 (m, 1H, CH_2Bpin), 0.79 – 0.74 (m, 1H, CH_2Bpin) ppm

^{13}C NMR (101 MHz, $CDCl_3$) δ 140.0 (H_2C=CH), 136.3 (ArCH), 136.3 (ArCH), 135.1 (ArC), 134.7 (ArC), 129.4 (ArCH), 129.3 (ArCH), 127.4 (ArCH), 127.2 (ArCH), 115.7 ($H_2$$C$=CH), 83.0 (4×$C$($CH_3$)$_2$), 79.4 (CHOTBDPS), 27.2 (OSi(C(CH$_3$)$_3$Ph$_2$), 25.3 (C(CH$_3$)$_2$), 25.0 (C(CH$_3$)$_2$), 24.9 (C(CH$_3$)$_2$), 24.8 (C(CH$_3$)$_2$), 19.6 (OSi(C(CH$_3$)$_3$Ph$_2$) ppm

HRMS (*m/z*): (ESI) calc'd for $C_{33}H_{50}B_2O_5SiNa$ [M+Na]$^+$: 599.3518, found: 599.3506

IR (neat) ν_{max}: 2977, 2930, 1353, 1315, 1137, 1059, 846 and 701 cm^{-1}

$[\alpha]_D^{20}$: +17.1 (c = 0.9, $CHCl_3$)

2,2'-[(2*S*,3*S*)-3-(Benzyloxy)pent-4-ene-1,2-diyl]bis(4,4,5,5-tetramethyl-1,3,2-dioxaborolane) (67)

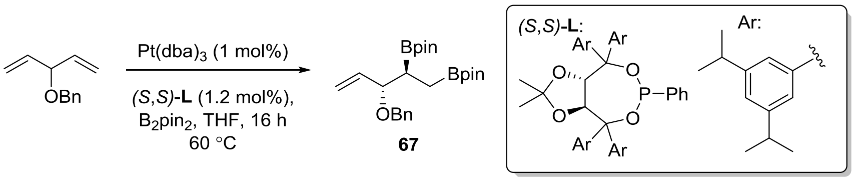

67 was synthesised according to general procedure A, with **25g** as the diene, but heated at 80 °C (oil bath) for 16 hr. Purification by flash column chromatography (SiO_2; pentane:EtOAc 20:1) yielded the desired 1,2-bis(boronic ester) (94 mg, 22%, >95:5 d.r.) as a colorless oil.

TLC: R_f = 0.48 (pentane:EtOAc 10:1)

^{1}H NMR: (400 MHz, $CDCl_3$) δ 7.36 – 7.19 (m, 5H, ArH), 5.79 (ddd, J = 17.7, 9.5, 8.0 Hz, 1H, CH=CH_2), 5.20 (s, 1H, CH=CH^aH^b), 5.18 – 5.15 (m, 1H, CH=CH^aH^b), 4.55 (d, J = 12.0 Hz, 1H, CH^aH^bPh), 4.34 (d, J = 12.0 Hz, 1H, CH^aH^bPh), 3.80 (t, J = 8.0 Hz, 1H, CHOBn), 1.49 (ddd, J = 10.7, 8.0, 5.0 Hz, 1H, CHBpin), 1.20 (s, 24H, Bpin), 1.11 (dd, J = 16.0, 5.0 Hz, 1H, CH^aH^bBpin), 0.85 (dd, J = 16.0, 10.7 Hz, 1H, CH^aH^bBpin) ppm

^{13}C NMR: (101 MHz, $CDCl_3$) δ 139.3 (ArC), 138.8 ($H_2C{=}CH$), 128.2 (ArCH), 127.8 (ArCH), 127.2 (ArCH), 117.3 ($H_2C{=}CH$), 83.3 (CHOBn), 83.1 (2×$C(CH_3)_2$), 83.0 (2×$C(CH_3)_2$), 70.1 (CH_2Ph), 25.1 ($C(CH_3)_2$), 25.0 ($C(CH_3)_2$), 24.9 ($C(CH_3)_2$), 24.8 ($C(CH_3)_2$) ppm

HRMS (*m*/*z*): (ESI) calc'd for $C_{24}H_{38}B_2O_5Na$ $[M+Na]^+$: 451.2806, found: 451.2802

IR (neat) ν_{max}: 2978, 2931, 1370, 1317 and 1140 cm^{-1}

$[\alpha]_D^{20}$**:** –11.5 (c = 1.0, $CHCl_3$)

2,2'-[3-(Methoxymethoxy)pent-4-ene-1,2-diyl]bis(4,4,5,5-tetramethyl-1,3,2-dioxaborolane) (68)

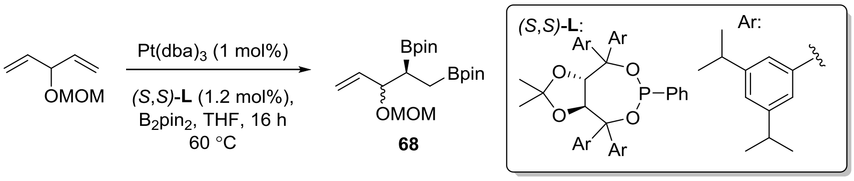

68 was synthesised according to general procedure A, with **25h** as the diene, and purified by flash column chromatography (SiO_2; pentane:EtOAc 20:1) to yield the desired 1,2-bis(boronic ester) (134 mg, 35%, 67:33 d.r.) as a colorless oil.

TLC: R_f = 0.41 (pentane:EtOAc 10:1)

^{1}H NMR: (400 MHz, $CDCl_3$) δ 5.76 – 5.64 (m, 1H, CH=CH$_2$), 5.20 – 5.11 (m, 2H, CH=CH_2), 4.70 – 4.65 (m, 1H, $OCH^aH^bOCH_3$), 4.53 – 4.49 (m, 1H, $OCH^aH^bOCH_3$), 4.05 (t, J = 7.4 Hz, 1H, CHOMOM, *anti*), 3.97 (t, J = 8.5 Hz, 1H, CHOMOM, *syn*), 3.36 (s, 3H, OCH_2OCH_3, *syn*), 3.35 (s, 3H, OCH_2OCH_3, *anti*), 1.49 – 1.40 (m, 1H, CHBpin), 1.22 (s, 24H, Bpin, *syn*), 1.21 (s, 24H, Bpin, *anti*), 1.09 – 1.04 (m, 1H, CH^aH^bBpin), 0.88 – 0.80 (m, 1H, CH^aH^bBpin) ppm

^{13}C NMR: (101 MHz, $CDCl_3$) δ 138.4 ($H_2C{=}CH$, *syn*), 138.1 ($H_2C{=}CH$, *anti*), 117.9 ($H_2C{=}CH$, *anti*), 117.6 ($H_2C{=}CH$, *syn*), 93.9 (OCH_2OCH_3, *anti*), 93.8 (OCH_2OCH_3, *syn*), 83.2 (2×$C(CH_3)_2$, *anti*), 83.2 (2×$C(CH_3)_2$, *syn*), 83.0 (2×$C(CH_3)_2$, *syn*), 83.0 (2×$C(CH_3)_2$, *anti*), 80.9 (CHOMOM, *anti*), 80.3 (CHOMOM, *syn*), 55.8 (OCH_2OCH_3, *syn*), 55.6 (OCH_2OCH_3), 25.1 ($C(CH_3)_2$], *anti*), 25.1 ($C(CH_3)_2$), 25.1 ($C(CH_3)_2$), 25.1 ($C(CH_3)_2$), 24.9 ($C(CH_3)_2$), 24.9 ($C(CH_3)_2$), 24.8 ($C(CH_3)_2$), 24.8 ($C(CH_3)_2$) ppm

HRMS (*m*/*z*): (ESI) calc'd for $C_{19}H_{36}B_2O_6Na$ $[M+Na]^+$: 405.2597, found: 405.2605

IR (neat) ν_{max}: 2978, 2930, 1369, 1313, 1141 and 1032 cm^{-1}

3.3 Proof of absolute stereochemistry of 6d

(2*S*,3*R*)-Pent-4-ene-1,2,3-triol (69)

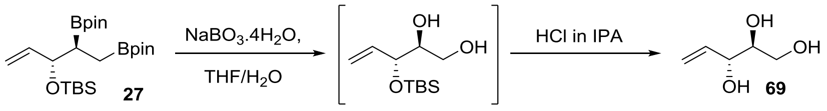

In order to prove the relative stereochemistry, a small sample of 1,2-bis(boronic ester) **27** was transformed into the corresponding triol **69**. Oxidation with $NaBO_3{\cdot}4H_2O$ in THF/H_2O (1:1), followed by deprotection using HCl (in isopropanol) delivered the literature known triol. By comparison of ^{13}C NMR data we were able to prove the *anti*-selectivity of the diboration reaction forming **27**.

^{1}H NMR: (400 MHz, $CDCl_3$) δ 6.02 (ddd, J = 17.0, 10.6, 6.2 Hz, 1H, C*H*=CH$_2$), 5.33 (dd, J = 17.0, 1.7 Hz, 1H, CH=CH^aH^b), 5.21 (dd, J = 10.6, 1.7 Hz, 1H, CH=CH^aH^b), 4.11 – 4.06 (m, 1H, =CHC*H*OH), 3.72 – 3.66 (m, 1H, C*H*(OH)CH$_2$OH), 3.62 – 3.56 (m, 2H, CH(OH)CH_2OH) ppm

^{13}C NMR (101 MHz, $CDCl_3$) δ 139.2 (H$_2$C=*C*H), 116.4 (H$_2$*C*=CH), 76.0 (=CH*C*HOH), 74.9 (*C*H(OH)CH$_2$OH), 64.4 (CH(OH)*C*H$_2$OH) ppm

The ^{13}C NMR data match those reported for the *anti*-diastereoisomer of the triol.[28]

$[\alpha]_D^{20}$**:** +21.2 (c = 0.7, MeOH)

[28] T. Gracza, T. Hasenöhrl, U. Stahl, V. Jäger, *Synthesis* **1991**, 1108.

(2*S*,3*R*)-Pent-4-ene-1,2,3-triyl tris(4-bromobenzoate) (70)

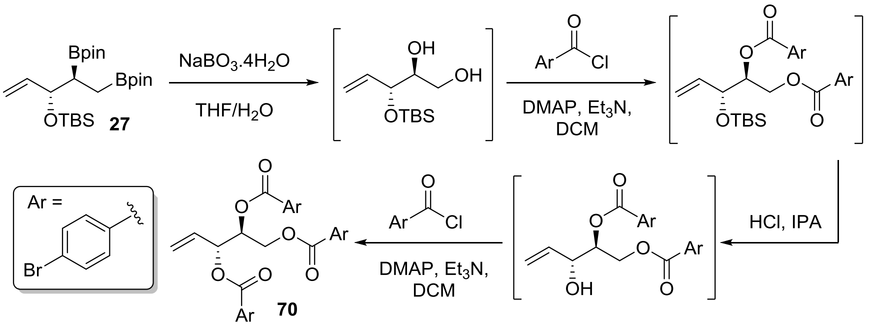

In order to determine the enantiomeric ratio of 1,2-bis(boronic) ester **27**, it was transformed into the corresponding tris-benzoate **70**.

A small sample of the 1,2-bis(boronic ester) was transformed to the corresponding diol by using excess $NaBO_3 \cdot 4H_2O$ in THF/H_2O (1:1). We protected the diol using excess 4-bromobenzoyl chloride (2.5 eq.), triethylamine (3.0 eq.) and DMAP (0.2 eq.) in CH_2Cl_2. The TBS-group was removed using excess HCl in THF and the newly generated hydroxyl group was protected as the benzoate (for reagents see first protection). Purification by flash column chromatography (SiO_2; pentane:EtOAc 20:1) yielded the desired trisbenzoate **70** as a viscous, colorless oil.

TLC: $R_f = 0.50$ (pentane:EtOAc 9:1)

^{1}H NMR: (400 MHz, $CDCl_3$) δ 7.90 – 7.80 (m, 6H, ArH), 7.60 – 7.52 (m, 6H, ArH), 6.03 (ddd, *J* = 17.4, 10.4, 6.4 Hz, 1H, C*H*=CH$_2$), 5.93 (dd, *J* = 6.4, 4.3 Hz, 1H, C*H*CH=CH$_2$), 5.77 (ddd, *J* = 7.3, 4.3, 3.8 Hz, 1H, C*H*CH$_2$), 5.53 (dd, *J* = 17.4, 1.2 Hz, 1H, CH=CH^aH^b), 5.43 (dd, *J* = 10.4, 1.2 Hz, 1H, CH=CH^aH^b), 4.73 (dd, *J* = 12.1, 3.8 Hz, 1H, CH^aH^b), 4.61 (dd, *J* = 7.3, 12.1 Hz, 1H, CH^aH^b) ppm

^{13}C NMR: (101 MHz, $CDCl_3$) δ 165.5 (CO), 165.0 (CO), 164.6 (CO), 131.3 (*C*H=CH$_2$), 132.1 (ArCH), 132.0 (ArCH), 132.0 (ArCH), 131.4 (ArCH), 131.3 (ArCH), 131.3 (ArCH), 128.9 (ArC), 128.8 (ArC), 128.7 (ArC), 128.5 (ArC), 128.4 (ArC), 128.4 (ArC), 120.7 (CH=*C*H$_2$), 73.9 (*C*HCH=CH$_2$), 72.4 (d, *C*HCH$_2$), 62.7 (CH$_2$) ppm

HRMS (*m/z*): (ESI) calc'd for $C_{26}H_{19}Br_3O_6Na$ $[M+Na]^+$: 686.8624, found: 686.8649

IR (neat) ν_{max}: 2967, 1721, 1589, 1255, 1094, 1010 and 752 cm^{-1}

$[\alpha]_D^{20}$**:** –49.1 (*c* = 0.60, $CHCl_3$)

Chiral HPLC: Daicel Chiralpak-IA column (25 cm) with guard, 10.0% isopropanol in hexane, 0.5 mL/min, ambient temperature, 210.8 nm: t_R = 38.5 min (minor), 42.4 min (major), e.r. = 98:2

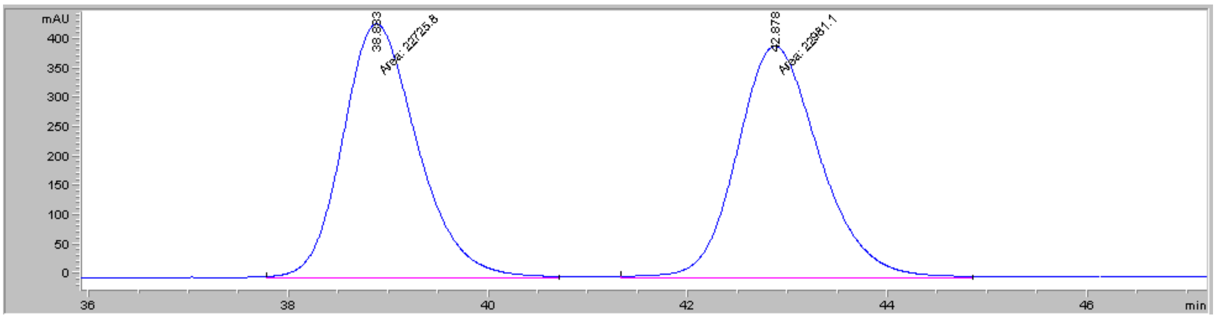

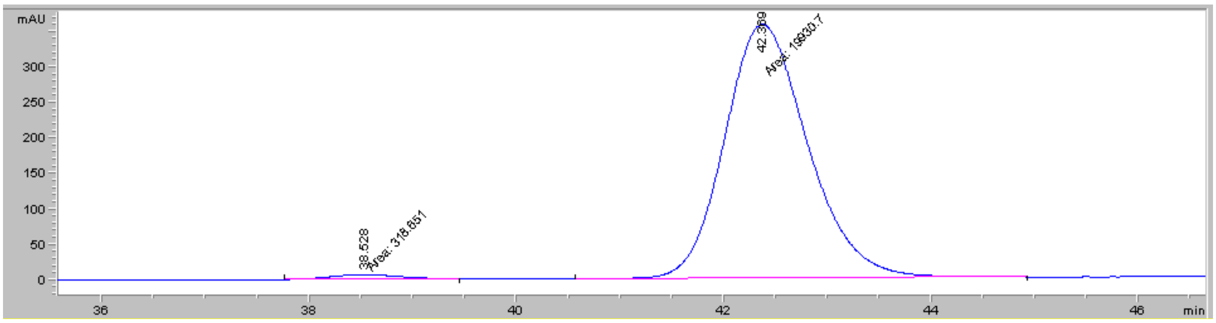

4. Total synthesis of Sch725674

4.1 Synthesis of starting materials

Heptane-1,7-diyl bis(2,4,6-triisopropylbenzoate) (24b)

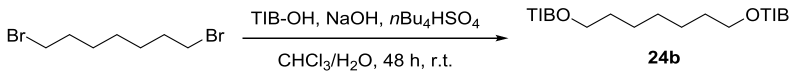

A solution of NaOH (3.2 g, 80 mmol, 4.0 eq.) and *n*Bu_4HSO_4 (679 mg, 2.0 mmol, 0.1 eq.) in H_2O (50 mL) was added to a solution of 2,4,6-triisopropylbenzoic acid (13.1 g, 53 mmol, 2.6 eq.) in $CHCl_3$ (50 mL). The biphasic reaction mixture was stirred vigorously and 1,7-dibromoheptane (3.4 mL, 5.2 g, 20 mmol, 1.0 eq.) was added. The emulsion was stirred for 48 hr until the reaction was complete. The phases were separated and the aqueous phase was extracted with $CHCl_3$ (2×50 mL). The combined organic phases were washed with brine (100 mL), dried over $MgSO_4$, filtered and concentrated under reduced pressure. Purification by flash column chromatography (SiO_2; pentane:EtOAc 95:5) yielded bisbenzoate **24b** (11.7 g, 99%) as a viscous colorless oil.

TLC: R_f = 0.43 (pentane:EtOAc 20:1)

^{1}H NMR: (400 MHz, $CDCl_3$) δ 7.00 (s, 4H, ArH), 4.29 (t, J = 6.6 Hz, 4H, 2×CH_2OTIB), 2.88 (hept, J = 6.8 Hz, 2H, 2×C*H*(CH_3)$_2$), 2.84 (hept, J = 6.8 Hz, 4H, 4×C*H*(CH_3)$_2$), 1.77 – 1.69 (m, 4H, 2×CH_2), 1.47 – 1.38 (m, 6H, 3×CH_2), 1.24 (d, J = 6.8 Hz, 12H, 2×CH(CH_3)$_2$), 1.24 (d, J = 6.8 Hz, 24H, 4×CH(CH_3)$_2$) ppm

^{13}C NMR (101 MHz, $CDCl_3$) δ 171.2 (C=O), 150.2 (ArC), 144.8 (ArC), 130.8 (ArC), 121.0 (ArCH), 65.1 (CH_2OTIB), 34.6 (2×*C*H(CH_3)$_2$), 31.6 (4×*C*H(CH_3)$_2$, 29.1 (CH_2), 28.7 (2×CH_2), 26.2 (2×CH_2), 24.3 (4×CH(*C*H_3)$_2$), 24.1 (2×CH(*C*H_3)$_2$) ppm

HRMS (*m*/*z*): (ESI) calc'd for $C_{39}H_{60}O_4Na$ $[M+Na]^+$: 615.4384, found: 615.4396

IR (neat) ν_{max}: 2960, 1723, 1250 and 1075 cm^{-1}

4,4,5,5-Tetramethyl-2-pentyl-1,3,2-dioxaborolane (23)

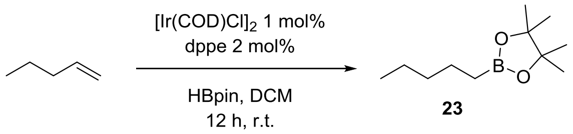

$[Ir(COD)Cl]_2$ (48 mg, 0.07 mmol, 1 mol%) and dppe (57 mg, 0.14 mmol, 2 mol%) were added to a flame-dried Schenk-flask. The flask was purged three times with N_2 before dichloromethane (16 mL), pinacol borane (1.04 mL, 7.17 mmol, 1.00 eq.) and 1-pentene (1.06 mL, 9.69 mmol, 1.35 eq.) were added. After the reaction was stirred for 12 hr at ambient temperature, the solvent was carefully removed under reduced pressure and the crude product was purified by flash column chromatography (SiO_2; pentane). Due to its high volatility the desired boronic ester **23** (corrected mass: 1.31 g, 92%) was obtained as a mixture with pentane.

TLC: R_f = 0.41 (pentane:Et_2O 98:2)

^{1}H NMR: (400 MHz, $CDCl_3$) δ 1.44 – 1.36 (m, 3H, CH_2), 1.32 – 1.24 (m, 5H, CH_2), 1.24 (s, 12H, Bpin), 0.76 (t, *J* = 7.8 Hz, 3H, CH_3) ppm

^{13}C NMR (101 MHz, $CDCl_3$) δ 83.0 (2×*C*(CH_3)$_2$), 34.9 (CH_2), 25.0 (2×C(*C*H_3)$_2$), 23.8 (CH_2), 22.6 (CH_2), 14.2 (CH_3) ppm

HRMS (*m*/*z*): (ESI) calc'd for $C_{11}H_{23}BO_2Na$ $[M+Na]^+$: 221.1685, found: 221.1691

IR (neat) ν_{max}: 2927, 1371, 1315 and 1145 cm^{-1}

3.2 Linear synthesis of Sch725674

(7*R*,9*S*,10*R*)-7,9,10-Tris[(*tert*-butyldimethylsilyl)oxy]dodec-11-en-1-yl 2,4,6-triisopropylbenzoate (28)

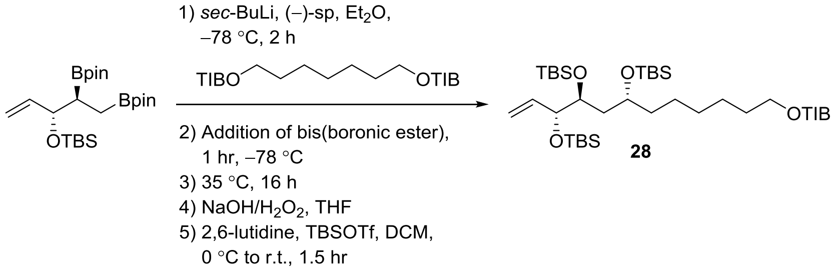

sec-BuLi (1.2 M, 2.2 mL, 2.65 mmol, 1.2 eq.) was added dropwise to a solution of TIB-ester **24b** (1.70 g, 2.87 mmol, 1.3 eq.) and (–)-sparteine (0.66 mL, 2.87 mmol, 1.3 eq.) in anhydrous Et_2O (2 mL) at –78 °C (dry ice/acetone). The purple solution was stirred at –78 °C for 2 hr before the dropwise addition of bisboronic ester **27** (1.00 g, 2.21 mmol, 1.00 eq.) in anhydrous Et_2O (0.5 mL). The reaction mixture was stirred for 1 hr at –78 °C. After warming the solution to ambient temperature, the flask was sealed and heated at 35 °C (oil bath) for 16 hr. The reaction was stopped by the addition of HCl (1 M, 10 mL). The phases were separated and the aqueous phase was extracted with Et_2O (3×10 mL). The combined organic phases were washed with brine (25 mL), dried over $MgSO_4$, filtered and concentrated under reduced pressure. The crude product was dissolved in THF (20 mL) and a few crystals of BHT were added. A 2:1 v:v mixture of 2 M aqueous NaOH (20 mL) and 30% aqueous H_2O_2 (10 mL) was prepared at 0 °C (ice/water), and this was slowly added to the organic solution at 0 °C (ice/water). The reaction was allowed to warm to ambient temperature and was stirred for 1 hr. The mixture was diluted with H_2O (20 mL) and extracted with Et_2O (3×20 mL). The combined organic phases were washed with brine (50 mL), dried over $MgSO_4$ and concentrated under reduced pressure. Filtration over a short plug of silica (pentane:EtOAc 4:1) gave the crude diol. The diol and 2,6-lutidine (1.5 mL, 13.0 mmol, 6.2 eq.) were dissolved in anhydrous dichloromethane (22 mL) and the solution was cooled to 0 °C (ice/water). TBSOTf (2.0 mL, 8.7 mmol, 4.1 eq.) was then added. After warming to ambient temperature, the mixture was stirred for 1.5 hr then quenched by addition of H_2O (20 mL). The aqueous phase was extracted with CH_2Cl_2 (3×20 mL) and the combined organic phases were washed with brine (50 mL), dried over $MgSO_4$ and concentrated under reduced pressure. The crude residue was purified by flash column chromatography (SiO_2; pentane:Et_2O 98:2) to yield the protected 1,3-diol **28** (1.24 g, 70%) as a colorless oil.

TLC: R_f = 0.46 (pentane:Et_2O 98:2)

^{1}H NMR: (500 MHz, $CDCl_3$) δ 7.00 (s, 2H, ArH), 5.83 (ddd, *J* = 17.4, 10.5, 7.1 Hz, 1H, C*H*=CH_2), 5.16 – 5.10 (m, 2H, CH=C*H*$_2$), 4.29 (t, *J* = 6.7 Hz, 2H, CH_2OTIB), 3.95 – 3.92 (m, 1H, CHC*H*(OTBS)CH(OTBS)), 3.84 – 3.80 (m, 1H, CH(OTBS)C*H*(OTBS)CH_2), 3.79 – 3.74 (m, 1H, CH_2C*H*(OTBS)CH_2), 2.89 (hept, *J* = 6.9 Hz, 1H, C*H*(CH_3)$_2$), 2.85 (hept, *J* = 6.9 Hz, 2H, 2×C*H*(CH_3)$_2$), 1.72 (p, *J* = 6.9 Hz, CH_2), 1.52 – 1.28 (m, 10H, 5×CH_2), 1.24 (d, *J* = 6.9 Hz, 18H, 3×CH(C*H*$_3$)$_2$), 0.89 (s, 9H, OSi(CH_3)$_2$C(C*H*$_3$)$_3$), 0.87 (s, 9H, OSi(CH_3)$_2$C(C*H*$_3$)$_3$), 0.87 (s, 9H, OSi(CH_3)$_2$C(C*H*$_3$)$_3$), 0.10 (s, 3H, OSi(C*H*$_3$)$_2$^{t}Bu), 0.07 (s, 3H, OSi(C*H*$_3$)$_2$^{t}Bu), 0.05 (s, 3H, OSi(C*H*$_3$)$_2$^{t}Bu), 0.05 (s, 3H, OSi(C*H*$_3$)$_2$^{t}Bu), 0.03 (s, 3H, OSi(C*H*$_3$)$_2$^{t}Bu), 0.03 (s, 3H, OSi(C*H*$_3$)$_2$^{t}Bu) ppm

^{13}C NMR (126 MHz, $CDCl_3$) δ 171.2 (C=O), 150.2 (ArC), 144.8 (ArC), 138.0 (H_2C=*C*H), 130.9 (ArC), 121.0 (ArCH), 116.2 (H_2*C*=CH), 79.3 (CH*C*H(OTBS)CH(OTBS)), 74.6 (CH(OTBS)*C*H(OTBS)CH_2), 70.1 (CH_2*C*H(OTBS)CH_2), 65.2 (CH_2OTIB, 42.4 (CH_2), 38.3 (CH_2), 34.6 (*C*H(CH_3)$_2$), 29.7 (CH_2), 28.8 (CH_2), 26.0 (CH_2), 26.2 (OSi(CH_3)$_2$C(*C*H$_3$)$_3$), 26.2 (OSi(CH_3)$_2$C(*C*H$_3$)$_3$), 26.1 (OSi(CH_3)$_2$C(*C*H$_3$)$_3$), 25.1 (CH_2), 24.3 (2×CH(*C*H$_3$)$_2$), 24.1 (CH(*C*H$_3$)$_2$), 18.6 (OSi(CH_3)$_2$*C*(CH_3)$_3$), 18.5 (OSi(CH_3)$_2$*C*(CH_3)$_3$), 18.3 (OSi(CH_3)$_2$*C*(CH_3)$_3$), -3.3 (OSi(*C*H$_3$)$_2$^{t}Bu), -3.4 (OSi(*C*H$_3$)$_2$^{t}Bu), -3.9 (OSi(*C*H$_3$)$_2$^{t}Bu), -4.1 (OSi(*C*H$_3$)$_2$^{t}Bu), -4.4 (OSi(*C*H$_3$)$_2$^{t}Bu), -4.5 (OSi(*C*H$_3$)$_2$^{t}Bu) ppm

HRMS (*m*/*z*): (MALDI) calc'd for $C_{46}H_{88}O_5Si_3Na$ [M+Na]$^+$: 827.5832, found: 827.5838

IR (neat) ν_{max}: 2957, 2929, 2857, 1728, 1250, 1075, 833 and 773 cm^{-1}

$[\alpha]_D^{20}$**:** –4.1 (*c* = 0.79, $CHCl_3$)

(6*R*,12*R*,14*S*,15*R*)-12,14,15-Tris[(*tert*-butyldimethylsilyl)oxy]heptadec-16-en-6-ol (29)

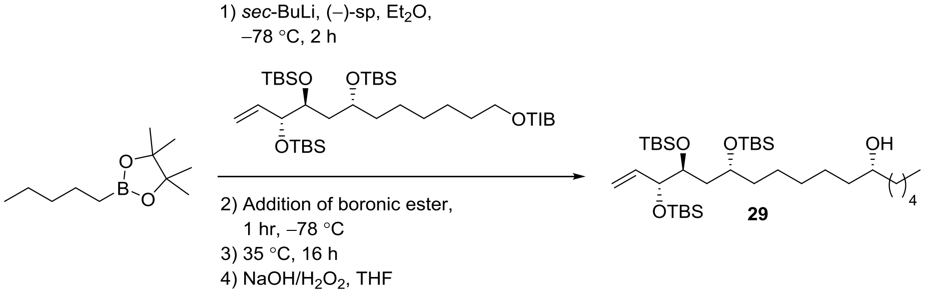

sec-BuLi (1.3 M, 3.4 mL, 4.4 mmol, 1.2 eq.) was added dropwise to a solution of TIB-ester **28** (3.00 g, 3.72 mmol, 1.00 eq.) and (+)-sparteine (1.11 mL, 4.84 mmol, 1.30 eq.) in anhydrous Et_2O (19 mL) at –78 °C (dry ice/acetone). The solution was stirred at –78 °C for 2 hr before the dropwise addition of boronic ester **23** (1.03 g, 5.21 mmol, 1.40 eq.) in anhydrous Et_2O (3.0 mL). The reaction mixture was stirred for 1 hr at –78 °C. After warming the solution to ambient temperature, the flask was sealed and heated at 35 °C (oil bath) for 16 hr. The reaction was stopped by the addition of HCl (1 M, 10 mL). The phases were separated and the aqueous phase was extracted with Et_2O (3×10 mL). The combined organic phases were washed with brine (25 mL), dried over $MgSO_4$, filtered and concentrated under reduced pressure. The crude product was dissolved in THF (50 mL) and a few crystals of BHT were added. A 2:1 v:v mixture of 2 M aqueous NaOH (50 mL) and 30% aqueous H_2O_2 (25 mL) was prepared at 0 °C (ice/water), and this was slowly added to the organic solution at 0 °C (ice/water). The reaction was allowed to warm to ambient temperature and was stirred for 1 hr. The mixture was diluted with H_2O (25 mL) and extracted with Et_2O (3×50 mL). The combined organic phases were washed with brine (100 mL), dried over $MgSO_4$ and concentrated under reduced pressure. The crude reside was purified by flash column chromatography (SiO_2; pentane:Et_2O 96:4) to yield alcohol **29** (2.04 g, 85%) as a colorless oil.

TLC: R_f = 0.21 (pentane:Et_2O 96:4)

^{1}H NMR: (400 MHz, $CDCl_3$) δ 5.83 (ddd, J = 17.4, 10.7, 6.9 Hz, 1H, C*H*=CH_2), 5.16 – 5.10 (m, 2H, CH=CH_2), 3.96 – 3.92 (m, 1H, CHC*H*(OTBS)CH(OTBS)), 3.84 – 3.80 (m, 1H, CH(OTBS)C*H*(OTBS)CH_2), 3.79 – 3.74 (m, 1H, CH_2C*H*(OTBS)CH_2), 3.61 – 3.54 (m, 1H, C*H*OH), 1.52 – 1.25 (m, 20H, 10×CH_2), 0.90 (s, 9H, $OSi(CH_3)_2C(CH_3)_3$), 0.88 (s, 9H, $OSi(CH_3)_2C(CH_3)_3$), 0.87 (s, 9H, $OSi(CH_3)_2C(CH_3)_3$), 0.90 – 0.86 (m, 3H, CH_3), 0.10 (s, 3H, $OSi(CH_3)_2{}^t$Bu), 0.08 (s, 3H, $OSi(CH_3)_2{}^t$Bu), 0.05 (s, 3H, $OSi(CH_3)_2{}^t$Bu), 0.05 (s, 3H, $OSi(CH_3)_2{}^t$Bu), 0.04 (s, 3H, $OSi(CH_3)_2{}^t$Bu), 0.03 (s, 3H, $OSi(CH_3)_2{}^t$Bu) ppm

^{13}C NMR (101 MHz, $CDCl_3$) δ 138.0 (H_2C=*C*H), 116.2 (H_2*C*=CH), 79.3 (CH*C*H(OTBS)CH(OTBS), 74.6 (CH(OTBS)*C*H(OTBS)CH_2), 72.1 (CHOH), 70.2 (CH_2*C*H(OTBS)CH_2), 42.4 (CH_2), 38.3 (CH_2), 37.6 (CH_2), 37.6 (CH_2), 32.1 (CH_2), 30.0 (CH_2), 26.2 (OSi(CH_3)$_2$C(CH_3)$_3$), 26.2 (OSi(CH_3)$_2$C(CH_3)$_3$), 26.1 (OSi(CH_3)$_2$C(CH_3)$_3$), 25.8 (CH_2), 25.5 (CH_2), 25.2 (CH_2), 22.8 (CH_2), 18.6 (OSi(CH_3)$_2$*C*(CH_3)$_3$), 18.5 (OSi(CH_3)$_2$*C*(CH_3)$_3$), 18.3 (s, OSi(CH_3)$_2$*C*(CH_3)$_3$), 14.2 (CH_3), -3.4 (OSi(CH_3)$_2$^{t}Bu), -3.4 (OSi(CH_3)$_2$^{t}Bu), -3.9 (OSi(CH_3)$_2$^{t}Bu), -4.1 (OSi(CH_3)$_2$^{t}Bu), -4.4 (OSi(CH_3)$_2$^{t}Bu), -4.4 (OSi(CH_3)$_2$^{t}Bu) ppm

HRMS (*m/z*): (ESI) calc'd for $C_{35}H_{76}O_4Si_3Na$ $[M+Na]^+$: 667.4944, found: 667.4963

IR (neat) ν_{max}: 3360, 2929, 2857, 1252, 1067, 832 and 772 cm^{-1}

$[\alpha]_D^{20}$**:** –7.1 (*c* = 1.02, $CHCl_3$)

(4*R*,5*S*,7*R*,13*R*,*E*)-Methyl 4,5,7-tris[(tert-butyldimethylsilyl)oxy]-13-hydroxyoctadec-2-enoate (71)

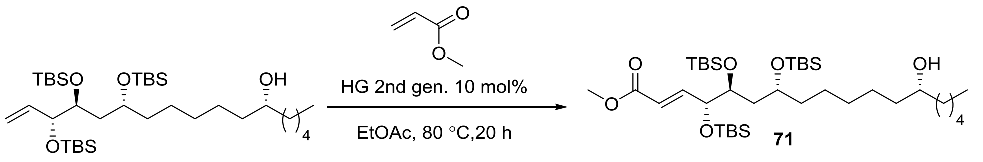

Olefin **29** (150 mg, 0.23 mmol, 1.0 eq.) was dissolved in a flame-dried Schlenk-tube in anhydrous ethyl acetate (0.6 mL) and nitrogen was bubbled through the solution for 5 min. Methyl acrylate (0.07 mL, 0.69 mmol, 3.00 eq.) was then added. The solution was heated to 80 °C (oil bath). In a separate pear-shaped flask Hoveyda-Grubbs Catalyst 2nd Generation (14.6 mg, 23 µmol, 10 mol%) was dissolved in anhydrous ethyl acetate (1.3 mL) and nitrogen was bubbled through the solution for 5 min. The catalyst was added dropwise over 19 hr to the olefin solution *via* syringe pump. After addition, the solution was stirred for another hour at 80 °C before it was allowed to cool to ambient temperature. The solvent was removed under reduced pressure and the residue was purified directly by flash column chromatography (SiO_2; pentane:EtOAc 94:6 to 90:10) to yield the α,β-unsaturated ester **71** (109 mg, 67%) as a colorless oil.

TLC: R_f = 0.44 (pentane:EtOAc 90:10)

^{1}H NMR: (400 MHz, $CDCl_3$) δ 6.94 (dd, *J* = 15.7, 5.3 Hz, 1H, MeO_2CCH=C*H*), 5.98 (dd, *J* = 15.7, 1.6 Hz, MeO_2CC*H*=CH), 4.16 (m, 1H, CHC*H*(OTBS)CH(OTBS)), 3.87 (dt, *J* = 6.9,

3.1 Hz, 1H, CH(OTBS)C*H*(OTBS)CH_2), 3.74 (s, 3H, CO_2CH_3), 3.77 – 3.72 (m, 1H, CH_2C*H*(OTBS)CH_2), 3.61 – 3.54 (m, 1H, C*H*OH), 1.58 – 1.22 (m, 20H, 10×CH_2), 0.91 (s, 9H, $OSi(CH_3)_2C(CH_3)_3$), 0.91 – 0.86 (m, 3H, CH_3), 0.86 (s, 18H, 2× $OSi(CH_3)_2C(CH_3)_3$), 0.09 (s, 3H, $OSi(CH_3)_2{}^t$Bu), 0.07 (s, 3H, $OSi(CH_3)_2{}^t$Bu), 0.07 (s, 3H, $OSi(CH_3)_2{}^t$Bu), 0.05 (s, 3H, $OSi(CH_3)_2{}^t$Bu), 0.04 (s, 6H, 2× $OSi(CH_3)_2{}^t$Bu) ppm

^{13}C NMR (101 MHz, $CDCl_3$) δ 166.9 (*C*O_2CH_3), 148.2 (O_2CC=*C*H), 121.6 (O_2C*C*=CH), 77.3 (CH*C*H(OTBS)CH(OTBS), 74.3 (CH(OTBS)*C*H(OTBS)CH_2), 72.1 (CHOH), 70.0 (CH_2*C*H(OTBS)CH_2), 51.7 (CO_2*C*H_3), 42.4 (CH_2), 38.3 (CH_2), 37.6 (CH_2), 37.6 (CH_2), 32.1 (CH_2), 30.0 (CH_2), 26.2 ($OSi(CH_3)_2C(CH_3)_3$), 26.1 ($OSi(CH_3)_2C(CH_3)_3$), 26.1($OSi(CH_3)_2C(CH_3)_3$), 25.8 (CH_2), 25.5 (CH_2), 25.1 (CH_2), 22.8 (CH_2), 18.6 ($OSi(CH_3)_2C(CH_3)_3$), 18.4 ($OSi(CH_3)_2C(CH_3)_3$), 18.2 ($OSi(CH_3)_2C(CH_3)_3$), 14.2 (CH_3), -3.4 ($OSi(CH_3)_2{}^t$Bu), -3.4 ($OSi(CH_3)_2{}^t$Bu), -3.9 ($OSi(CH_3)_2{}^t$Bu), -4.3 ($OSi(CH_3)_2{}^t$Bu), -4.4 ($OSi(CH_3)_2{}^t$Bu), -4.5 ($OSi(CH_3)_2{}^t$Bu) ppm

HRMS (*m*/*z*): (ESI) calc'd for $C_{37}H_{78}O_6Si_3Na$ $[M+Na]^+$: 725.4998, found: 725.4988

IR (neat) ν_{max}: 2929, 2857, 1731, 1253, 1087, 832 and 773 cm^{-1}

$[\alpha]_D^{20}$: –11.6 (*c* = 0.69 $CHCl_3$)

(4*R*,5*S*,7*R*,13*R*,*E*)-4,5,7-Tris[(tert-butyldimethylsilyl)oxy]-13-hydroxyoctadec-2-enoic acid (30)

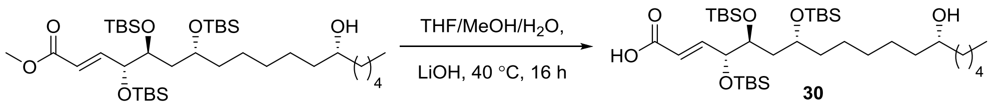

$LiOH{\cdot}H_2O$ (603 mg, 14.4 mmol, 10.0 eq.) was added to a solution of ester **71** (1.01 g, 1.44 mmol, 1.0 eq.) in a THF-MeOH-Water mixture (1:1:1, 30 mL each), which was subsequently heated at 40 °C (oil bath) for 16 hr. After addition of H_2O (50 mL) and HCl (1 M, 50 mL), the reaction was extracted with Et_2O (3×100 mL). The combined organic phases were washed with brine (100 mL), dried over $MgSO_4$, filtered and concentrated under reduced pressure to give acid **30** (0.98 g, 99%) as a colorless, viscous oil. The product was used in the next step without further purification.

TLC: R_f = 0.67 (pentane:EtOAc 70:30)

^{1}**H NMR:** (400 MHz, $CDCl_3$) δ 7.04 (dd, J = 15.6, 5.1 Hz, 1H, $HO_2CCH{=}CH$), 5.99 (dd, J = 15.4, 1.3 Hz, $HO_2CCH{=}CH$), 4.20 (m, 1H, CHC*H*(OTBS)CH(OTBS)), 3.92 – 3.86 (m, 1H, CH(OTBS)C*H*(OTBS)CH_2), 3.78 – 3.72 (m, 1H, CH_2CH(OTBS)CH_2), 1.65 – 1.20 (m, 20H, 10×CH_2), 3.63 – 3.55 (m, 1H, C*H*OH), 0.92 (s, 9H, $OSi(CH_3)_2C(CH_3)_3$), 0.92 – 0.87 (m, 3H, CH_3), 0.87 (s, 18H, 2× $OSi(CH_3)_2C(CH_3)_3$), 0.09 (s, 3H, $OSi(CH_3)_2{}^t$Bu), 0.08 (s, 6H, 2× $OSi(CH_3)_2{}^t$Bu), 0.06 (s, 3H, $OSi(CH_3)_2{}^t$Bu), 0.05 (s, 3H, $OSi(CH_3)_2{}^t$Bu), 0.05 (s, 3H, $OSi(CH_3)_2{}^t$Bu) ppm

^{13}**C NMR** (101 MHz, $CDCl_3$) δ 171.2 (CO_2H), 150.5 ($O_2CC{=}CH$), 121.3 ($O_2CC{=}CH$), 77.3 (CHCH(OTBS)CH(OTBS)), 74.3 (CH(OTBS)CH(OTBS)CH_2), 72.2 (CHOH), 70.0 (CH_2CH(OTBS)CH_2), 42.5 (CH_2), 38.2 (CH_2), 37.5 (CH_2), 37.5 (CH_2), 32.1 (CH_2), 29.9 (CH_2), 26.2 ($OSi(CH_3)_2C(CH_3)_3$), 26.1 ($OSi(CH_3)_2C(CH_3)_3$), 26.1 ($OSi(CH_3)_2C(CH_3)_3$), 25.8 (CH_2), 25.5 (CH_2), 25.2 (CH_2), 22.8 (CH_2), 18.6 ($OSi(CH_3)_2C(CH_3)_3$), 18.4 ($OSi(CH_3)_2C(CH_3)_3$), 18.2 ($OSi(CH_3)_2C(CH_3)_3$), 14.2 (CH_3), -3.4 ($OSi(CH_3)_2{}^t$Bu), -3.5 ($OSi(CH_3)_2{}^t$Bu), -3.9 ($OSi(CH_3)_2{}^t$Bu), -4.3 ($OSi(CH_3)_2{}^t$Bu), -4.4 ($OSi(CH_3)_2{}^t$Bu), -4.5 ($OSi(CH_3)_2{}^t$Bu) ppm

HRMS (*m/z*): (ESI) calc'd for $C_{36}H_{76}O_6Si_3Na$ $[M+Na]^+$: 711.4842, found: 711.4865

IR (neat) ν_{max}: 2929, 2857, 1701, 1253, 1073, 832 and 773 cm^{-1}

$[\alpha]_D^{20}$**:** –13.5 (c = 0.46, $CHCl_3$)

(5*R*,6*S*,8*R*,14*R*,*E*)-5,6,8-Tris[(tert-butyldimethylsilyl)oxy]-14-pentyloxacyclotetradec-3-en-2-one (30)

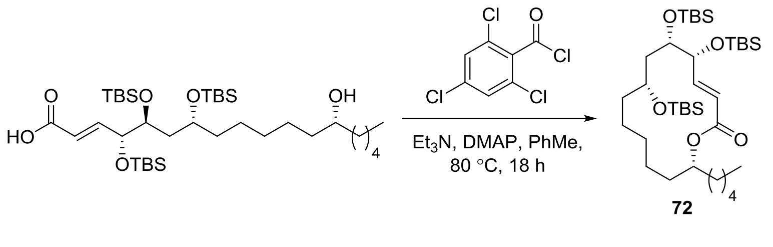

Acid **71** (977 mg, 1.42 mmol, 1.00 eq.) and triethylamine (0.60 mL, 4.25 mmol, 3.00 eq.) were dissolved in anhydrous toluene (16 mL). The solution was cooled to 0 °C (ice/water) and 2,4,6-trichlorobenzoylchloride (0.27 mL, 1.70 mmol, 1.20 eq.) was added in one portion. After 5 min the reaction was warmed to ambient temperature and stirred for 4 hr to form the mixed anhydride. The solution of the anhydride was further diluted with anhydrous toluene (6 mL) and added dropwise to a solution of 4-dimethylaminopyridine (347 mg, 2.84 mmol, 2.00 eq.)

in anhydrous toluene (500 mL) at 80 °C (oil bath) over 16 hr *via* syringe pump. After complete addition the mixture was stirred for 2 hr at 80 °C before it was allowed to cool to ambient temperature. The reaction was quenched by addition of saturated aqueous $NaHCO_3$ (300 mL). The phases were separated and the organic phase was washed with brine (200 mL), dried over Na_2SO_4 and concentrated under reduced pressure. The crude residue was purified by flash column chromatography (SiO_2; pentane:EtOAc 99:1) to give macrolactone **72** (831 mg, 87%) as a colorless oil.

TLC: R_f = 0.35 (pentane:EtOAc 98:2)

^{1}H NMR: (400 MHz, $CDCl_3$) δ 6.69 (dd, J = 15.8, 7.2 Hz, 1H, $O_2CCH=CH$), 5.89 (dd, J = 15.8, 1.5 Hz, $O_2CCH=CH$), 4.89 (dddd, J = 9.8, 7.6, 5.6, 2.2 Hz, CHO_2C), 4.43 (d, J = 7.2 Hz, 1H, CHC*H*(OTBS)CH(OTBS)CH_2), 3.72 (tdd, J = 11.5, 3.7, 1.2, 1H, CH_2C*H*(OTBS)CH_2), 3.64 (dd, J = 10.9, 4.0 Hz, 1H, CHCH(OTBS)C*H*(OTBS)CH_2), 2.24 (ddd, J = 14.1, 10.9, 4.1 Hz, 1H, CH_2), 1.70 – 1.11 (m, 19H, 10×CH_2), 0.92 (s, 9H, $OSi(CH_3)_2C(CH_3)_3$), 0.92 – 0.89 (m, 3H, CH_3), 0.89 (s, 9H, $OSi(CH_3)_2C(CH_3)_3$), 0.89 (s, 9H, $OSi(CH_3)_2C(CH_3)_3$), 0.11 (s, 3H, $OSi(CH_3)_2{}^t$Bu), 0.11 (s, 3H, $OSi(CH_3)_2{}^t$Bu), 0.09 (s, 3H, $OSi(CH_3)_2{}^t$Bu), 0.08 (s, 3H, $OSi(CH_3)_2{}^t$Bu), 0.07 (s, 3H, $OSi(CH_3)_2{}^t$Bu), 0.06 (s, 3H, $OSi(CH_3)_2{}^t$Bu) ppm

^{13}C NMR (101 MHz, $CDCl_3$) δ 166.9 (CO_2CH), 148.6 ($O_2CC=CH$), 121.8 ($O_2CC=CH$), 79.1 (CH*C*H(OTBS)CH(OTBS)), 76.2 (CHO_2C), 74.8 (CH(OTBS)*C*H(OTBS)CH_2), 68.9 (CH_2*C*H(OTBS)CH_2), 42.8 (CH_2), 35.4 (CH_2), 34.4 (CH_2), 32.6 (CH_2), 31.9 (CH_2), 27.4 (CH_2), 26.2 ($OSi(CH_3)_2C(CH_3)_3$), 26.2 ($OSi(CH_3)_2C(CH_3)_3$), 26.1 ($OSi(CH_3)_2C(CH_3)_3$), 25.3 (CH_2), 23.8 (CH_2), 23.6 (CH_2), 22.7 (CH_2), 18.6 ($OSi(CH_3)_2C(CH_3)_3$), 18.3 ($OSi(CH_3)_2C(CH_3)_3$), 18.3 ($OSi(CH_3)_2C(CH_3)_3$), 14.2 (CH_3), -3.5 ($OSi(CH_3)_2{}^t$Bu), -4.3 ($OSi(CH_3)_2{}^t$Bu), -4.5 ($OSi(CH_3)_2{}^t$Bu), -4.5 ($OSi(CH_3)_2{}^t$Bu), -4.7 ($OSi(CH_3)_2{}^t$Bu), -4.7 ($OSi(CH_3)_2{}^t$Bu) ppm

HRMS (*m/z*): (ESI) calc'd for $C_{36}H_{74}O_5Si_3Na$ $[M+Na]^+$: 693.4736, found: 693.4761

IR (neat) ν_{max}: 2929, 2857, 1719, 1253, 1049, 832 and 773 cm^{-1}

$[\alpha]_D^{20}$: +19.3 (c = 0.22, $CHCl_3$)

(5*R*,6*S*,8*R*,14*R*,*E*)-5,6,8-Trihydroxy-14-pentyloxacyclotetradec-3-en-2-one/Sch 725674 (21)

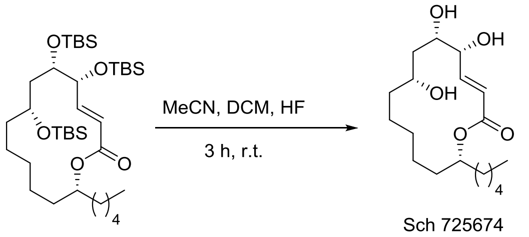

Macrolactone **72** (43.2 mg, 64.4 μmol) was dissolved in acetonitrile (3 mL) and dichloromethane (1 mL) and hydrofluoric acid (48 wt. % in H_2O, 0.5 mL) was added at ambient temperature. After 3 hr the reaction was quenched with saturated aqueous $NaHCO_3$ (10 mL). After extraction with EtOAc (3×10 mL) the combined organic phases were washed with brine (25 ml), dried over Na_2SO_4 and concentrated under reduced pressure. Purification by flash column chromatography (SiO_2; MeOH:CH_2Cl_2 95:5) yielded the natural product **21** (19.1 mg, 90%) as an amorphous white solid.

Using 527 mg of **72**, 226 mg of Sch 725674 could be obtained (88%).

TLC: R_f = 0.50 (EtOAc)

^{1}H NMR: (400 MHz, $CDCl_3$) δ 6.87 (dd, J = 15.8, 6.1 Hz, 1H, O_2CCH=C*H*), 6.08 (dd, J = 15.8, 1.6 Hz, O_2CC*H*=CH), 4.95 (dddd, J = 10.0, 7.6, 5.1, 2.3 Hz, 1H, CHO_2C), 4.48 (ddd, J = 6.1, 3.0, 1.6 Hz, 1H, CHC*H*(OH)CH(OH)CH_2), 3.99 (p, J = 6.2 Hz, 1H, CH_2C*H*(OH)CH_2), 3.85 (ddd, J = 5.7, 4.4, 3.0 Hz, 1H, CHCH(OH)C*H*(OH)CH_2), 1.83 (ddd, J = 14.7, 6.5, 6.0 Hz, 1H, CH_2), 1.75 – 1.10 (m, 19H, 10×CH_2), 0.90 (t, J = 6.5 Hz, 3H, CH_3) ppm

^{13}C NMR (101 MHz, CD_3OD) δ 168.2 (*C*O_2CH), 149.2 (O_2CC=*C*H), 122.9 (O_2C*C*=CH), 77.5 (*C*HO_2C), 75.9 (CH*C*H(OH)CH(OH)CH_2), 72.8 (CHCH(OH)*C*H(OH)CH_2), 69.3 (CH_2*C*H(OH)CH_2), 38.2 (CH_2), 36.7 (CH_2), 36.4 (CH_2), 33.9 (CH_2), 32.8 (CH_2), 29.4 (CH_2), 26.8 (CH_2), 26.2 (CH_2), 25.6 (CH_2), 23.6 (CH_2), 14.3 (CH_3) ppm

HRMS (*m*/*z*): (ESI) calc'd for $C_{18}H_{32}O_5Na$ $[M+Na]^+$: 351.2142, found: 351.2143

$[\alpha]_D^{20}$**:** +3.5 (c = 0.29, MeOH)

The analytical data are in complete agreement with those reported for Sch 725674.[29]

5. NMR Data

[29] (a) S.-W. Yang, T.-M Chan, J. Terracciano, D. Loebenberg, M. Patel, M. Chu, *J. Antibiot.* **2005**, 58, 535; (b) J. D. Moretti, X. Wang, D. P. Curran, *J. Am. Chem. Soc.* **2012**, 134, 7963.

tert-Butyldimethyl((1-(4,4,5,5-tetramethyl-1,3,2-dioxaborolan-2-yl)heptyl)oxy)silane (34):

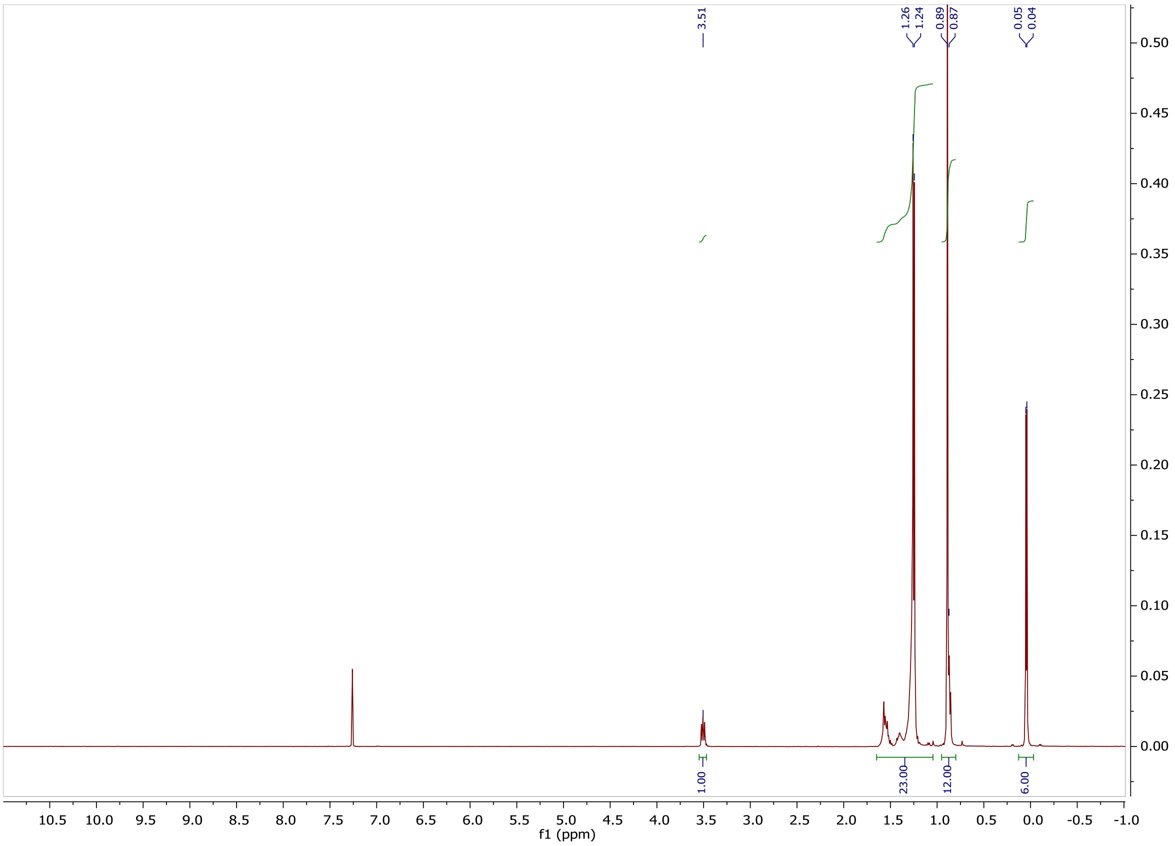

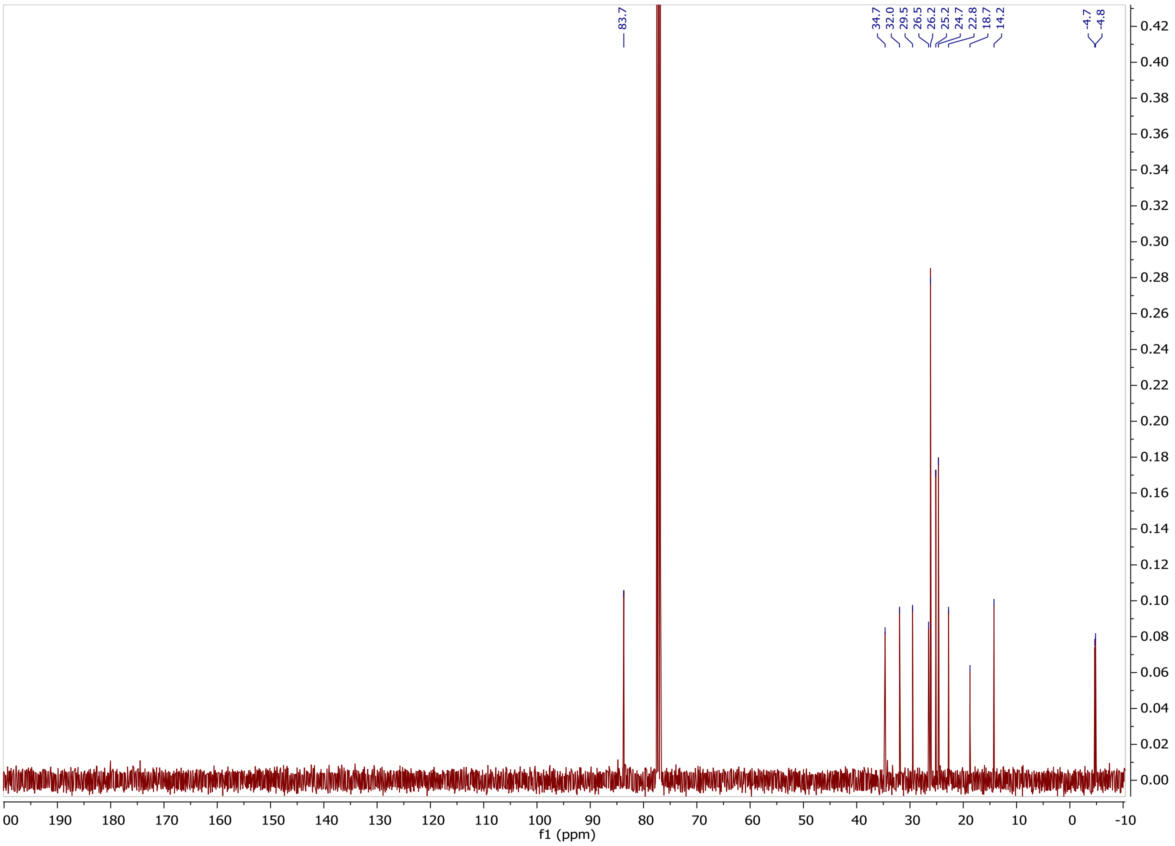

***tert*-Butyldimethyl((1-(4,4,5,5-tetramethyl-1,3,2-dioxaborolan-2-yl)octan-2-yl)oxy)silane (35):**

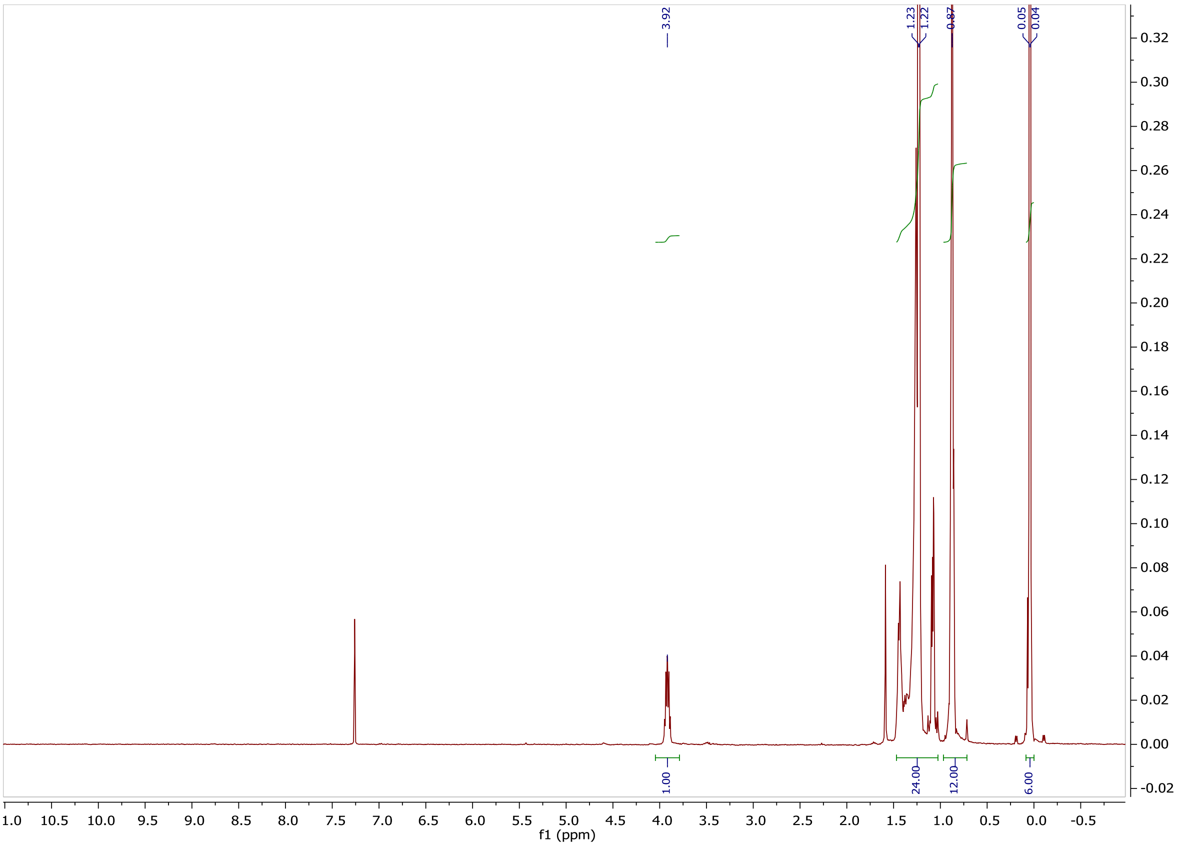

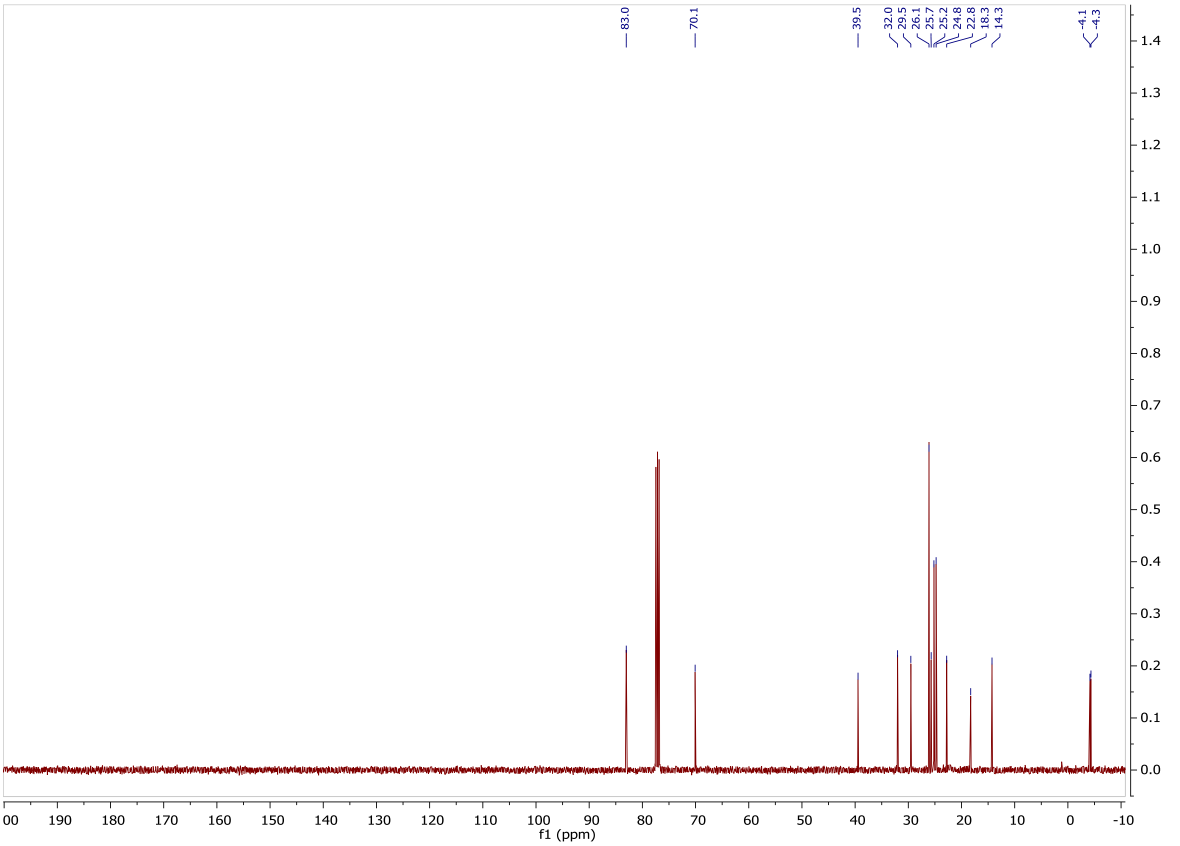

2,2'-(Octane-1,2-diyl)bis(4,4,5,5-tetramethyl-1,3,2-dioxaborolane) (2)

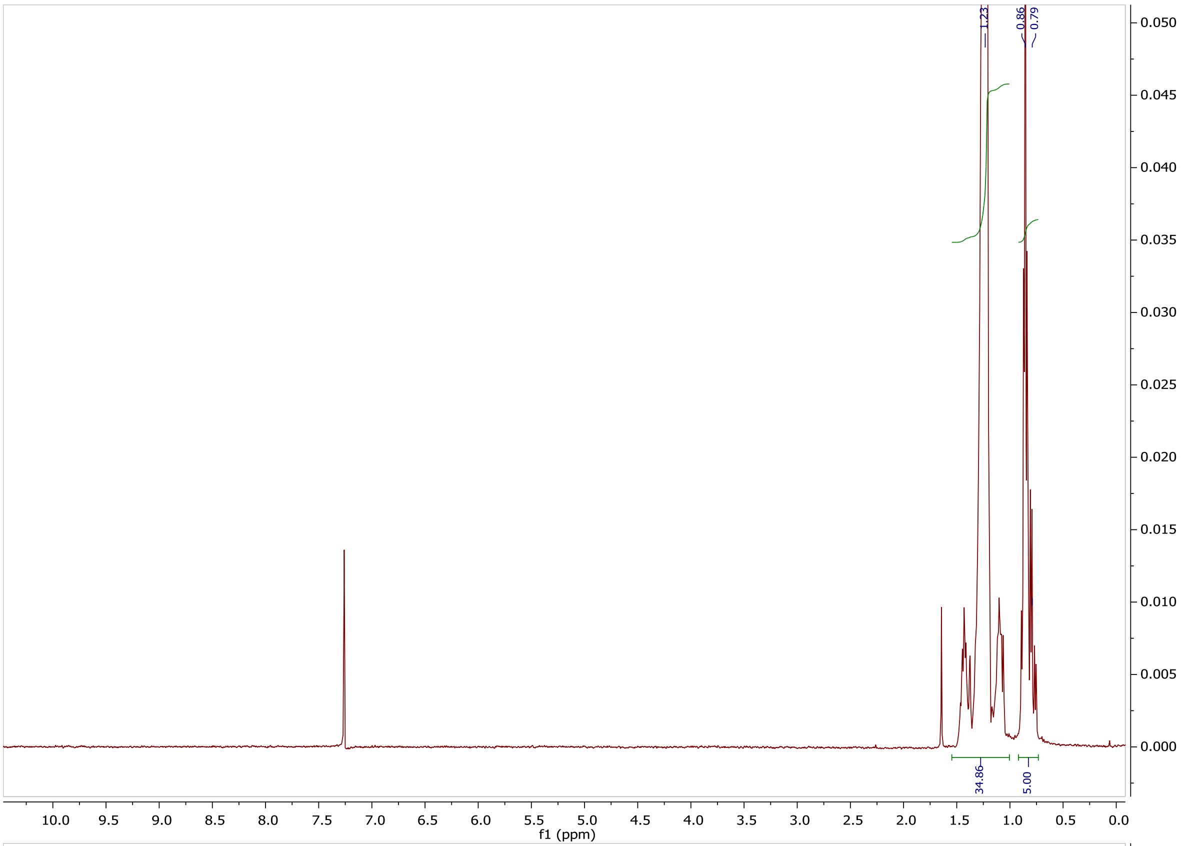

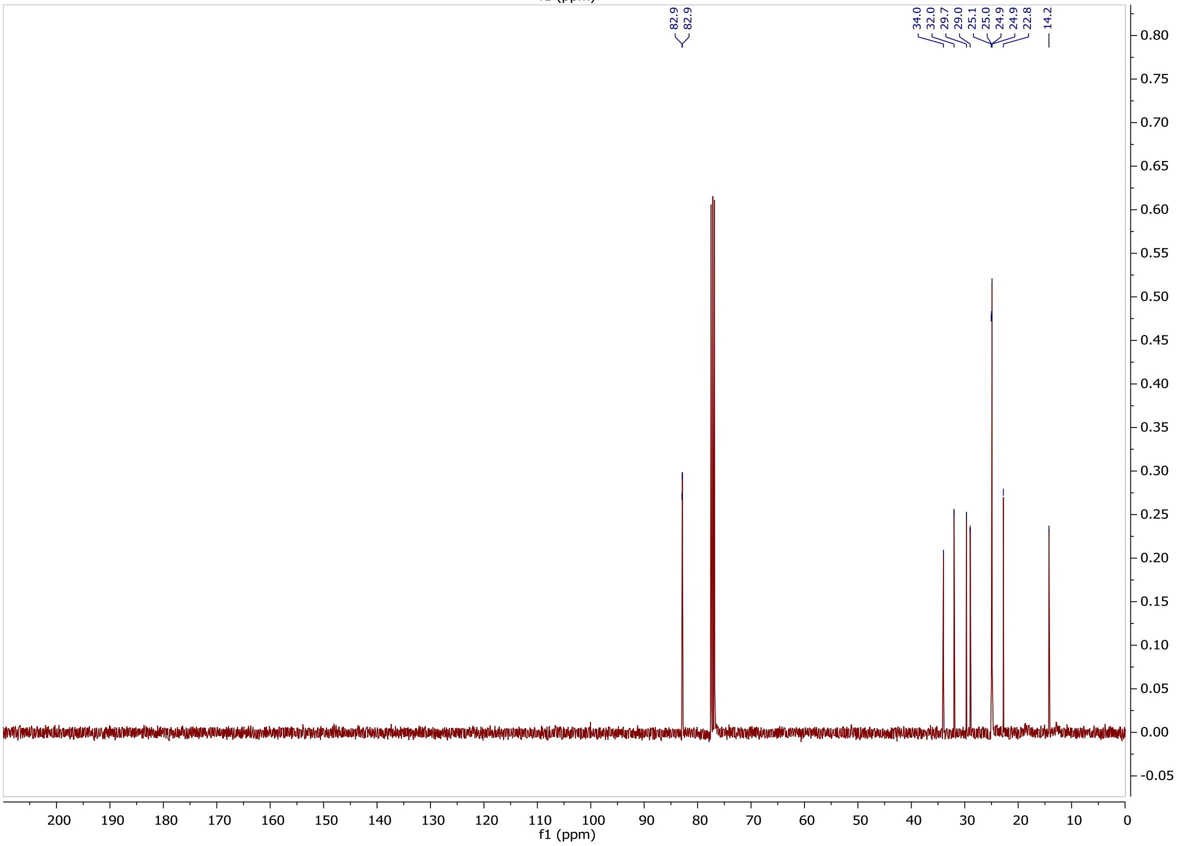

Isopentyl 2,4,6-triisopropylbenzoate (36):

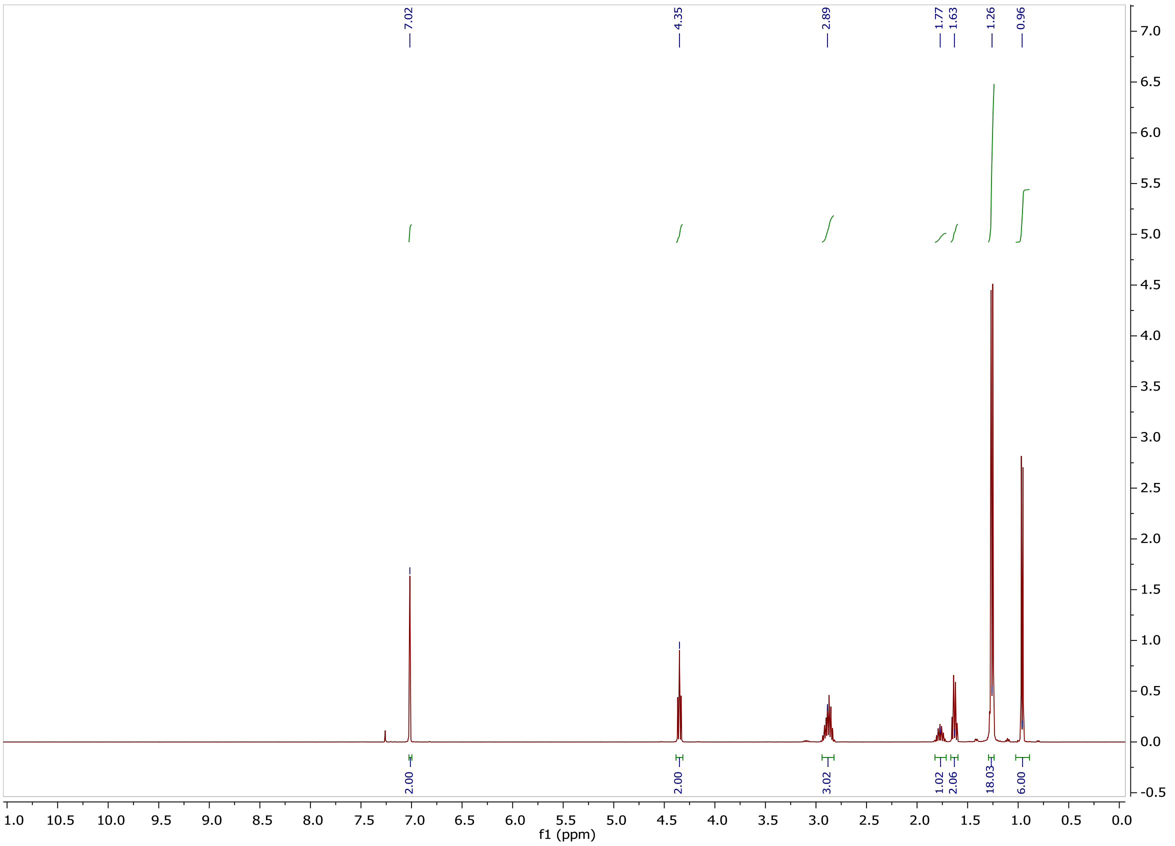

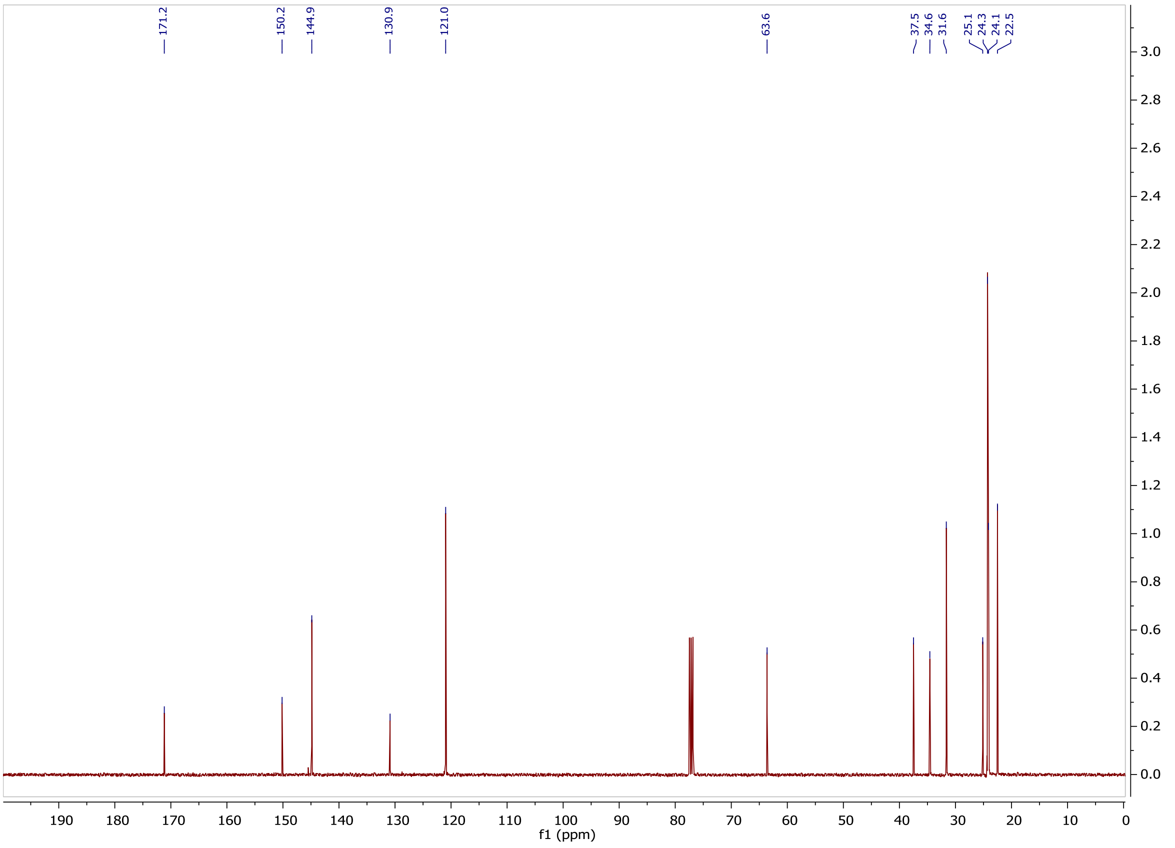

4,4,5,5-Tetramethyl-2-(2-methyldecan-4-yl)-1,3,2-dioxaborolane (37):

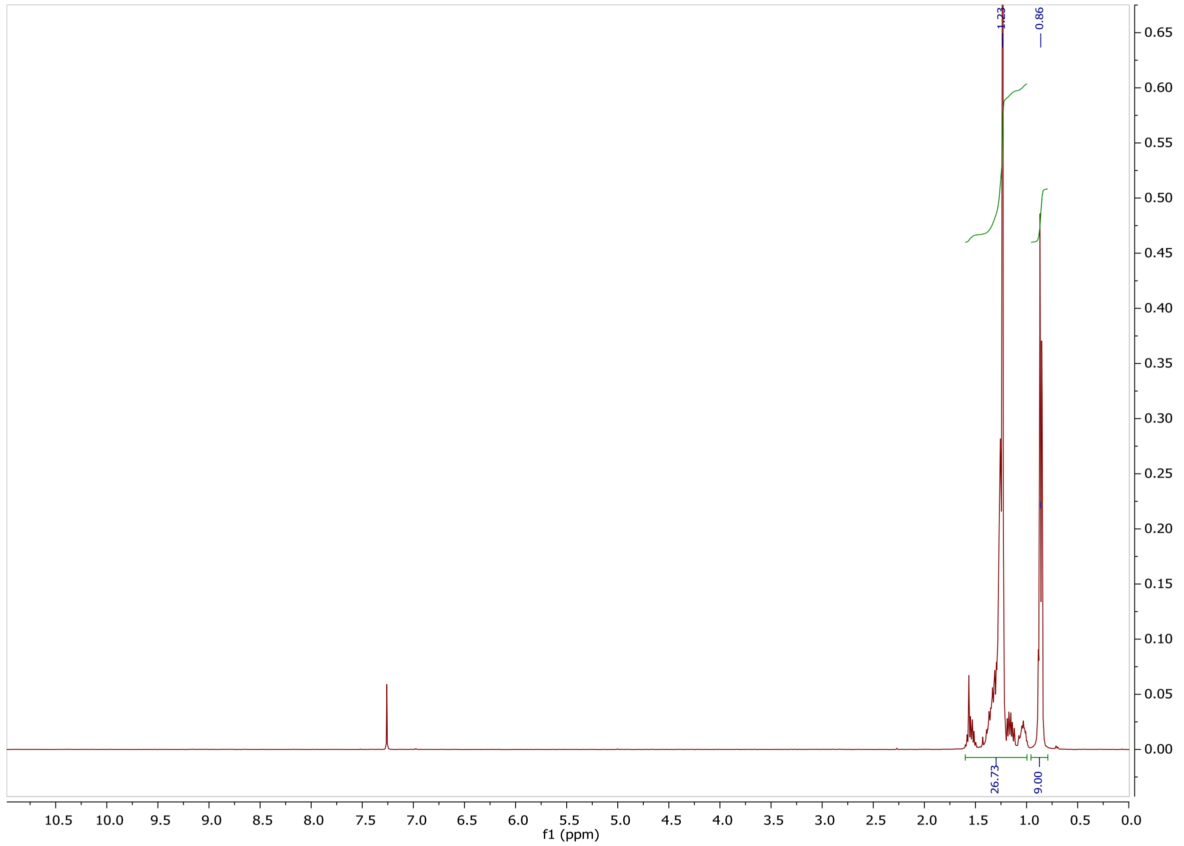

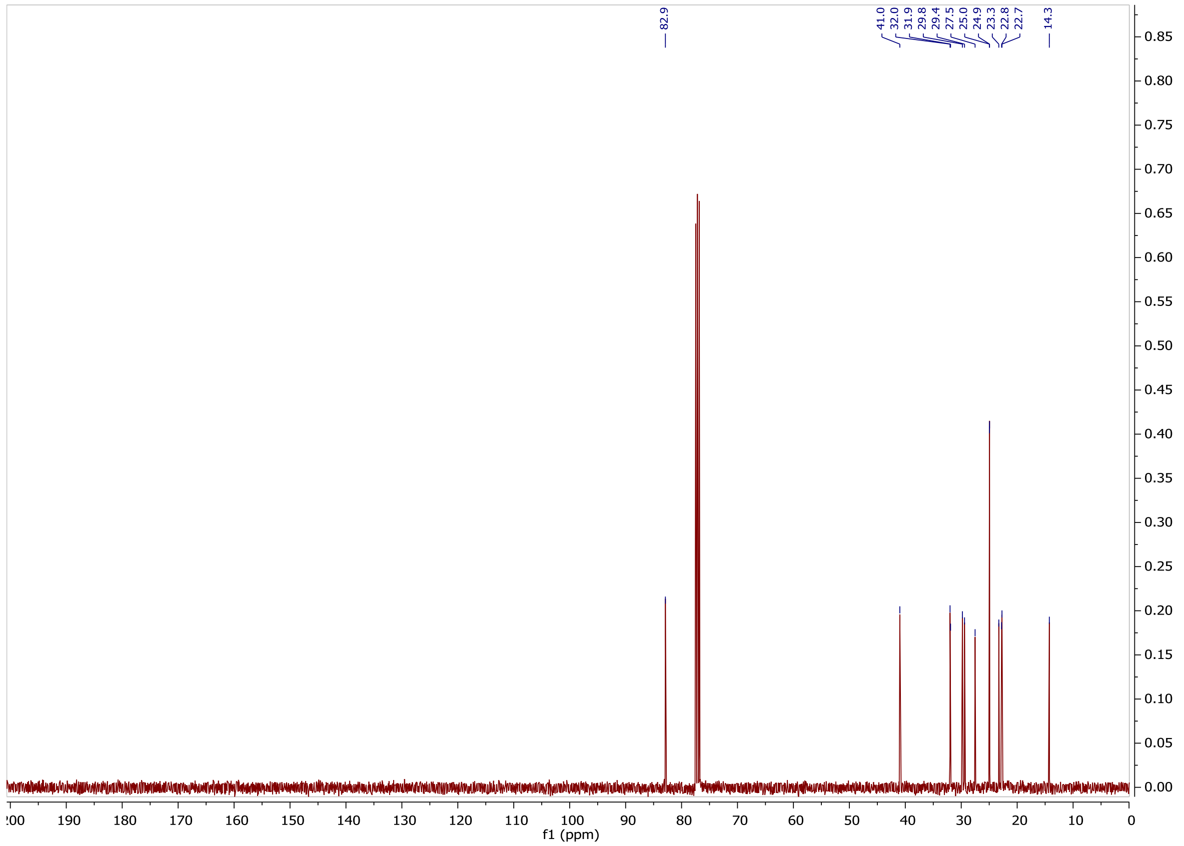

4,4,5,5-Tetramethyl-2-(2-methylnonan-3-yl)-1,3,2-dioxaborolane (38):

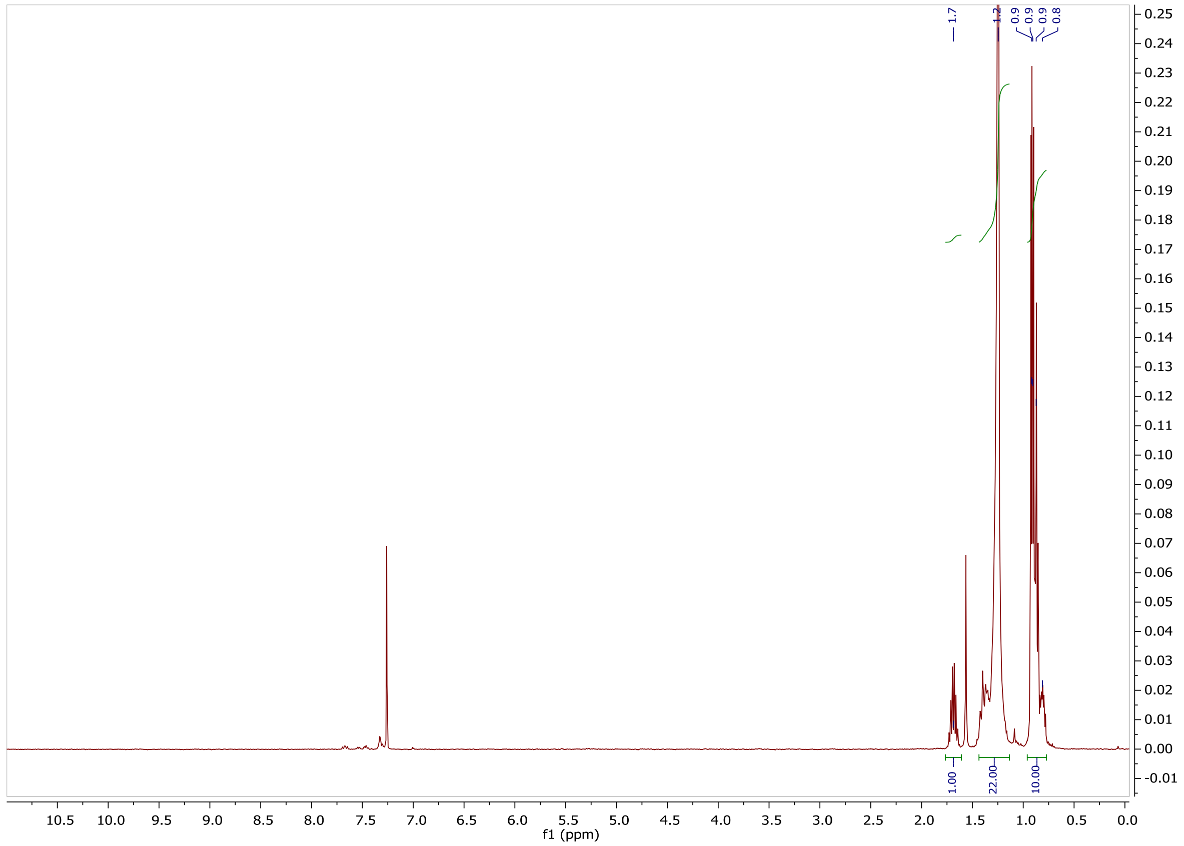

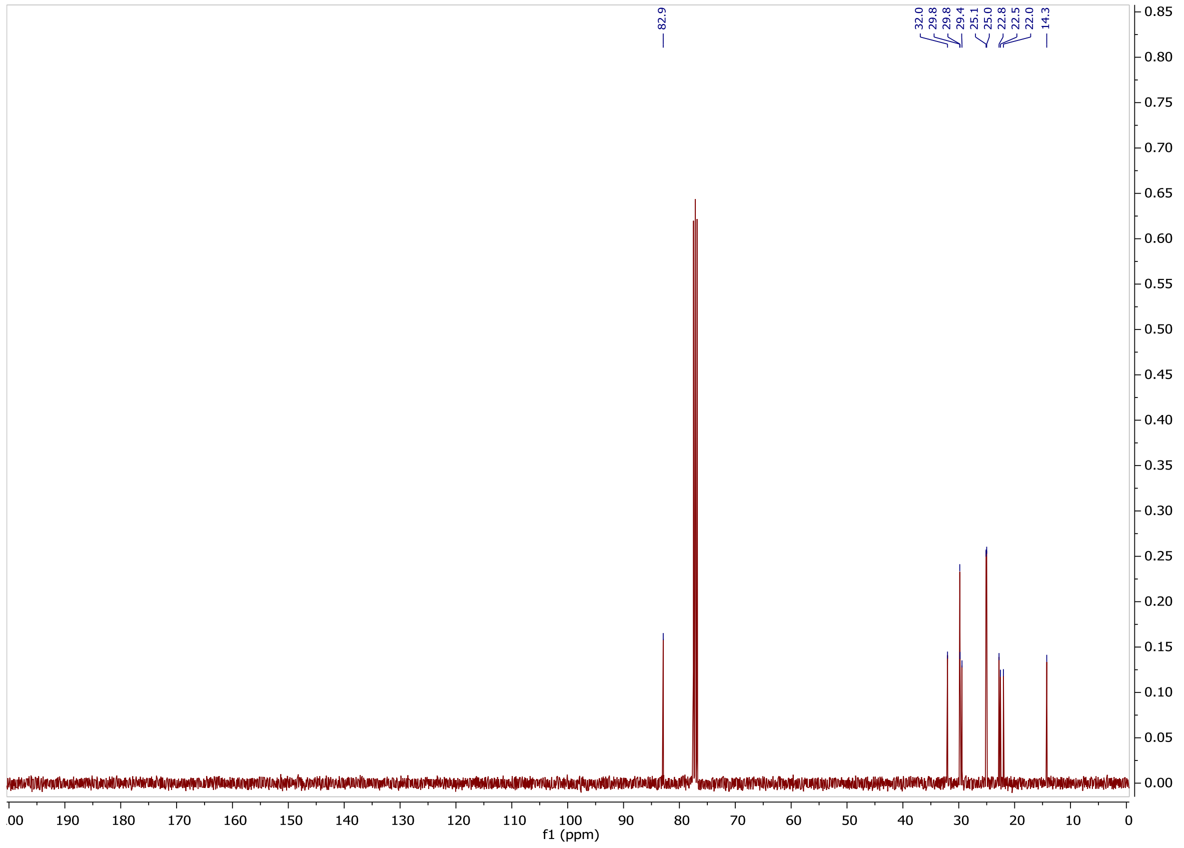

2-(2-Isopropyloctyl)-4,4,5,5-tetramethyl-1,3,2-dioxaborolane (39):

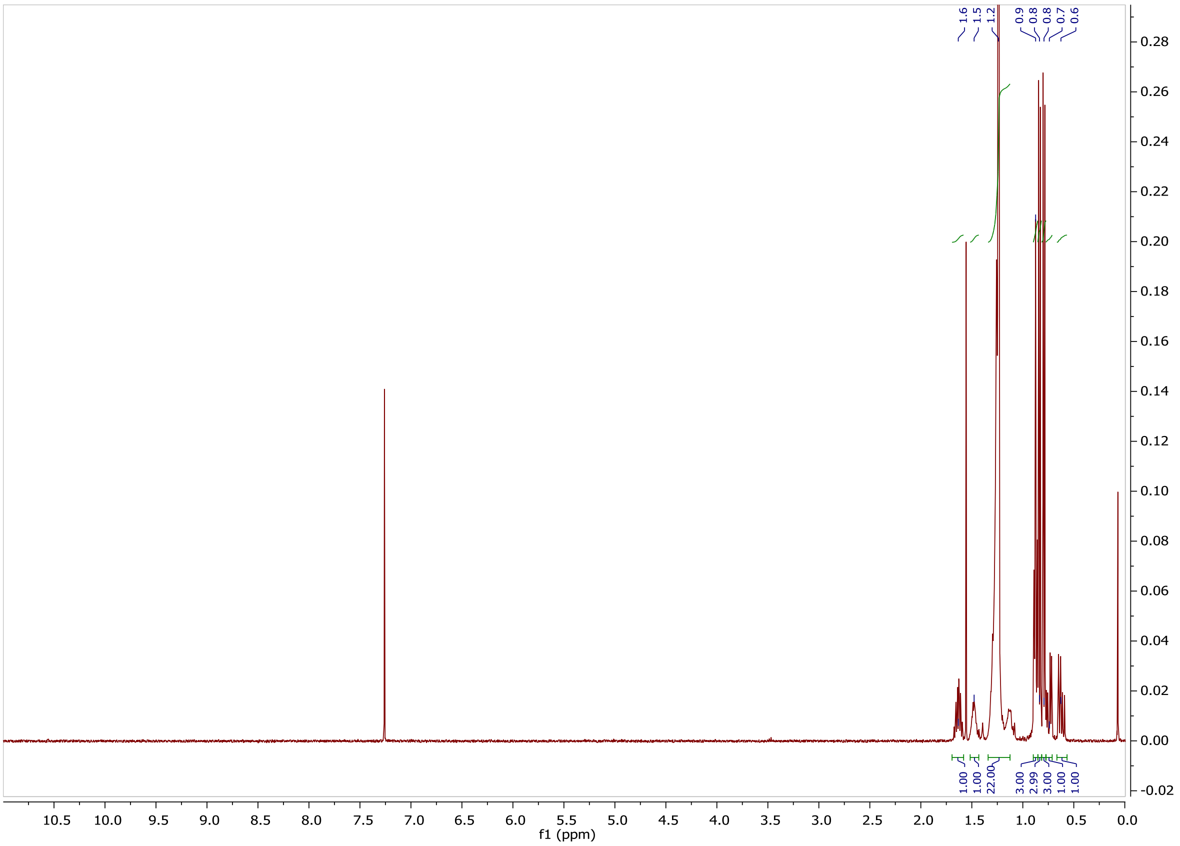

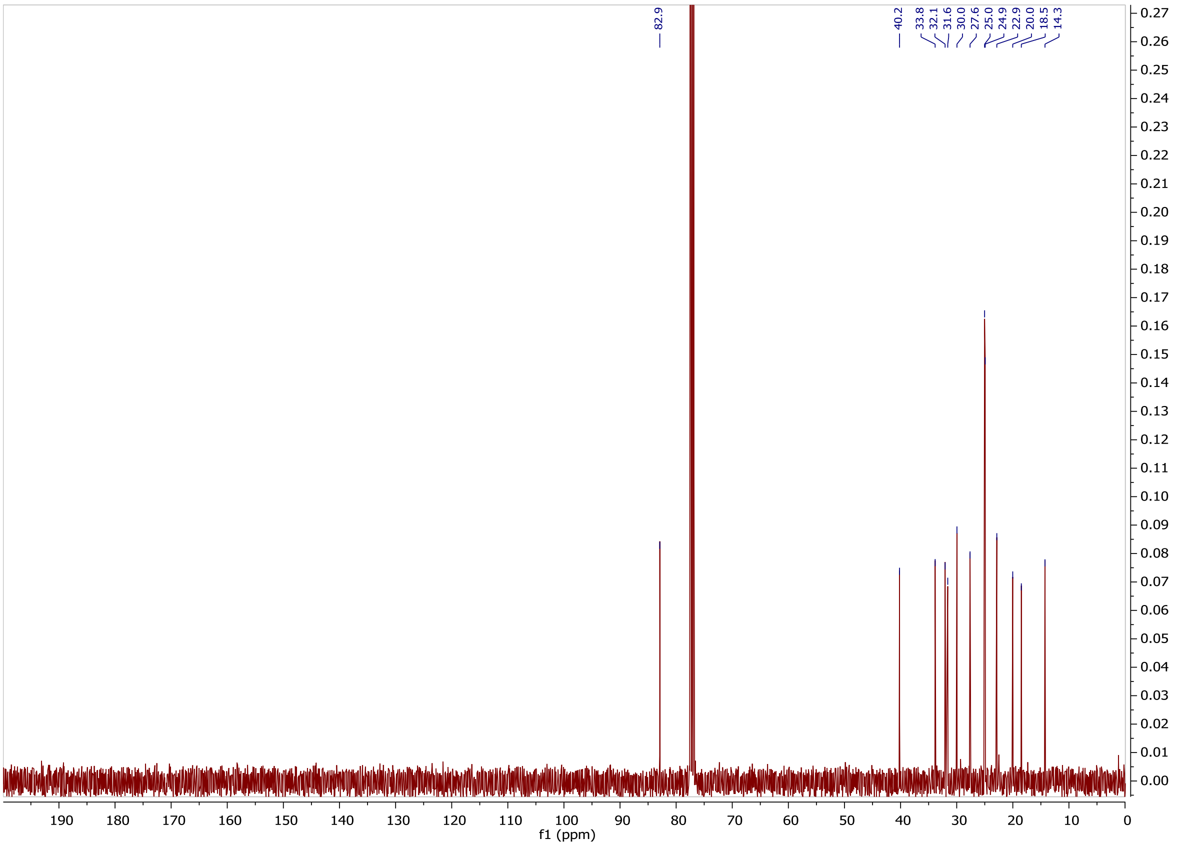

***tert*-Butyl((1-(4-methoxyphenyl)-3-(4,4,5,5-tetramethyl-1,3,2-dioxaborolan-2-yl)undecan-5-yl)oxy)dimethylsilane (40):**

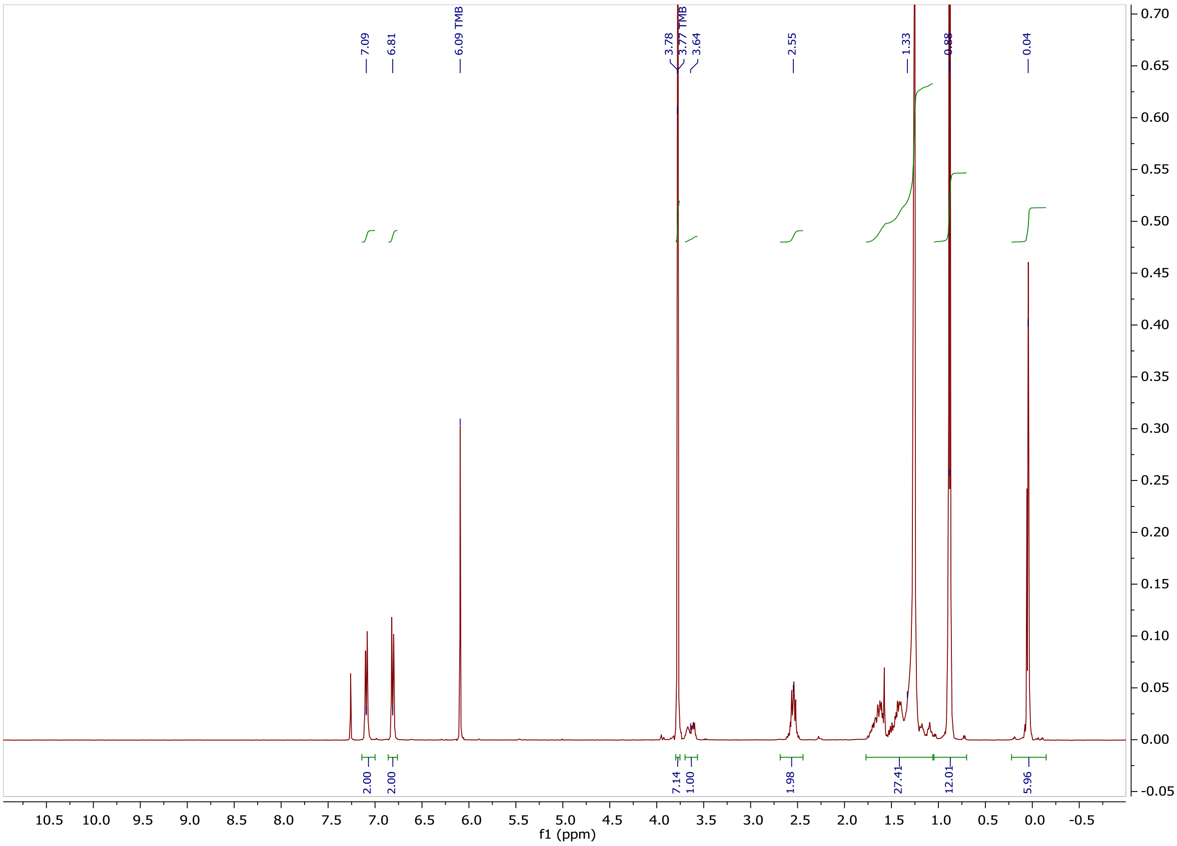

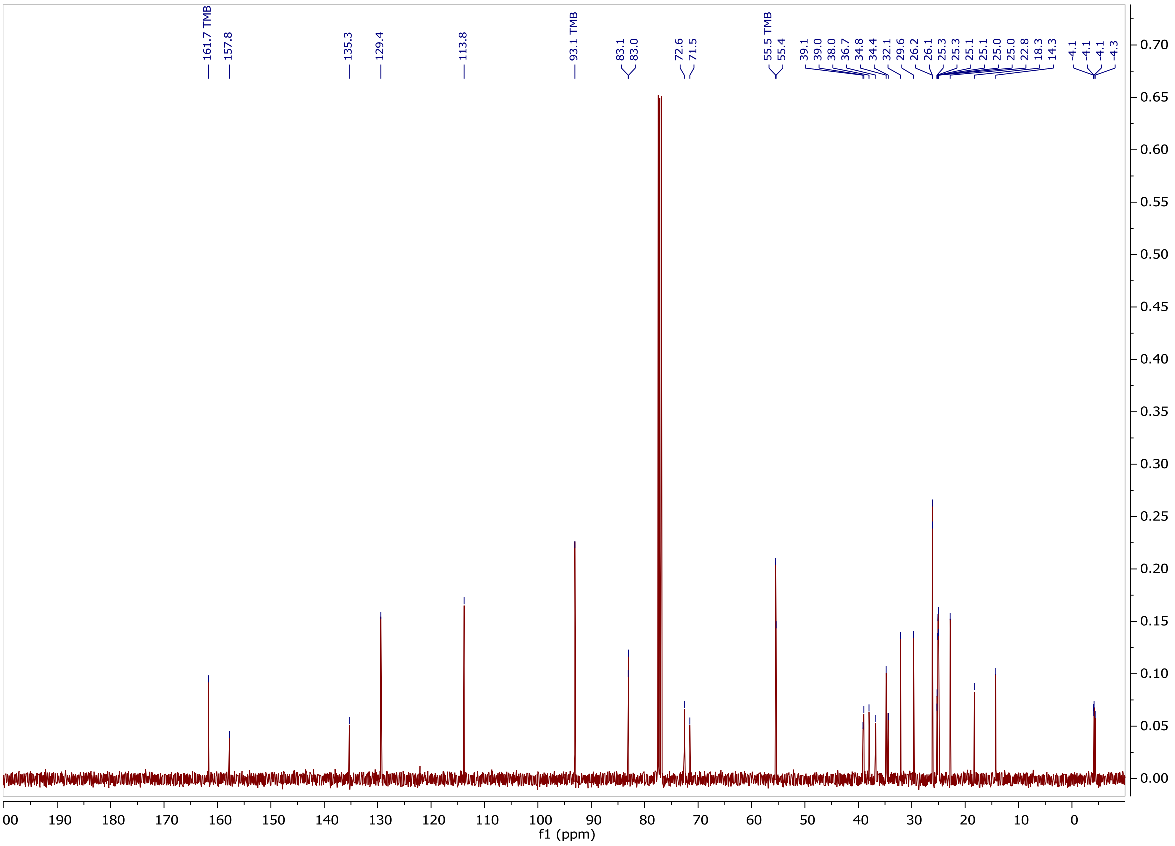

(3*S*,5*S*)-1-(4-Methoxyphenyl)undecane-3,5-diol (*S*,*S*-3):

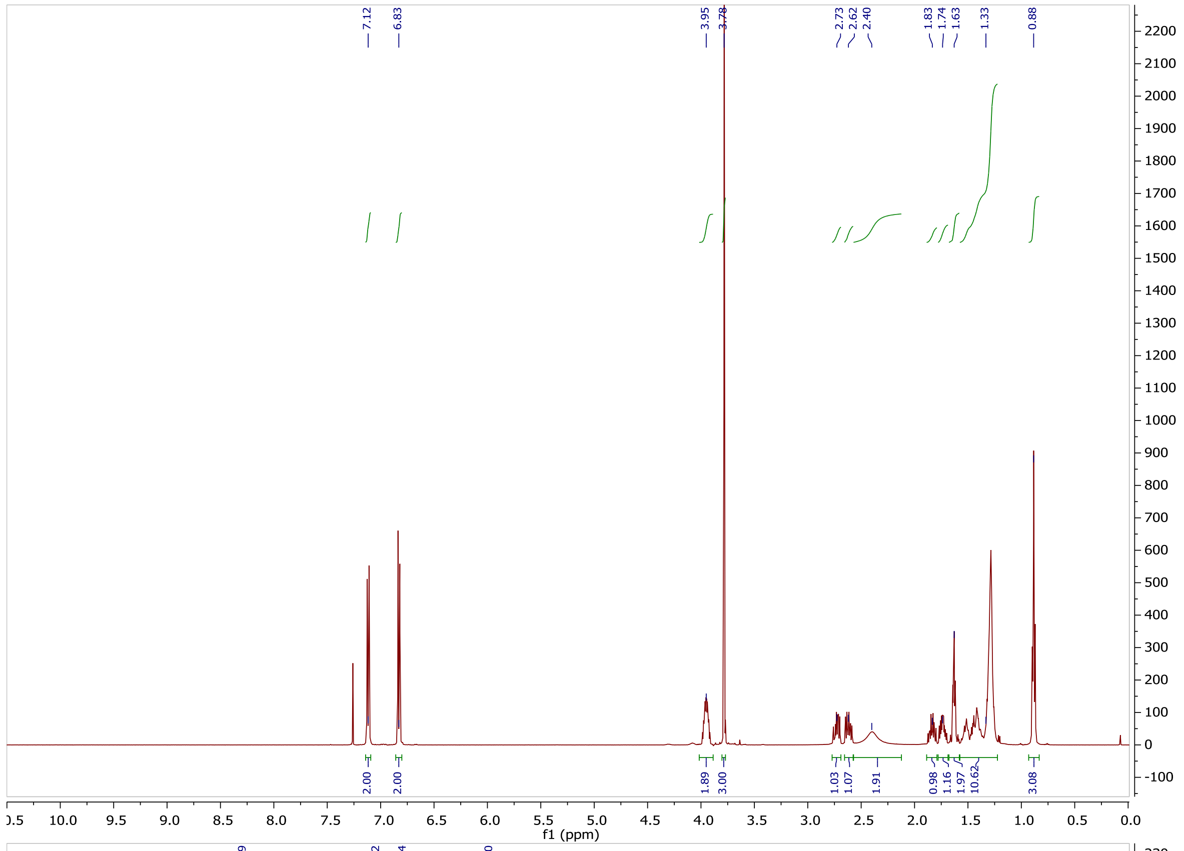

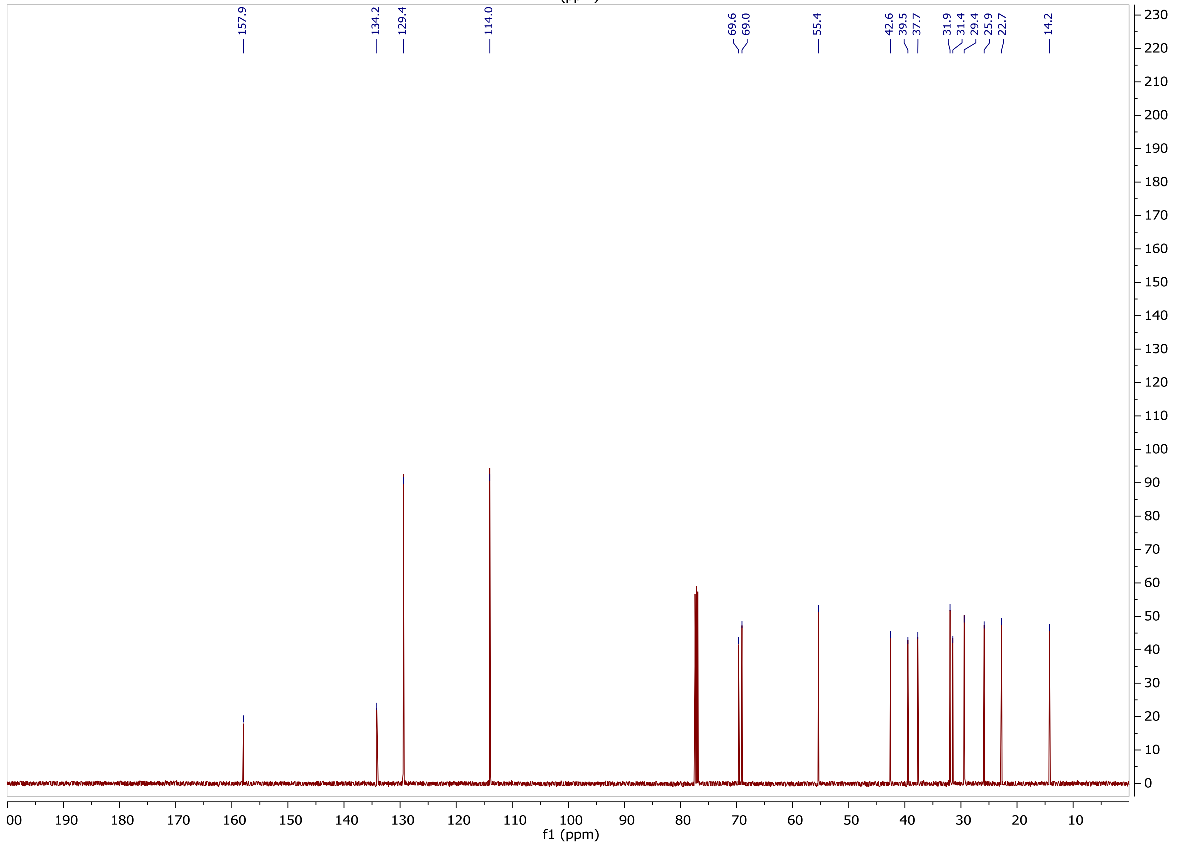

(3*R*,5*S*)-1-(4-Methoxyphenyl)undecane-3,5-diol (*R*,*S*-3):

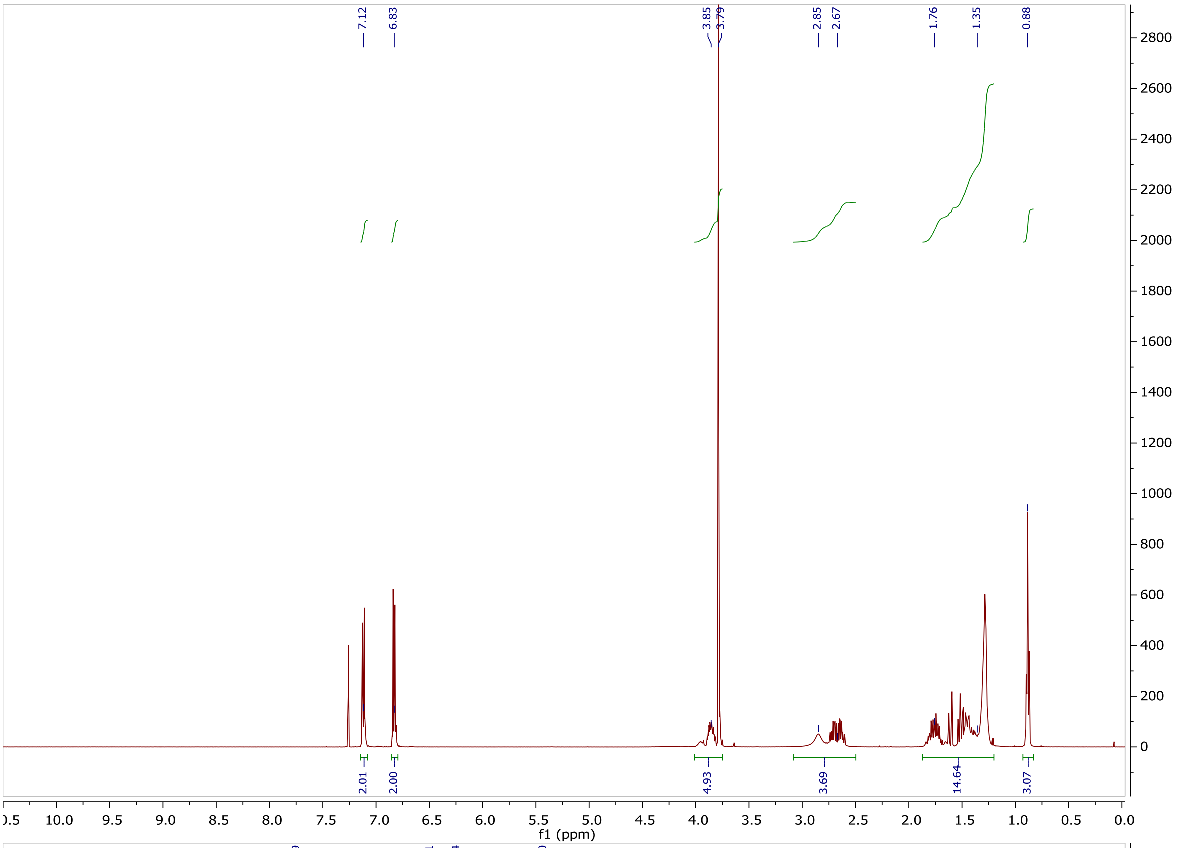

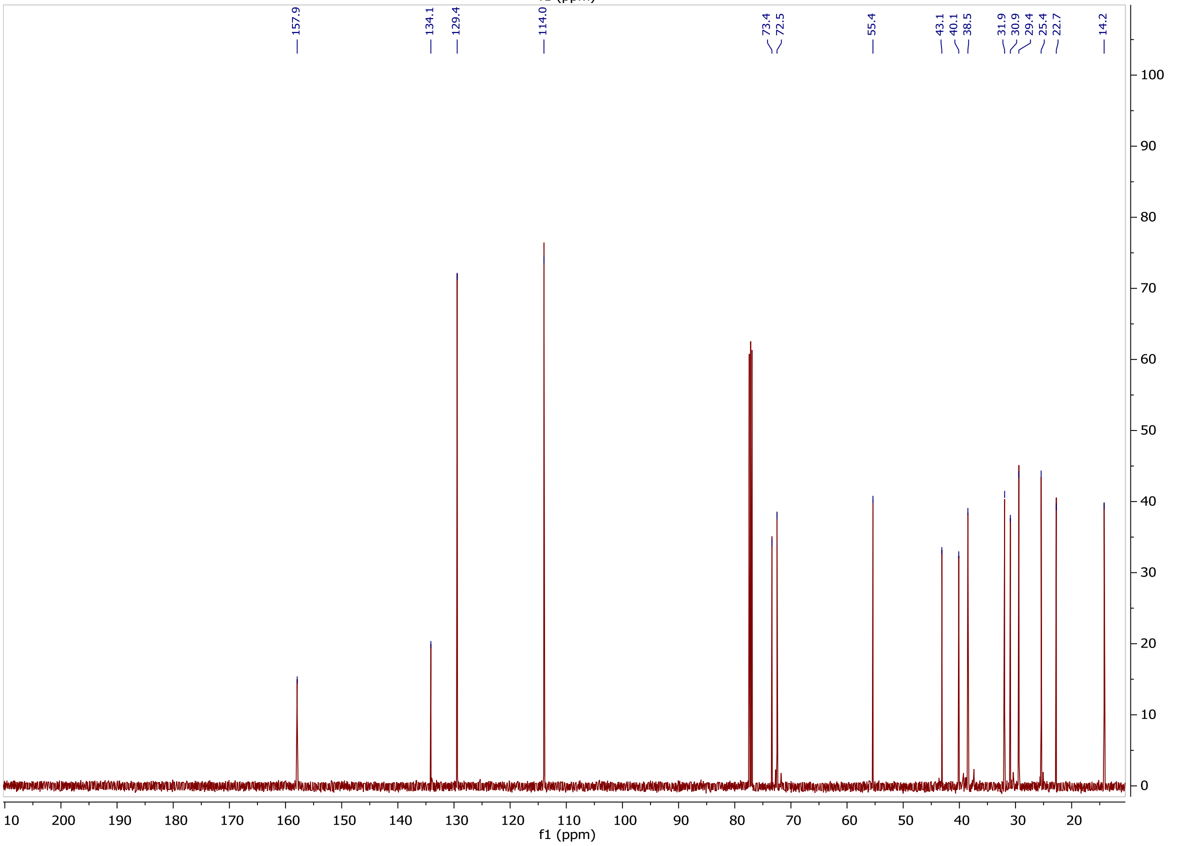

(3*S*,4*S*,6*S*)-4-Hexyl-1,8-bis(4-methoxyphenyl)octane-3,6-diol (3b)

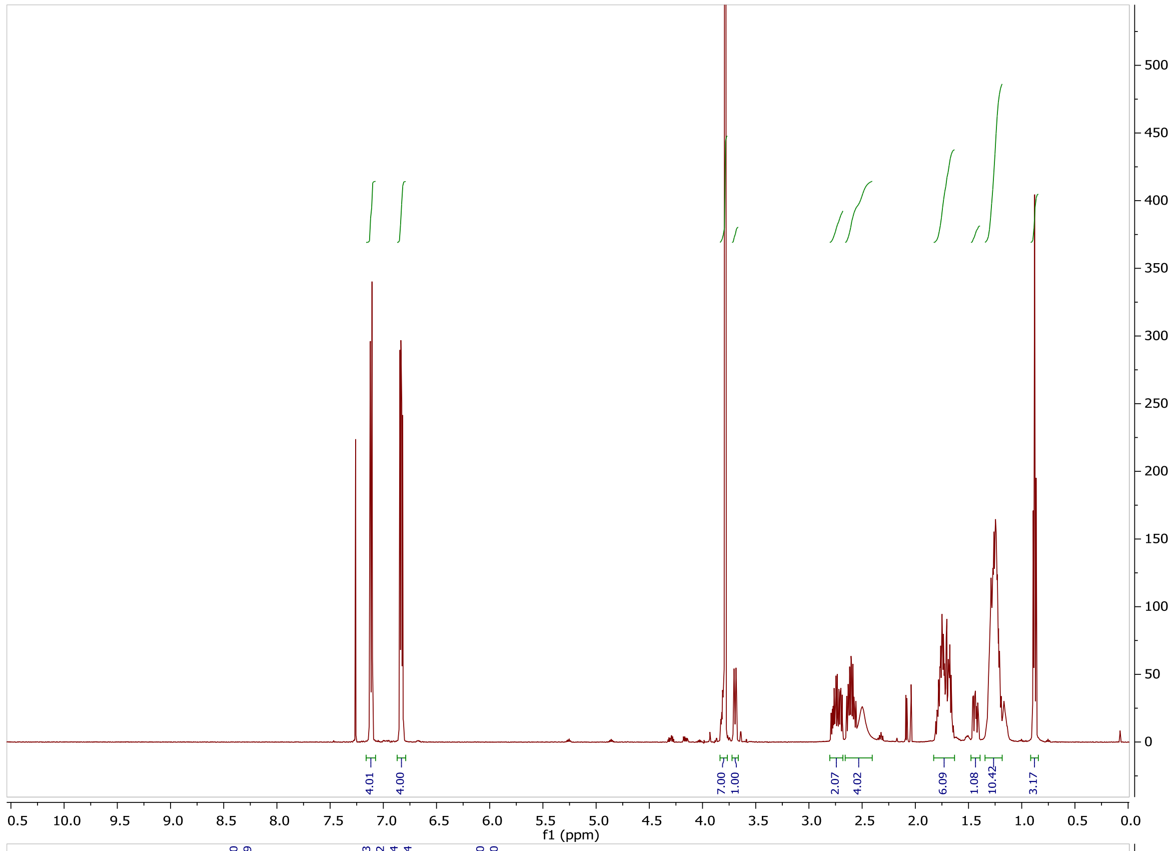

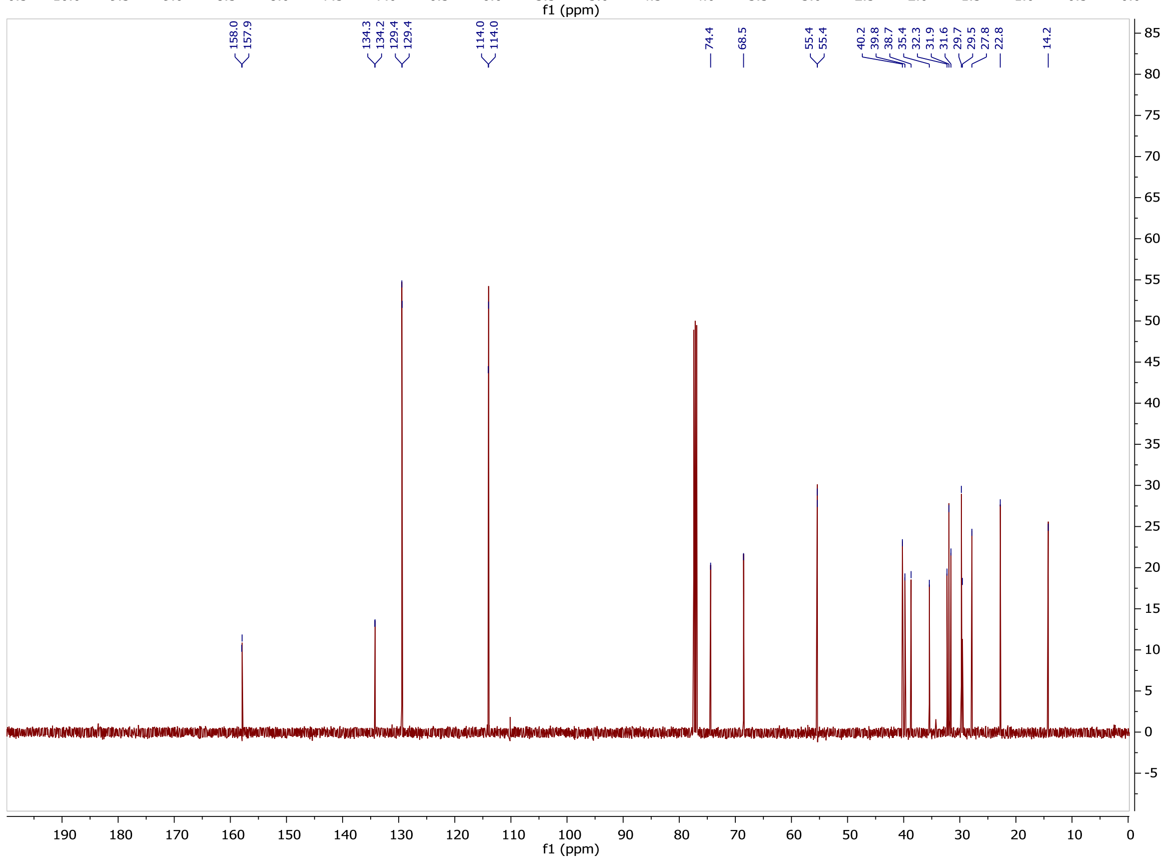

3-((*tert*-Butyldimethylsilyl)oxy)propyl diisopropylcarbamate (52):

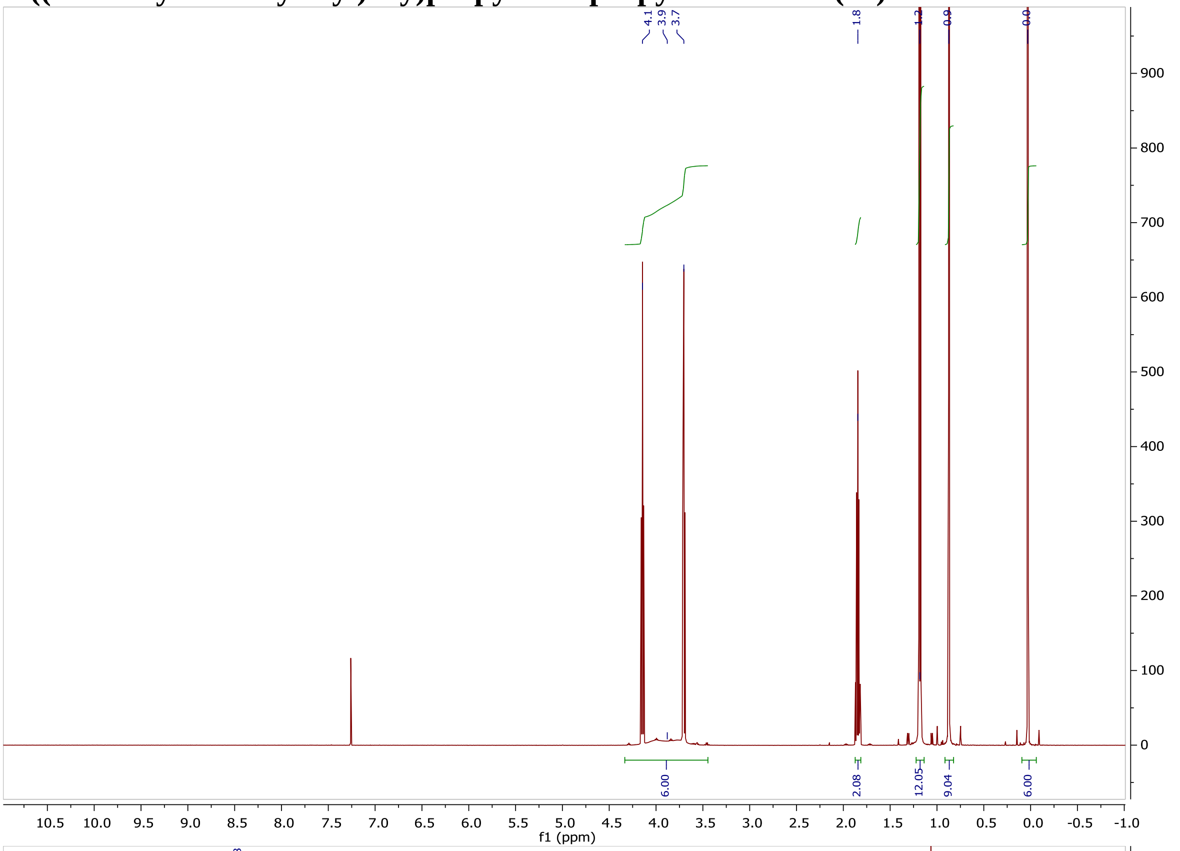

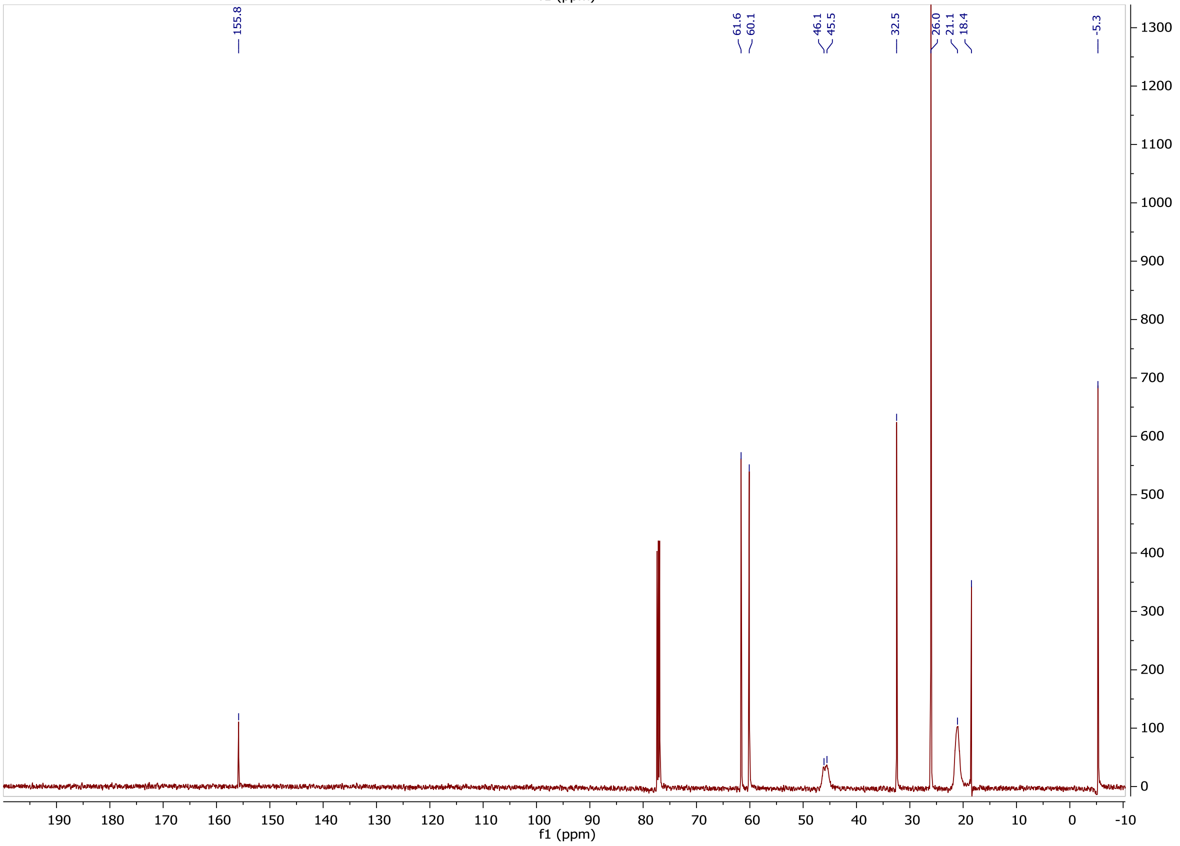

tert-Butyl hept-6-enoate (53):

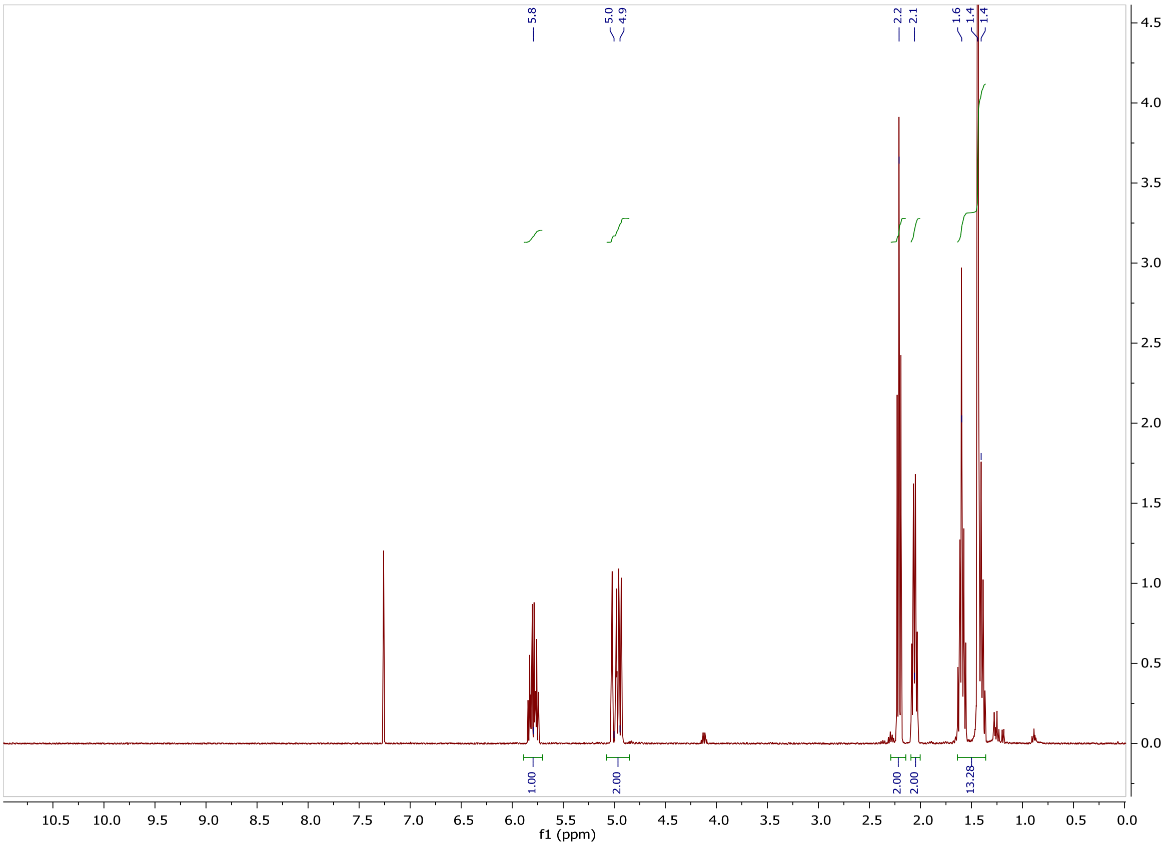

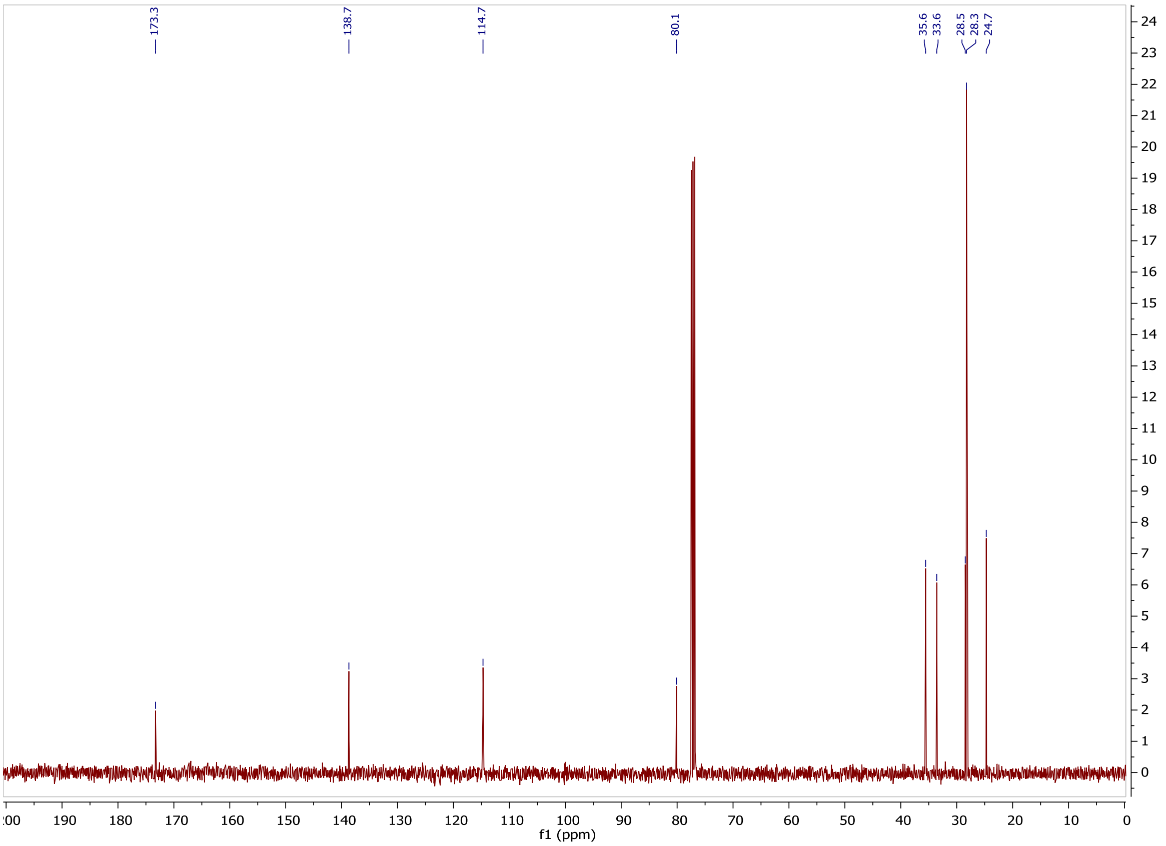

tert-Butyl (_R_)-6,7-bis(4,4,5,5-tetramethyl-1,3,2-dioxaborolan-2-yl)heptanoate (54):

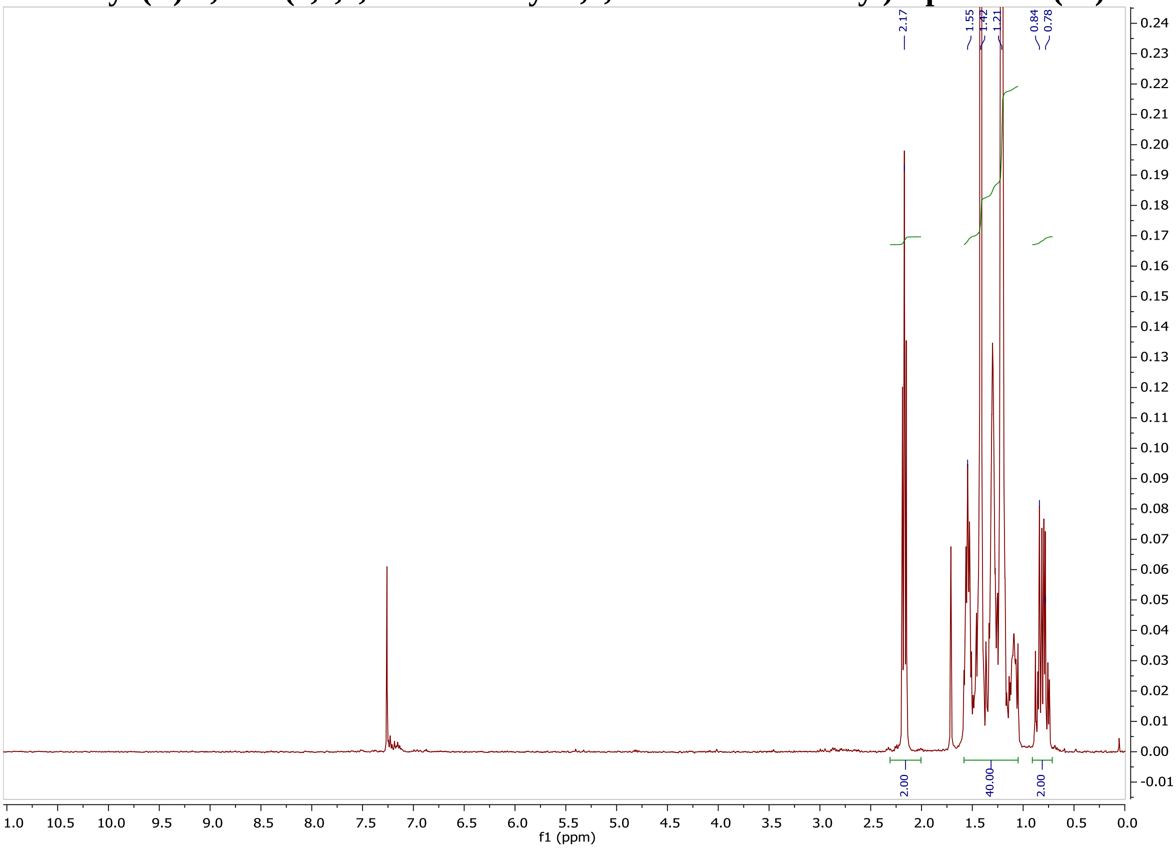

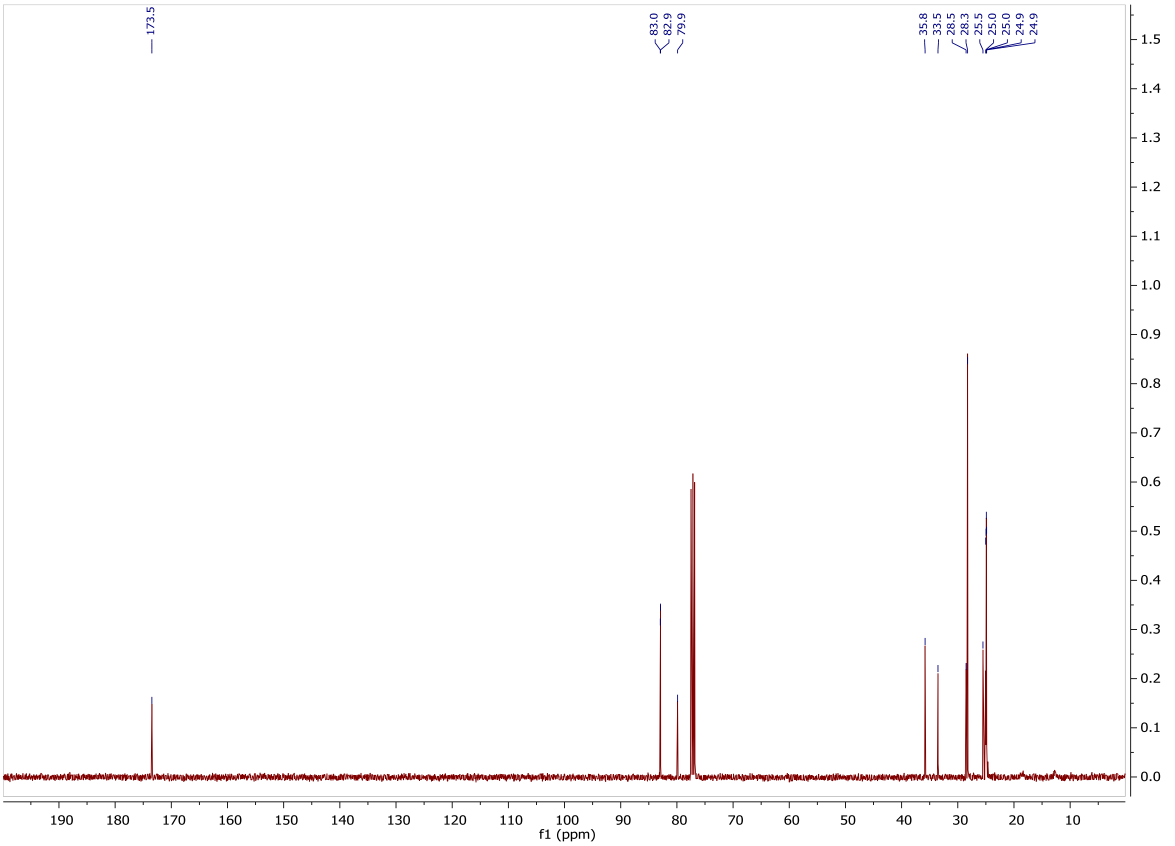

(*R*)-2,2'-(1-(4-Methoxyphenyl)ethane-1,2-diyl)bis(4,4,5,5-tetramethyl-1,3,2-dioxaborolane) (55):

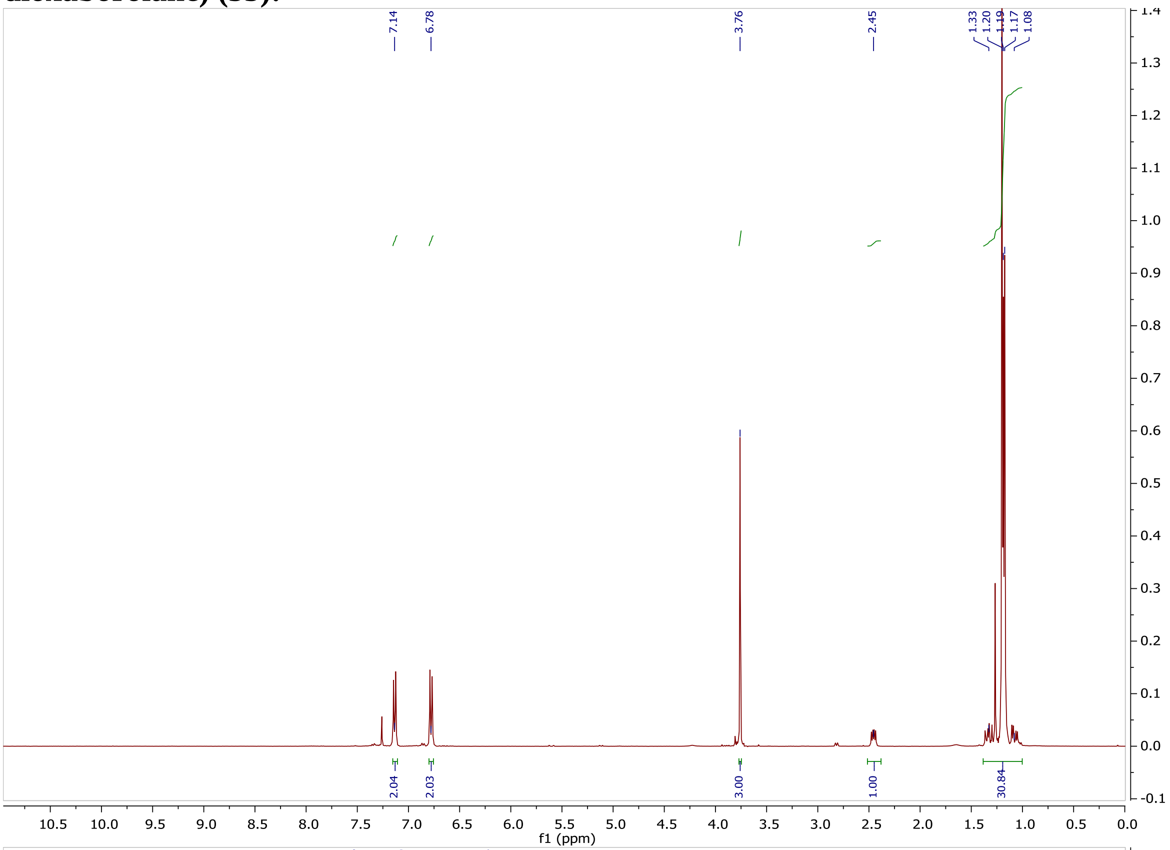

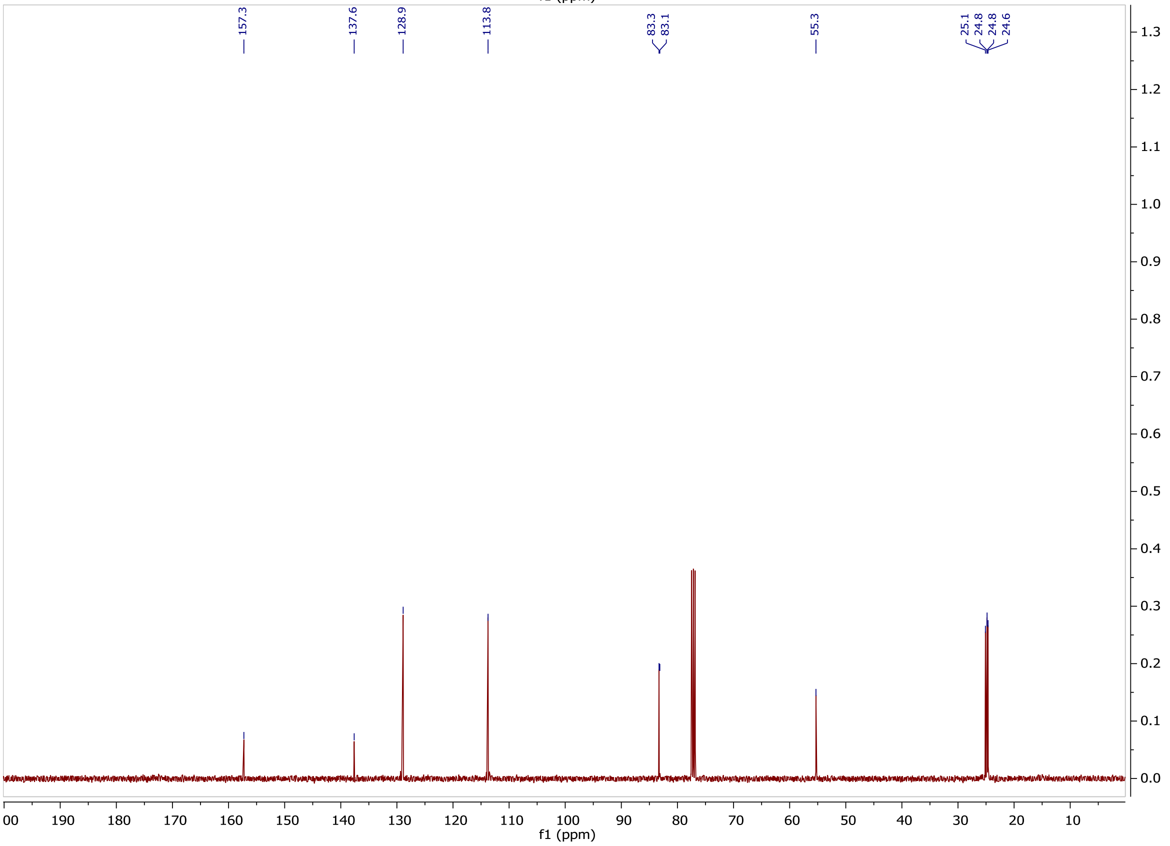

(*R*)-4,5-Bis(4,4,5,5-tetramethyl-1,3,2-dioxaborolan-2-yl)pentyl diisopropylcarbamate (56):

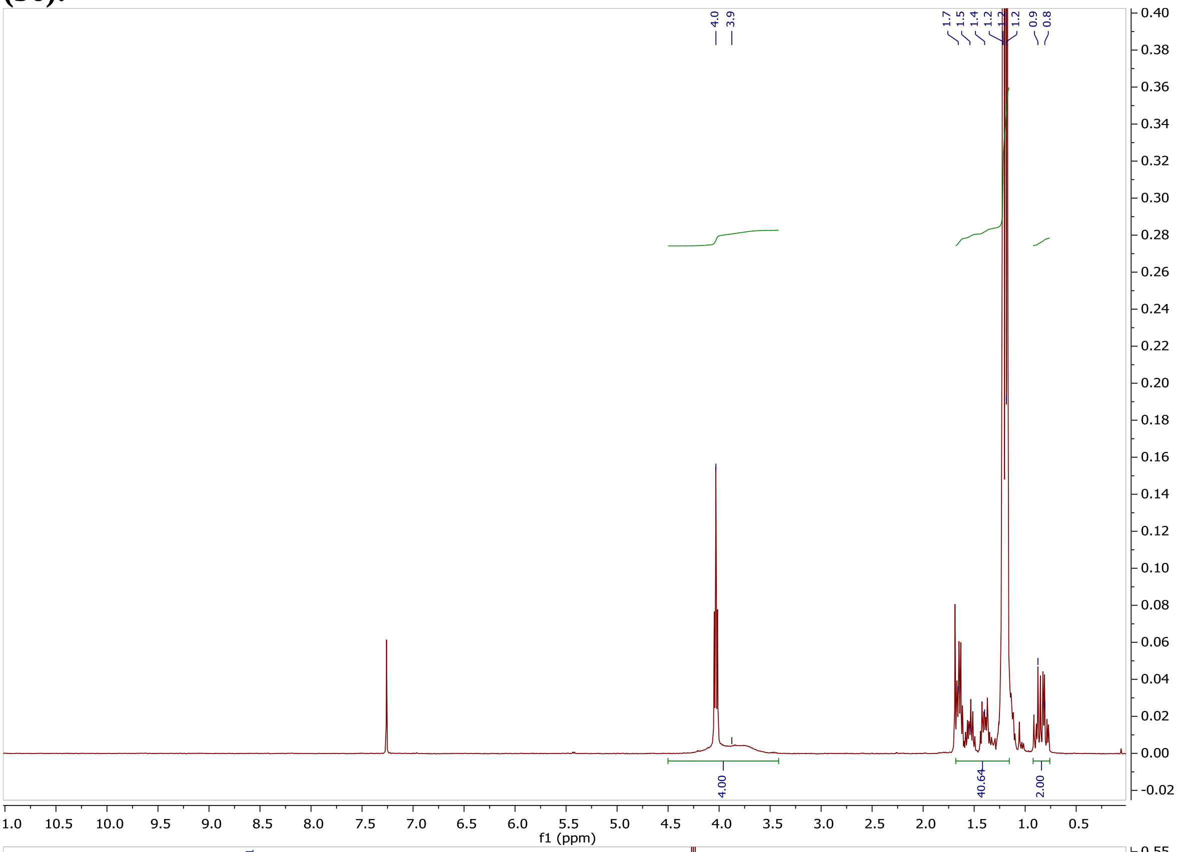

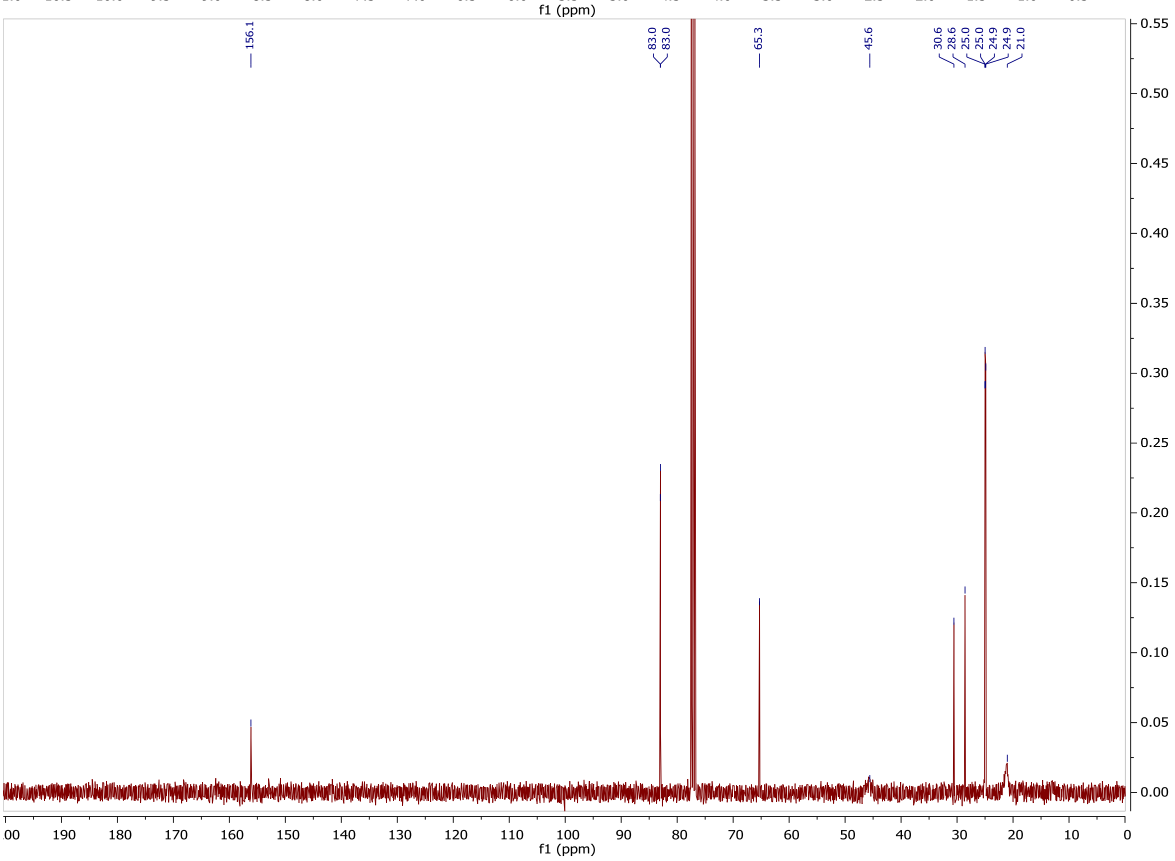

(*R*)-2,2'-(4,4-dimethylpentane-1,2-diyl)bis(4,4,5,5-tetramethyl-1,3,2-dioxaborolane) (57):

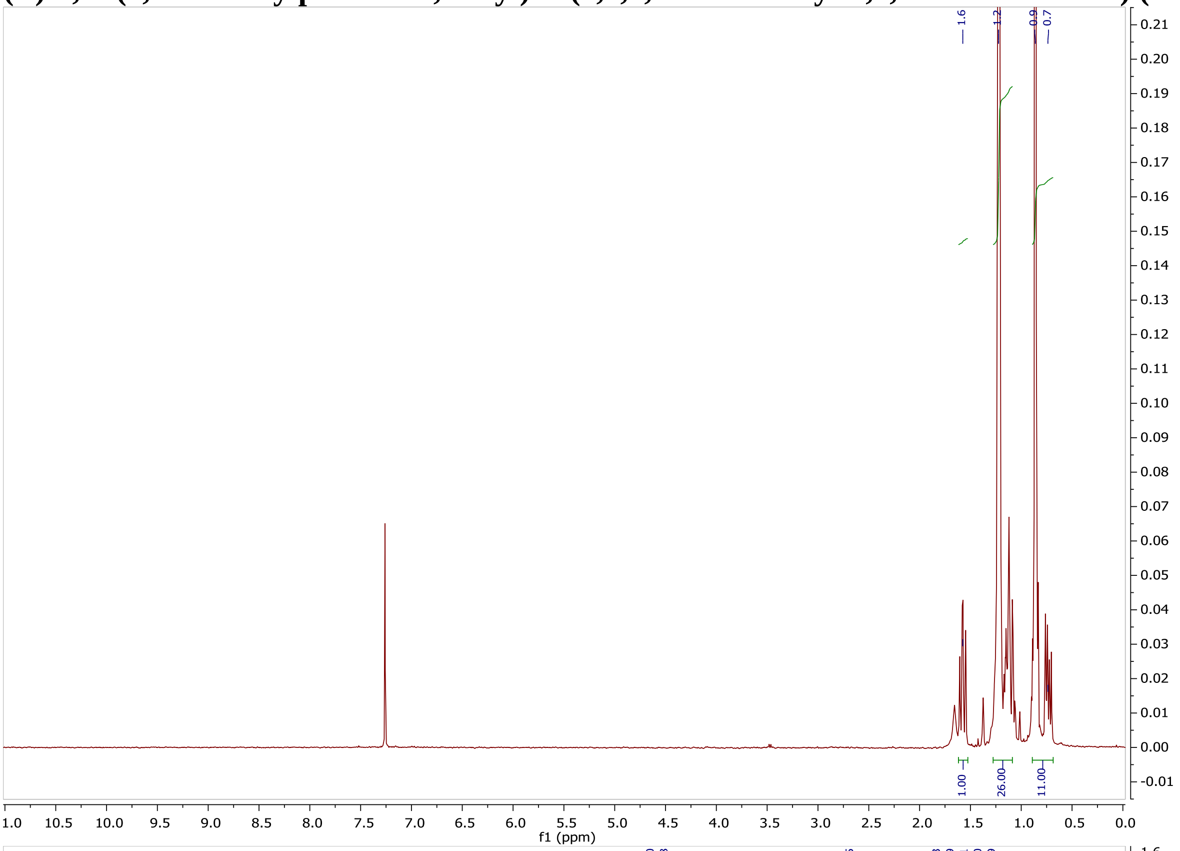

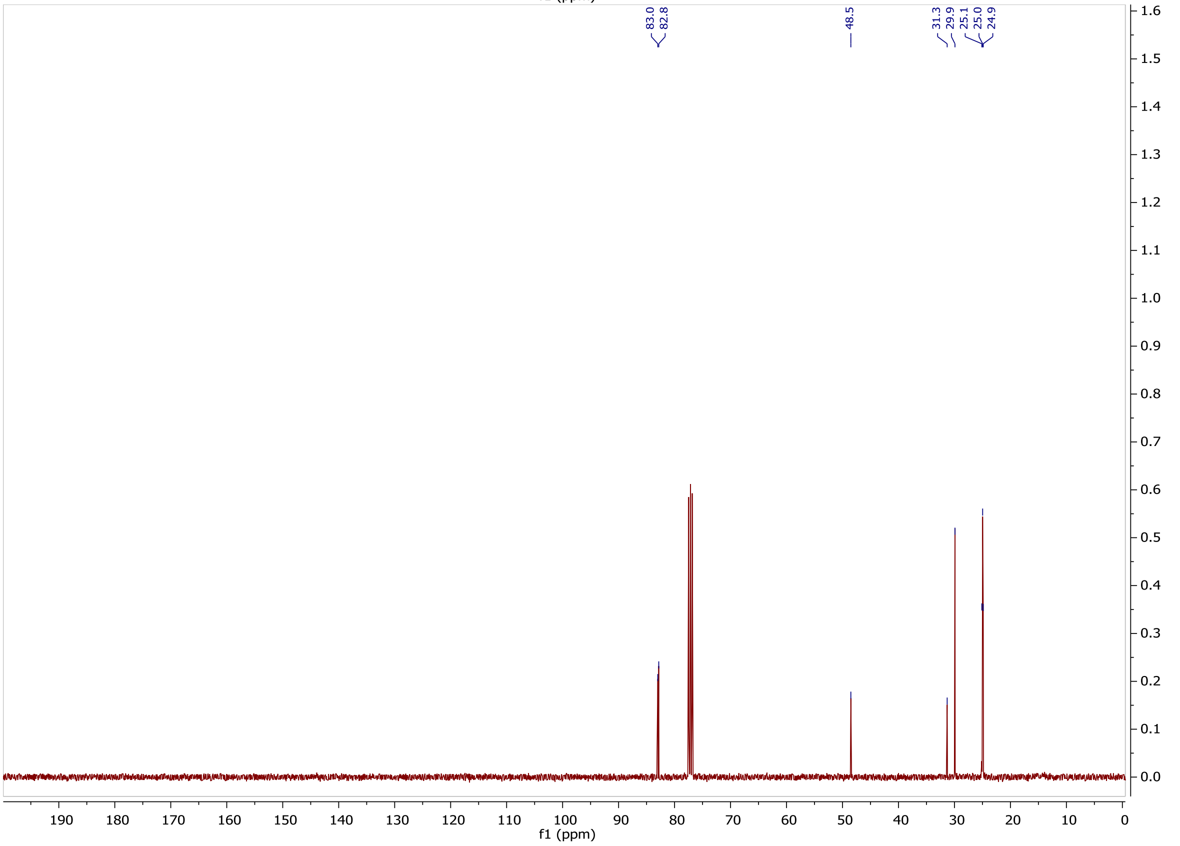

(((2*S*,4*R*)-4,5-Bis(4,4,5,5-tetramethyl-1,3,2-dioxaborolan-2-yl)pentan-2-yl)oxy)(tert-butyl)diphenylsilane (58):

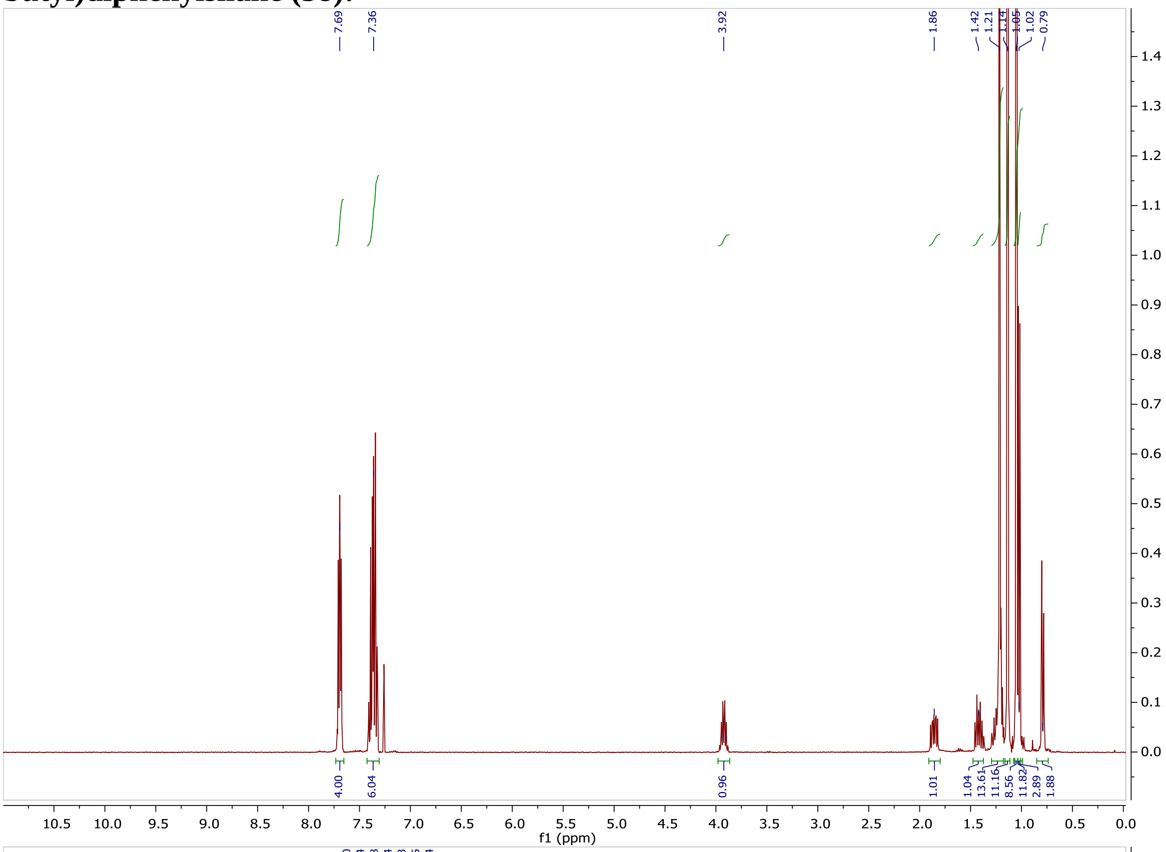

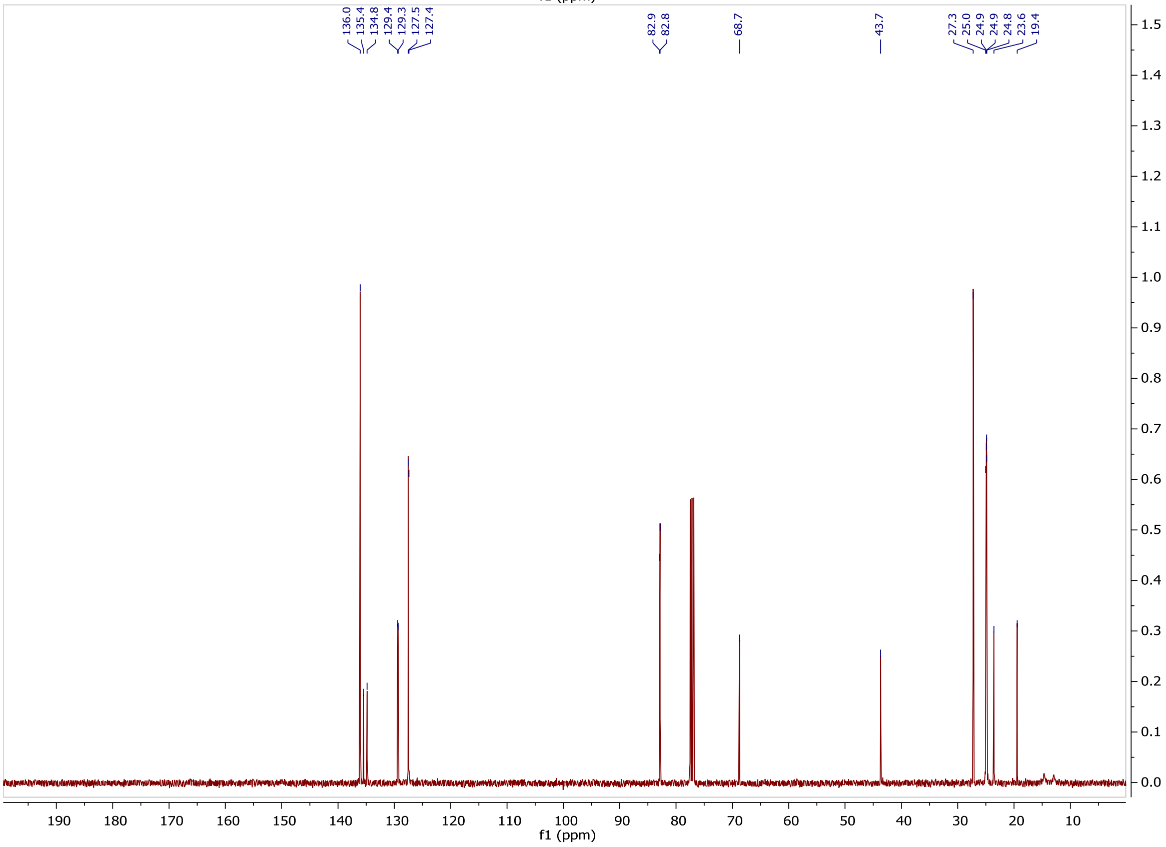

2,2'-((2*R*,4*S*)-4,8-Dimethylnon-7-ene-1,2-diyl)bis(4,4,5,5-tetramethyl-1,3,2-dioxaborolane) (59):

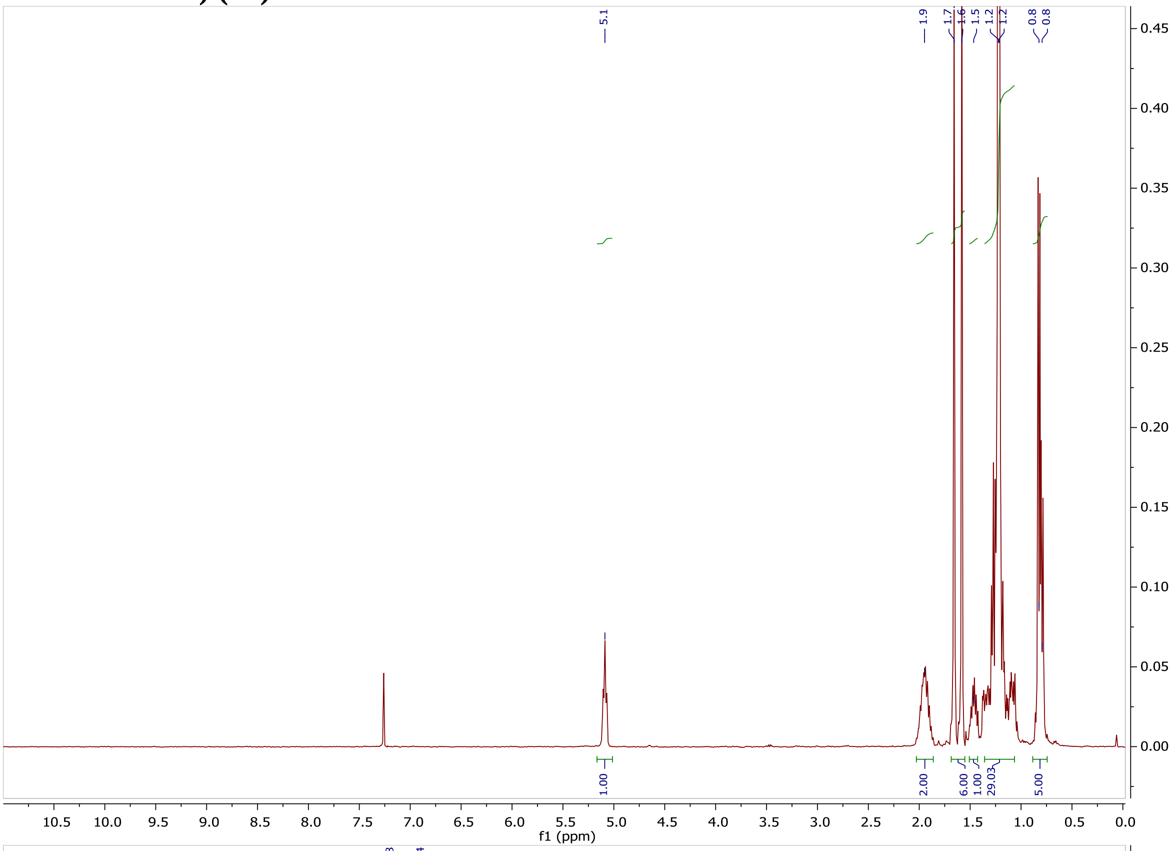

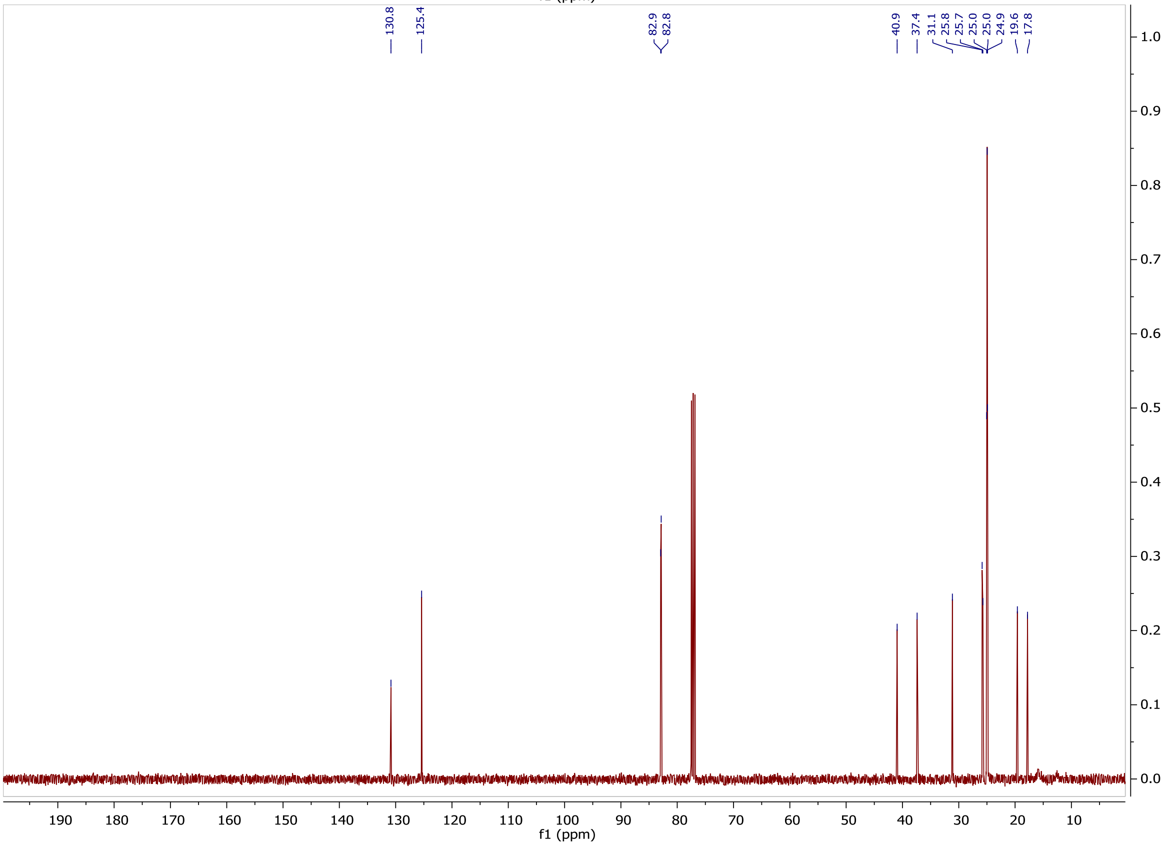

***tert*-Butyl(((2*R*,4*S*,5*S*)-2,4-dimethyl-5,6-bis(4,4,5,5-tetramethyl-1,3,2-dioxaborolan-2-yl)hexyl)oxy)diphenylsilane (60):**

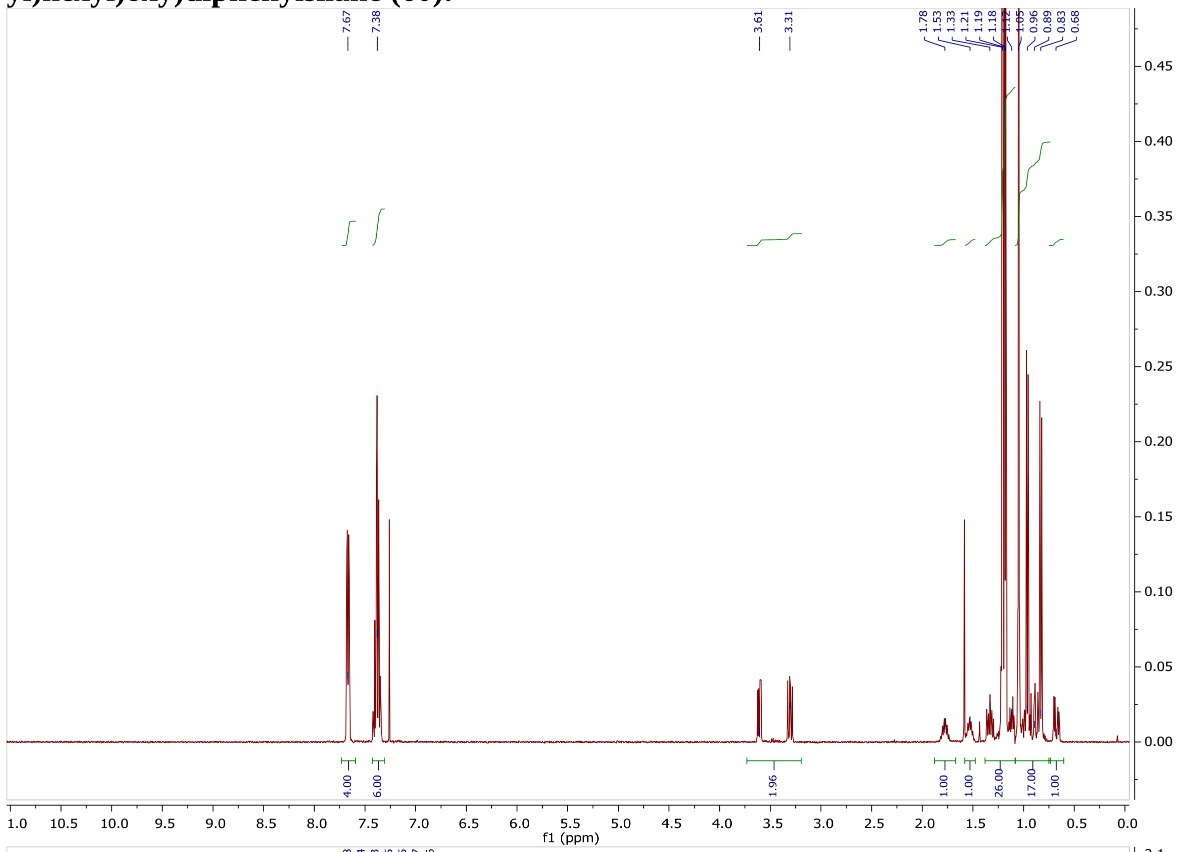

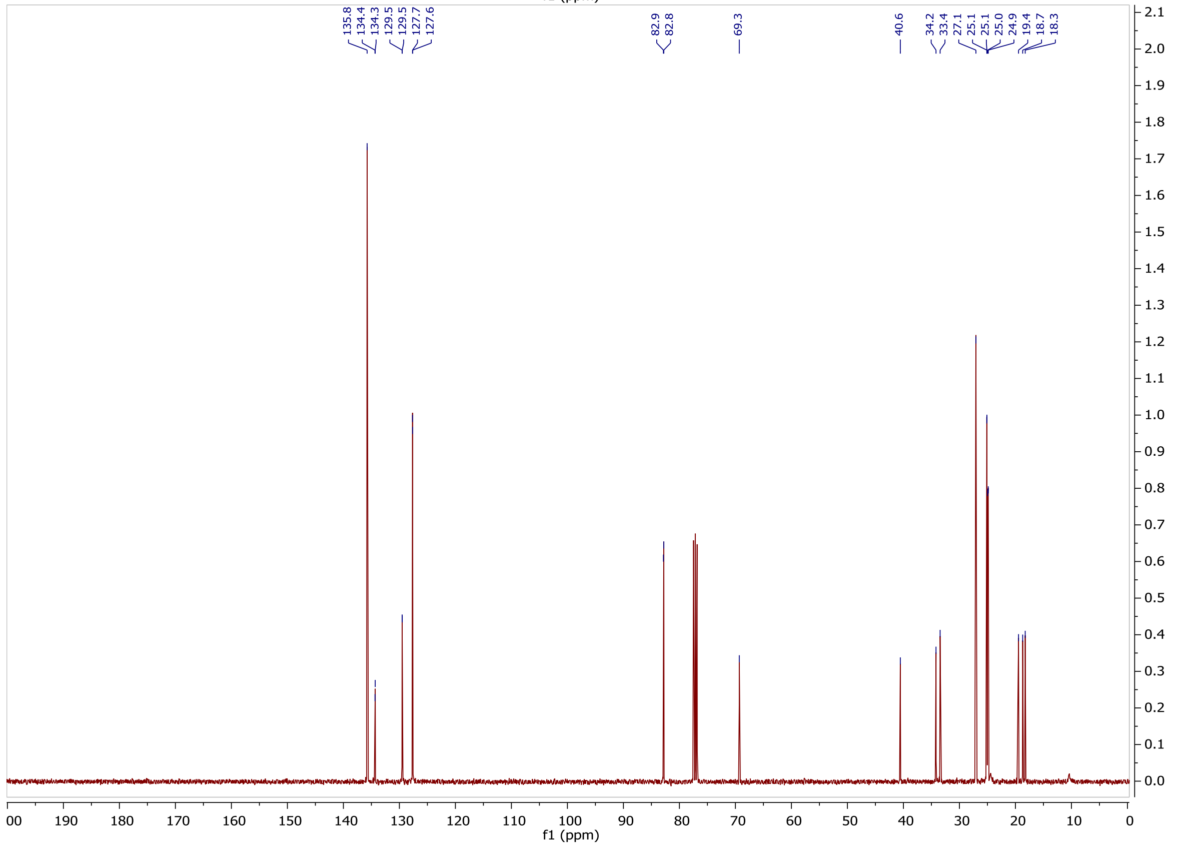

1-(But-3-en-1-yl)-5-(4-fluorophenyl)-2-isopropyl-*N*,4-diphenyl-1H-pyrrole-3-carboxamide (61):

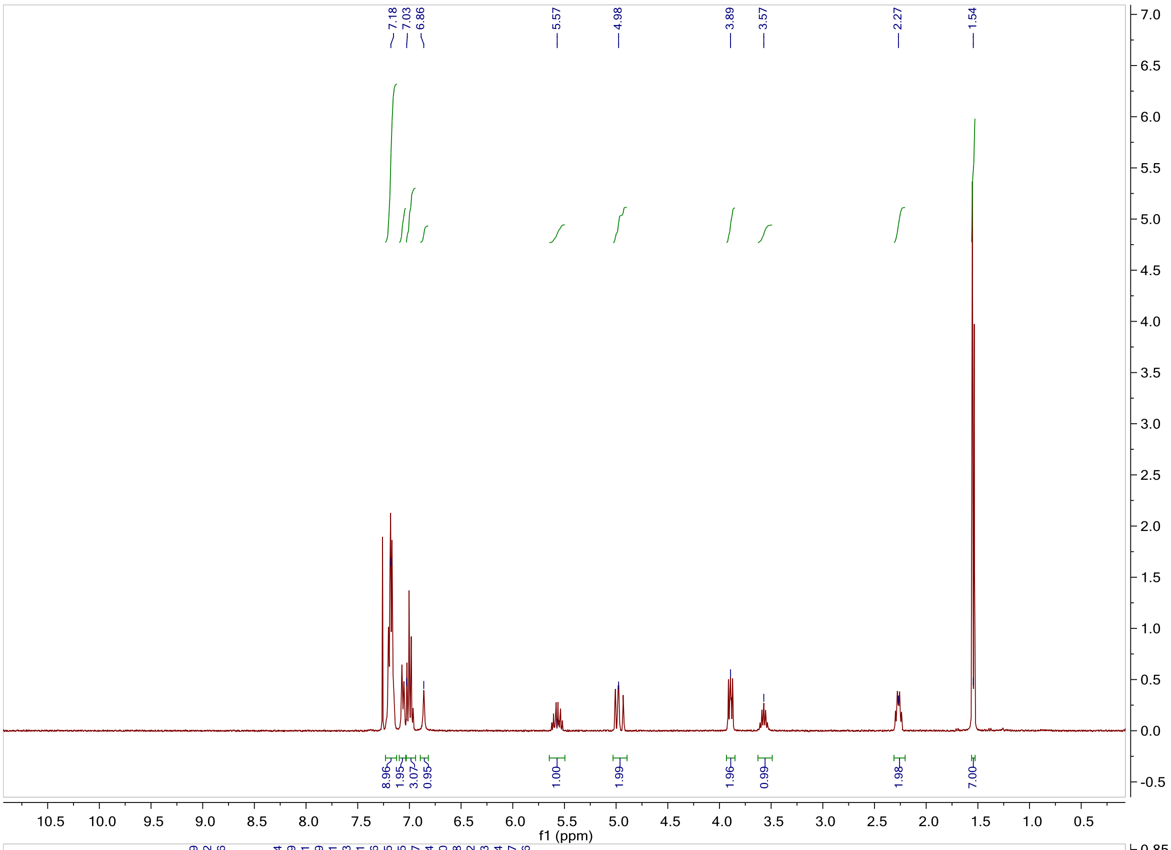

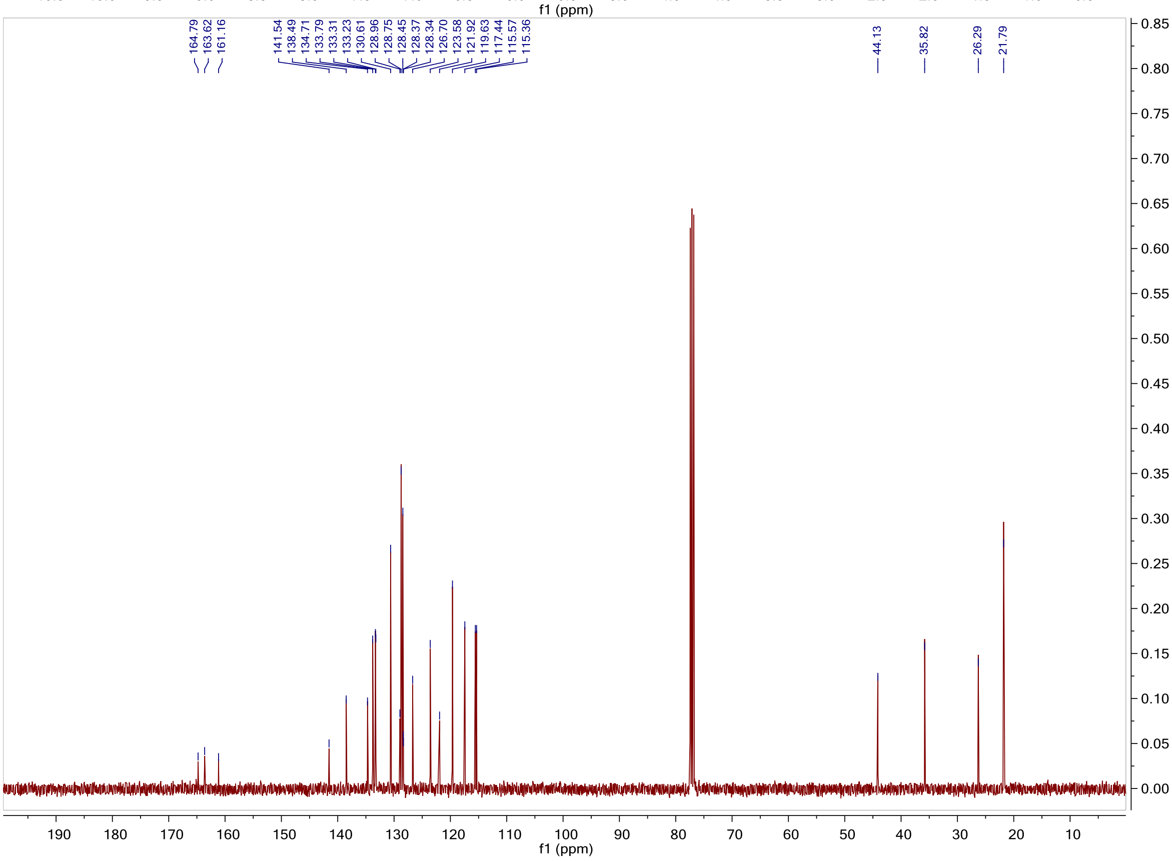

(*S*)-1-(3,4-Bis(4,4,5,5-tetramethyl-1,3,2-dioxaborolan-2-yl)butyl)-5-(4-fluorophenyl)-2-isopropyl-*N*,4-diphenyl-1H-pyrrole-3-carboxamide (62):

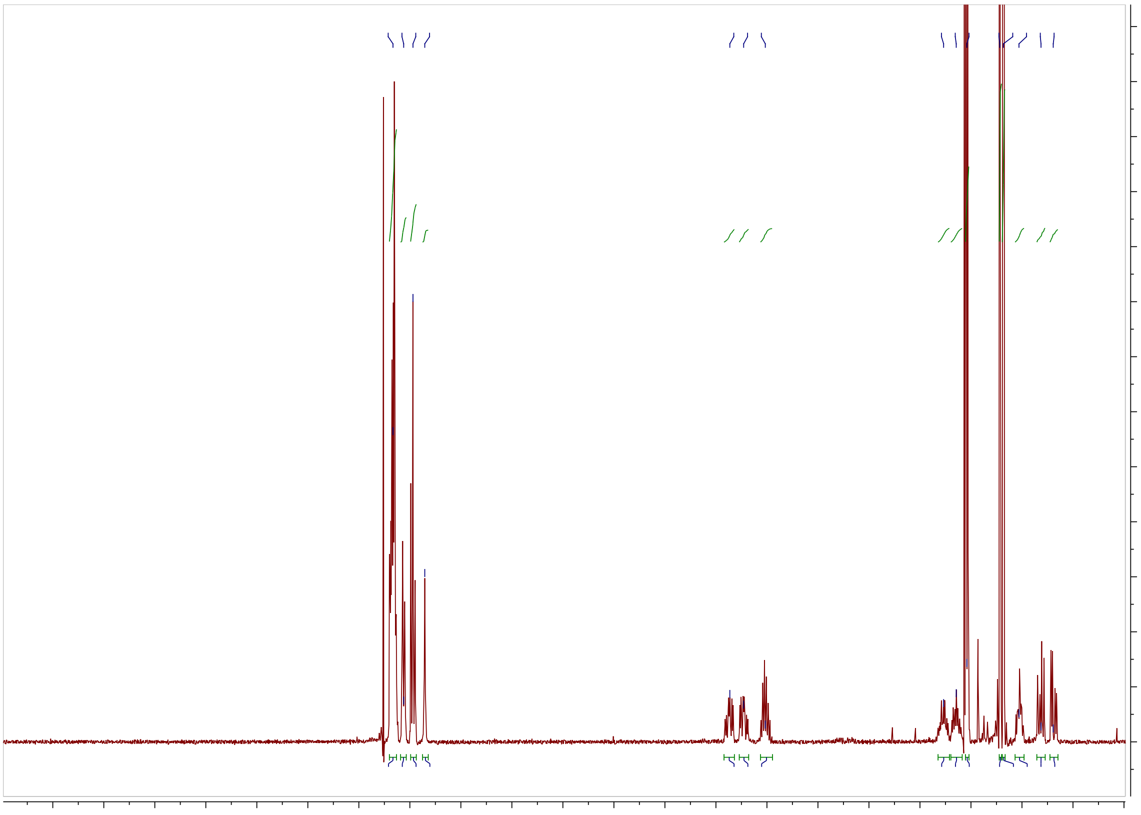

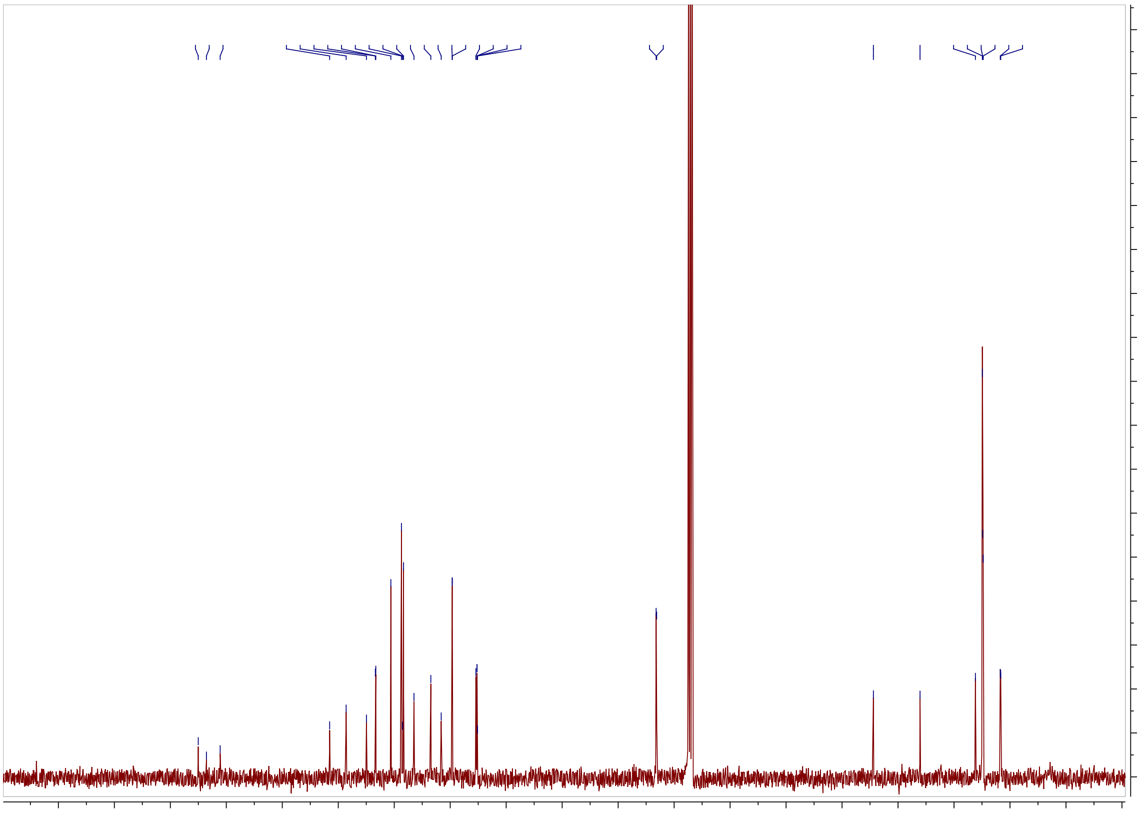

3-((*tert*-Butyldimethylsilyl)oxy)propyl 2,4,6-triisopropylbenzoate (63):

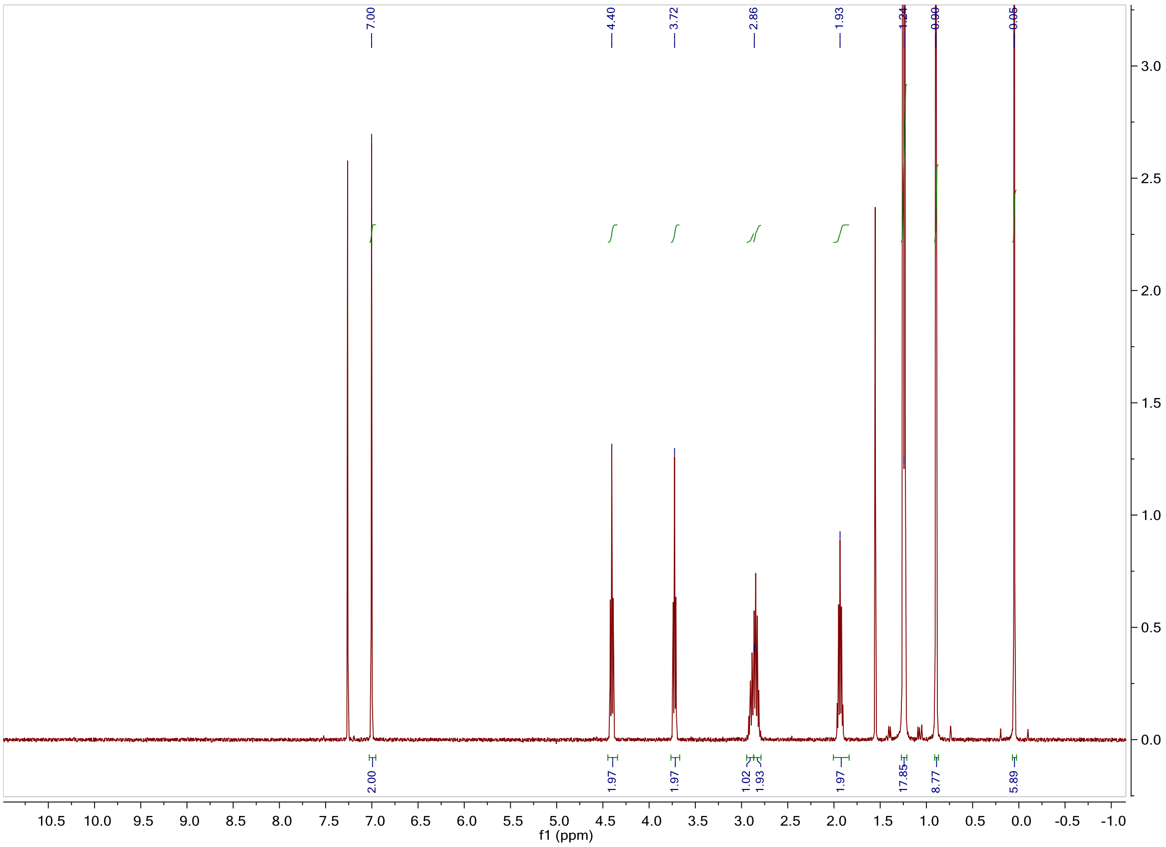

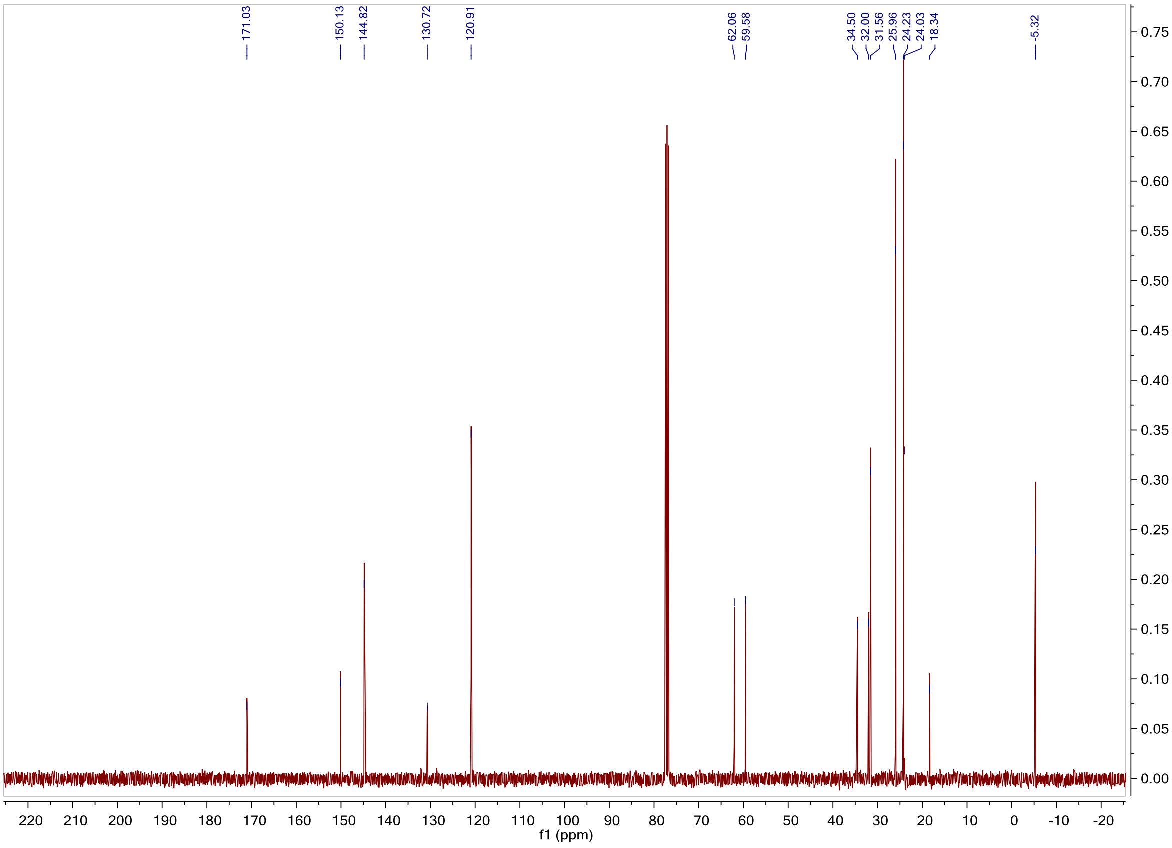

(3*R*,5*R*)-2-Methylundecane-3,5-diol (4):

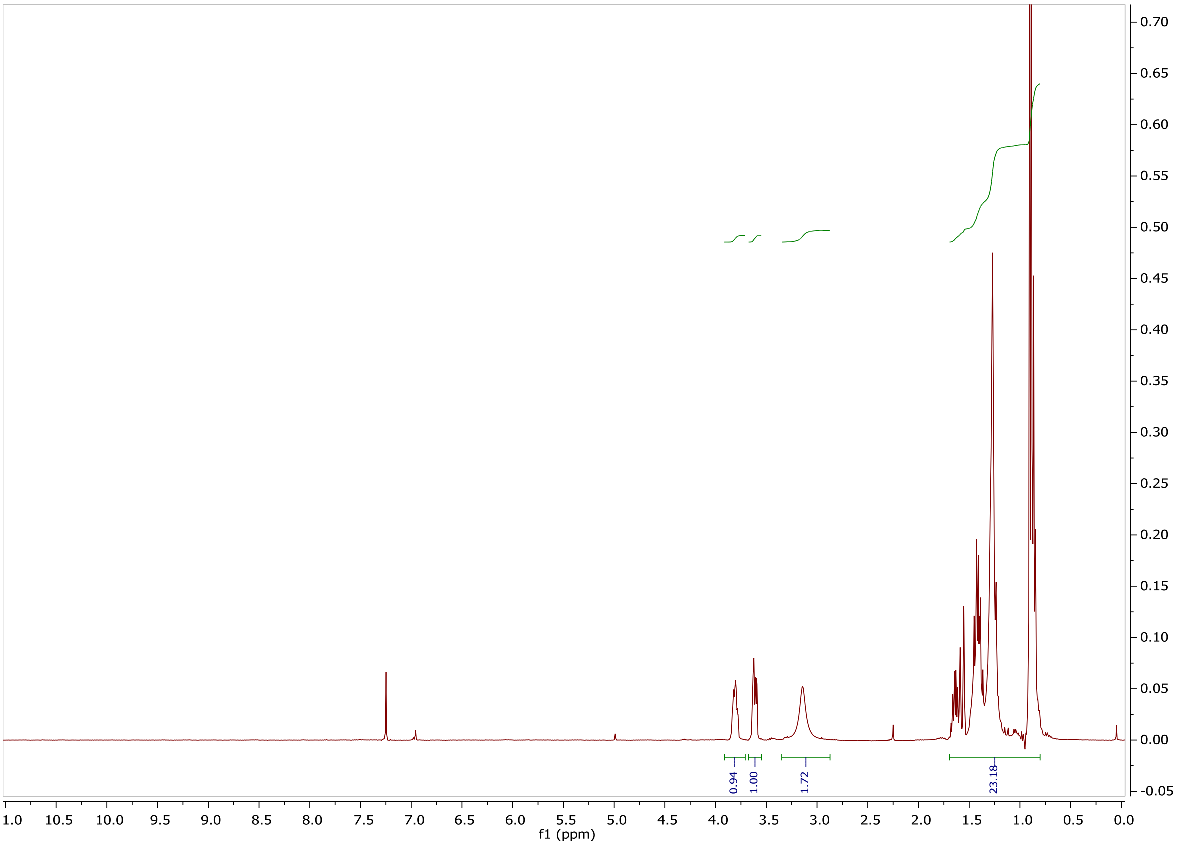

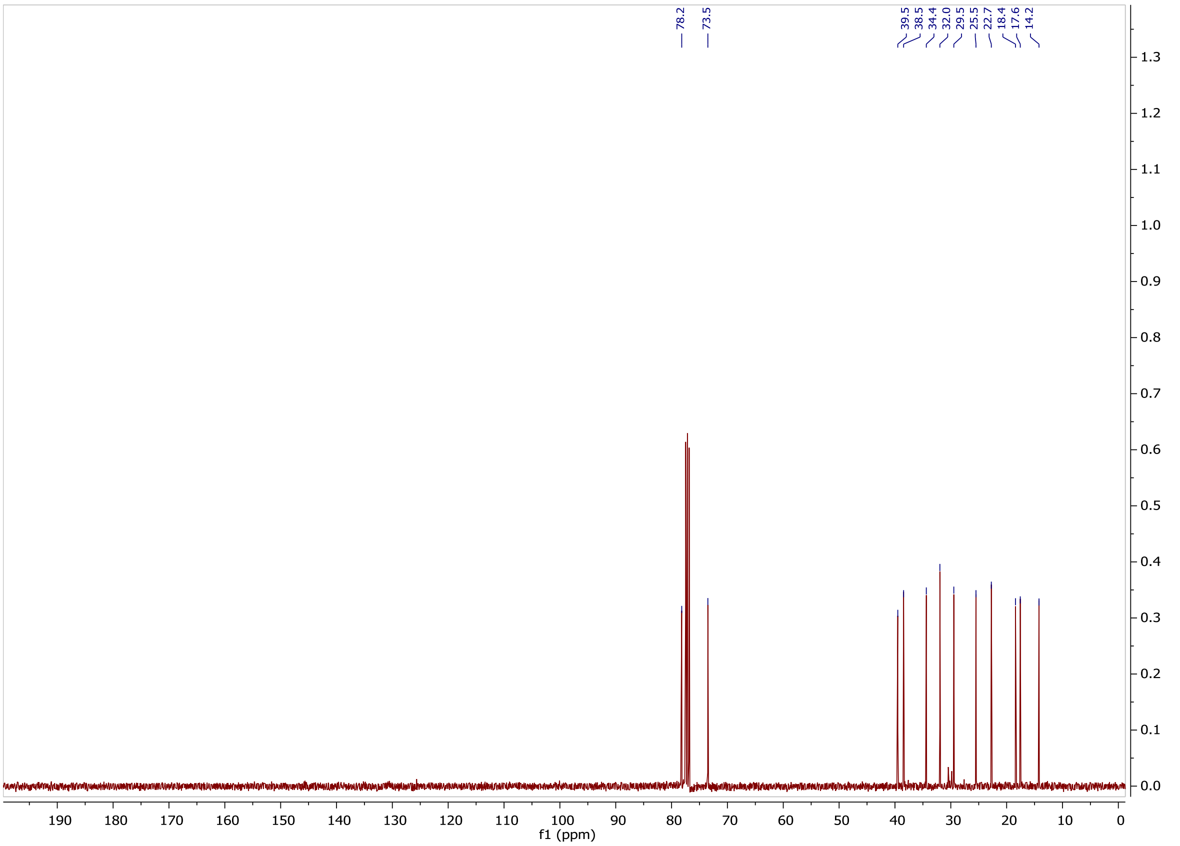

(5*S*,7*R*)-Tridec-1-ene-5,7-diol (5):

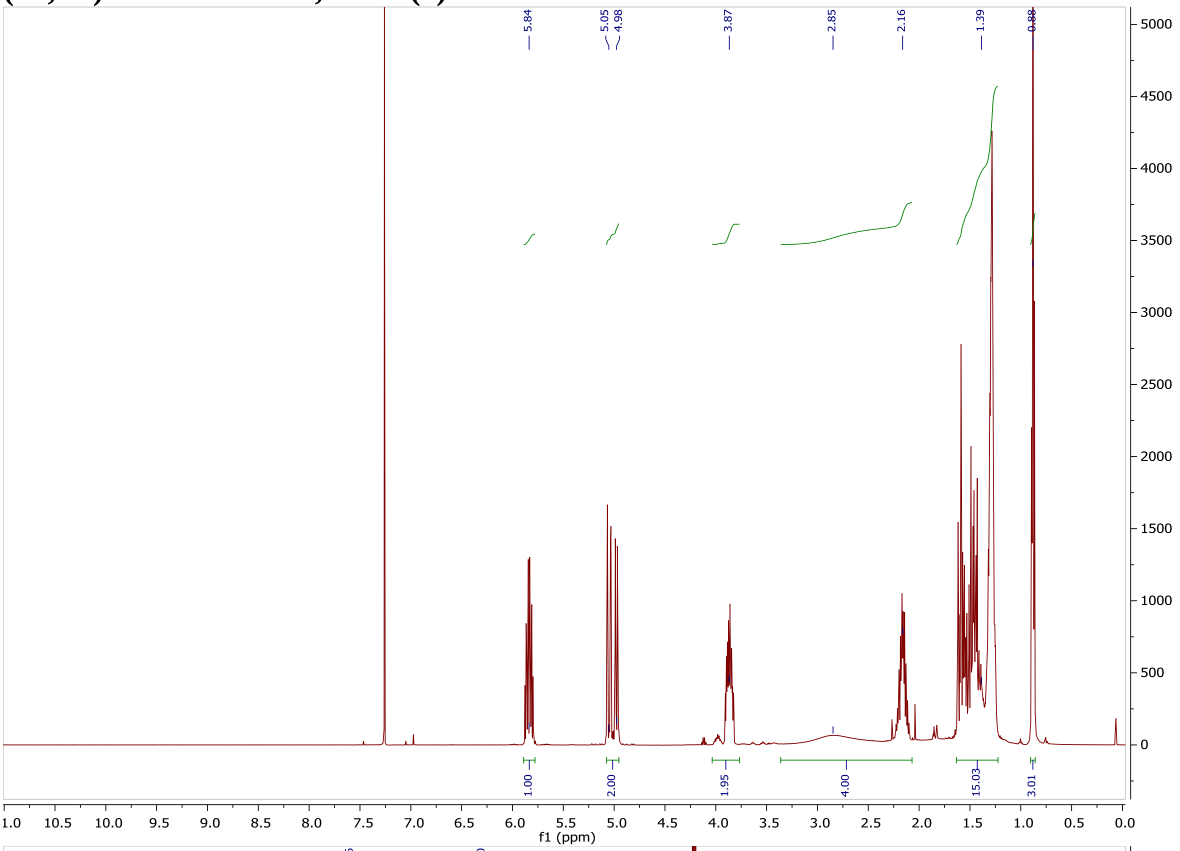

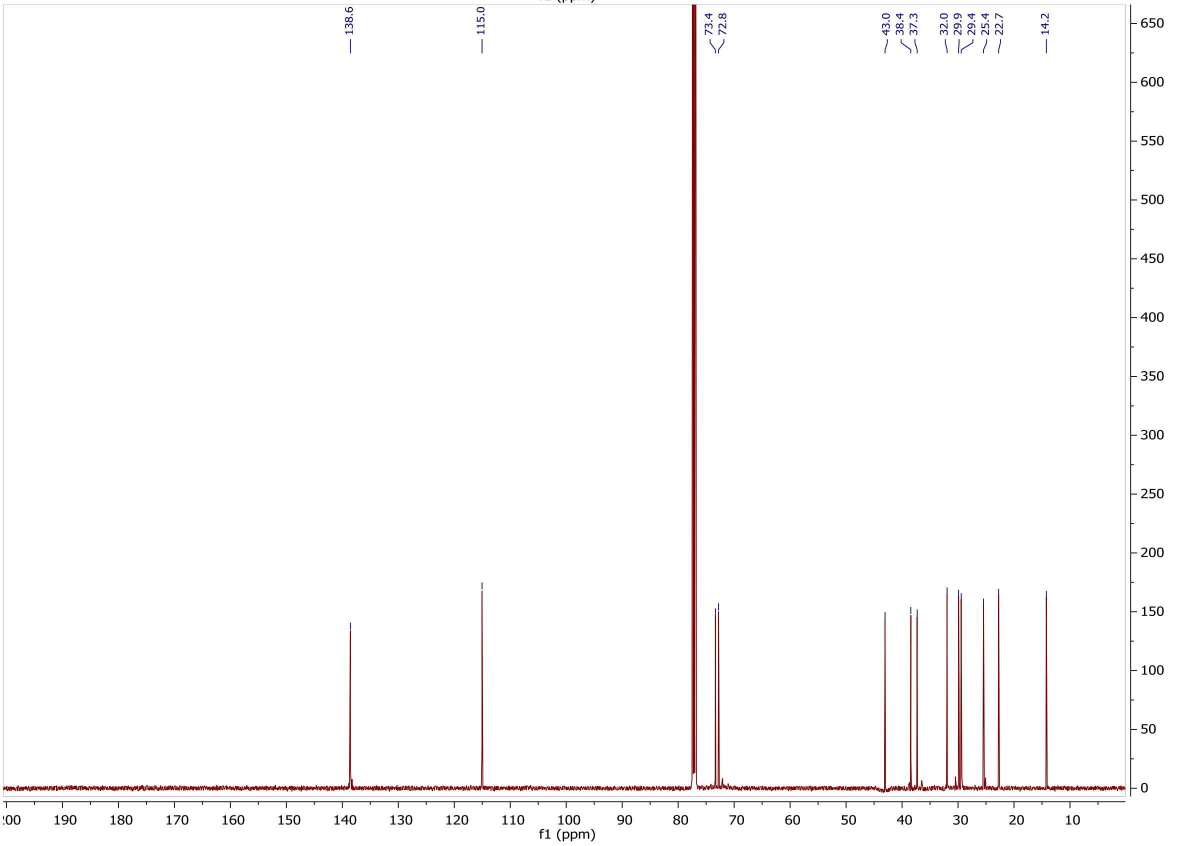

(3*S*,5*R*)-1-((*tert*-Butyldimethylsilyl)oxy)undecane-3,5-diol (6):

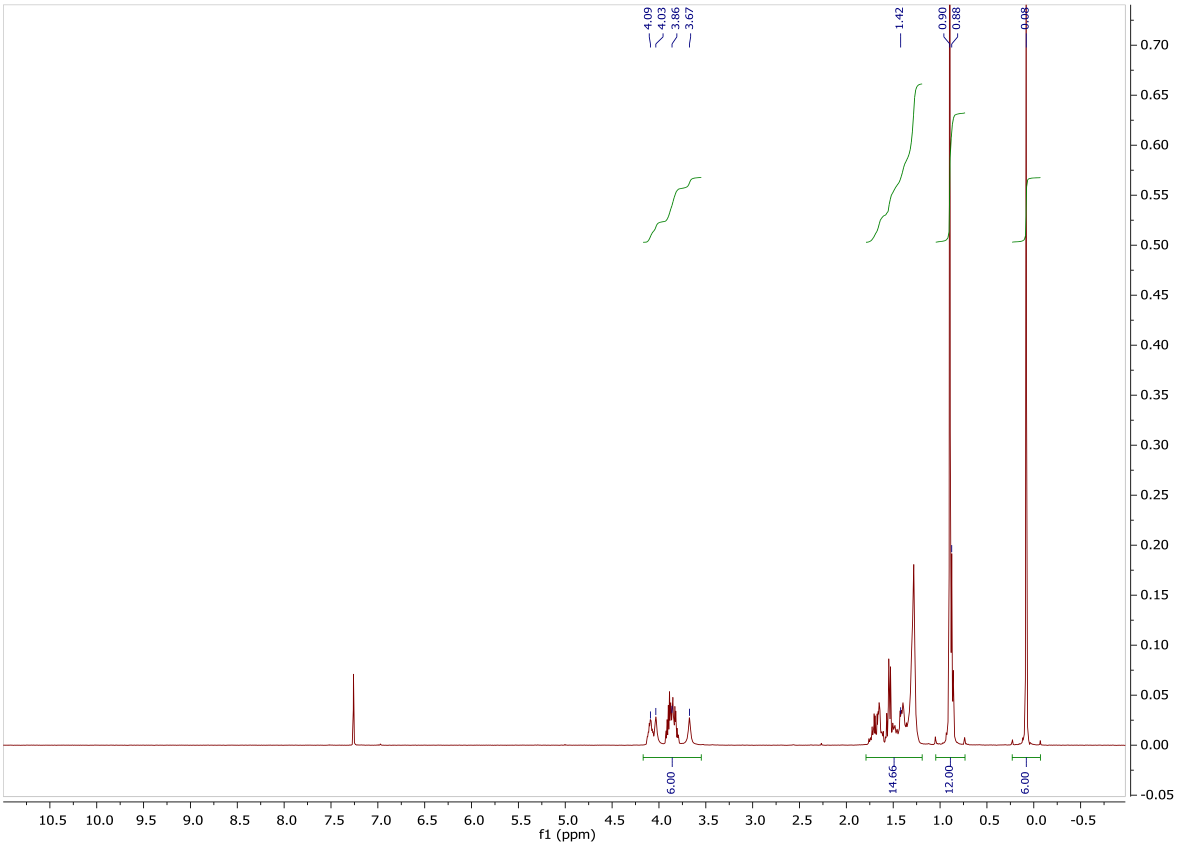

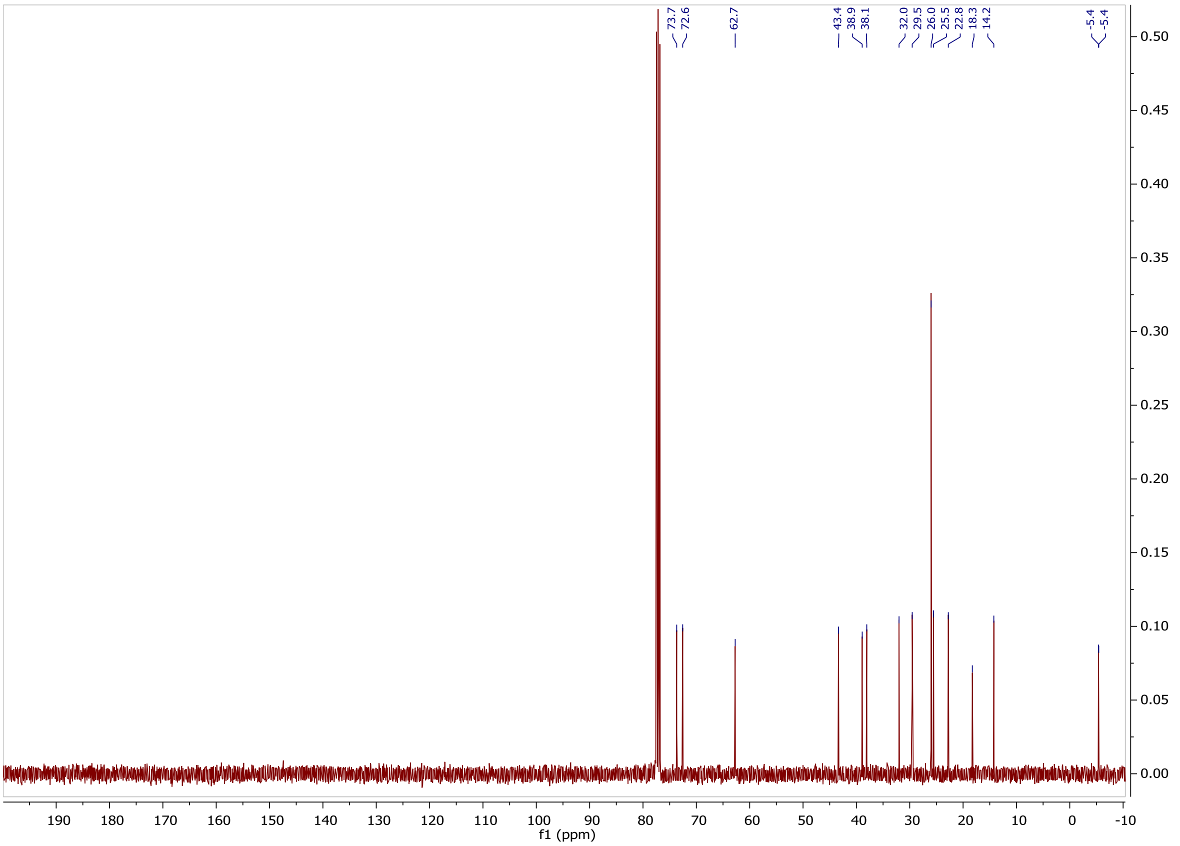

(2*S*,4*R*,5*R*,7*R*)-1-(Methoxymethoxy)-2,4-dimethyltridecane-5,7-diol (7):

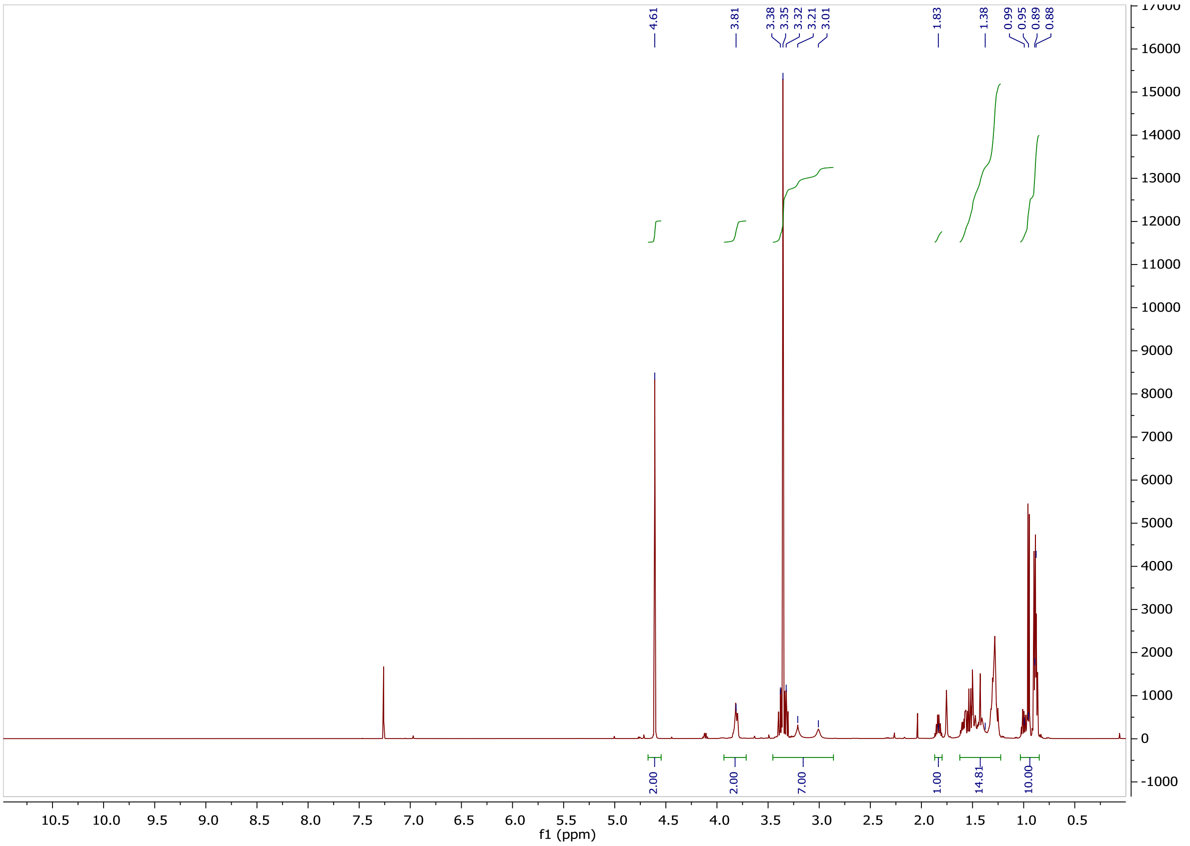

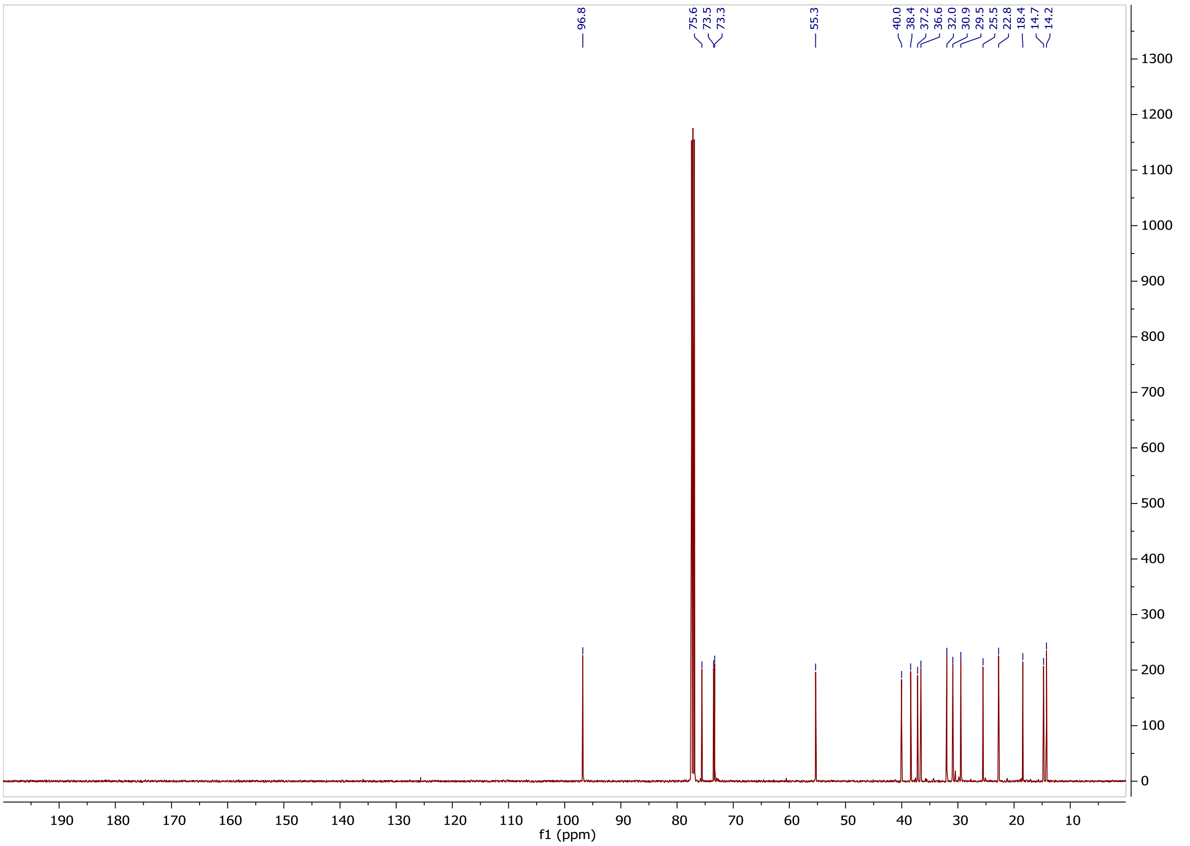

(4*R*,6*R*,*E*)-4-Methyldodec-2-ene-4,6-diol (8):

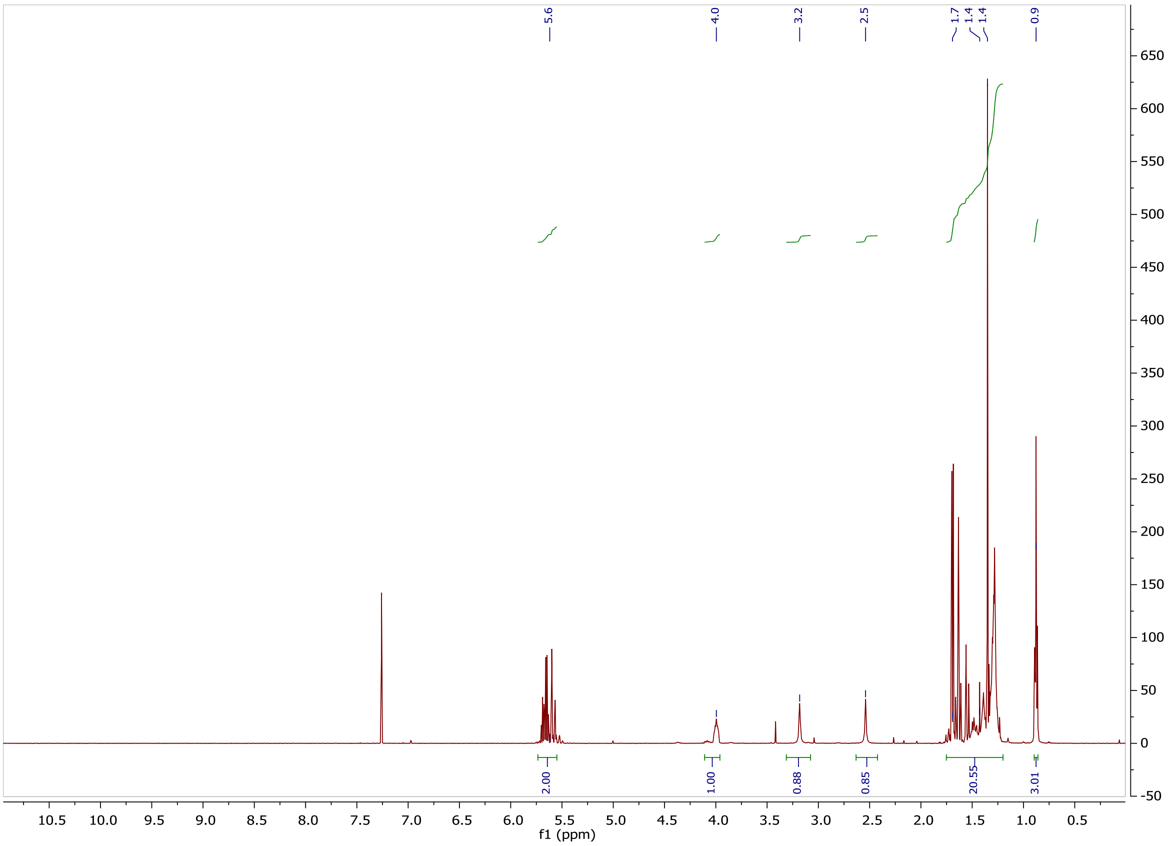

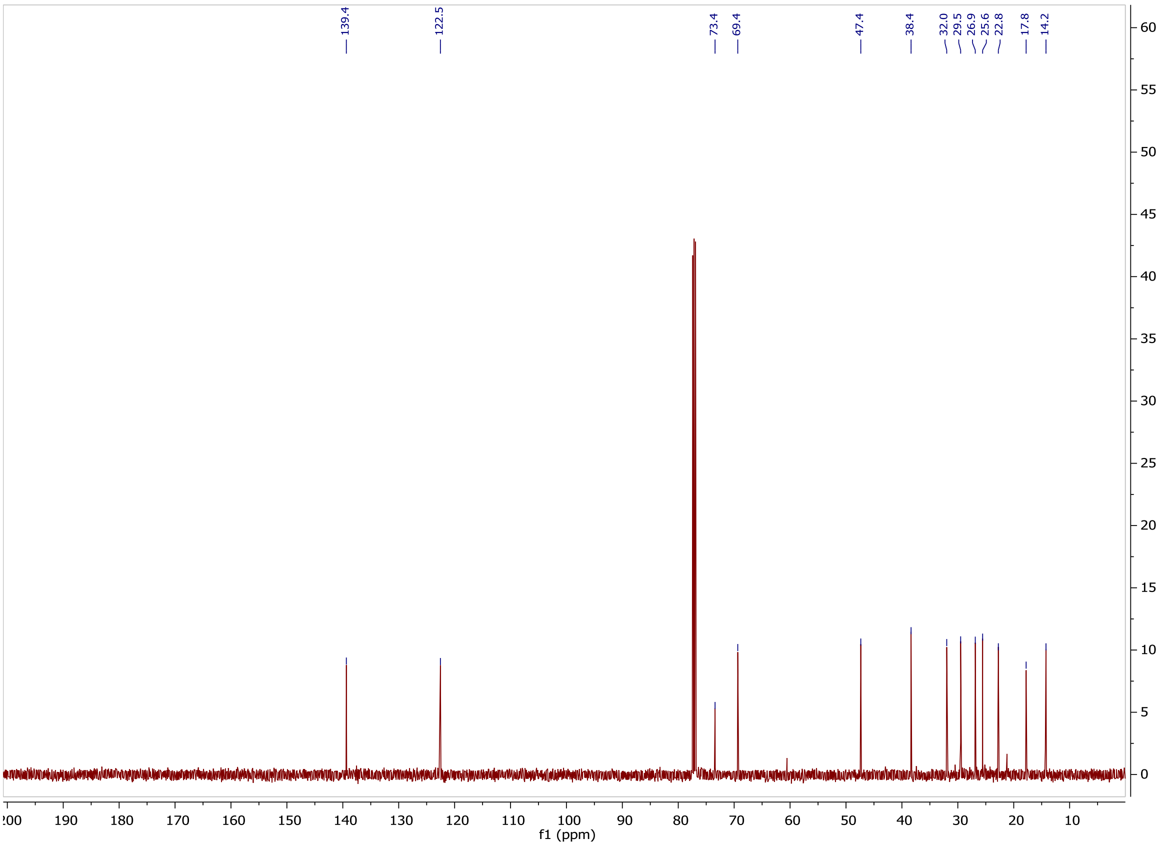

(3*R*,5*R*)-3-Methyl-1-phenylundecane-3,5-diol (9):

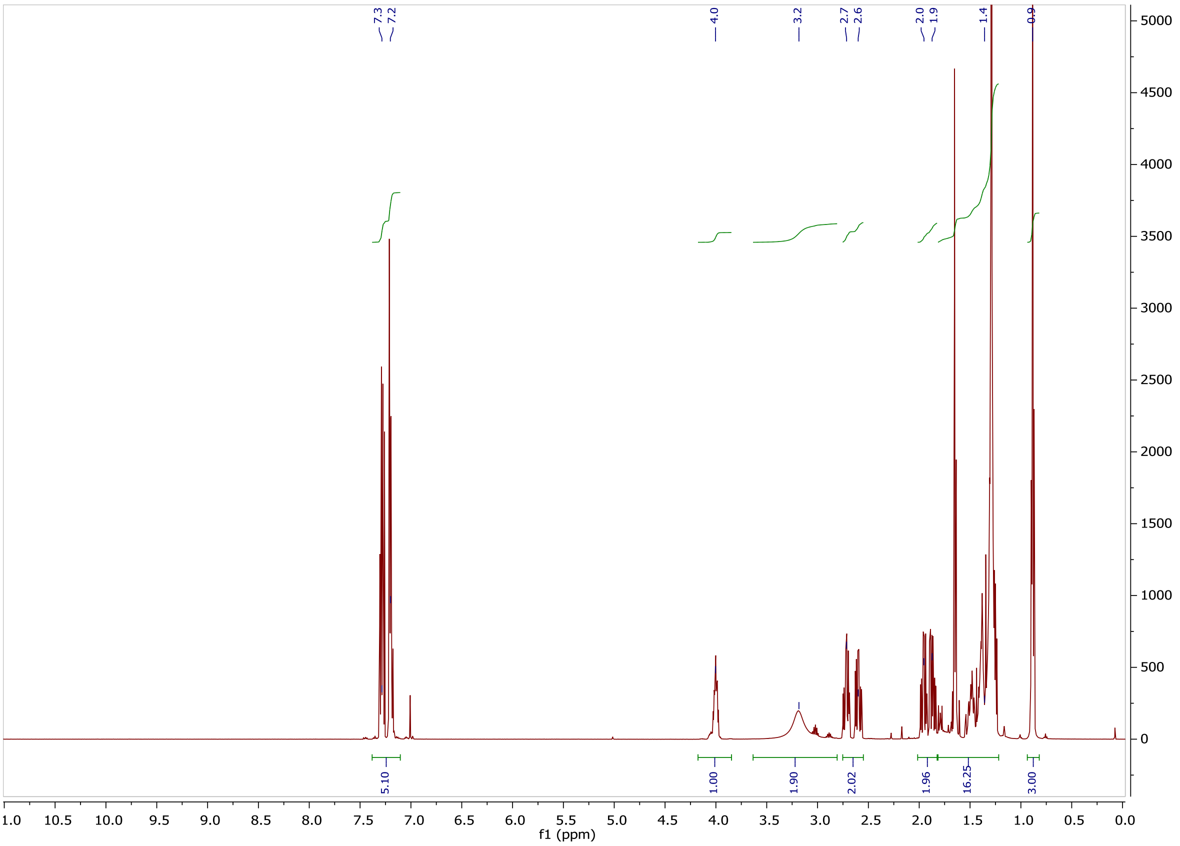

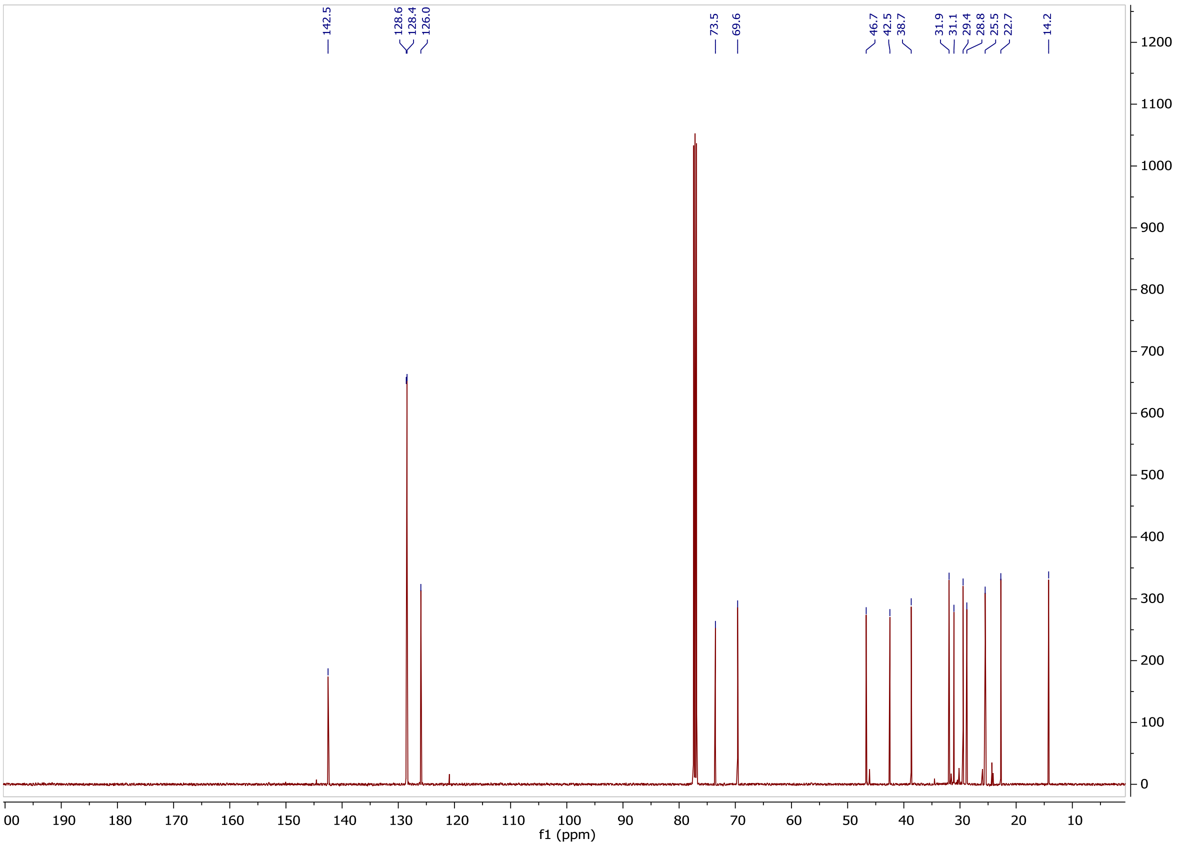

(2*R*,4*R*)-2-(4-Methoxyphenyl)decane-2,4-diol (10):

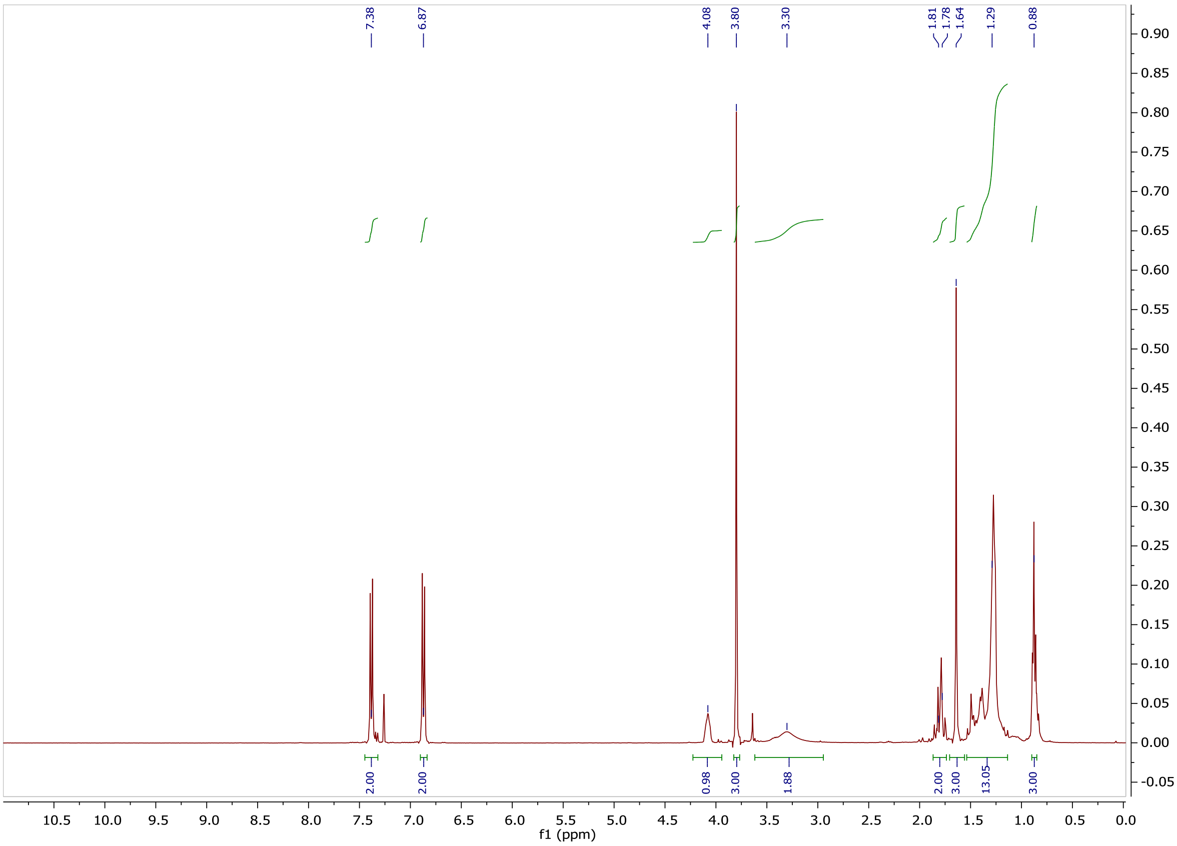

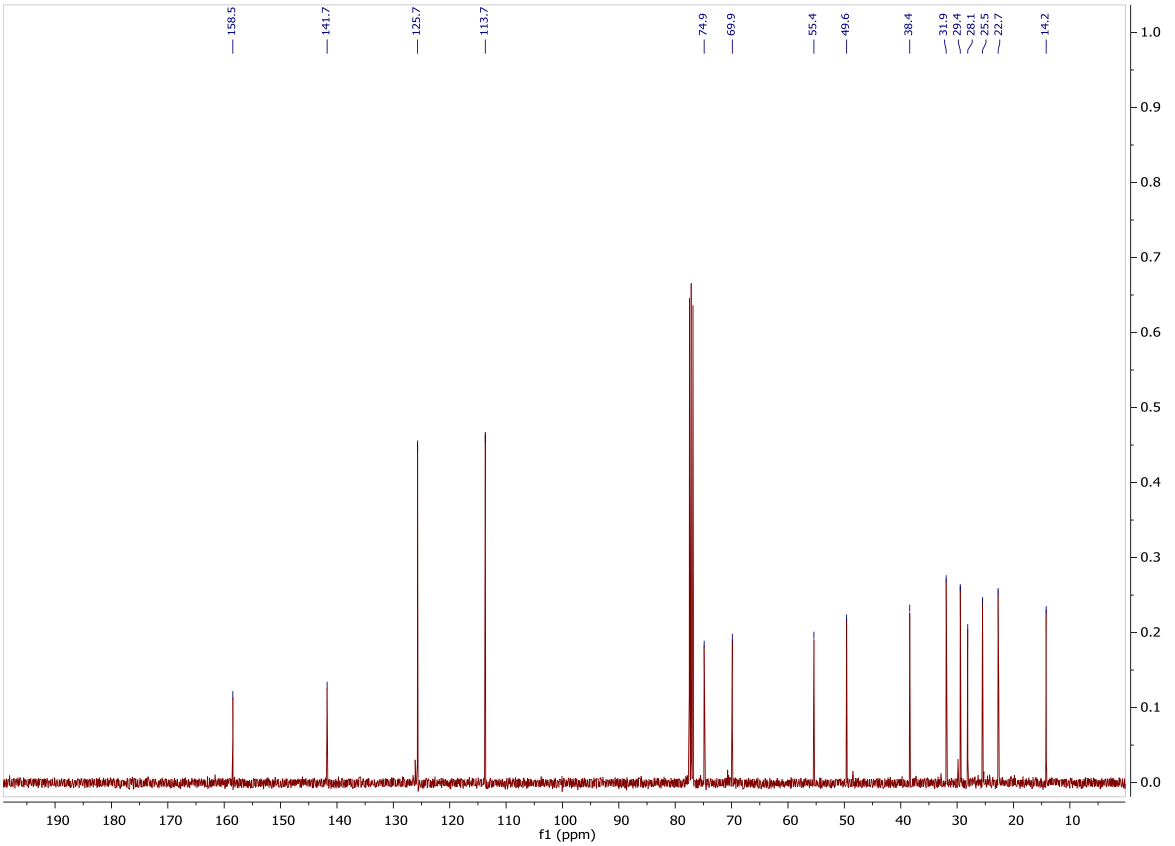

(2*R*,4*R*)-2-Phenyldecane-2,4-diol (11):

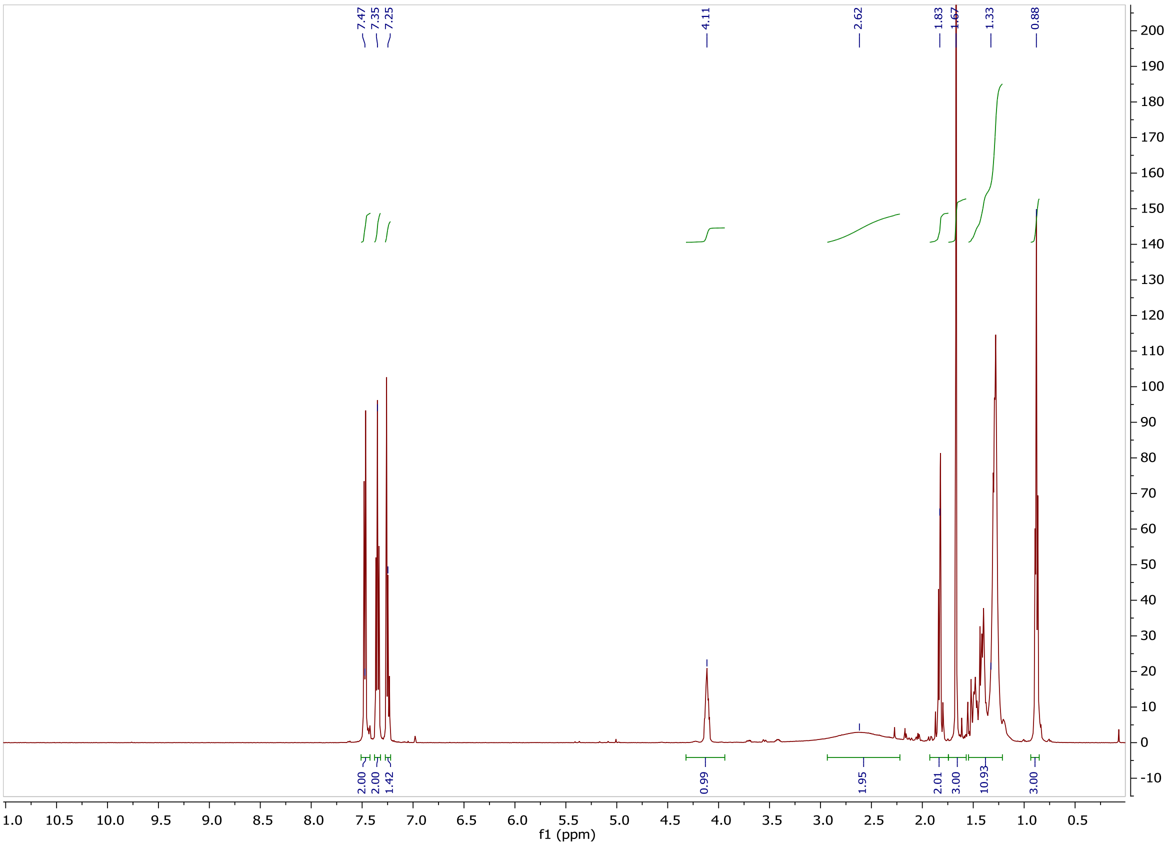

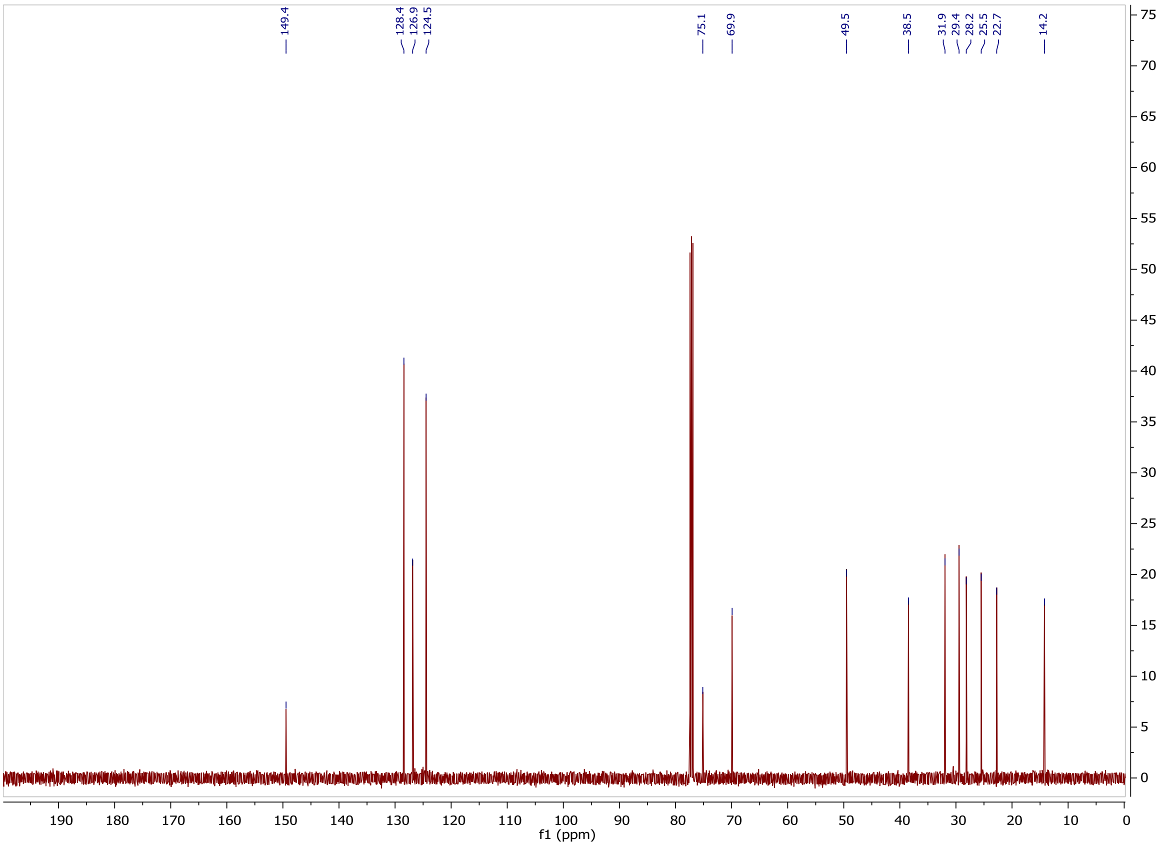

(2*R*,4*R*)-2-(4-Fluorophenyl)decane-2,4-diol (12):

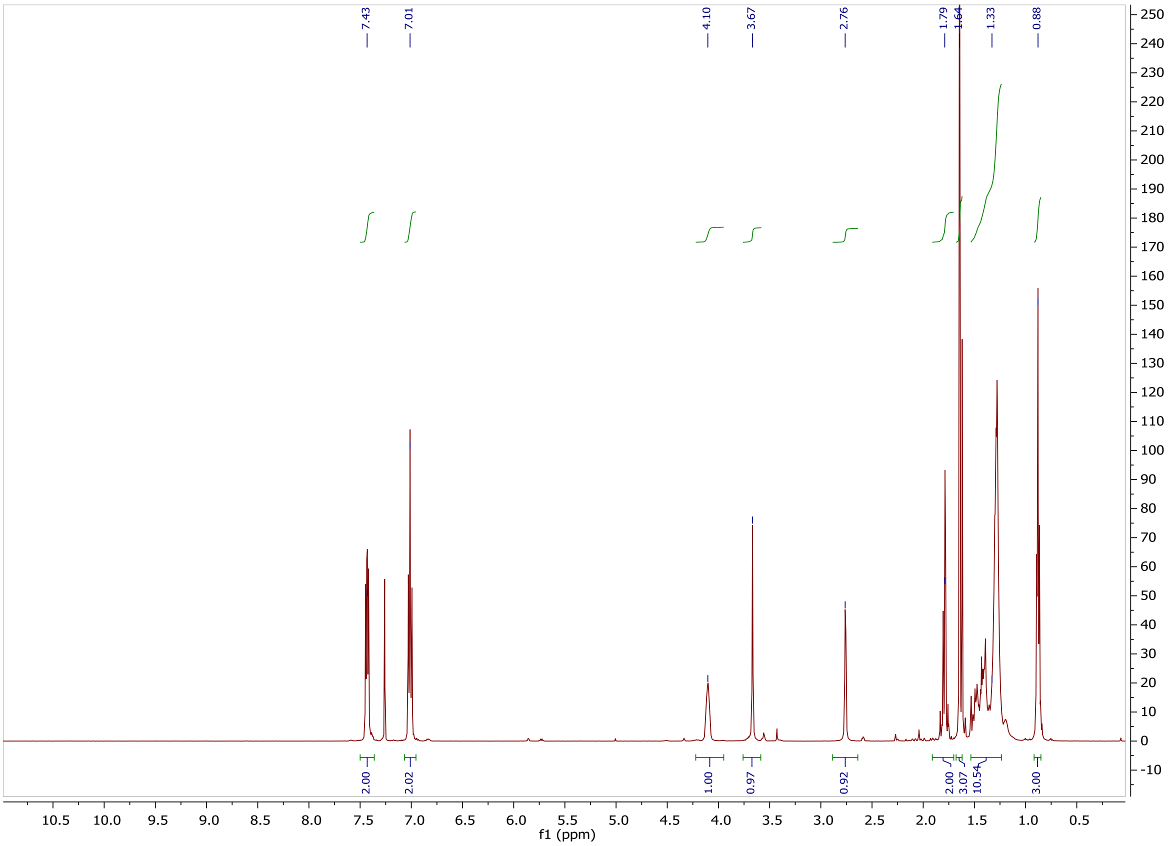

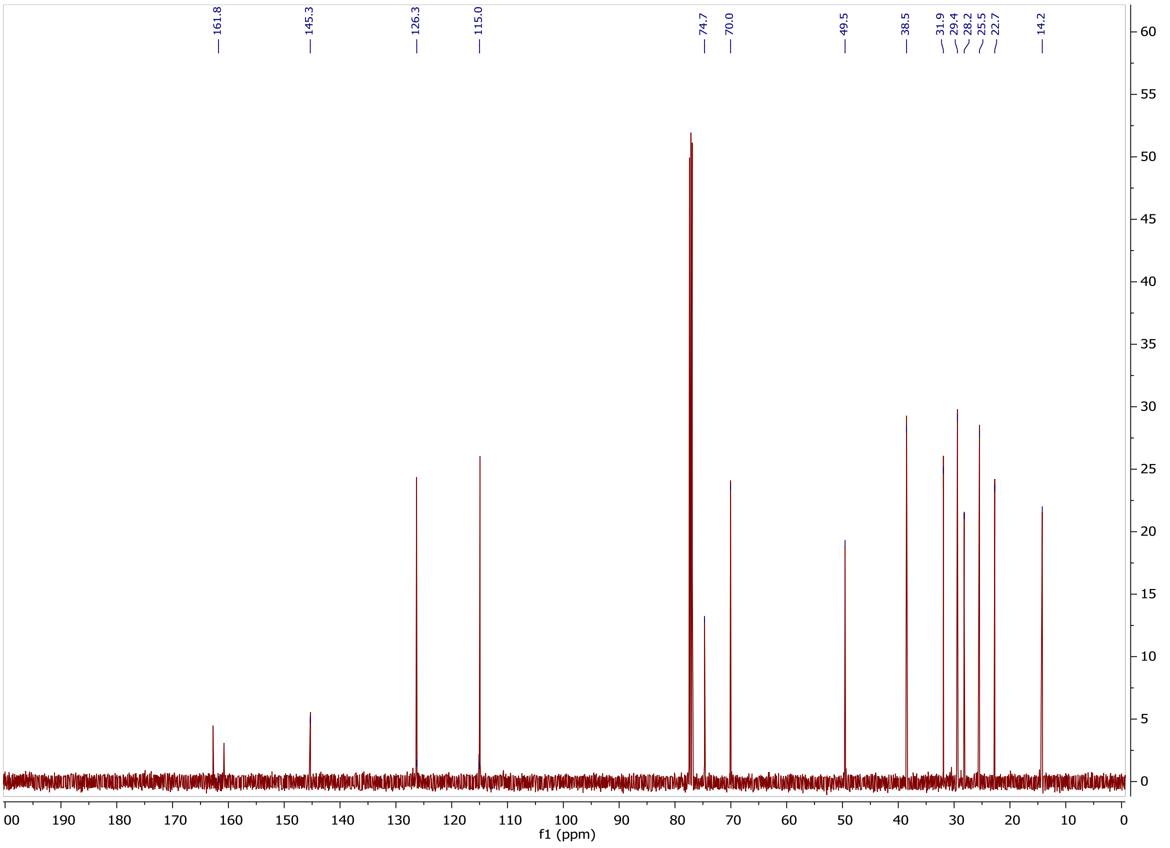

tert-Butyl (6S,8R)-6,8-dihydroxy-8-phenylnonanoate (13):

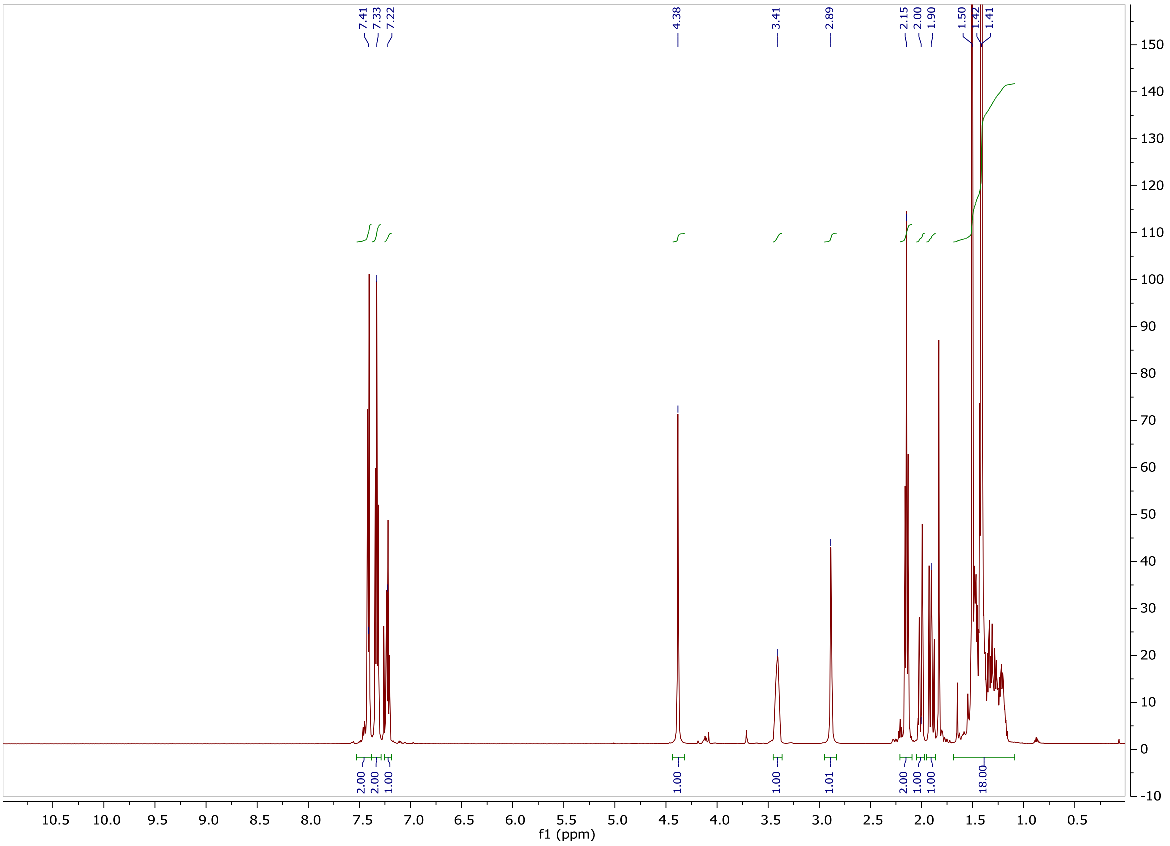

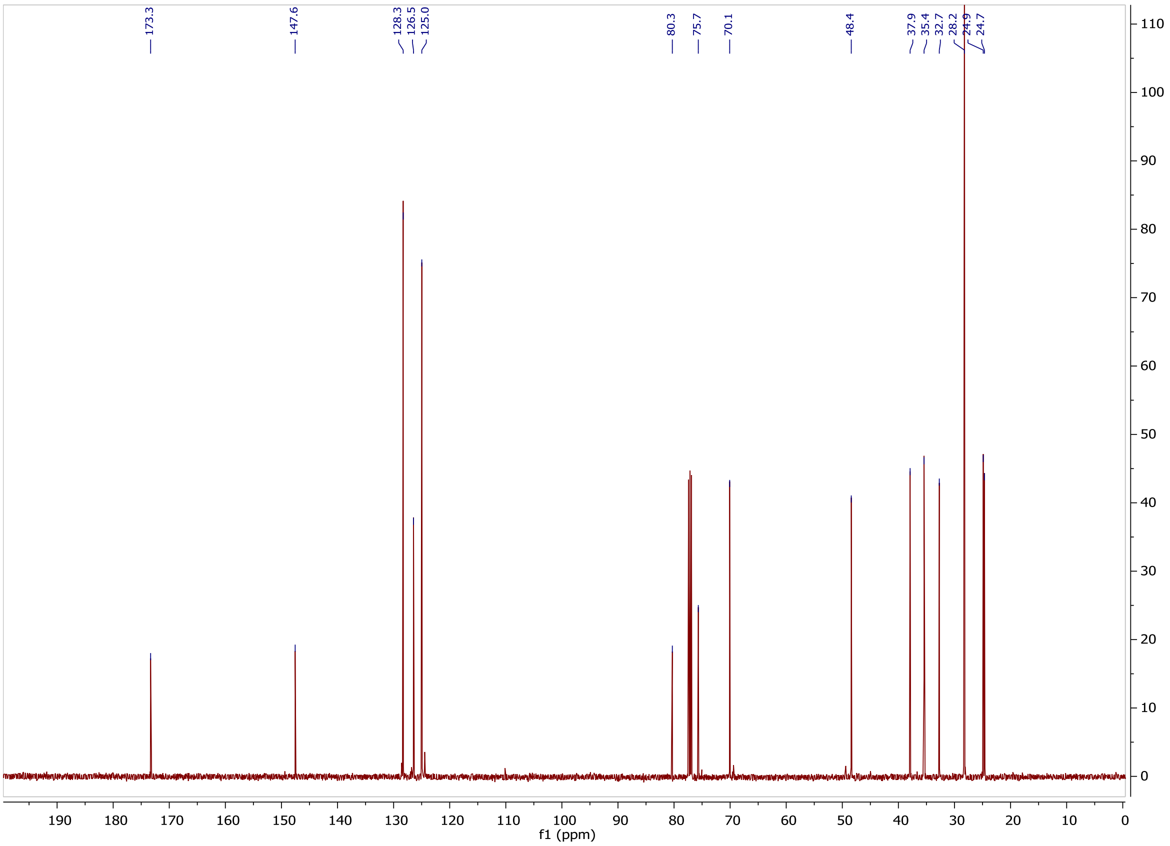

(1*R*,3*R*)-1-(4-Methoxyphenyl)-3-phenylbutane-1,3-diol (14):

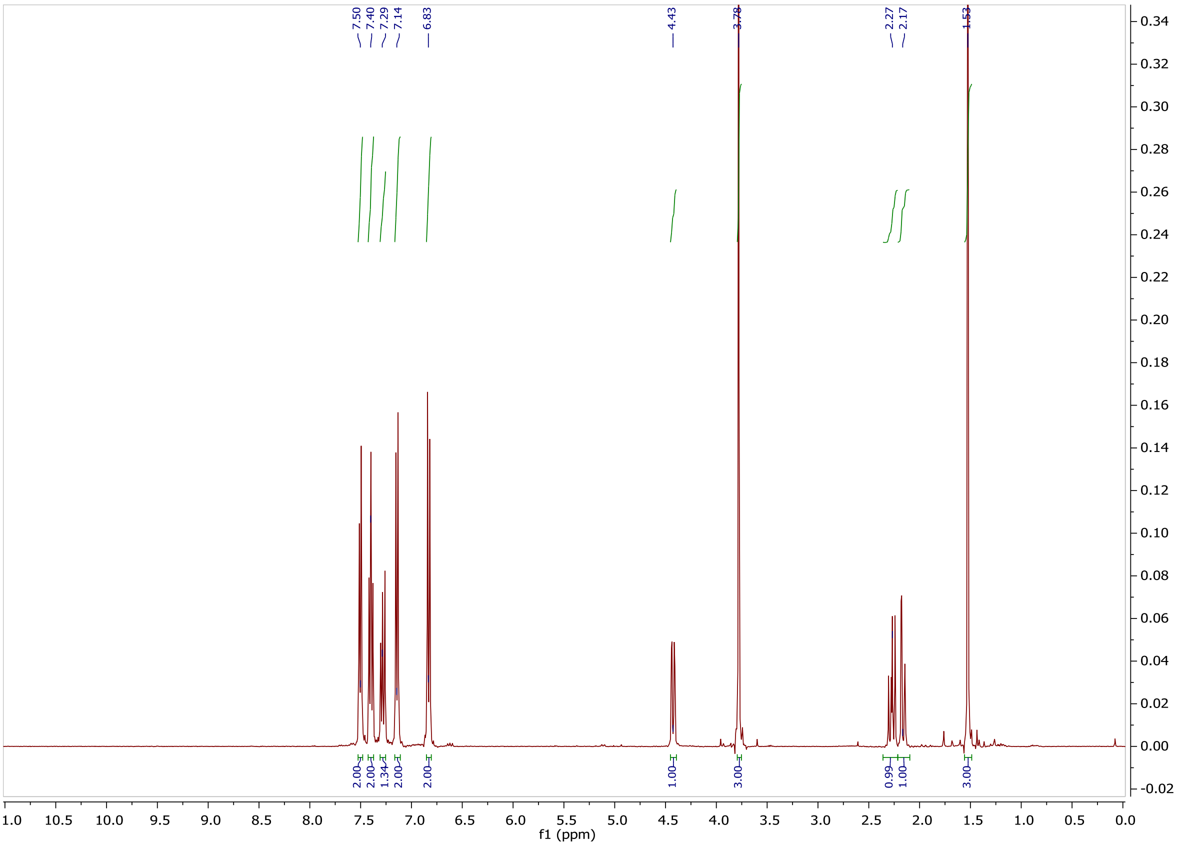

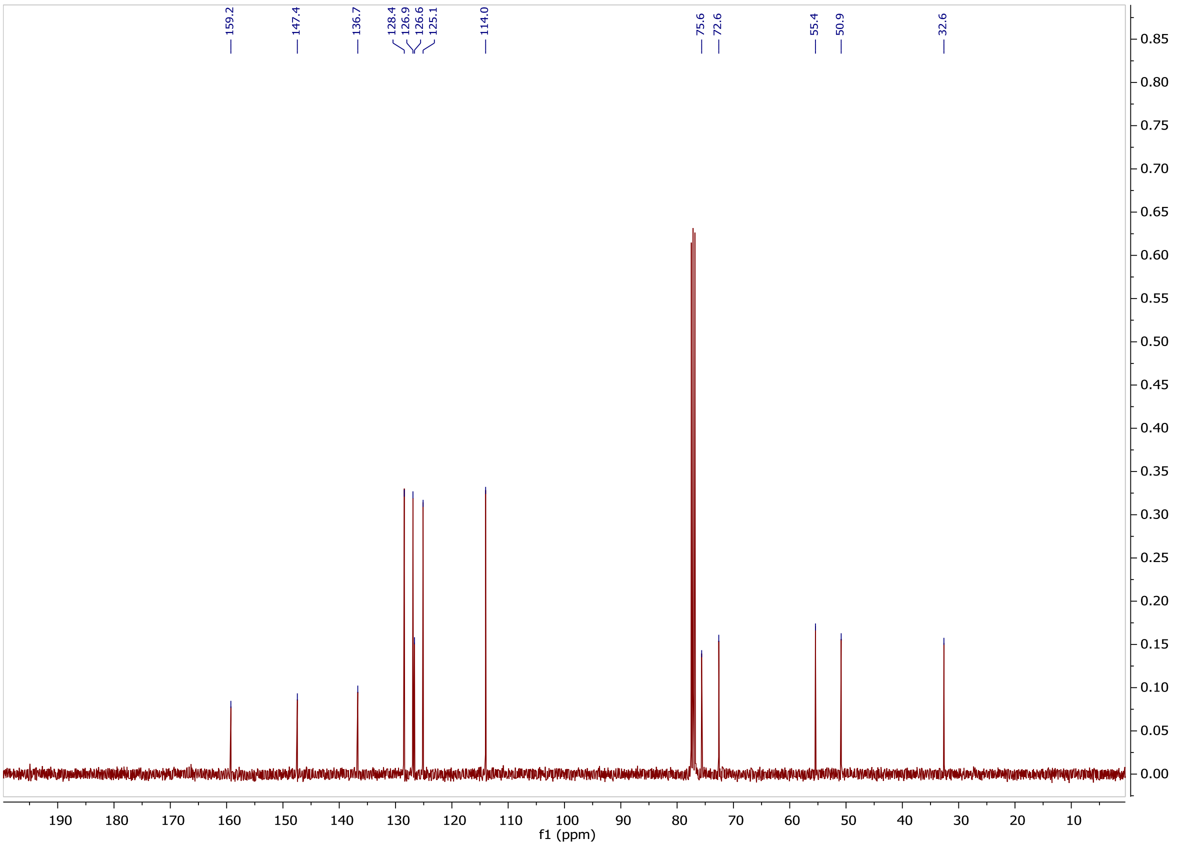

(4*S*,6*R*)-4,6-Dihydroxy-6-phenylheptyl diisopropylcarbamate (15):

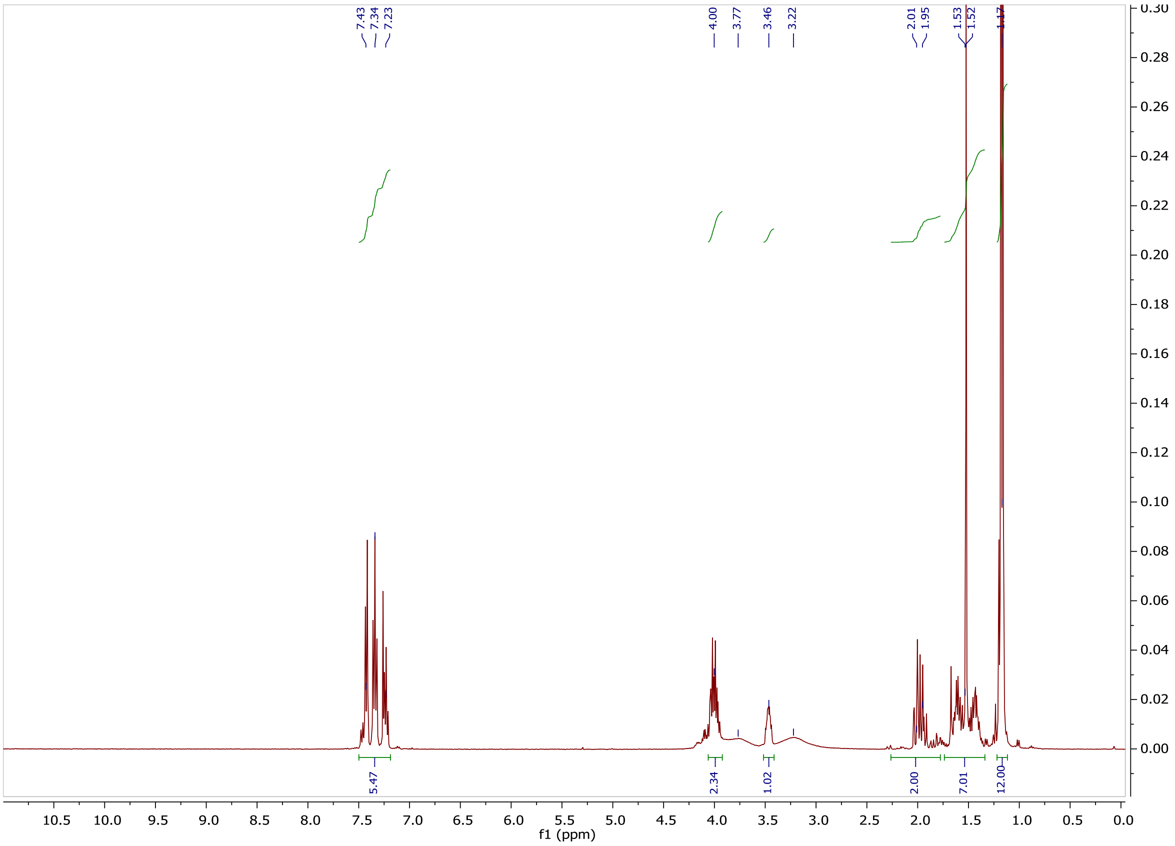

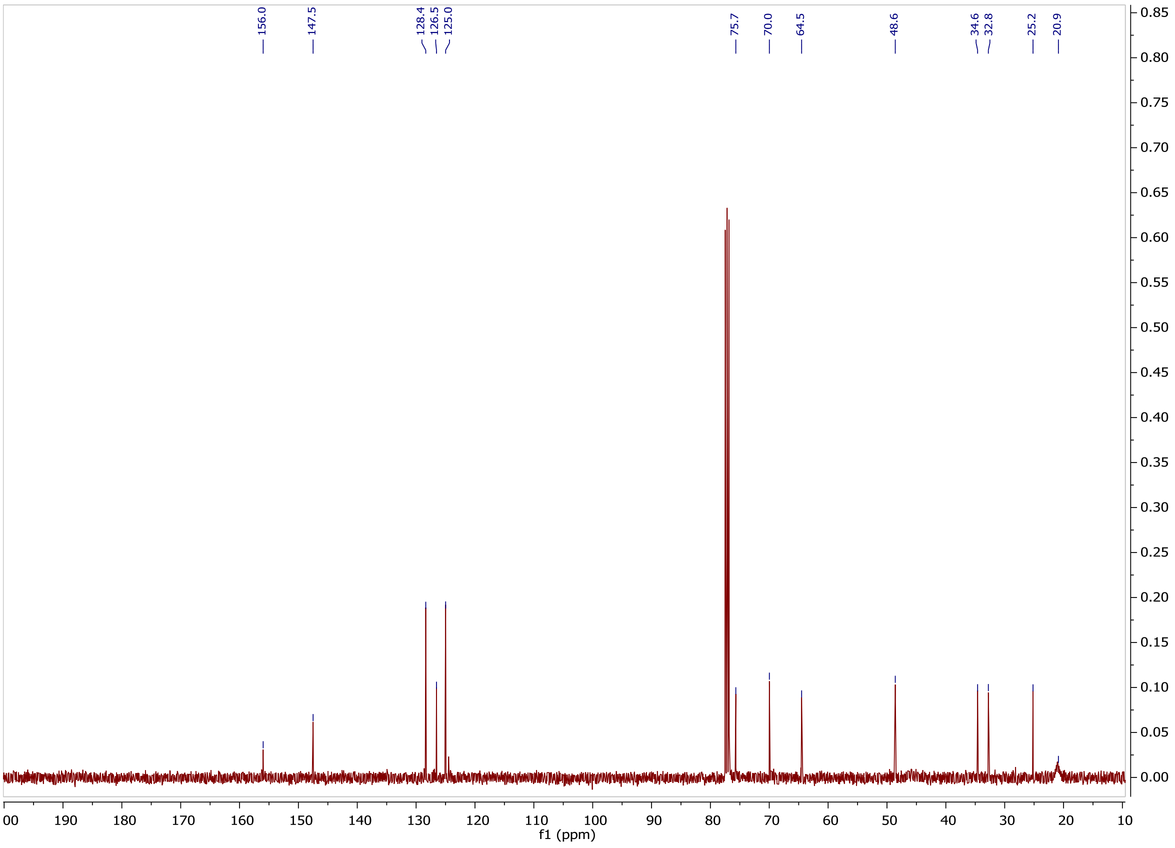

(2*R*,4*S*)-6,6-Dimethyl-2-phenylheptane-2,4-diol (16):

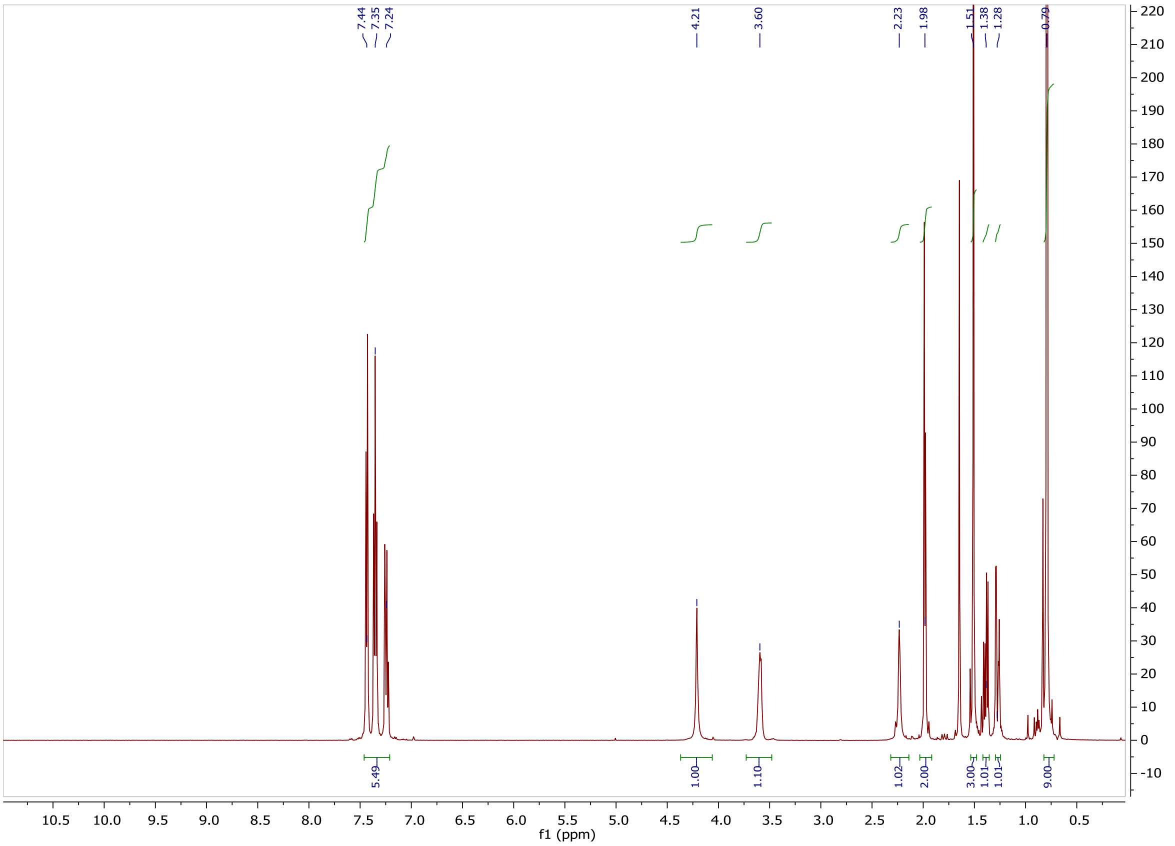

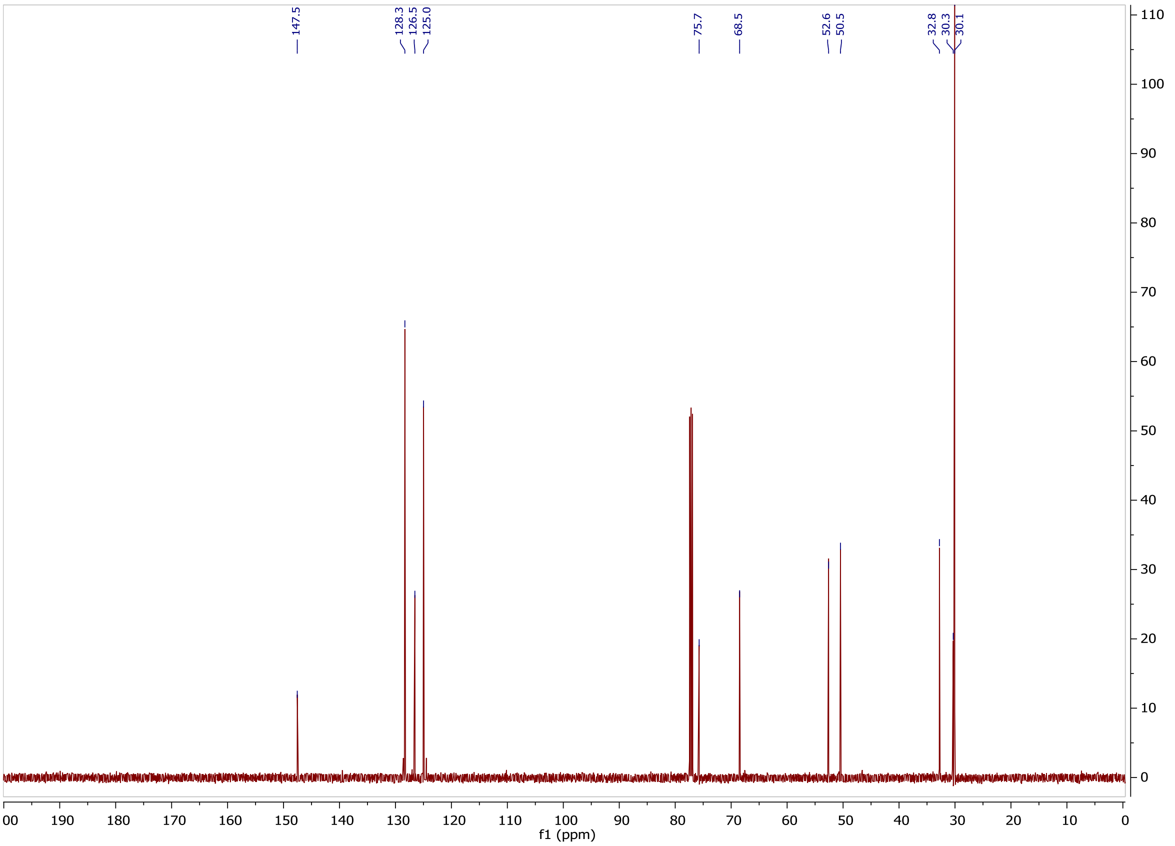

(2*R*,4*S*,6*S*)-6-((*tert*-Butyldiphenylsilyl)oxy)-2-phenylheptane-2,4-diol (17):

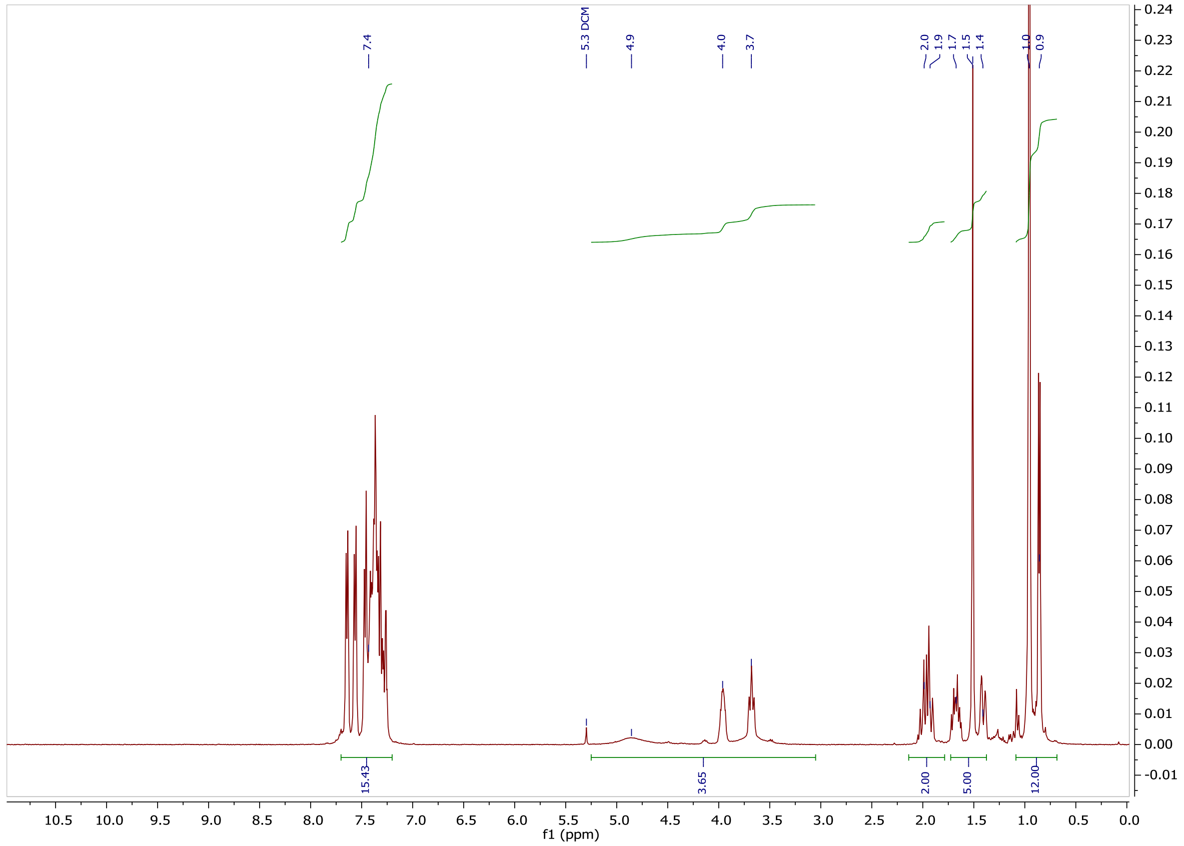

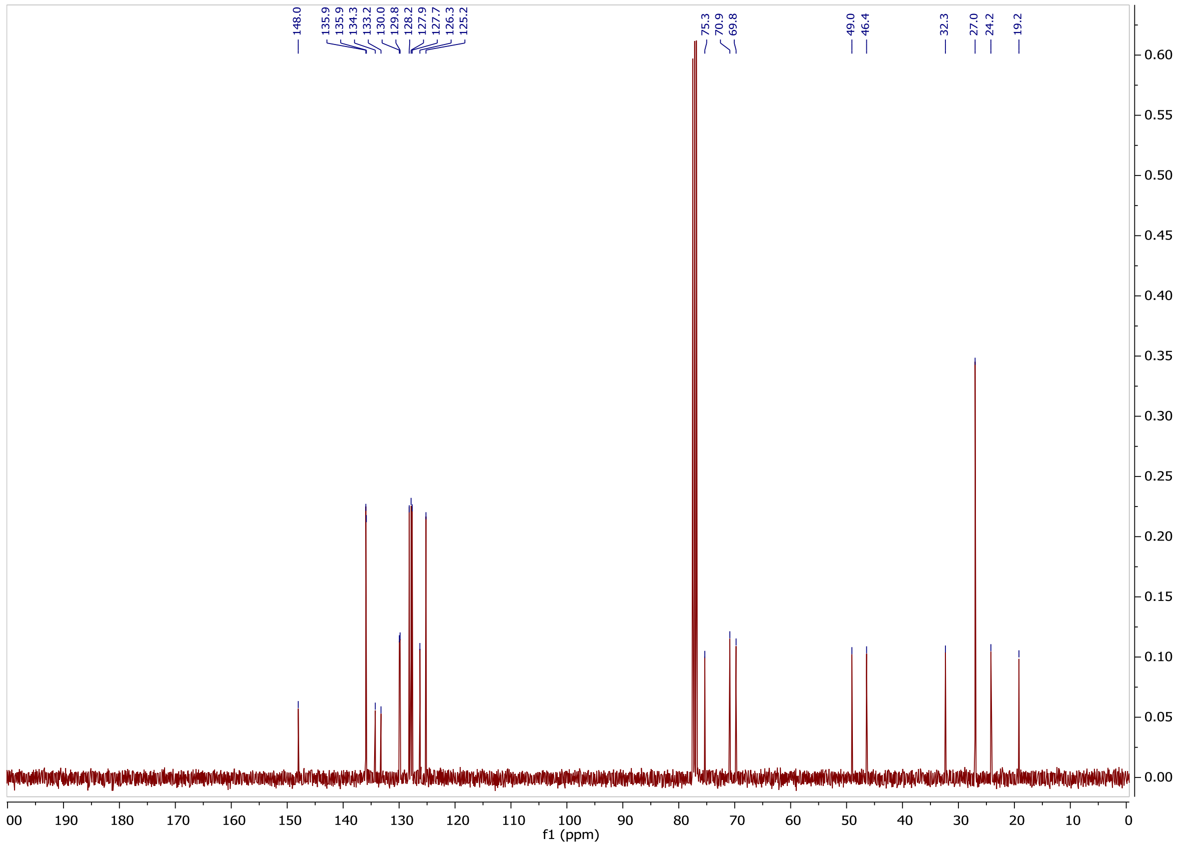

(2*R*,4*S*,6*S*)-6,10-Dimethyl-2-phenylundec-9-ene-2,4-diol (18):

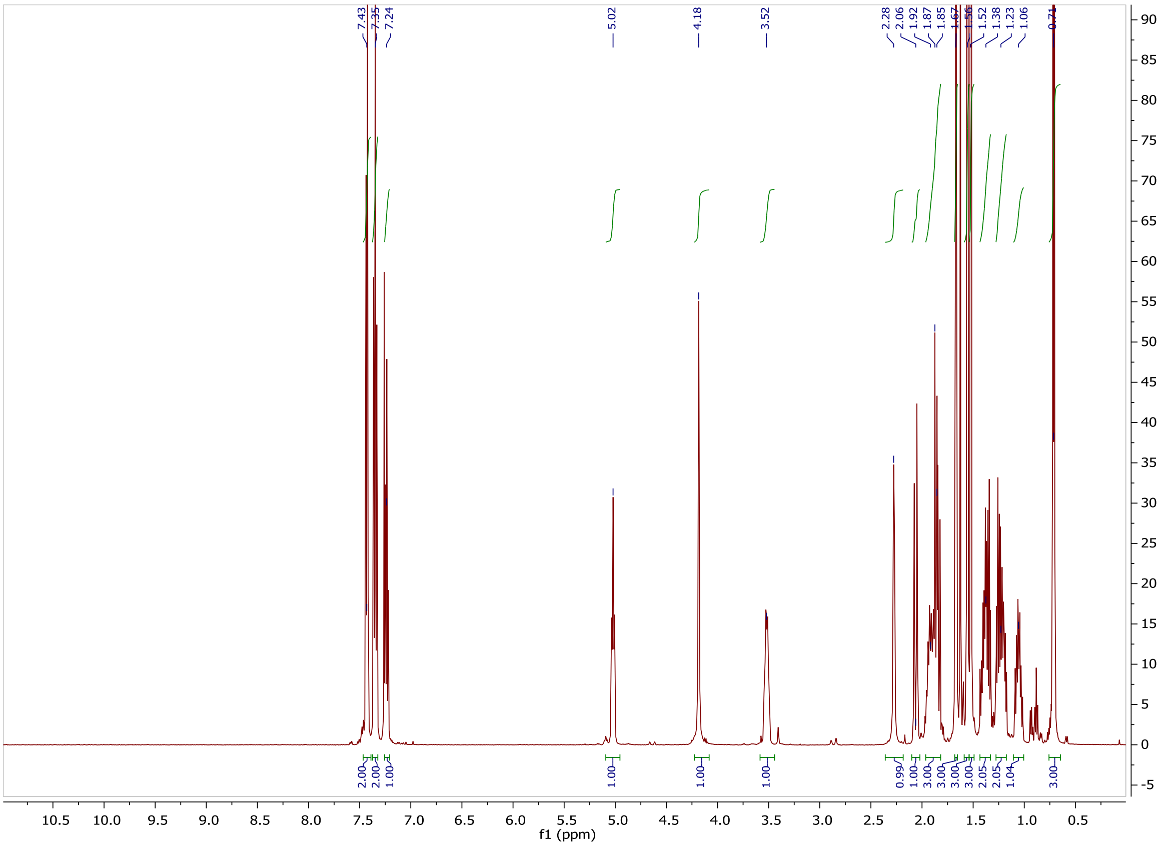

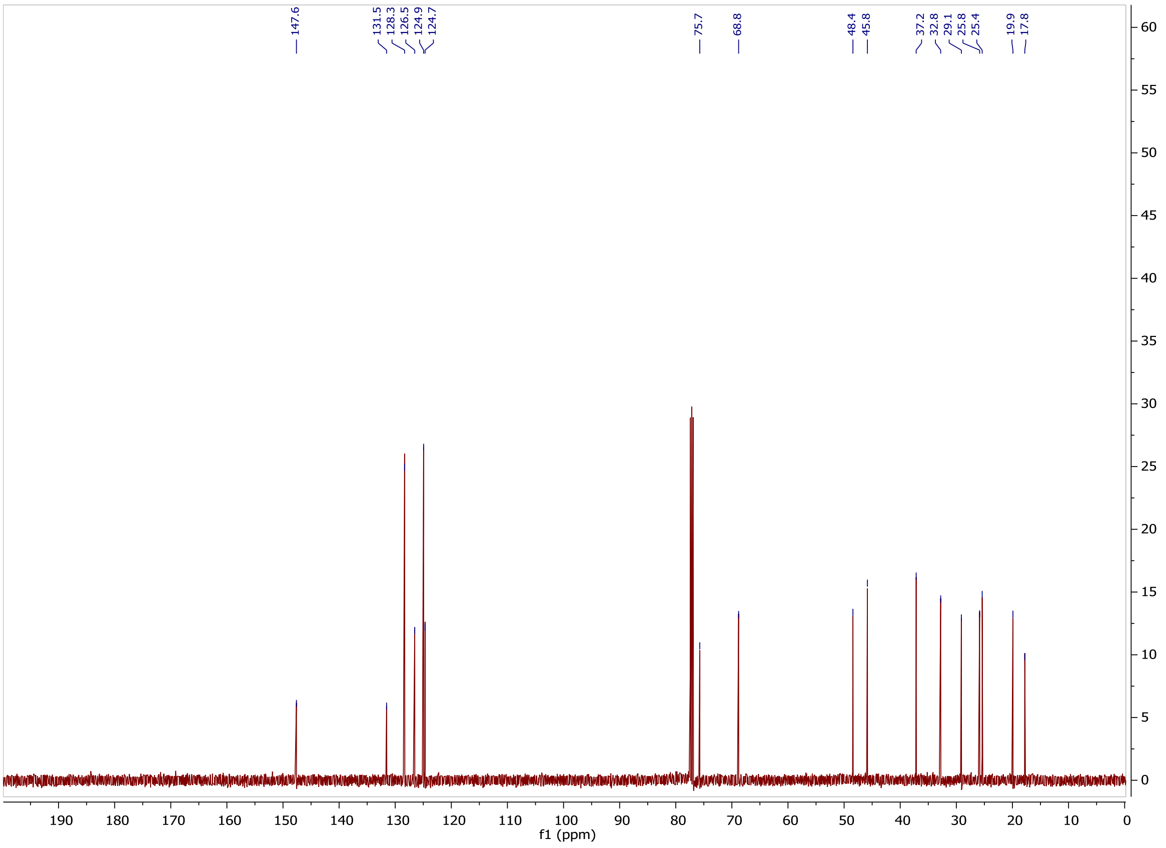

(2*R*,4*S*,5*S*,7*R*)-8-((*tert*-Butyldiphenylsilyl)oxy)-5,7-dimethyl-2-phenyloctane-2,4-diol (19):

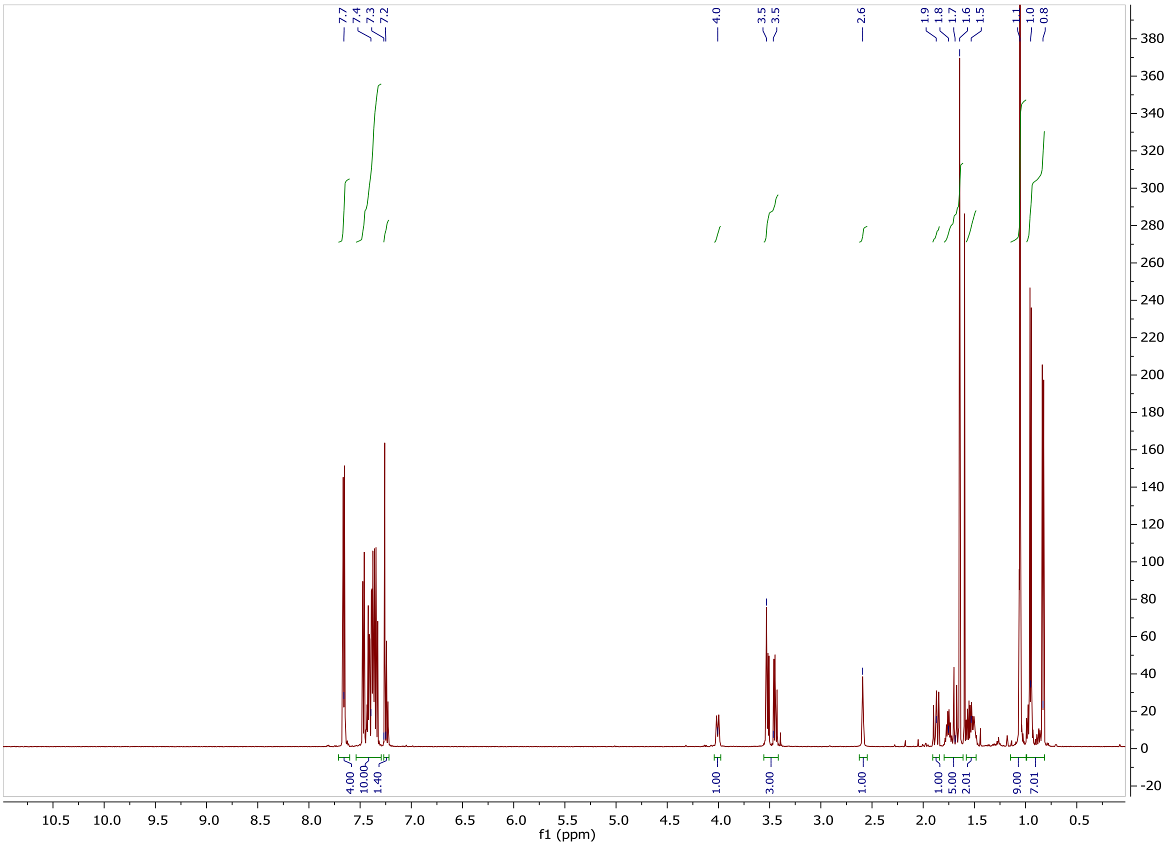

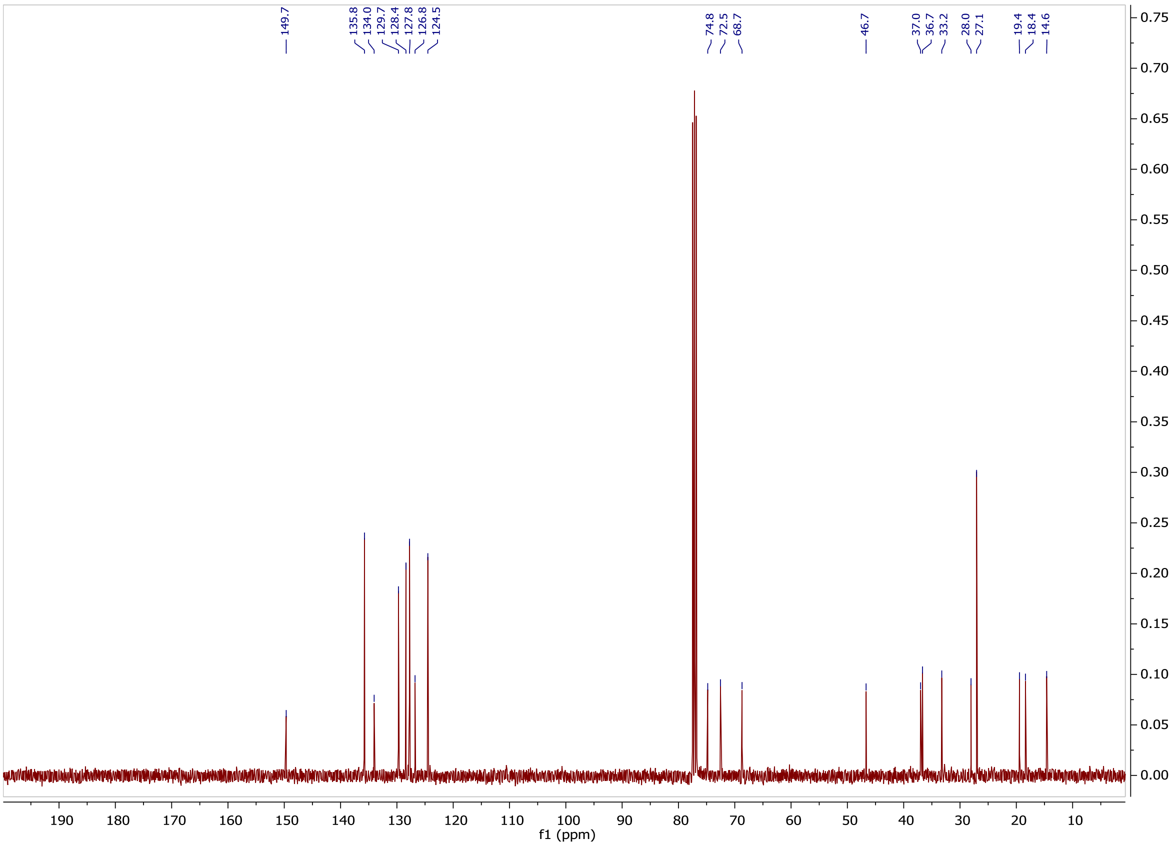

1-((3*R*,5*S*)-7-((*tert*-Butyldimethylsilyl)oxy)-3,5-dihydroxyheptyl)-5-(4-fluorophenyl)-2-isopropyl-N,4-diphenyl-1H-pyrrole-3-carboxamide (20):

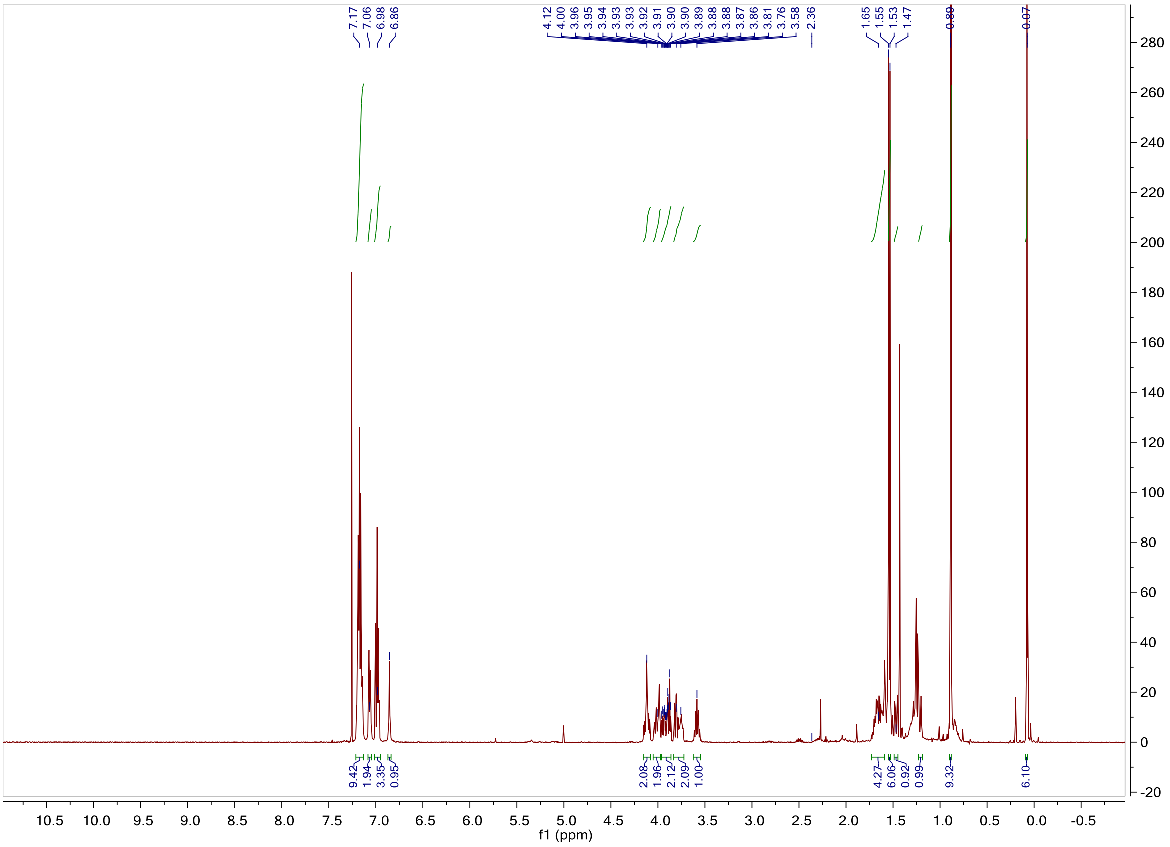

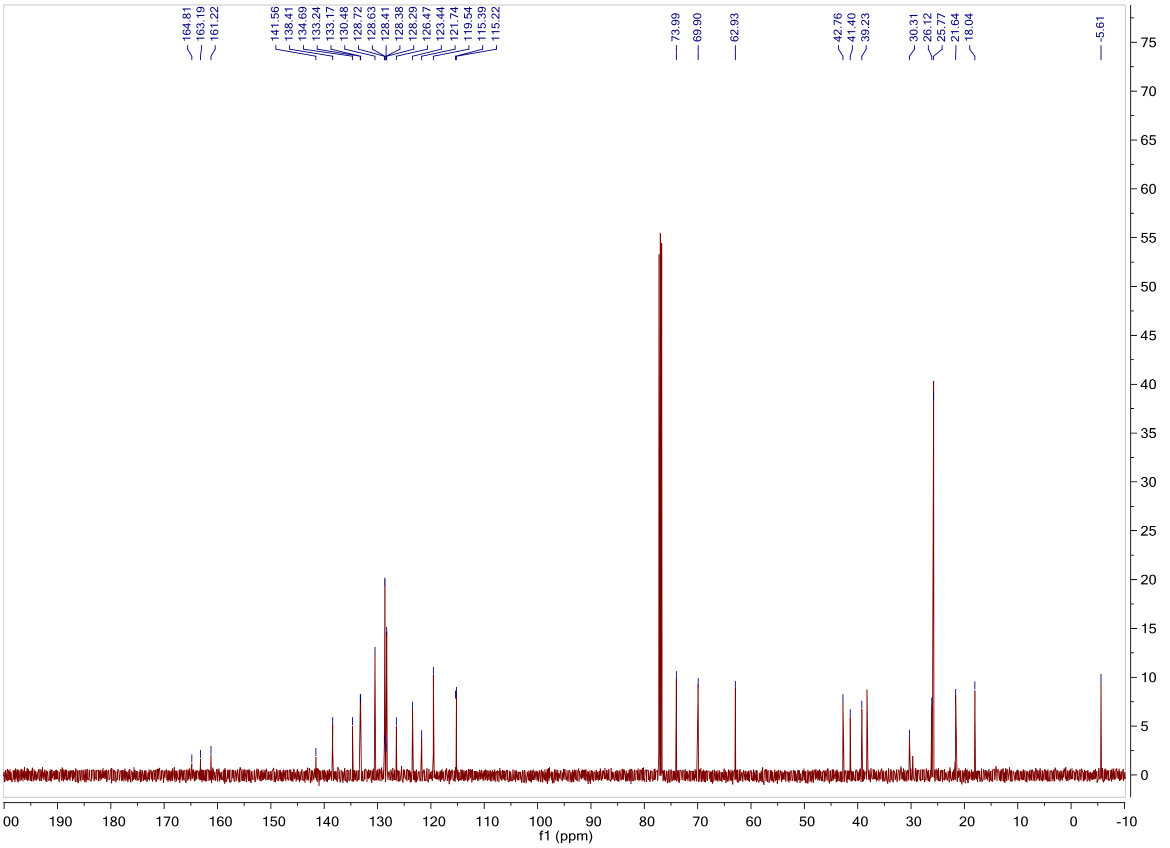

tert-Butyldimethyl(penta-1,4-dien-3-yloxy)silane (25a)

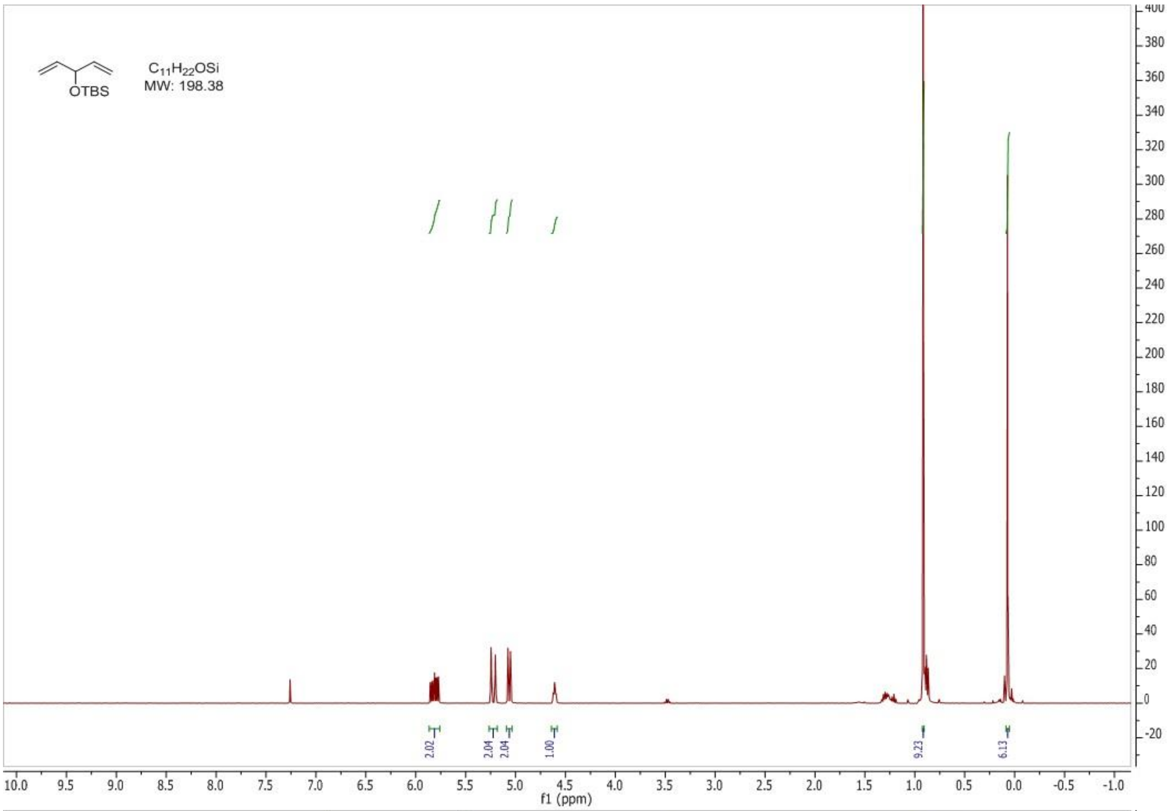

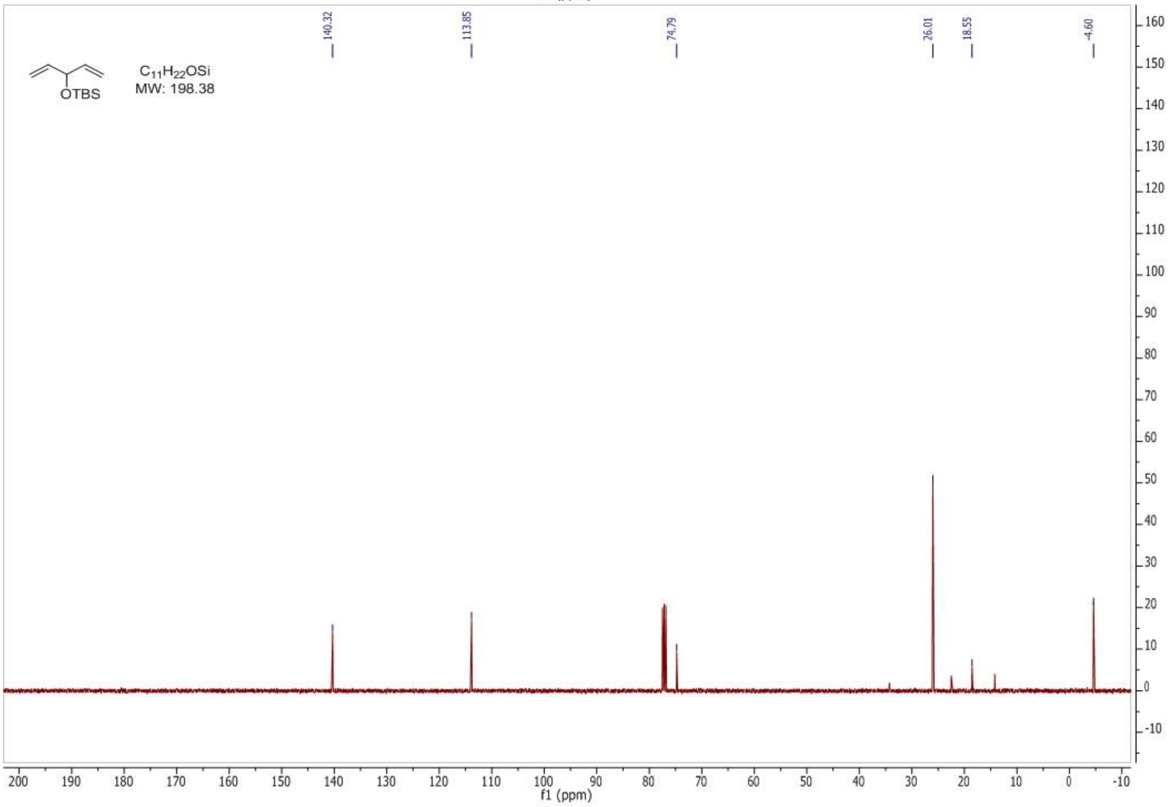

***tert*-Butyl(penta-1,4-dien-3-yloxy)diphenylsilane (25e)**

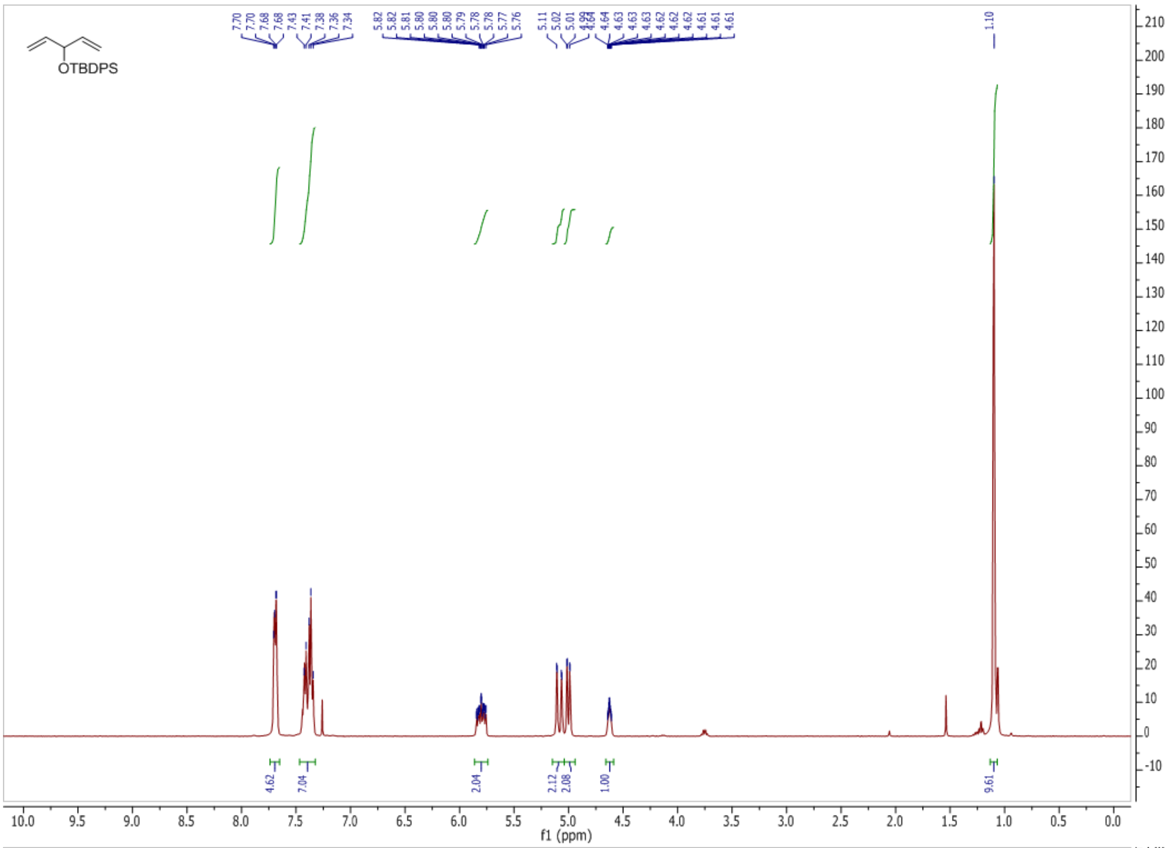

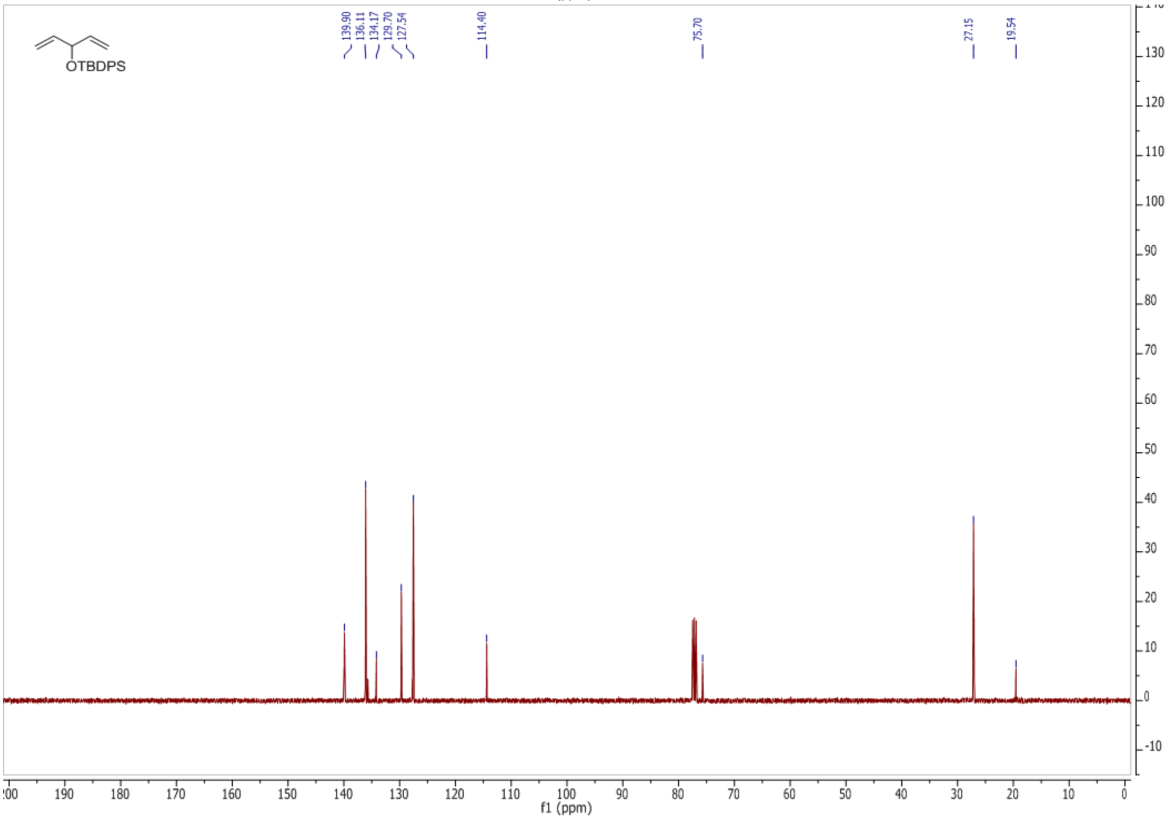

[(Penta-1,4-dien-3-yloxy)methyl]benzene (25g)

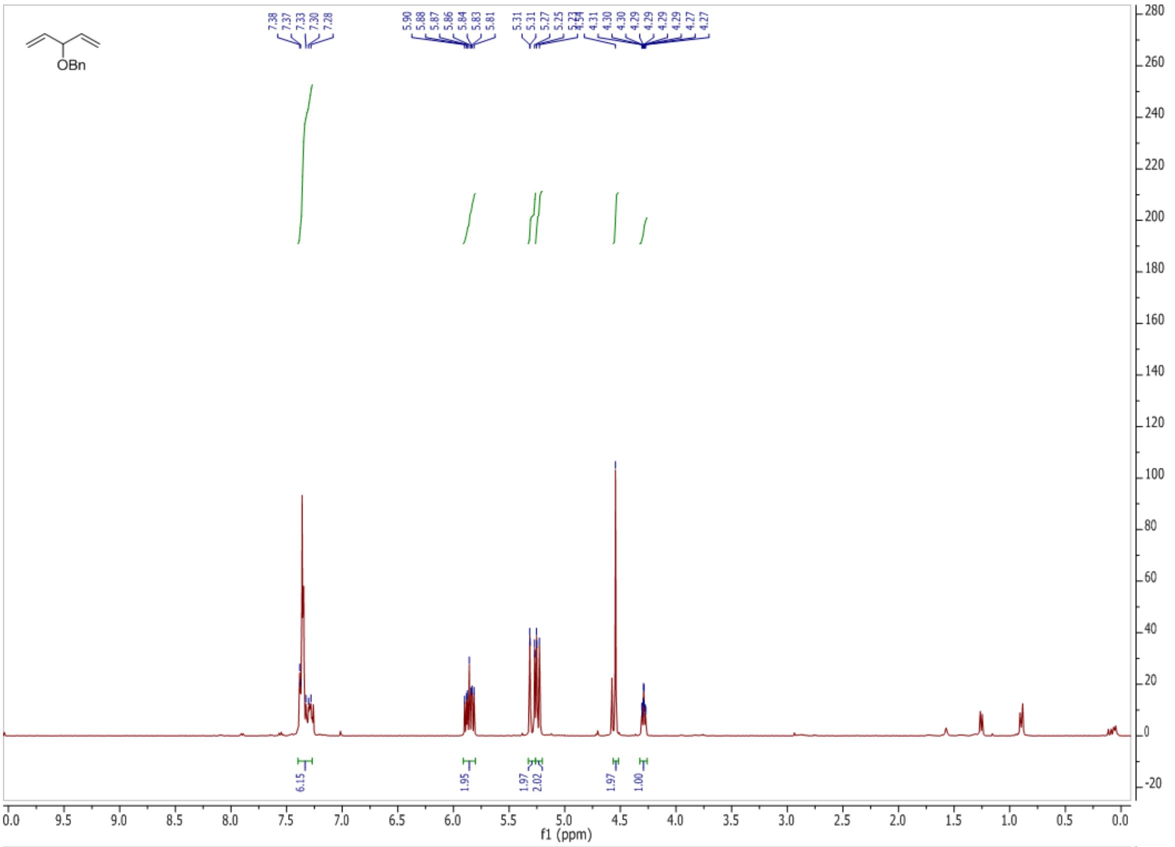

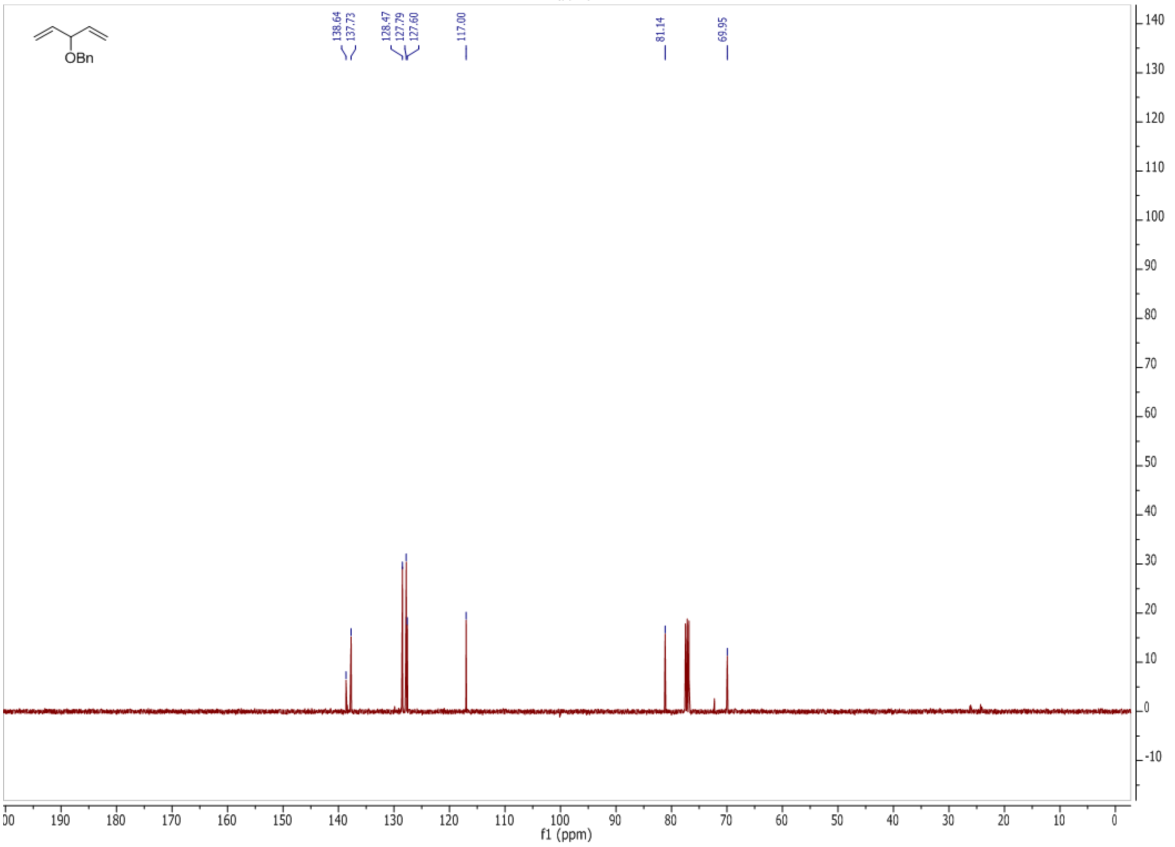

3-(Methoxymethoxy)penta-1,4-diene (25h)

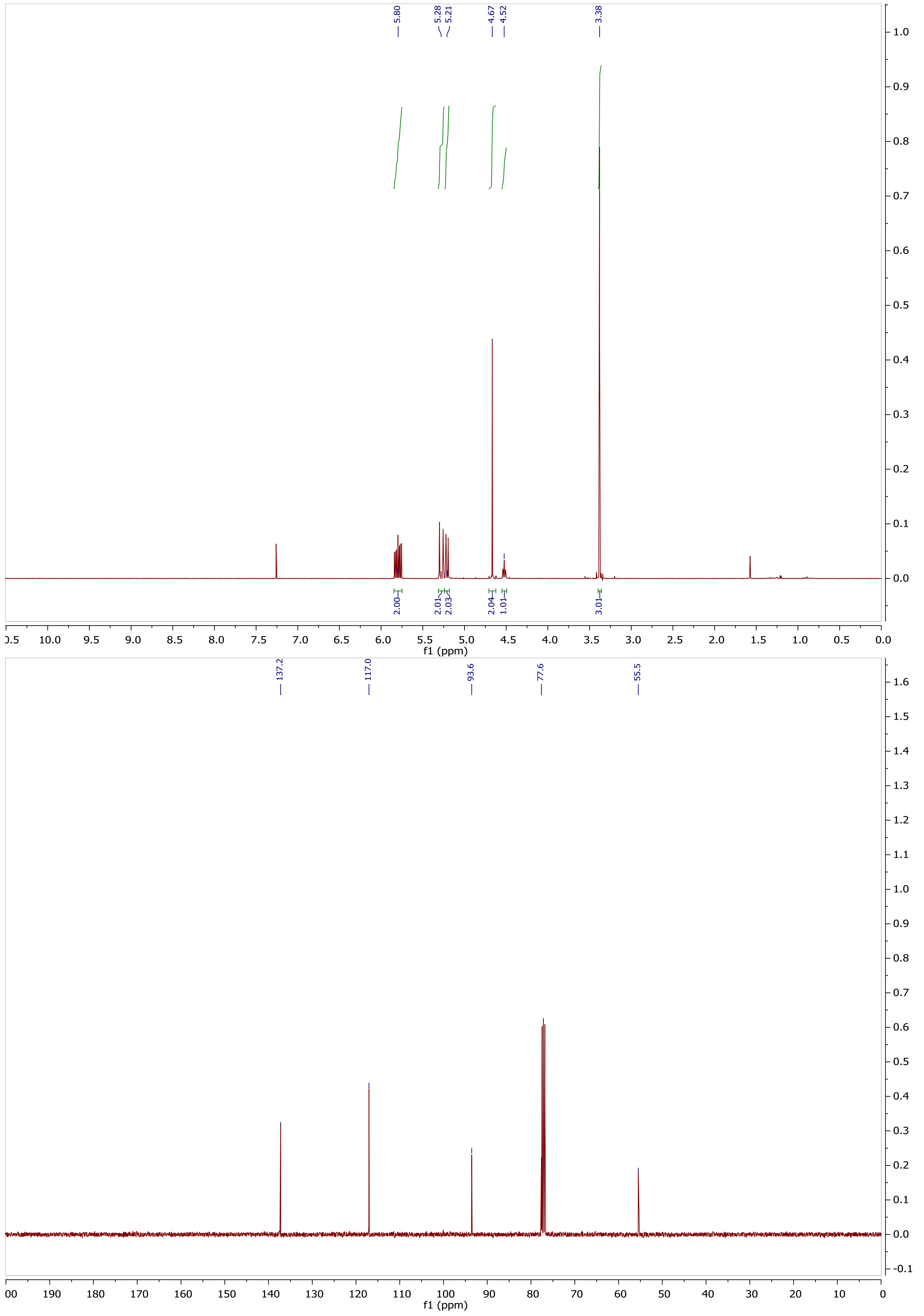

{[(3*S*,4*S*)-4,5-Bis(4,4,5,5-tetramethyl-1,3,2-dioxaborolan-2-yl)pent-1-en-3-yl]oxy}triethylsilane (64)

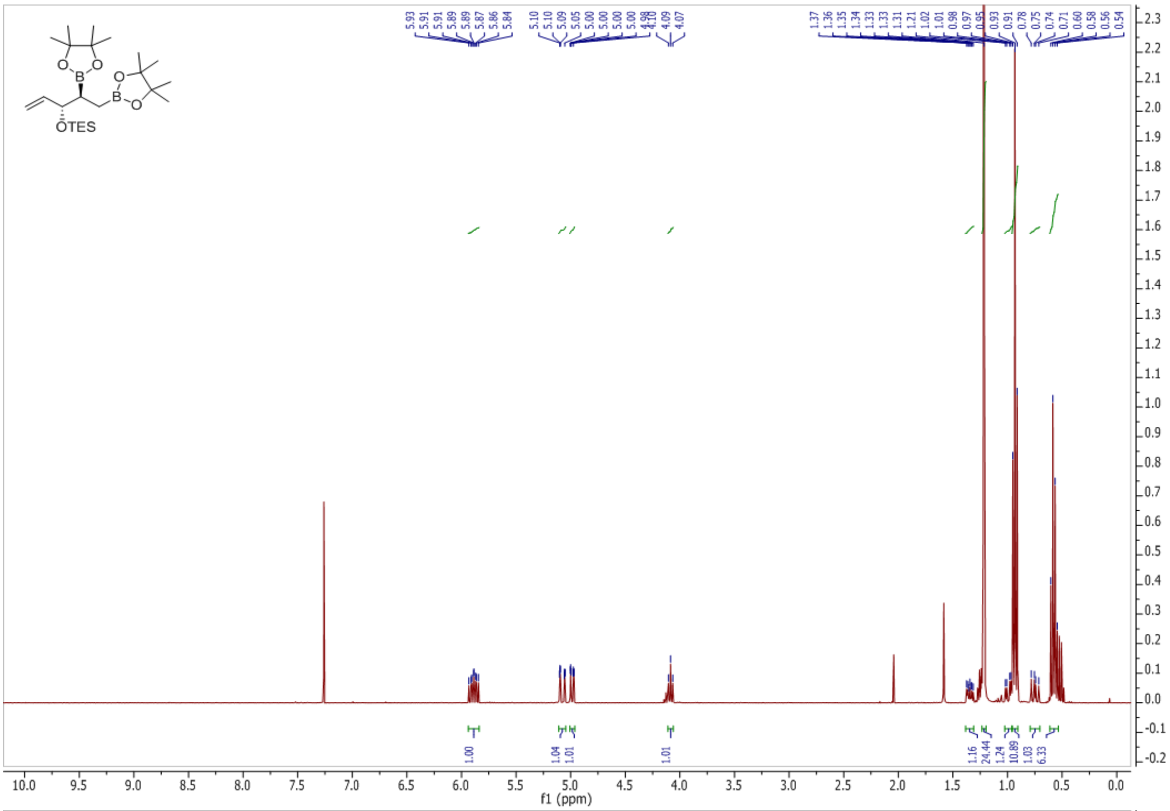

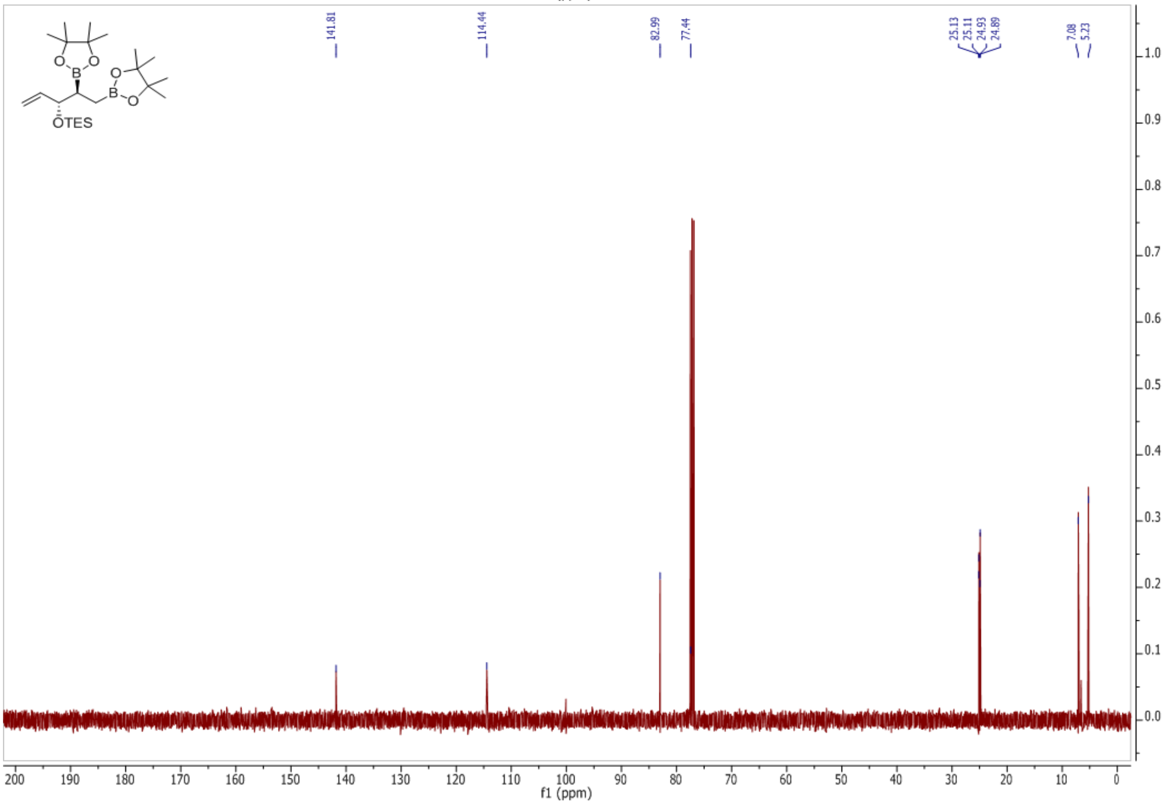

{[(3*S*,4*S*)-4,5-Bis(4,4,5,5-tetramethyl-1,3,2-dioxaborolan-2-yl)pent-1-en-3-yl]oxy}triisopropylsilane (65)

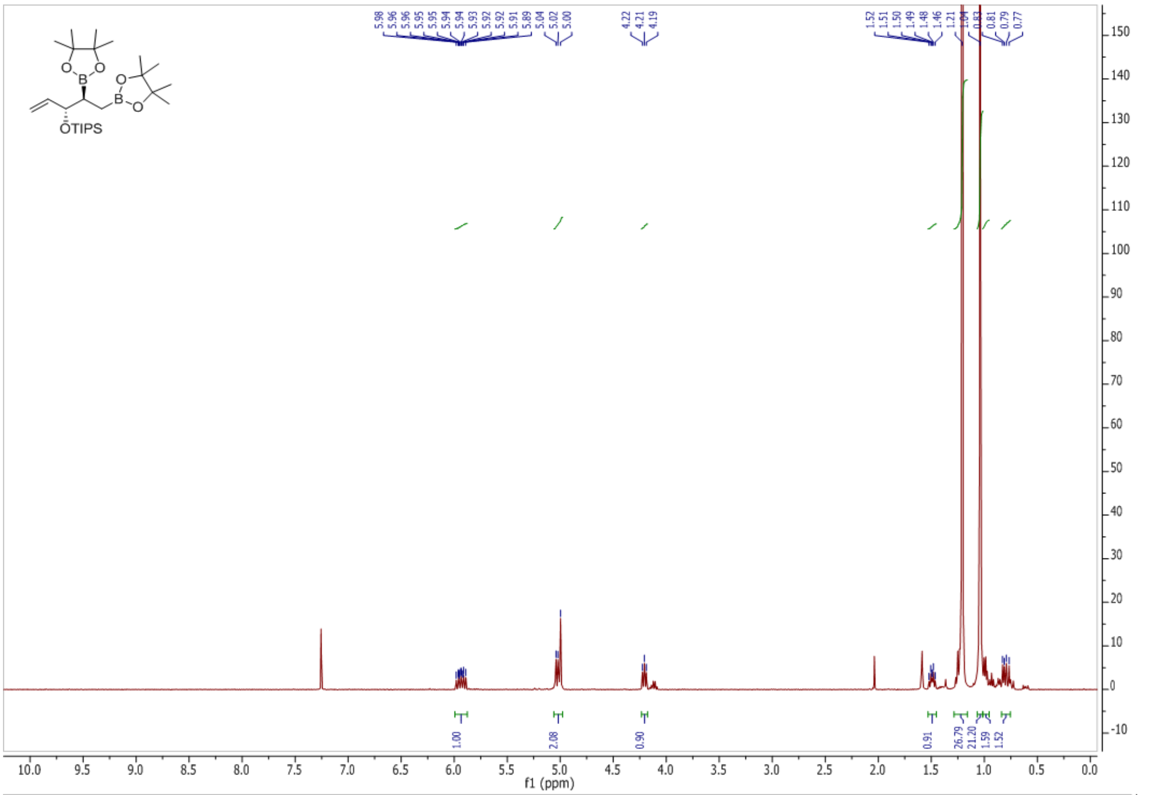

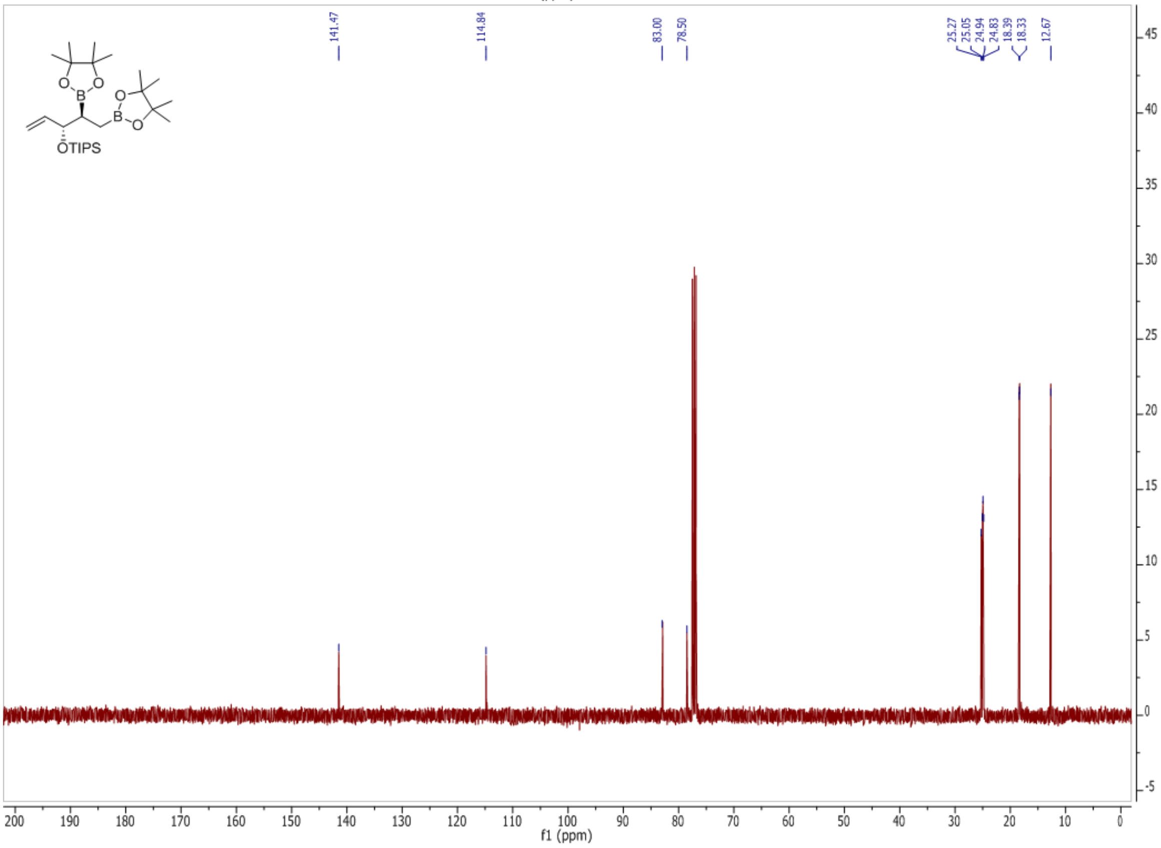

{[(3*S*,4*S*)-4,5-Bis(4,4,5,5-tetramethyl-1,3,2-dioxaborolan-2-yl)pent-1-en-3-yl]oxy}(*tert*-butyl)dimethylsilane (27)

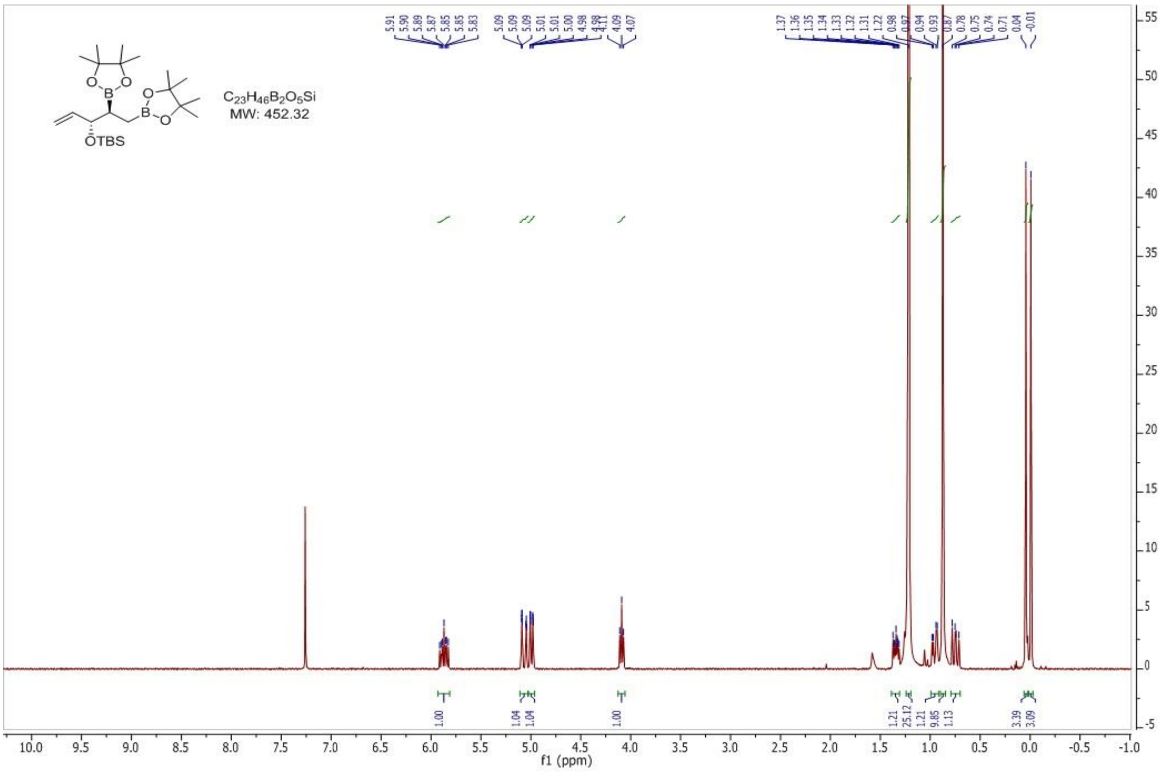

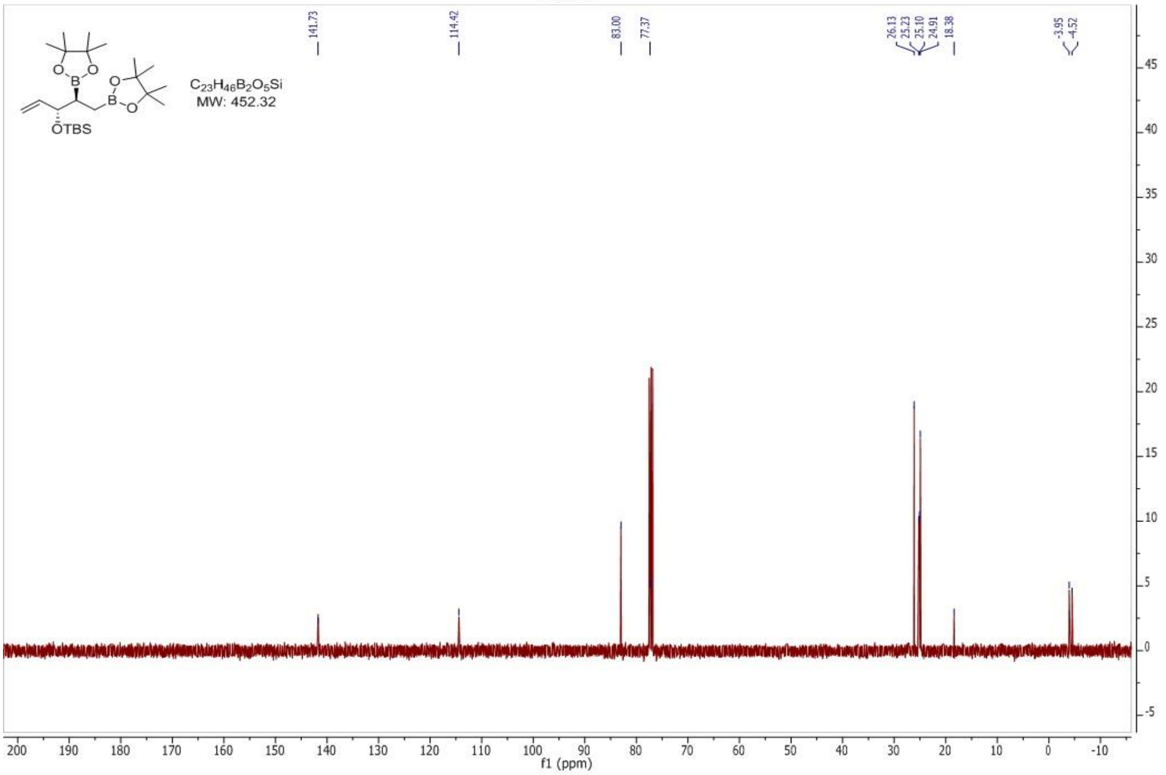

{[(3*S*,4*S*)-4,5-Bis(4,4,5,5-tetramethyl-1,3,2-dioxaborolan-2-yl)pent-1-en-3-yl]oxy}(tert-butyl)diphenylsilane (66)

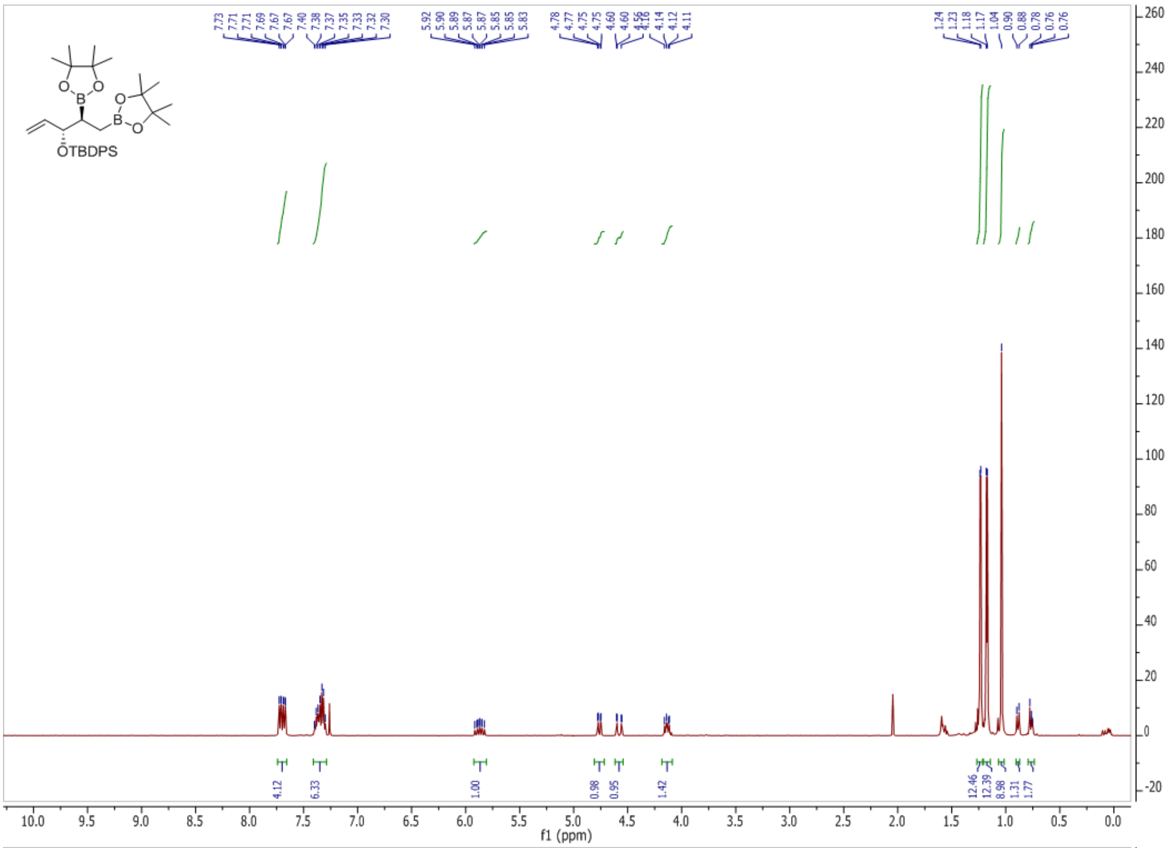

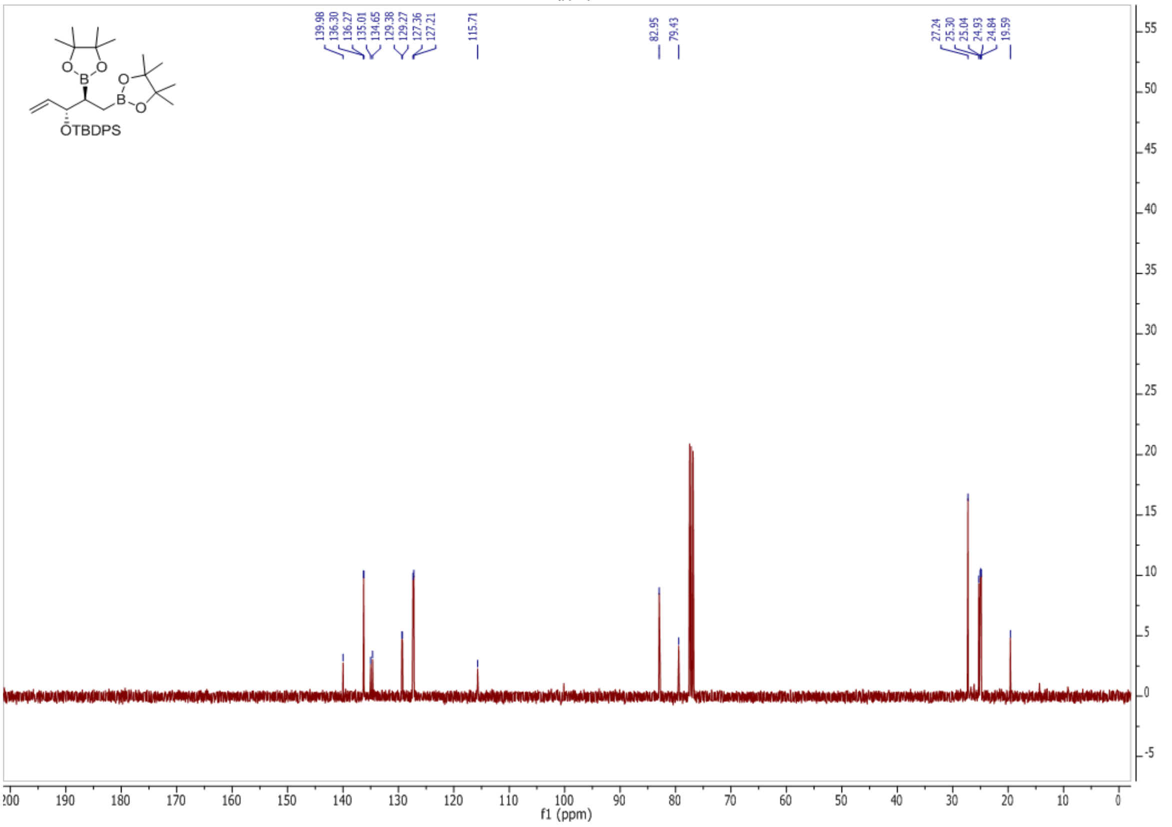

2,2'-[(2*S*,3*S*)-3-(Benzyloxy)pent-4-ene-1,2-diyl]bis(4,4,5,5-tetramethyl-1,3,2-dioxaborolane) (67)

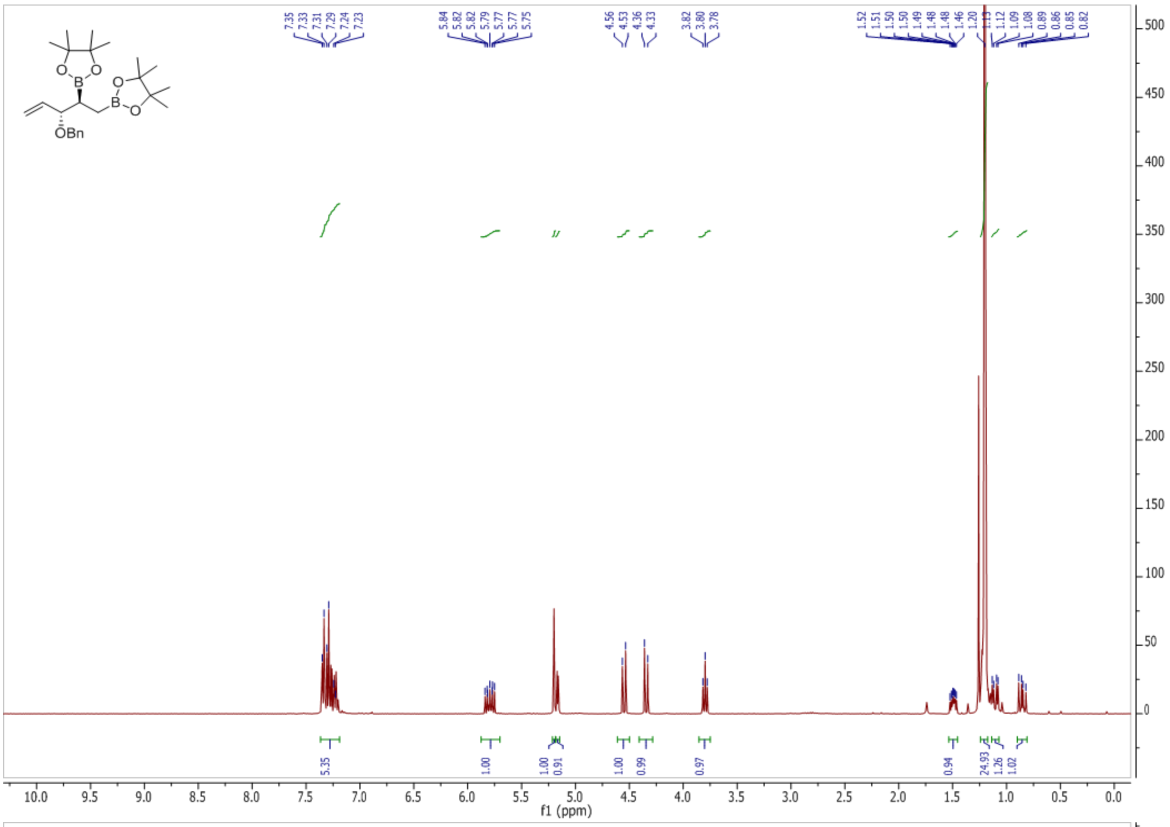

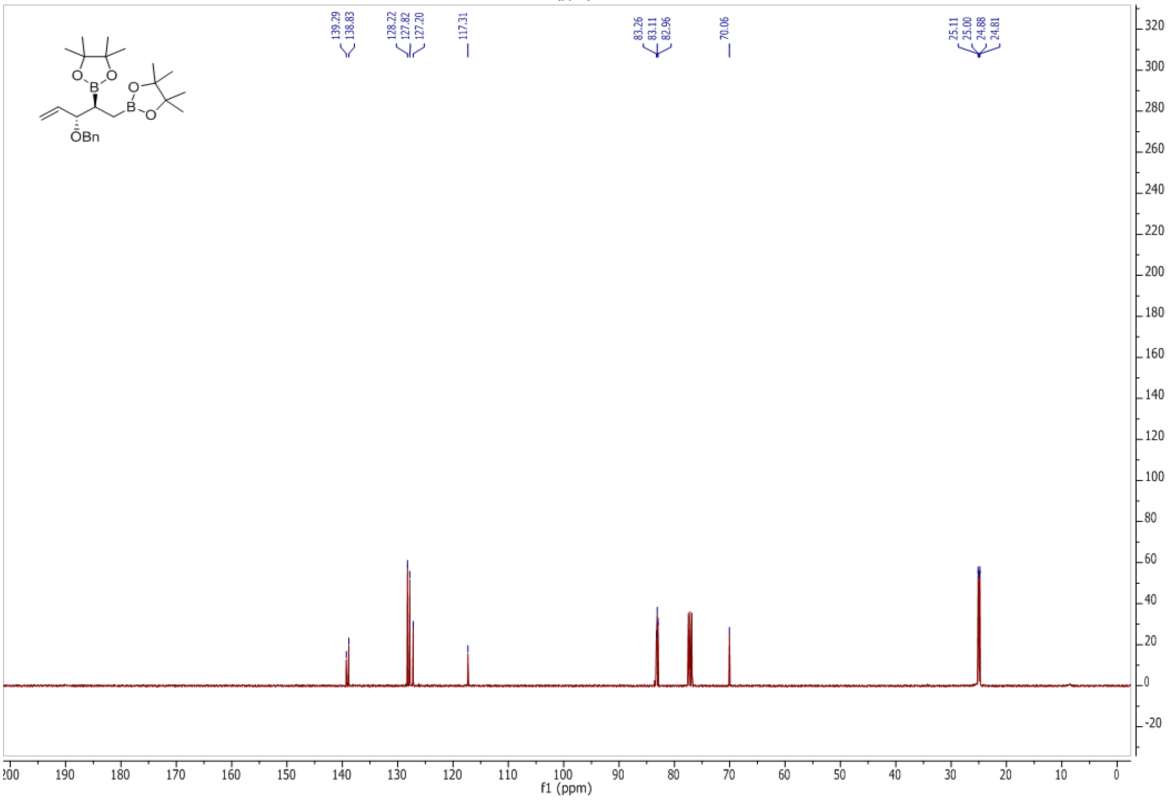

2,2'-[3-(Methoxymethoxy)pent-4-ene-1,2-diyl]bis(4,4,5,5-tetramethyl-1,3,2-dioxaborolane) (68)

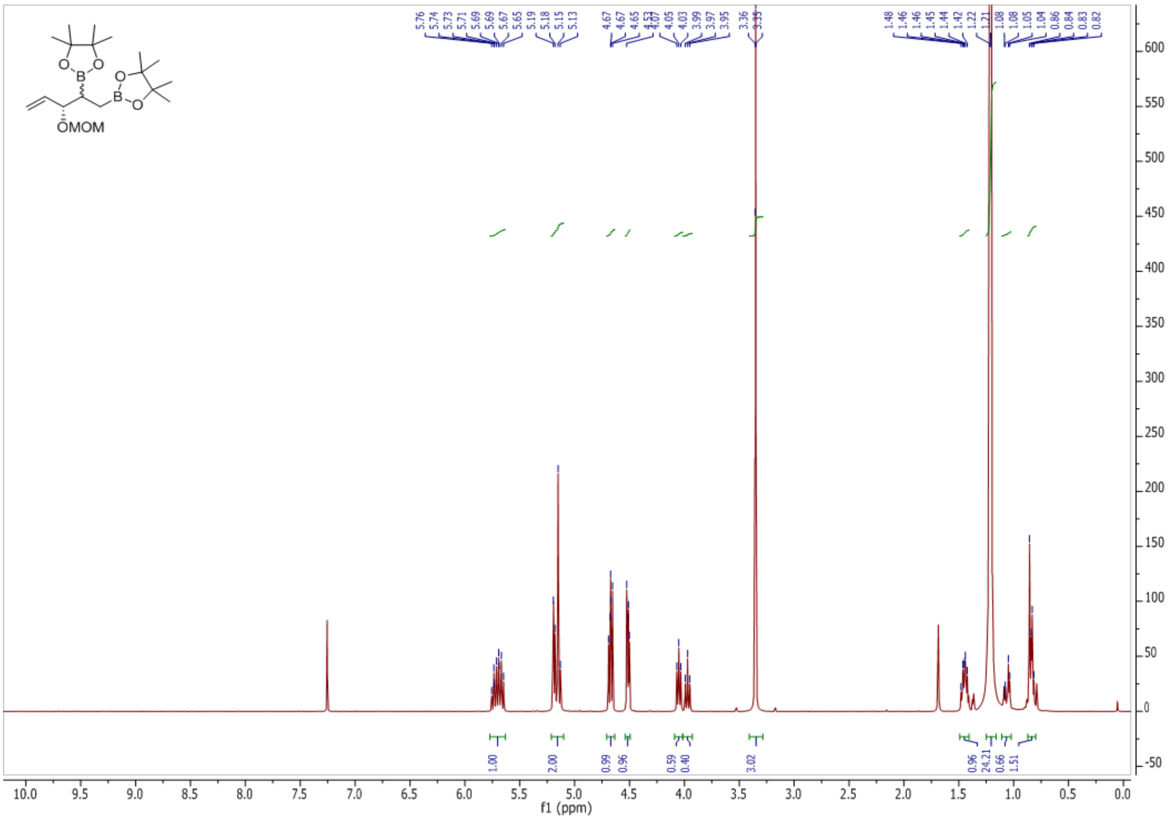

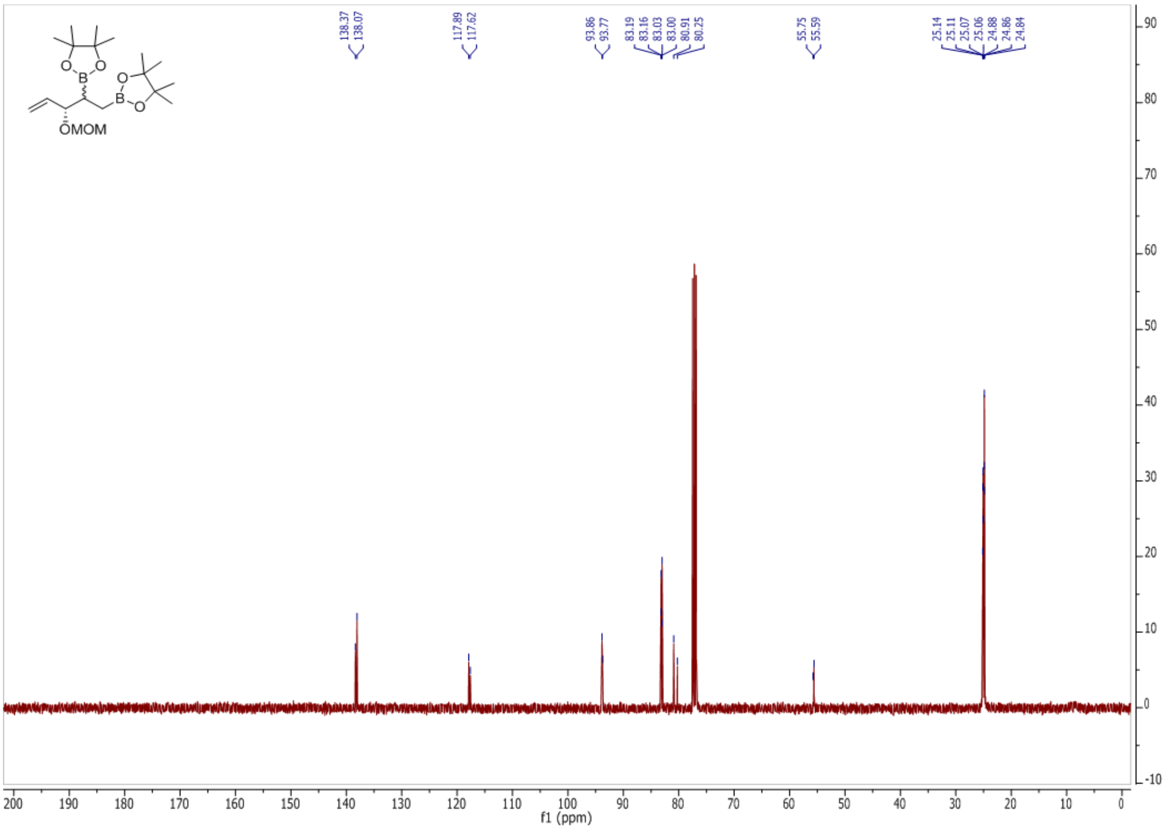

Heptane-1,7-diyl bis(2,4,6-triisopropylbenzoate) (24b)

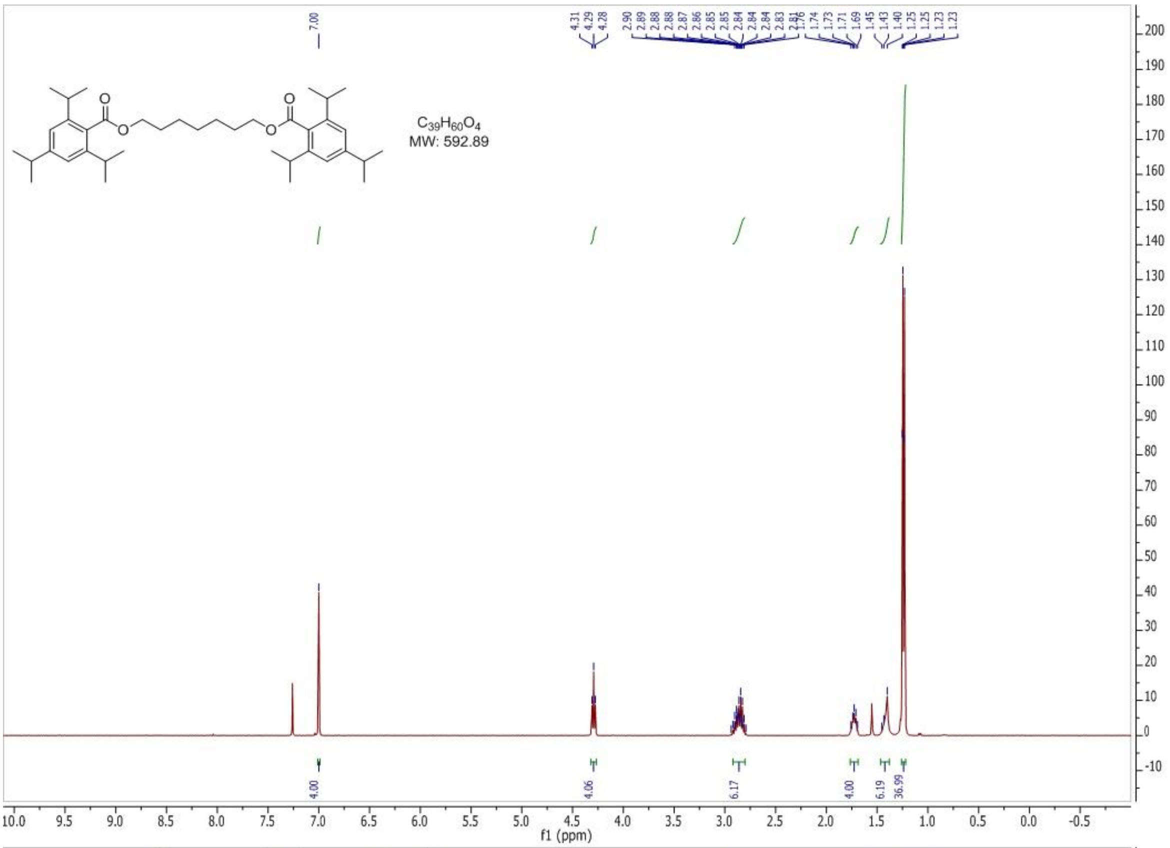

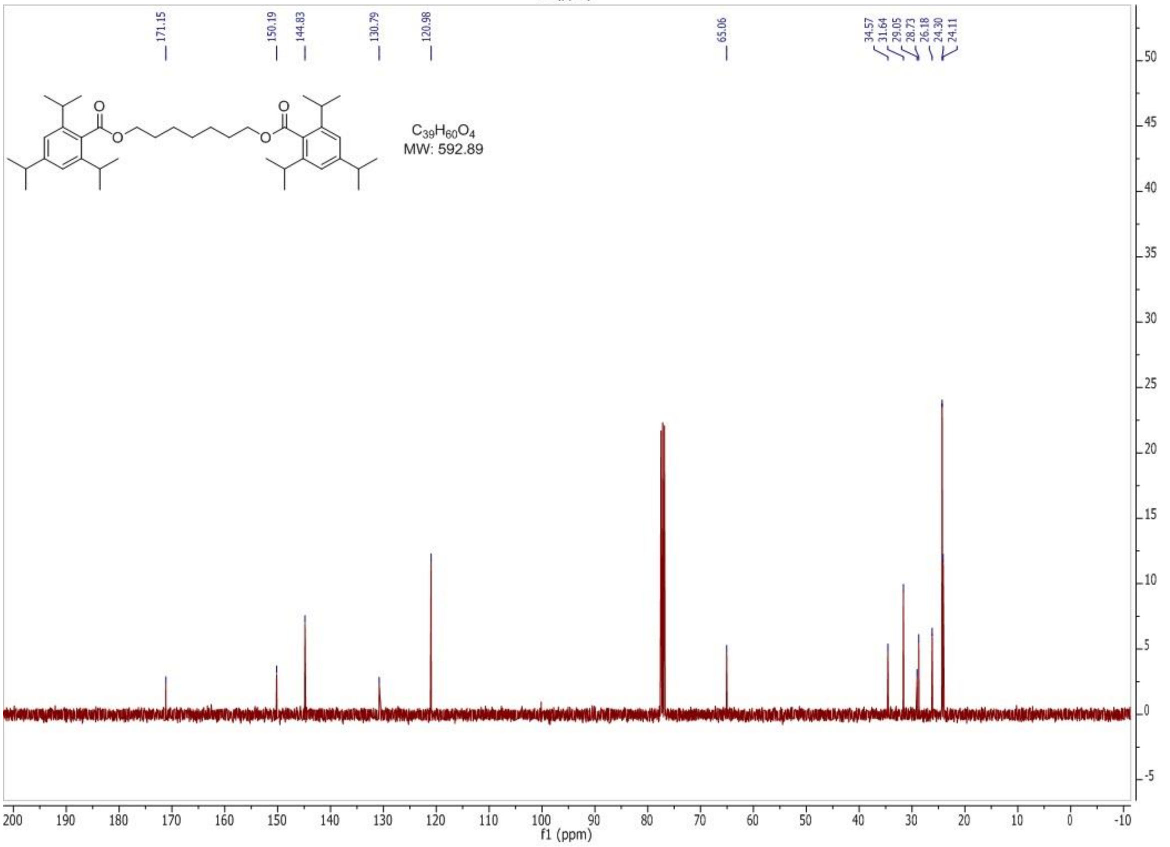

4,4,5,5-Tetramethyl-2-pentyl-1,3,2-dioxaborolane (23)

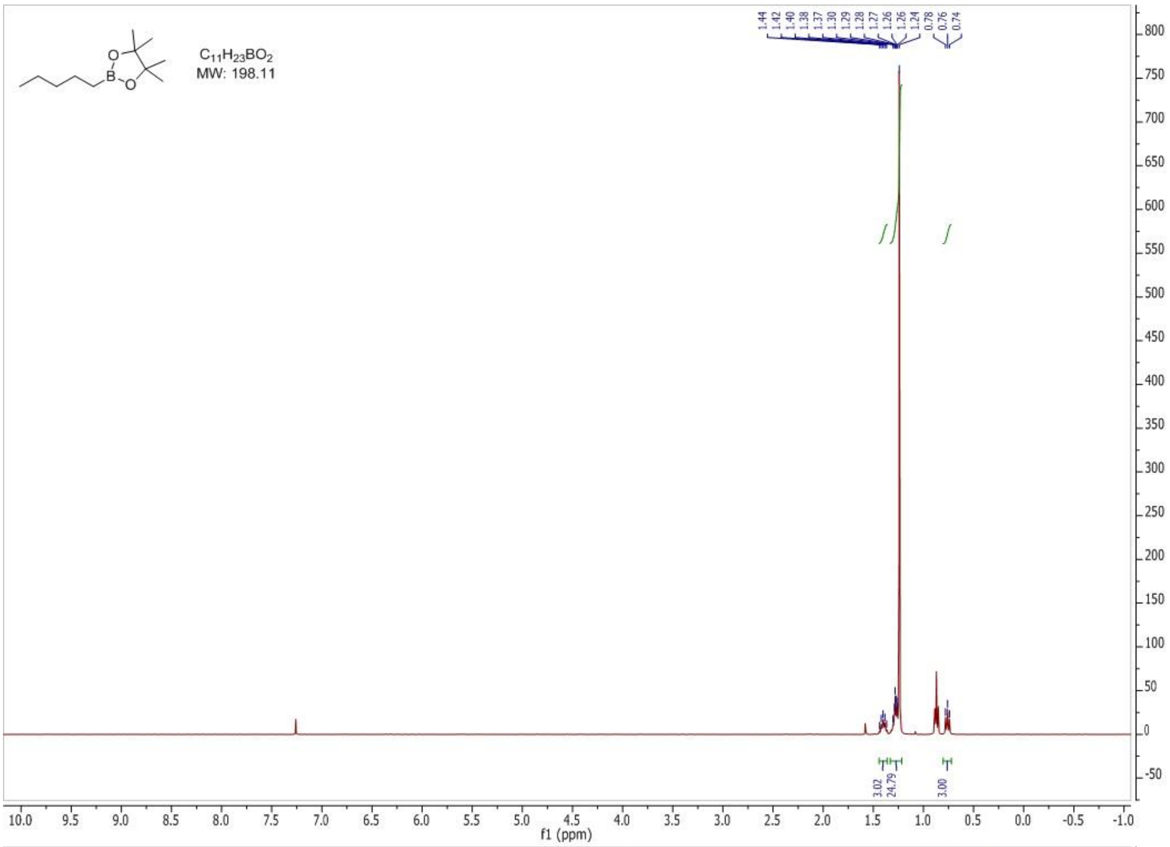

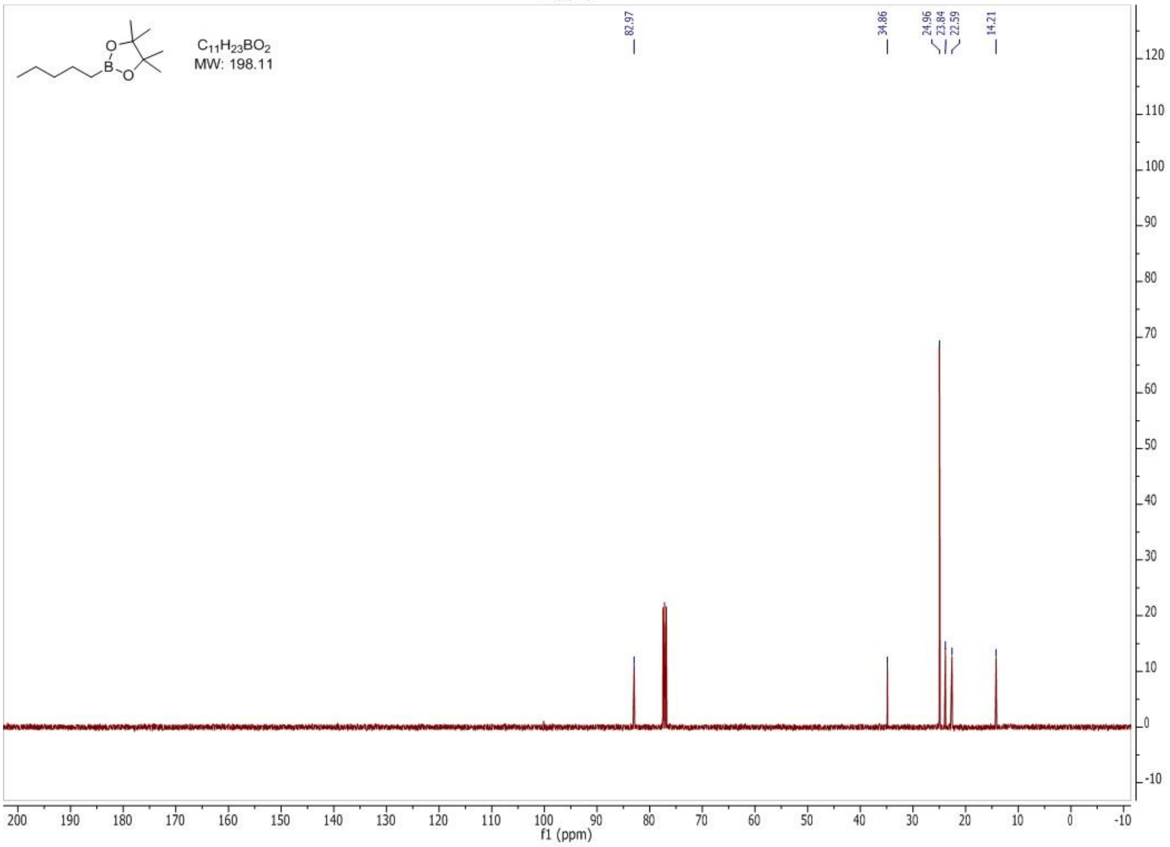

(7*R*,9*S*,10*R*)-7,9,10-Tris[(*tert*-butyldimethylsilyl)oxy]dodec-11-en-1-yl 2,4,6-triisopropylbenzoate (28)

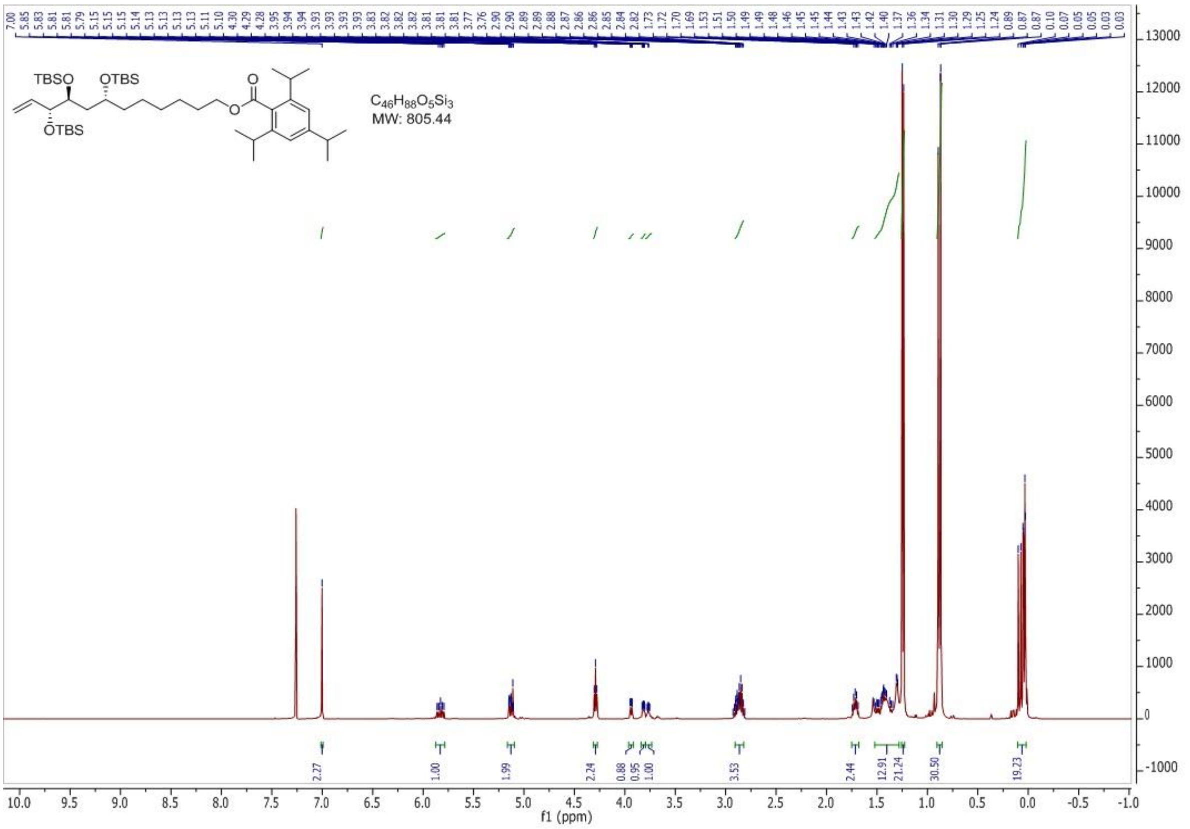

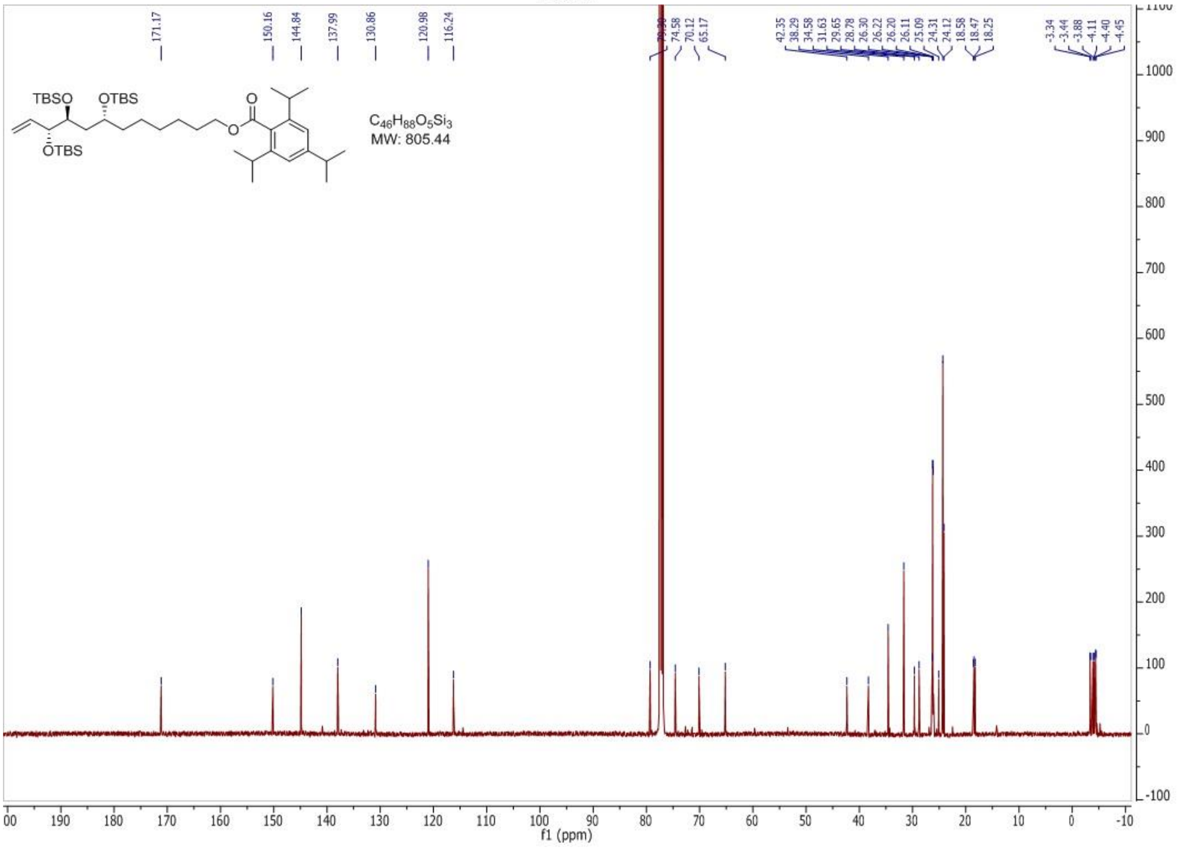

(6*R*,12*R*,14*S*,15*R*)-12,14,15-Tris[(*tert*-butyldimethylsilyl)oxy]heptadec-16-en-6-ol (29)

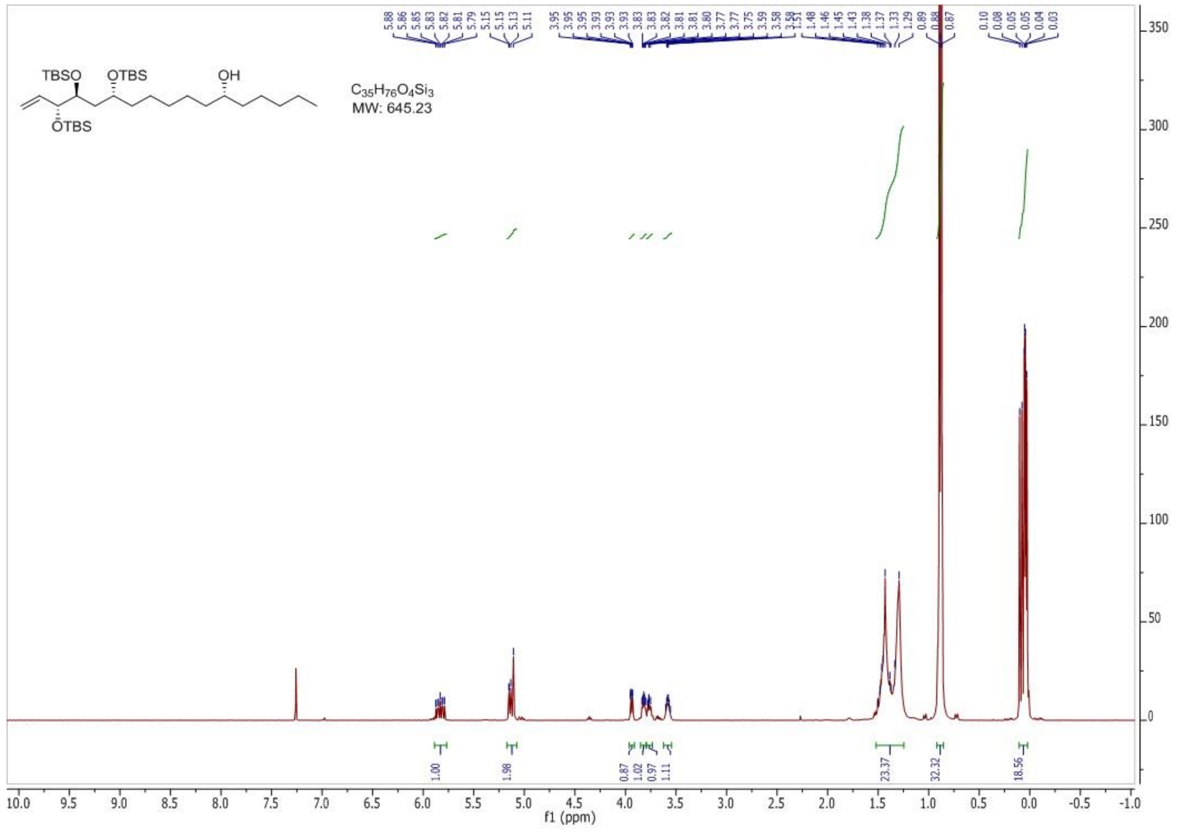

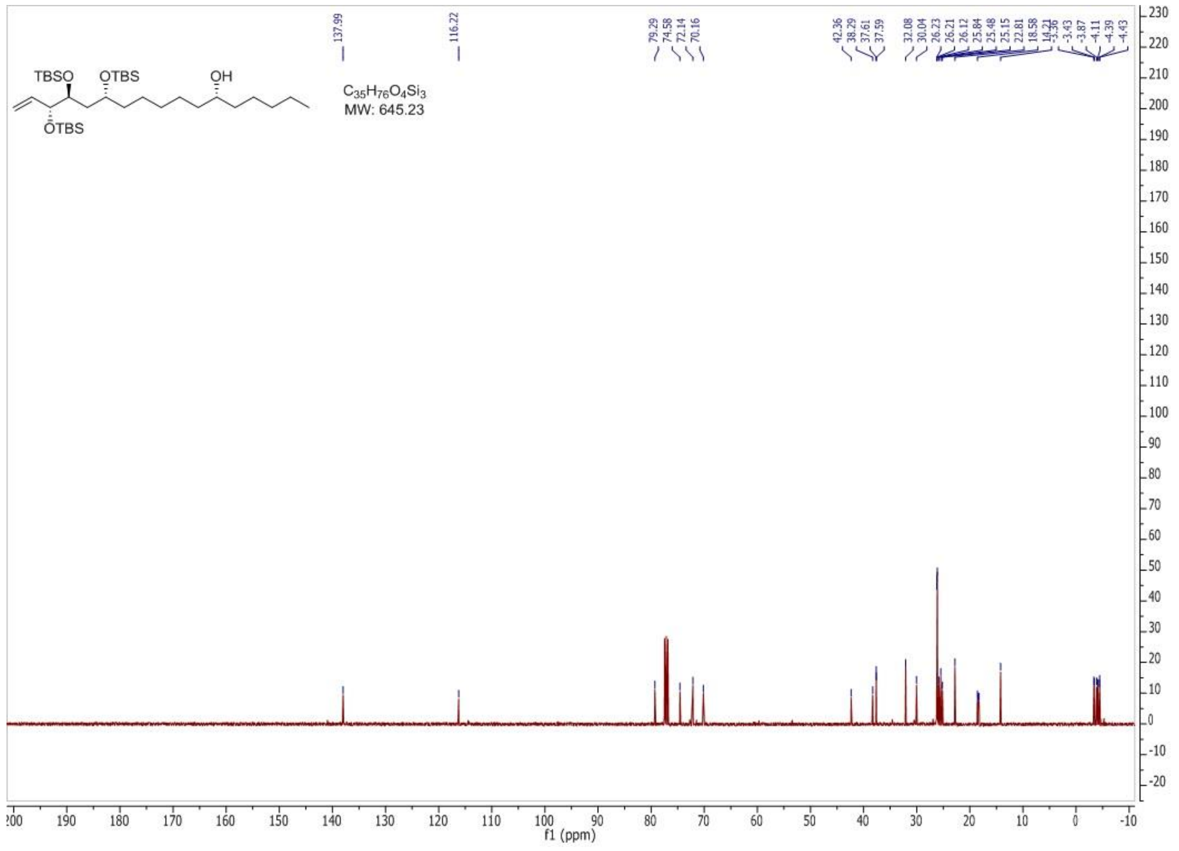

(4*R*,5*S*,7*R*,13*R*,*E*)-Methyl 4,5,7-tris[(tert-butyldimethylsilyl)oxy]-13-hydroxyoctadec-2-enoate (71)

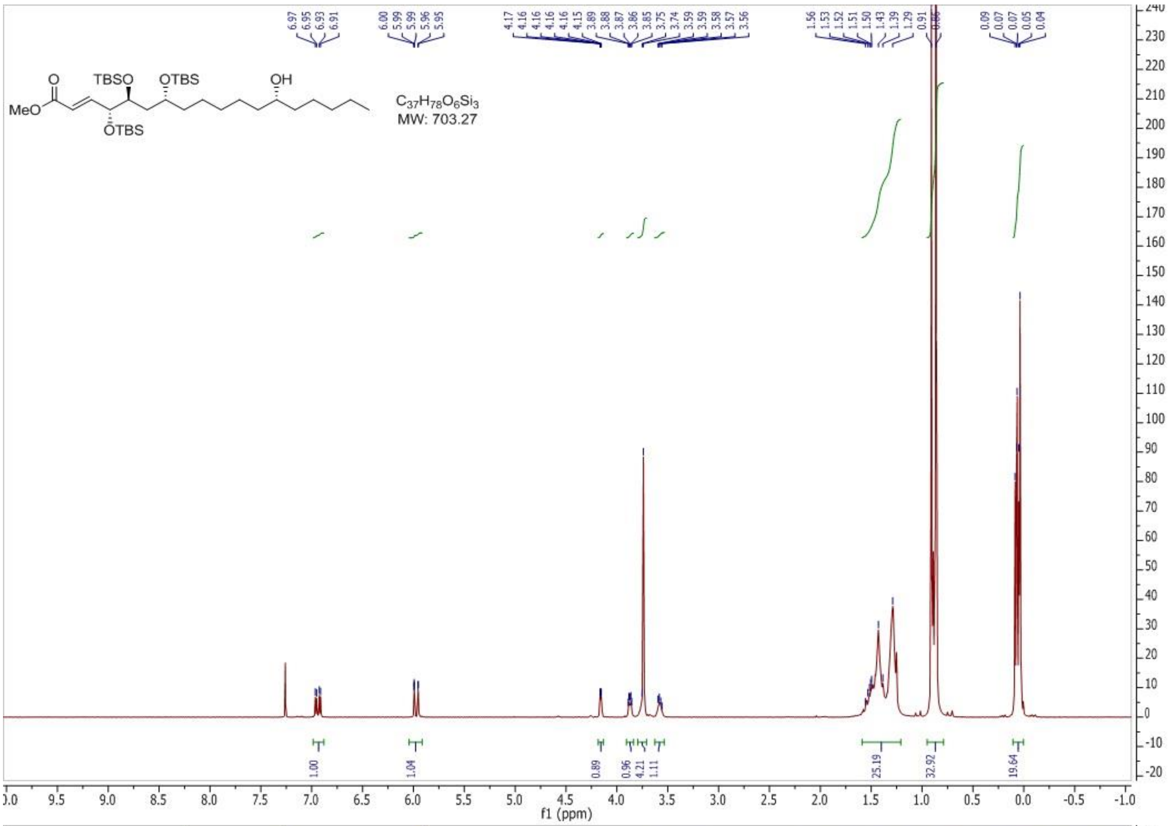

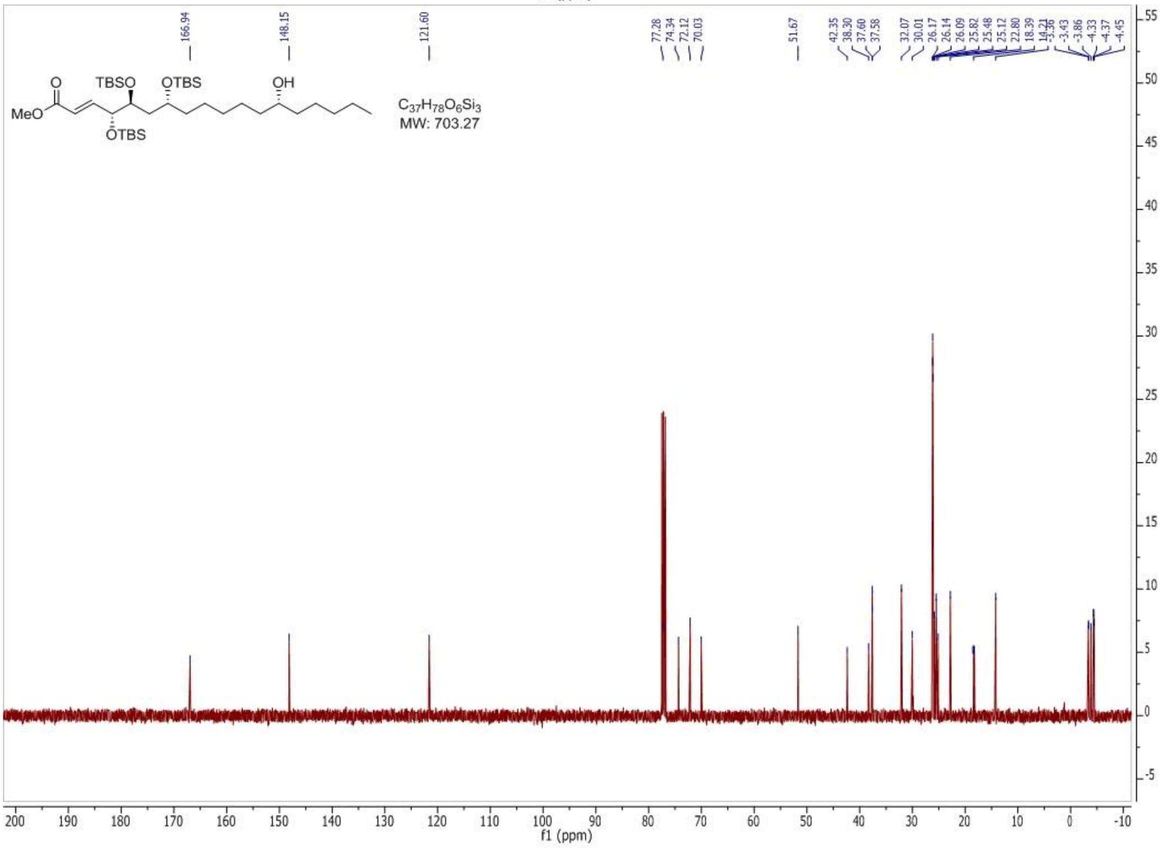

(4*R*,5*S*,7*R*,13*R*,*E*)-4,5,7-Tris[(tert-butyldimethylsilyl)oxy]-13-hydroxyoctadec-2-enoic acid (30)

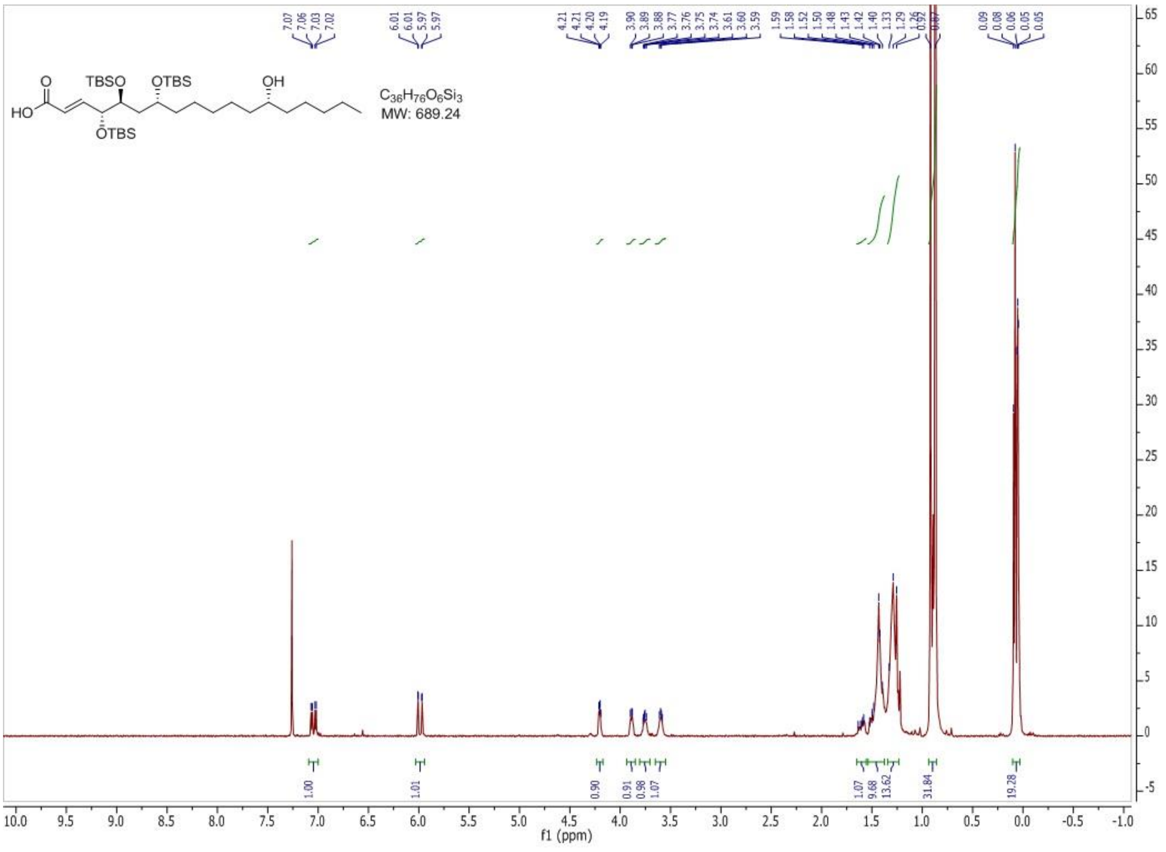

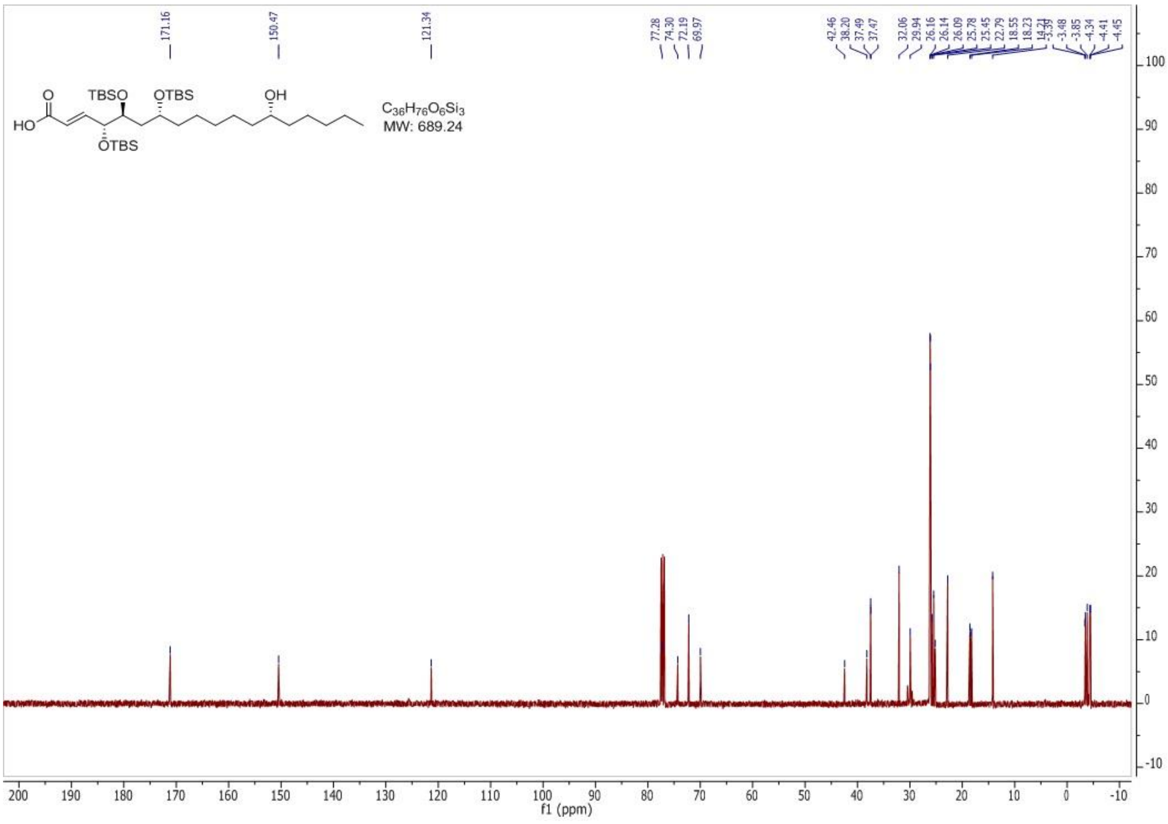

(5*R*,6*S*,8*R*,14*R*,*E*)-5,6,8-Tris[(tert-butyldimethylsilyl)oxy]-14-pentyloxacyclotetradec-3-en-2-one (72)

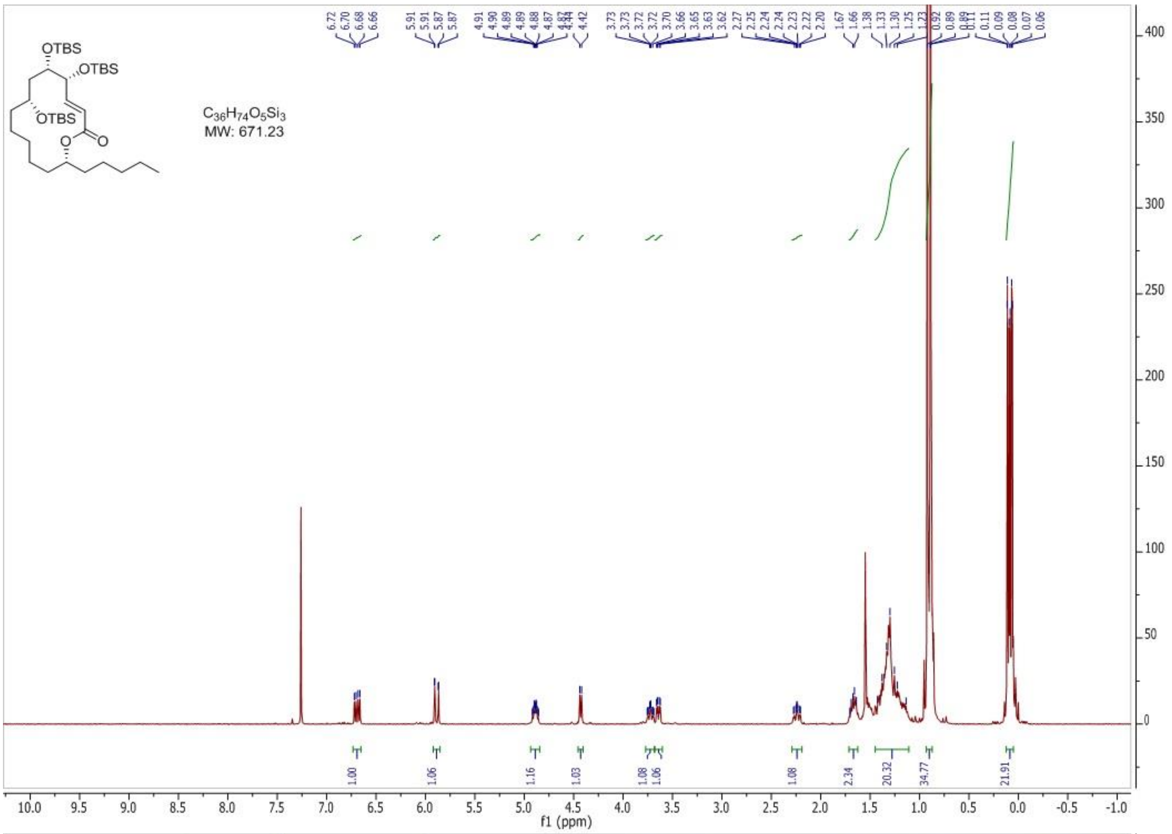

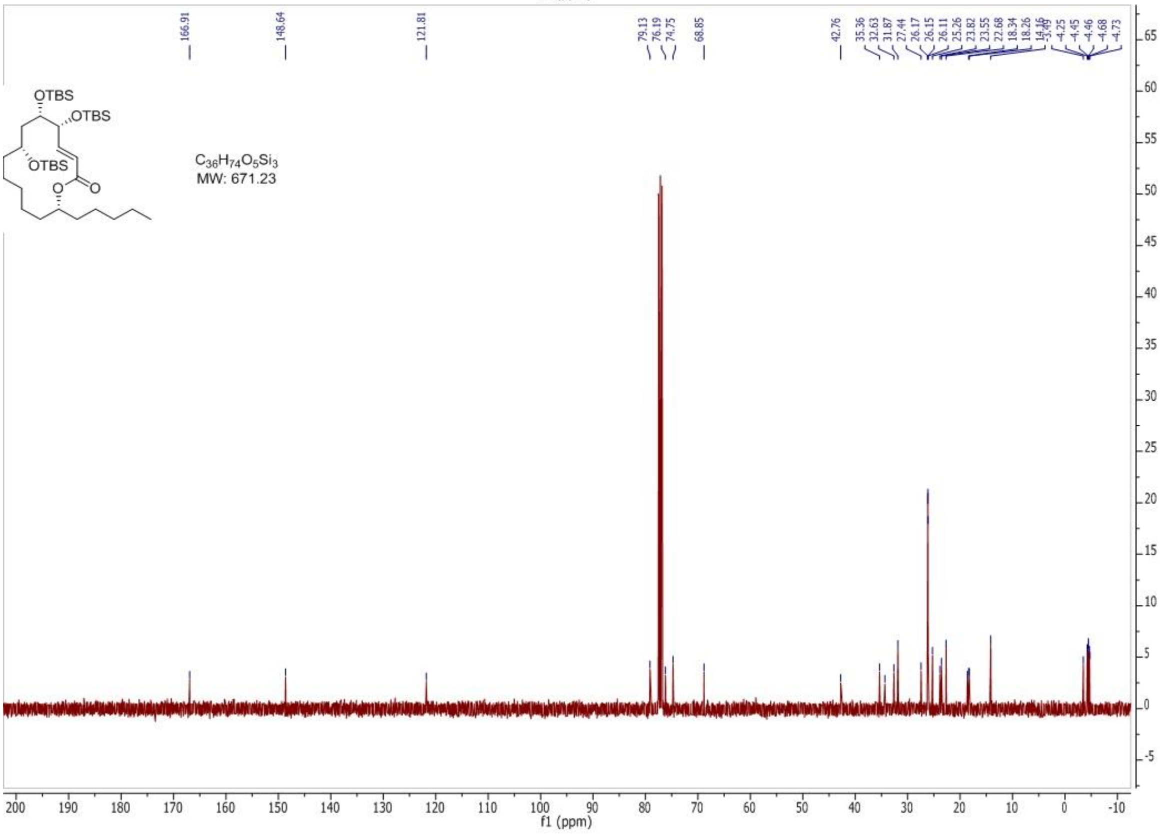

(5*R*,6*S*,8*R*,14*R*,*E*)-5,6,8-Trihydroxy-14-pentyloxacyclotetradec-3-en-2-one/ Sch 725674 (21)

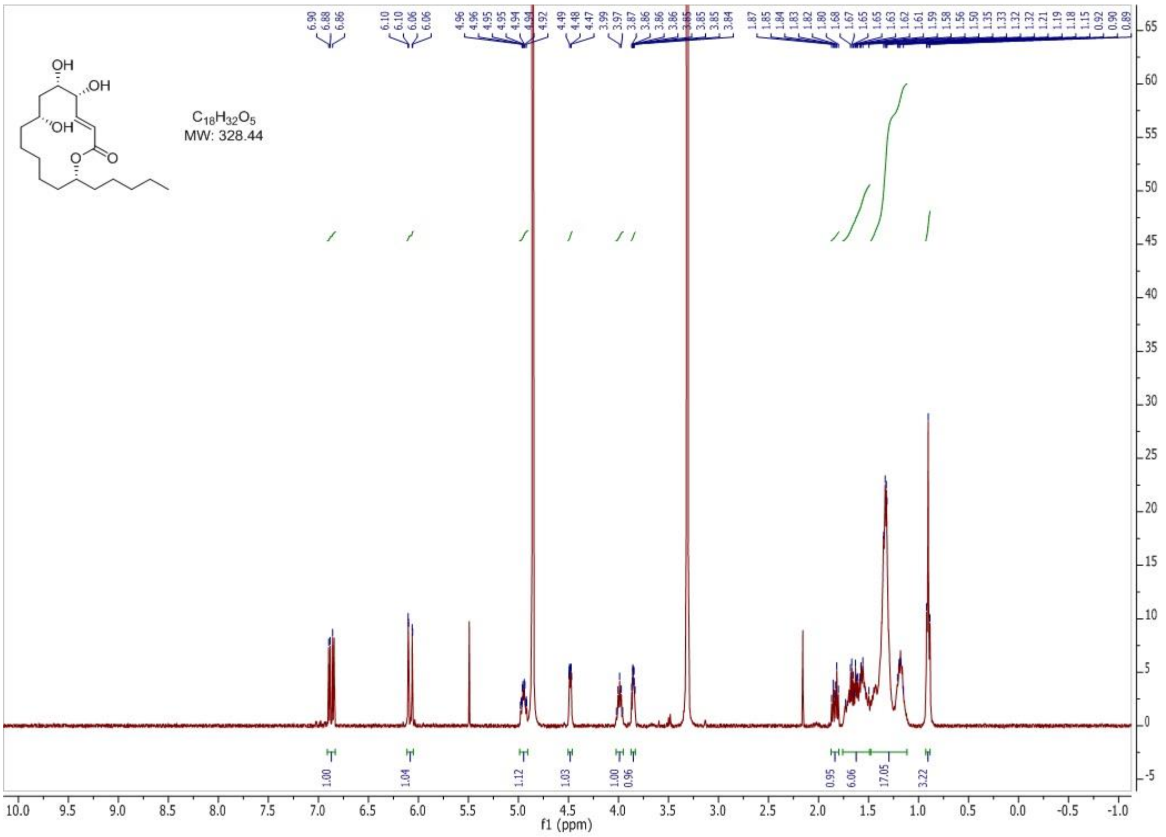

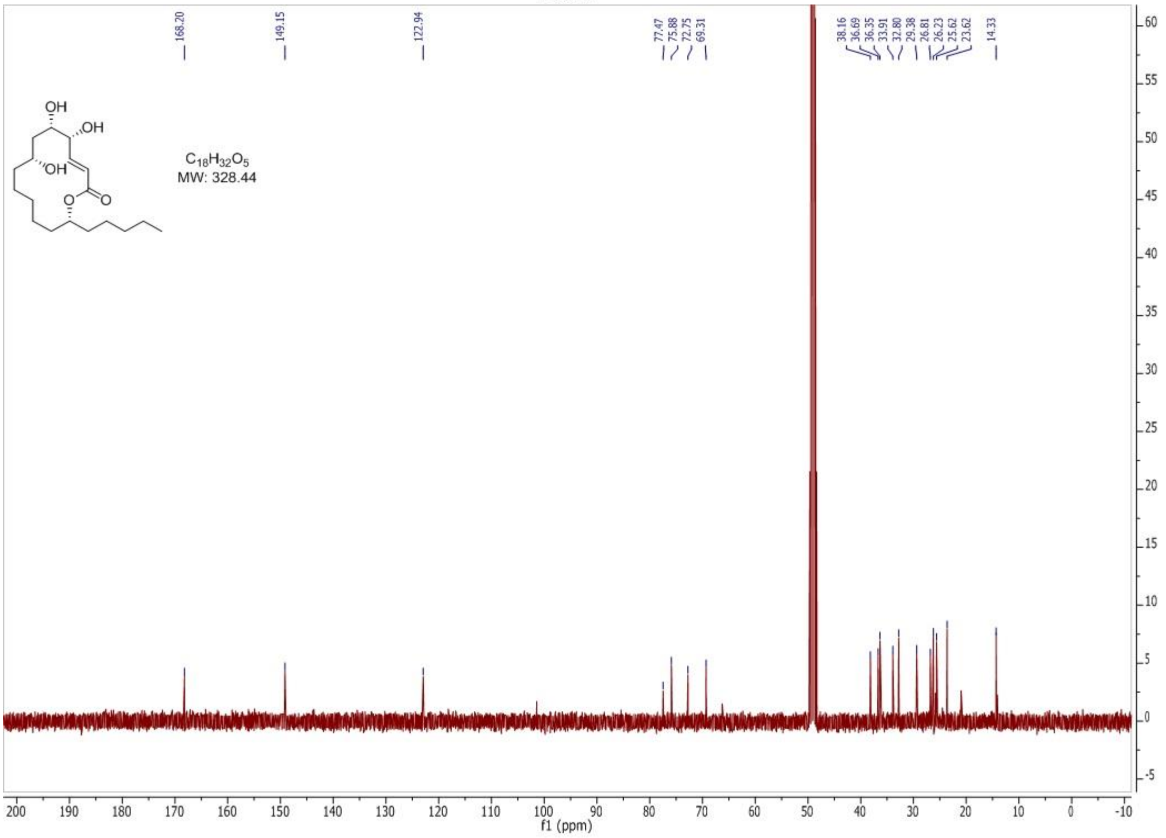

(2*S*,3*R*)-Pent-4-ene-1,2,3-triyl tris(4-bromobenzoate) (70)

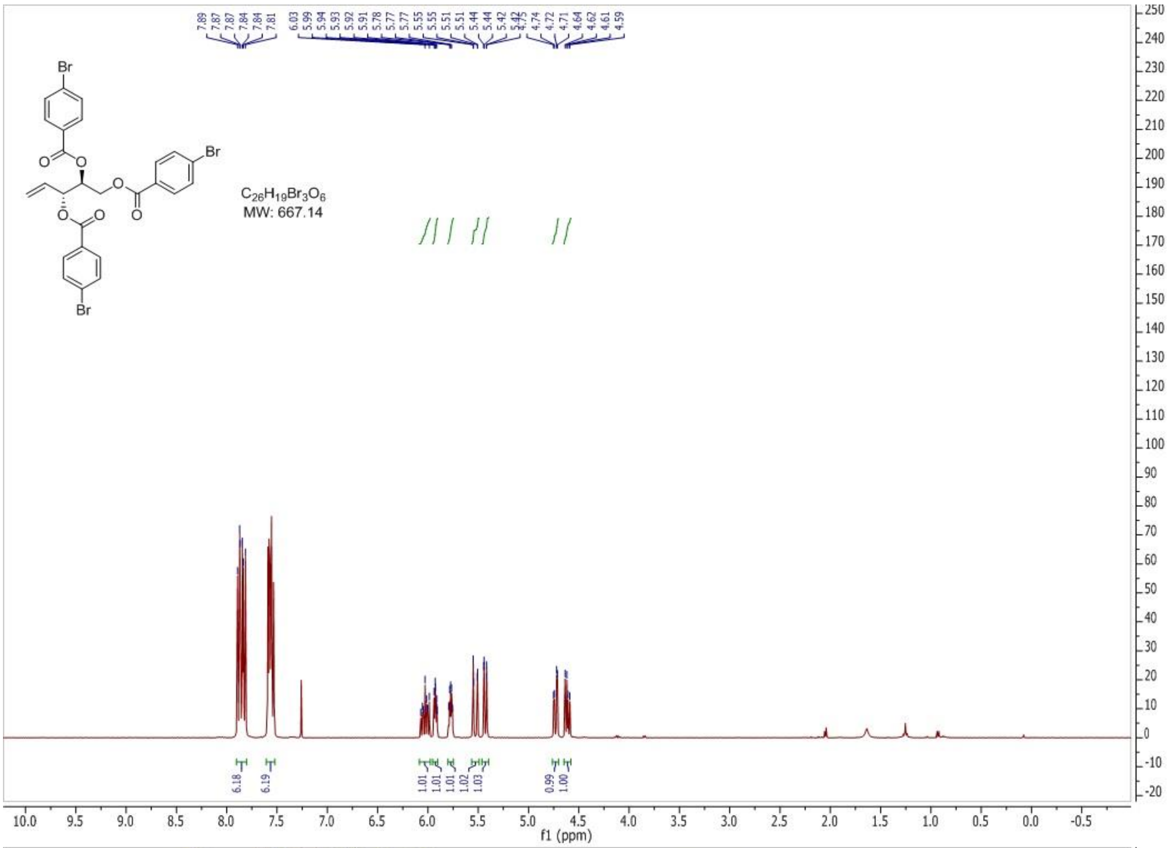

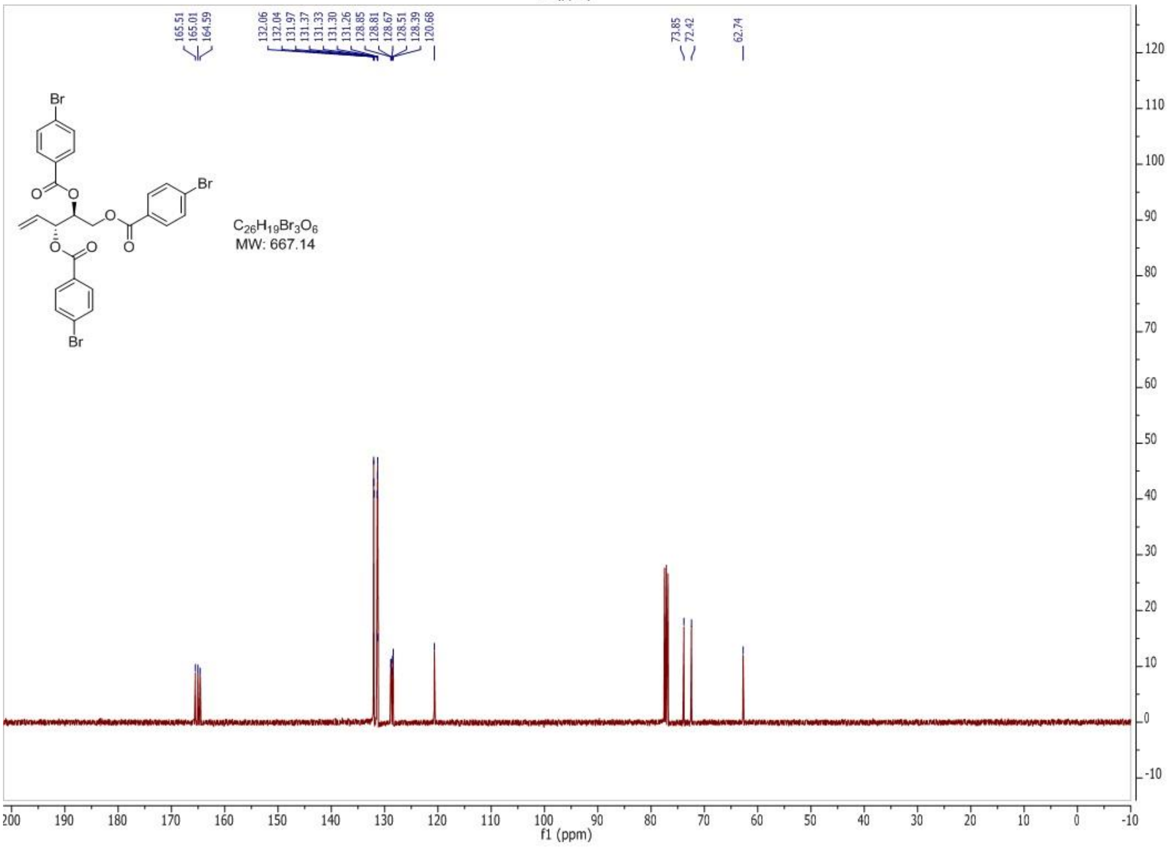

Supplement: Supplementary file 1 — Supplementary [file ANIE-55-14663-s001.pdf]
